# Aromatic C-H addition of ketones to imines enabled by manganese catalysis

**DOI:** 10.1038/s41467-017-01262-4

**Published:** 2017-10-27

**Authors:** Bingwei Zhou, Yuanyuan Hu, Ting Liu, Congyang Wang

**Affiliations:** 1 Beijing National Laboratory for Molecular Sciences, CAS Key Laboratory of Molecular Recognition and Function, CAS Research/Education Center for Excellence in Molecular Sciences, Institute of Chemistry, Chinese Academy of Sciences, Beijing, 100190 China; 2 University of Chinese Academy of Sciences, Beijing, 100049 China

## Abstract

Selectivity control of varied C–H bonds in a complex molecule is a long-standing goal and still a great challenge in C–H activation field. Most often, such selectivity is achieved by the innate reactivity of different C–H bonds. In this context, the classic Mannich reaction of acetophenone derivatives and imines is ascribed to the more reactive C(sp^3^)–H bonds α to the carbonyl, with the much less reactive aromatic C(sp^2^)–H bonds remaining intact. Herein we report an aromatic C(sp^2^)–H addition of ketones to imines enabled by manganese catalysis, which totally reverses the innate reactivity of C–H bonds α to the carbonyl and those on the aromatic ring. Diverse products of *ortho*-C–H aminoalkylated ketones, cyclized *exo*-olefinic isoindolines, and three-component methylated isoindolines can be successfully accessed under mild reaction conditions, which significantly expands the synthetic utilities of ketones as simple bulk chemicals.

## Abstract (web summary)

The Mannich reaction proceeds by functionalization of C(sp^3^)-H bonds alpha to carbonyls. Here, the authors suppress the Mannich reactivity of acetophenones in presence of a manganese catalyst and obtain valuable *o*-aminoalkylacetophenones and isoindolines via C(sp^2^)-H functionalization with imines.

## Introduction

**Fig. 1:**
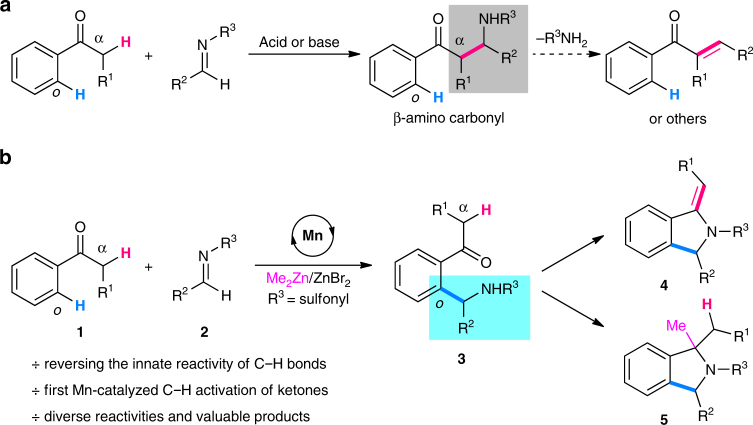
Innate and reversed reactivity of C–H bonds in ketones with imines. **a** The classic Mannich reaction of innately reactive α-C(sp^3^)–H bonds. **b** Mn-catalyzed *ortho*-C(sp^2^)–H addition by reversing the reactivity of C–H bonds (this work)

Ketones such as acetophenone are considered among the most easily accessible and practically useful building blocks in both laboratories and chemical industries. They undergo various transformations on the α-C–H bonds with a wide range of electrophiles, which now constitute an important chapter in many textbooks of organic chemistry. Among them, the Mannich reaction, enabling an addition of the α-C–H bond to an iminium ion or imine, has been known for a long time and represents one of the most classic reactions of ketones (Fig. 1a)^1–3^. It proceeds easily under either acidic or basic reaction conditions to afford the β-amino carbonyl and/or other derivatives. Of note, the C(sp^2^)–H bonds *ortho* to the carbonyl of ketones remain intact during this process, which shows that the reactivity of α-C–H bonds holds an absolute superiority over that of the *ortho*-C–H bonds on the benzene ring.

Recently, the directed C–H transformations of ketones^4,5^ have attracted immense attentions due to the prevalence of the carbonyl group in natural products, pharmaceuticals, and organic synthesis. Since the pioneering work of Ru-catalyzed aromatic C–H alkylation by Murai and others^6–10^, the ketone-directed C–H alkenylation^11–13^, arylation^14–16^, and amination^17–19^, among others^20–25^ have been elegantly demonstrated. Note that in most of these protocols the undesirable reactions on α-C–H bonds of ketones are not notorious by choosing suitable reaction partners. Moreover, these reactions have heavily relied on late transition metals (Ru, Rh, Pd, and Ir) so far. Developments of earth-abundant base metal catalyzed site-selective aromatic C–H transformations of ketones with more challenging imine electrophiles have not been reported yet. Despite of their huge synthetic interests, considerable challenges still remain in these processes, such as the formidably competitive Mannich and/or Aldol-type reactions of α-C–H bonds of ketones, the relatively inert reactivity of aromatic C–H bonds with a weakly coordinating ketone group^26^, and the lower catalytic reactivity of base metals compared with the precious ones. To address these issues, we resort to manganese-promoted C–H activation^27–43^, in which the stoichiometric cyclomanganation of ketones was shown by Kaesz and Nicholson as early as in 1975^44,45^. However, the manganese-catalyzed aromatic C–H transformations of ketones remain elusive.

Here, we describe, as our continuous interest in manganese catalysis^31–36^, a manganese-catalyzed site-selective aromatic C–H addition of ketones to imines under mild reaction conditions, while the conventional Mannich reaction is completely suppressed. Moreover, cyclized *exo*-olefinic isoindoline and three-component methylated isoindoline derivatives can be selectively obtained. Thus, such diverse reactivity provides a straightforward and efficient way to access varied functionalized isoindolines from simple ketones and imines.

## Results

### Optimization of Reaction conditions

**Fig. 2:**
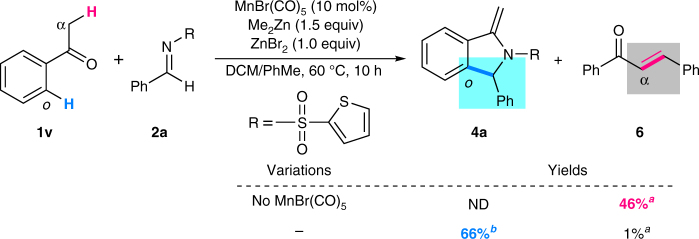
Evaluating the C–H bond selectivity of ketones by manganese-based catalytic system. ^a^GC-MS yield. ^b^Isolated yield. *DCM* dichloromethane, *ND* not detected

As shown in Fig. 1b, we intended to develop a manganese-catalyzed aromatic C–H addition of ketones to imines. At the outset, in order to simplify the reaction outcome we chose *t*-butyl phenyl ketone **1a** and imine **2a** as model substrates to screen the reaction parameters (see Supplementary Table 1 for more details). The optimal reaction conditions were obtained by using MnBr(CO)_5_ as a catalyst, Me_2_Zn/ZnBr_2_ as promoters in the solvent of 1,2-dichloroethane (DCE) at 60 °C. We then evaluated the reaction chemoselectivity by using acetophenone **1v** bearing α-C–H bonds as a substrate which was commonly used in the Mannich reaction (Fig. 2). Interestingly, when the reaction was carried out in the absence of MnBr(CO)_5_, the Mannich reaction took place overwhelmingly followed by elimination of an amine of the β-amino carbonyl intermediate to afford chalcone **6** in 46% gas chromatography–mass spectrometry (GC-MS) yield. In a sharp contrast, under the manganese catalysis product **4a** resulting from aromatic C–H addition/cyclization/elimination was obtained in 66% isolated yield. Remarkably, this represents a reversal of the usual reactivity between *ortho* C(sp^2^)–H bonds and α-C(sp^3^)–H bonds of ketones with imines achieved by using a transition metal catalyst.

### Investigations on substrate scopes

**Fig. 3:**
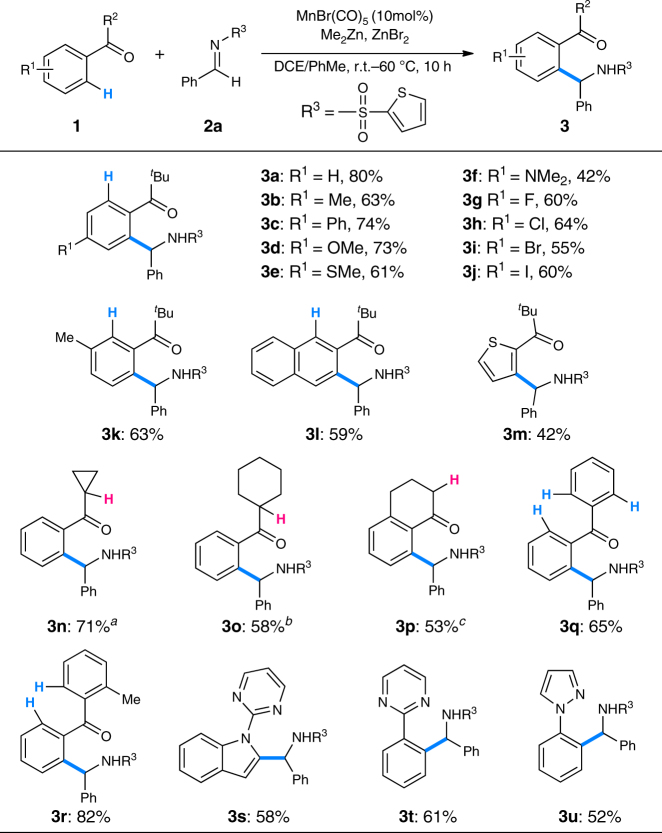
Scope of ketones for the *mono*-C–H addition reaction. Reaction conditions: **1** (1.5 mmol), **2a** (0.5 mmol), MnBr(CO)_5_ (0.05 mmol), Me_2_Zn (0.75 mmol, 1.2 M in toluene), ZnBr_2_ (0.5 mmol), DCE (0.4 M), 60 °C, 10 h. ^a^ **1n** (2.0 mmol), DCM (0.1 M), r.t., 16 h. ^b^ **1o** (2.0 mmol), DCM (0.1 M), r.t., 1 h. ^c^ **1p** (2.0 mmol), DCM (0.1 M), 40 °C, 1 h. *DCE* 1,2-dichloroethane, *DCM* dichloromethane

With the optimized conditions in hand, the scope of ketones was first explored (Fig. 3). Aromatic ketones bearing a wide range of electronically varied functional groups on the benzene ring delivered the corresponding aromatic C–H addition products successfully (**3a–j**). Ketones containing two sterically biased C–H bonds reacted with imine **2a** at the less hindered positions exclusively giving products **3k** and **3l** respectively. Heteroaromatic ketone 2,2-dimethyl-1-(thiophen-2-yl)propan-1-one **1m** was also a viable substrate affording the expected product **3m** in synthetically useful yield. Replacing the *t*-butyl group of **1a** by other alkyl groups bearing α-C–H bonds, the reaction worked as well even at room temperature or 40 °C leading to the expected products smoothly (**3n–p**). Of note, no Mannich-type products were detected in these reactions and the carbonyl-remaining products provide a handle for further synthetic elaborations. Importantly, benzophenone **1q** and phenyl(*o*-tolyl)methanone **1r** were also suitable substrates giving the *mono*-C–H addition products in good yields (**3q**, **3r**). In addition, arenes and heteroarenes bearing nitrogen-containing directing groups could also undergo the corresponding C–H aminoalkylation reaction with the current reaction conditions (**3s–u**).^46–51^ Of note, Ackermann has elegantly disclosed the related C–H aminoalkylation of indoles with imines in the absence of zinc additives at higher tempreture.^40^

**Fig. 4:**
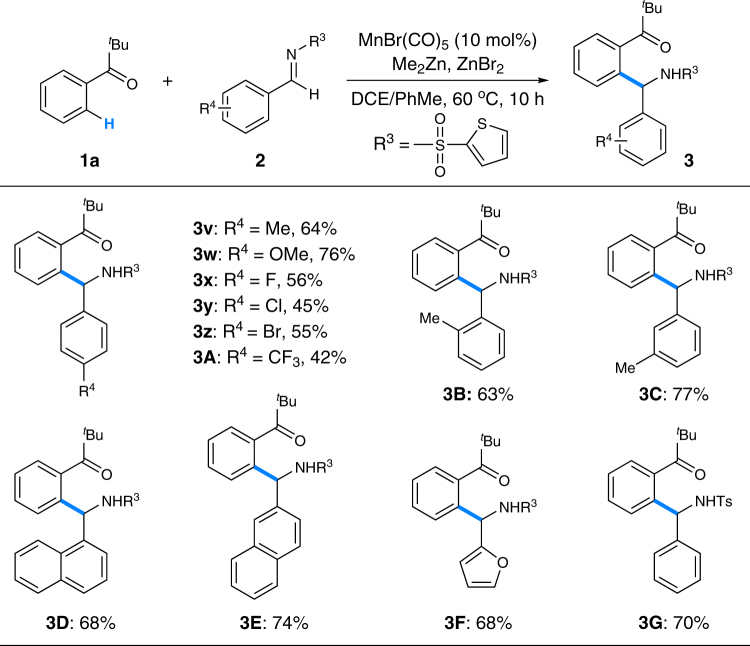
Scope of imines for the *mono*-C–H addition reaction. Reaction conditions: **1a** (1.5 mmol), **2** (0.5 mmol), MnBr(CO)_5_ (0.05 mmol), Me_2_Zn (0.75 mmol, 1.2 M in toluene), ZnBr_2_ (0.5 mmol), *DCE* (0.4 M), 60 °C, 10 h. *DCE* 1,2-dichloroethane

Next, the scope of imines was surveyed with ketone **1a** as the model substrate (Fig. 4). Both electron-donating and electron-withdrawing groups were well tolerated in the reaction with the former ones giving relatively higher yields of the aromatic C–H addition products (**3v-A**). *Ortho-* and *meta-*substituents on the benzene ring of imines showed comparable effect on the reaction yields (**3v** vs. **3B**, **3C**). Naphthyl imines with extended conjugation delivered the expected products smoothly (**3D**, **3E**). It seemed that the steric hindrance had limited influence on the reaction outcome (**3B**, **3D**). Heteroaromatic imine and *p*-tolylsulfonyl imine were also amenable to this protocol (**3F**, **3G**). Unfortunately, aliphatic imines failed to afford the corresponding products under the reaction conditions.

**Fig. 5:**
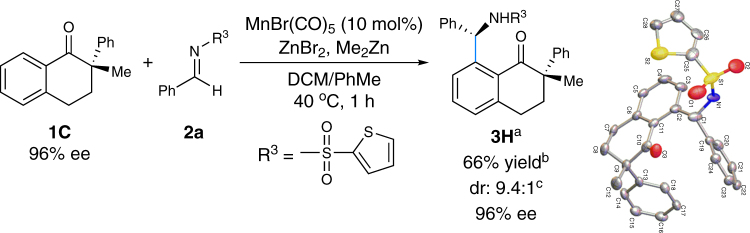
A diastereoselective *mono*-C–H addition reaction using chiral ketone **1 C**. ^a^The major diastereo-isomer was shown. ^b^Combined isolated yield. ^c^Determined by ^1^H NMR analysis of the crude product. *DCM* dichloromethane

When a chiral ketone, **1C** of 96% ee, was used as a substrate and treated with imine **2a** under the similar reaction conditions, the corresponding *ortho*-aminoalkylated product **3H** was isolated in 66% yield with a dr value of 9.4:1 (Fig. 5). The major diastereo-isomer of **3H** was in 96% ee, which reflected the *ee* value of ketone **1C**. Furthermore, the structural configuration of the major diastereo-isomer was confirmed by single-crystal X-ray diffraction analysis.

**Fig. 6:**
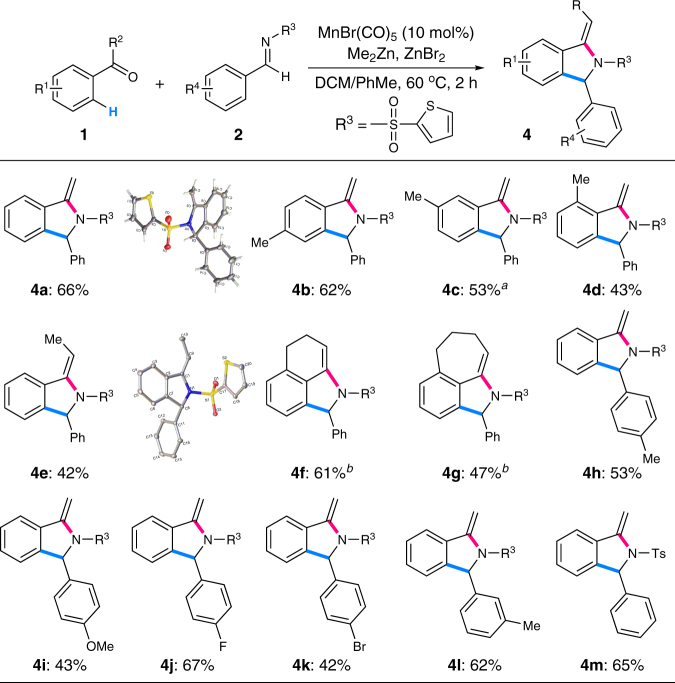
Substrate scope for the [3 + 2] annulations giving *exo*-olefinic isoindolines **4**. Reaction conditions: **1** (2.0 mmol), **2** (0.5 mmol), MnBr(CO)_5_ (0.05 mmol), Me_2_Zn (0.75 mmol, 1.2 M in toluene), ZnBr_2_ (0.5 mmol), DCM (0.1 M), 60 °C, 2 h. ^a^Combined yield of two regioisomers (3.9/1), major isomer **4c** was shown. ^b^Me_2_Zn (2.0 equiv.), 100 °C, 10 h. *DCM* dichloromethane

Interestingly, the *exo*-olefinic isoindoline products **4** could be selectively obtained from the reactions of imines and aryl alkyl ketones bearing α-C–H bonds by slightly tuning the reaction conditions (Fig. 6). Specifically, acetophenone **1v** was treated with imine **2a** at 60 °C for 2 h under the otherwise same conditions giving *exo*-olefinic isoindoline **4a** in 66% isolated yield. The structure of **4a** was unambiguously confirmed by single-crystal X-ray diffraction analysis. Introducing a methyl group into the *para*, *meta*, or *ortho* position of acetophenone resulted in the formation of the expected products successfully (**4b–d**). Remarkably, the reaction of propiophenone with imine **2a** provided exclusively isoindoline **4e** with an *E*-configuration of the *exo*-cyclic C = C bond, which was again unambiguously confirmed by single-crystal X-ray diffraction analysis. The steric compulsion between the methyl group and (2-thienyl)sulfonyl group might account for the observed configuration of the double bond. 1-Tetralone and 1-benzosuberone were also proved to be suitable substrates affording the corresponding tricyclic products successfully under the slightly modified reaction conditions (**4f**, **4g**). A series of imines bearing electronically varied functional groups were applicable to this reaction leading to the expected *exo*-olefinic isoindoline products smoothly (**4h–l**). *p*-Tolylsulfonyl imine was again susceptible to the reaction conditions giving the corresponding product in comparable yield (**4m**).

**Fig. 7:**
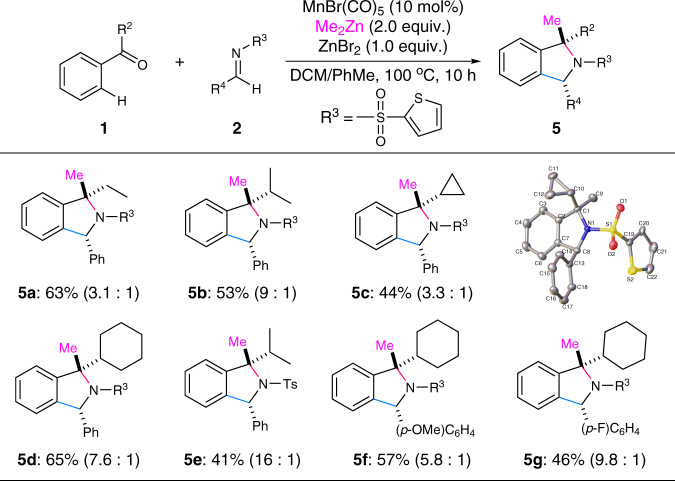
Substrate scope for the three-component reaction giving isoindolines **5**. Reaction conditions: **1** (2.0 mmol), **2** (0.5 mmol), MnBr(CO)_5_ (0.05 mmol), Me_2_Zn (2.0 mmol, 1.2 M in toluene), ZnBr_2_ (0.5 mmol), DCM (0.1 M), 100 °C, 10 h. The ratio of diastereoisomers (dr) was shown in parentheses. *DCM* dichloromethane

During our further investigations on the reaction parameters, we surprisingly found that a three-component reaction of ketone, imine, and dimethylzinc could be achieved simply by utilizing two equivalents of dimethylzinc at an elevated temperature under the otherwise same conditions (Fig. 7). Thus, a range of isoindolines bearing a *tetra*-substituted carbon center could be easily accessed from simple ketones and imines with moderate to good diastereoselectivity (**5a–g**). The structures of the major *cis*-diastereoisomers **5c** and **5d** were both confirmed by single-crystal X-ray diffraction analysis. It should be noted that the use of *t*-butyl phenyl ketone **1a** could not afford the corresponding three-component product presumably due to the increased steric hindrance of the congested *tetra*-substituted carbon center.

### Mechanistic studies

**Fig. 8:**
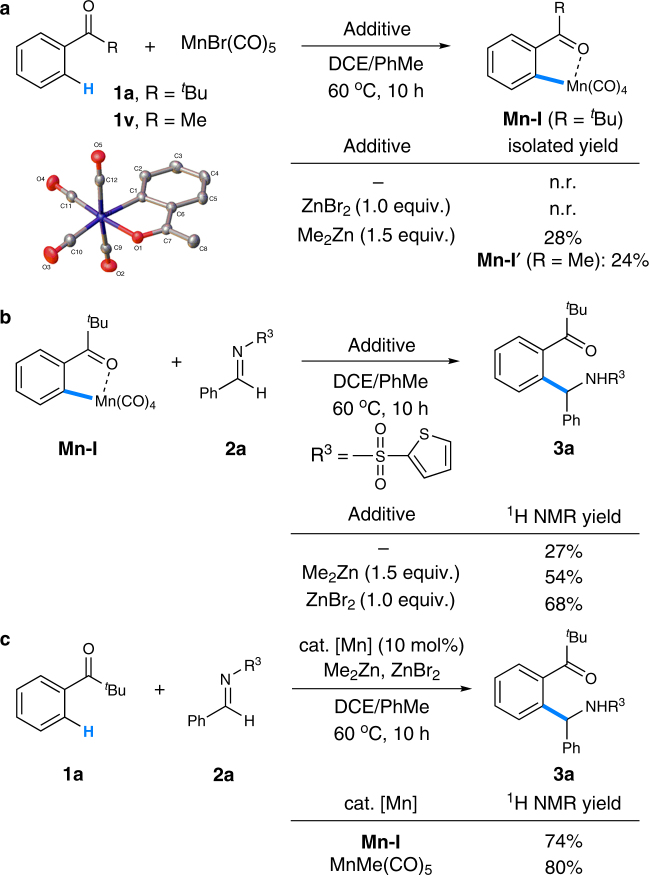
Mechanistic experiments. **a** Isolation of key intermediates **Mn-I** and **Mn-I′**. **b** Stoichiometric reactions of **Mn-I** and imine **2a**. **c** Reactions using **Mn-I** and MnMe(CO)_5_ as a catalyst. *DCE* 1,2-dichloroethane

To clarify the possible reaction pathways, a range of mechanistic experiments were conducted. First, the stoichiometric reaction of ketone **1a** with MnBr(CO)_5_ was examined and no product was detected (Fig. 8a). While no reaction occurred with the assistance of ZnBr_2_, the addition of Me_2_Zn to the reaction resulted in the formation of five-membered manganacycle **Mn-I** in 28% isolated yield. Also, enolizable acetophenone **1v** could delivered the corresponding manganacycle **Mn-I′** in comparable yield, whose structure was confirmed by single-crystal X-ray diffraction analysis. MnMe(CO)_5_, generated in situ from the transmetalation of MnBr(CO)_5_ with Me_2_Zn, might play a critical role in the step of C–H bond cleavage^35^. Second, treatment of **Mn-I** with imine **2a** afforded the C–H addition product **3a** in 27% ^1^H NMR yield (Fig. 8b). The reaction yields could be further improved by adding either Me_2_Zn or ZnBr_2_. Finally, the reactions of ketone **1a** and imine **2a** using manganacycle **Mn-I** or MnMe(CO)_5_ as a catalyst were examined and the corresponding product **3a** was formed in 74 and 80% yield, respectively (Fig. 8c). These results suggested that both the manganacycle **Mn-I** and MnMe(CO)_5_ might be the key intermediates in the reaction.

**Fig. 9:**
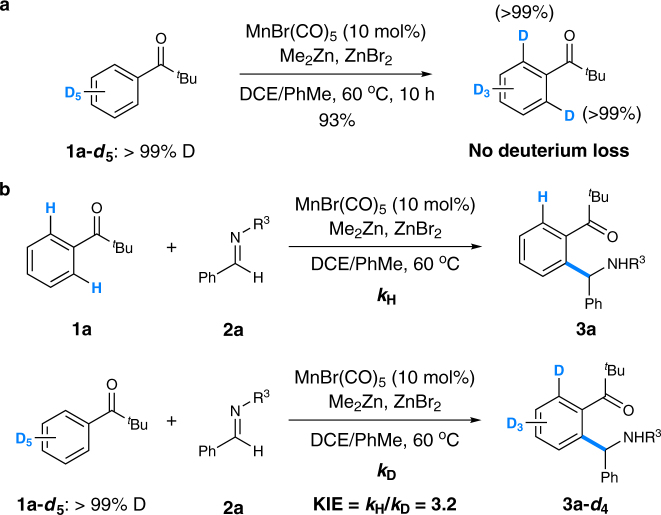
Deuterium-labeling experiments. **a** Probing the reversibility of the C–H bond cleavage. **b** Probing the kinetic isotope effect. *DCE* 1,2-dichloroethane

Furthermore, deuterium-labeling experiments were carried out in order to probe the nature of the C–H bond cleavage. First, *tert*-butyl(pentadeuteriophenyl)methone **1a-** ***d***_***5***_ was prepared and then subjected to the reaction conditions (Fig. 9a). No loss of deuterium was observed at the *ortho* positions of **1a-** ***d***_***5***_, which suggested an irreversible C–H bond cleavage step in the reaction. Next, two parallel reactions of **1a** and **1a-** ***d***_***5***_ with imine **2a** respectively were conducted (Fig. 9b). As a result, a kinetic isotope effect (KIE) value of 3.2 implied the C–H bond cleavage might be involved in the turnover-limiting step or in a prior step with a lower activation barrier^52^.

**Fig. 10:**
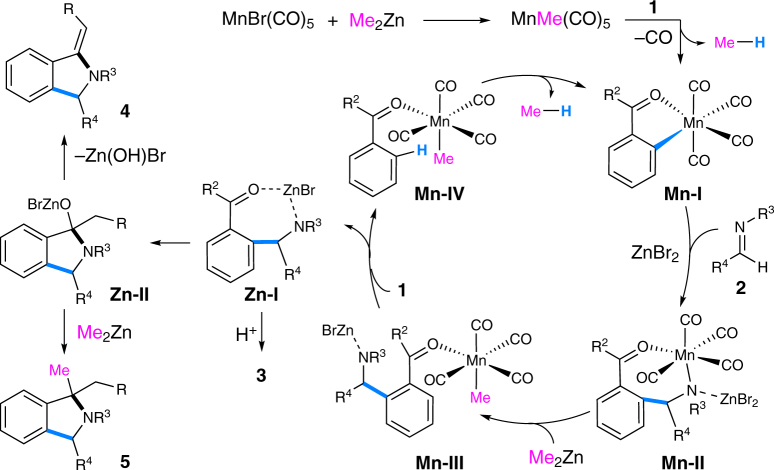
A proposed reaction mechanism. Key steps include formation of **Mn-I** followed by addition to imine yielding **Mn-II**, transmetalation of **Mn-II** with Me_2_Zn giving **Mn-III**, then producing **Mn-IV** and **Zn-I** by a ligand exchange with **1**, and C–H activation of **Mn-IV** regenerating **Mn-I**. Intramolecular cyclization of **Zn-I** yields **Zn-II** followed by either an elimination giving **4** or an intermolecular nucleophilic substitution with Me_2_Zn forming **5**

Based on the above results and literature clues^35,43,53^, a plausible reaction mechanism was depicted in Fig. 10. The reaction starts with the formation of MnMe(CO)_5_ from MnBr(CO)_5_ and Me_2_Zn. It further reacts with ketone **1** to give five-membered manganacycle **Mn-I** followed by addition to imine **2** yielding seven-membered manganacycle **Mn-II**. Transmetalation of **Mn-II** with Me_2_Zn affords intermediate **Mn-III**, which undergoes a ligand exchange with substrate **1** to produce species **Mn-IV** and **Zn-I**. An intramolecular C–H activation occurs in **Mn-IV** regenerating **Mn-I** and releasing methane^35^. Hydrolysis of **Zn-I** gives product **3**. Meanwhile, **Zn-I** may also undergo an intramolecular cyclization to yield intermediate **Zn-II**, which is followed by either an elimination of zinc salt giving *exo*-olefinic isoindoline **4** or an intermolecular nucleophilic substitution with Me_2_Zn forming isoindoline **5** under well-controlled reaction conditions.

## Discussion

In conclusion, aromatic C–H addition of ketones to imines was developed via manganese catalysis, which enabled to reverse the reactivity of labile C(sp^3^)–H bonds α to the carbonyl and inert C(sp^2^)–H bonds on the benzene ring of ketones. Thus, the classic Mannich reaction was completely depressed and a series of valuable products, namely the *ortho*-C–H aminoalkylated ketones, cyclized *exo*-olefinic isoindolines, and three-component methylated isoindolines, can be selectively achieved. Meanwhile, this protocol also represents a manganese-catalyzed aromatic C–H bond transformation of ketones since the parent stoichiometric cyclomanganation reaction was reported in 1975^44,45^. Further explorations on the manganese-catalyzed C–H activation reactions of ketones are underway in our laboratory.

## Methods

### General procedure for the formation of products **3**

To a 25 ml flame-dried Schlenk tube was added ZnBr_2_ (0.5 mmol, 112.5 mg, stored in glove box), MnBr(CO)_5_ (0.05 mmol, 10.0 mol%, 13.8 mg), DCE (1.25 mL), 2,2-dimethyl-1-phenylpropan-1-one **1a** (1.5 mmol, 243.0 mg), (*E*)-*N*-benzylidenethiophene-2-sulfonamide **2a** (0.5 mmol, 125.5 mg), and Me_2_Zn (0.75 mmol, 1.2 M in toluene, 0.625 mL) sequentially under nitrogen. The tube was sealed and stirred at 60 °C for 10 h. After completion, the reaction mixture was diluted with ethyl acetate (5.0 mL) and filtered through a short pad silica gel washing with ethyl acetate (20 mL). The filtrate was concentrated and purified by silica gel column chromatography to provide the product **3a** in 80% yield.

### General procedure for the formation of products **4**

To a Schlenk tube was added ZnBr_2_ (0.5 mmol, 112.5 mg), MnBr(CO)_5_ (0.05 mmol, 10.0 mol%, 13.8 mg), DCM (5.0 mL), acetophenone **1v** (2.0 mmol, 240.0 mg), (*E*)-*N*-benzylidene thiophene-2-sulfonamide **2a** (0.5 mmol, 125.5 mg), and Me_2_Zn (0.75 mmol, 1.2 M in toluene, 0.625 mL) sequentially under nitrogen. The tube was sealed and stirred at 60 °C for 2 h. After completion, the reaction mixture was diluted with ethyl acetate (10 mL) and filtered through a short pad silica gel washing with ethyl acetate (20 mL). The filtrate was concentrated and purified by silica gel column chromatography to provide **4a** in 66% yield.

### General procedure for the formation of products **5**

To a Schlenk tube was added ZnBr_2_ (0.5 mmol, 112.5 mg), MnBr(CO)_5_ (0.05 mmol, 10.0 mol%, 13.8 mg), DCM (5.0 mL), propiophenone **1z** (2.0 mmol, 276.0 mg), (*E*)-*N*-benzylidenethiophene-2 -sulfonamide **2a** (0.5 mmol, 125.5 mg), and Me_2_Zn (1.0 mmol, 1.2 M in toluene, 0.83 mL) sequentially under nitrogen. The tube was sealed and stirred at 100 °C for 10 h. After completion, the reaction mixture was diluted with ethyl acetate (10 mL) and filtered through a short pad silica gel washing with ethyl acetate (20 mL). The filtrate was concentrated and purified by silica gel column chromatography to provide **5a** in 63% yield (dr = 3.1:1).

### Data availability

All data supporting the findings of this study are available within the article and its Supplementary Information file or from the authors on reasonable request.

Supplementary crystallographic information files, which include structure factors, have been deposited with the Cambridge Crystallographic Data Centre (CCDC) as deposition numbers CCDC 1563929, **3H**; CCDC: 1532722, **4a**; CCDC: 1532723, **4e**; CCDC: 1532725, **5c**; CCDC: 1532724, **5d**; CCDC 1563930, **Mn-I′**. These data files can be obtained free of charge from http://www.ccdc.cam.ac.uk/data_request/cif.

## Electronic supplementary material

Supplementary Information
