# Supplementary material for: Aromatic C-H addition of ketones to imines enabled by manganese catalysis
Source: Nat Commun. 2017 Oct 27;8:1169. doi: 10.1038/s41467-017-01262-4 (PMC5660118; doi:10.1038/s41467-017-01262-4)
Supplement: Supplementary file 1 — Supplementary Information [file 41467_2017_1262_MOESM1_ESM.pdf]

Supplementary Methods

General Information

Unless otherwise noted, all reactions were carried out in flame-dried reaction vessels with Teflon screw caps under nitrogen. Solvents were purified and dried according to standard methods prior to use. All commercially available reagents were obtained from chemical suppliers and used after proper purification if necessary. Flash column chromatography was performed on silica gel (200-300 mesh) with the indicated solvent mixtures. TLC analysis was performed on pre-coated, glass-backed silica gel plates and visualized with UV light.

The ^{1}H NMR and ^{13}C NMR spectra were recorded on a Bruker 300 AV, 400 AV or 500 AV spectrometers. ^{19}F NMR spectra was recorded on a Bruker 500 AV. Chemical shifts (δ) were reported as parts per million (ppm) downfield from tetramethylsilane and the following abbreviations were used to identify the multiplicities: s = singlet, d = doublet, t = triplet, q = quartet, m = multiplet, dd = doublet of doublets, dt = doublet of triplets, dq = doublet of quartets, br = broad and all combinations thereof can be explained by their integral parts. Coupling constant (*J*) was reported in hertz unit (Hz). GC yield was performed on a SHIMADZU QP-2010SE GC-MS spectrometer. The enantiomeric excesses were determined by HPLC analysis on Chiral Daicel Chiralpak AD-H or ID. The separation of diastereoisomers was performed on a preparative HPLC LC 3000 spectrometer. The high resolution mass spectra (HRMS) were recorded on a Thermo Scientific Exactive spectrometer.

General procedure for the preparation of ketones

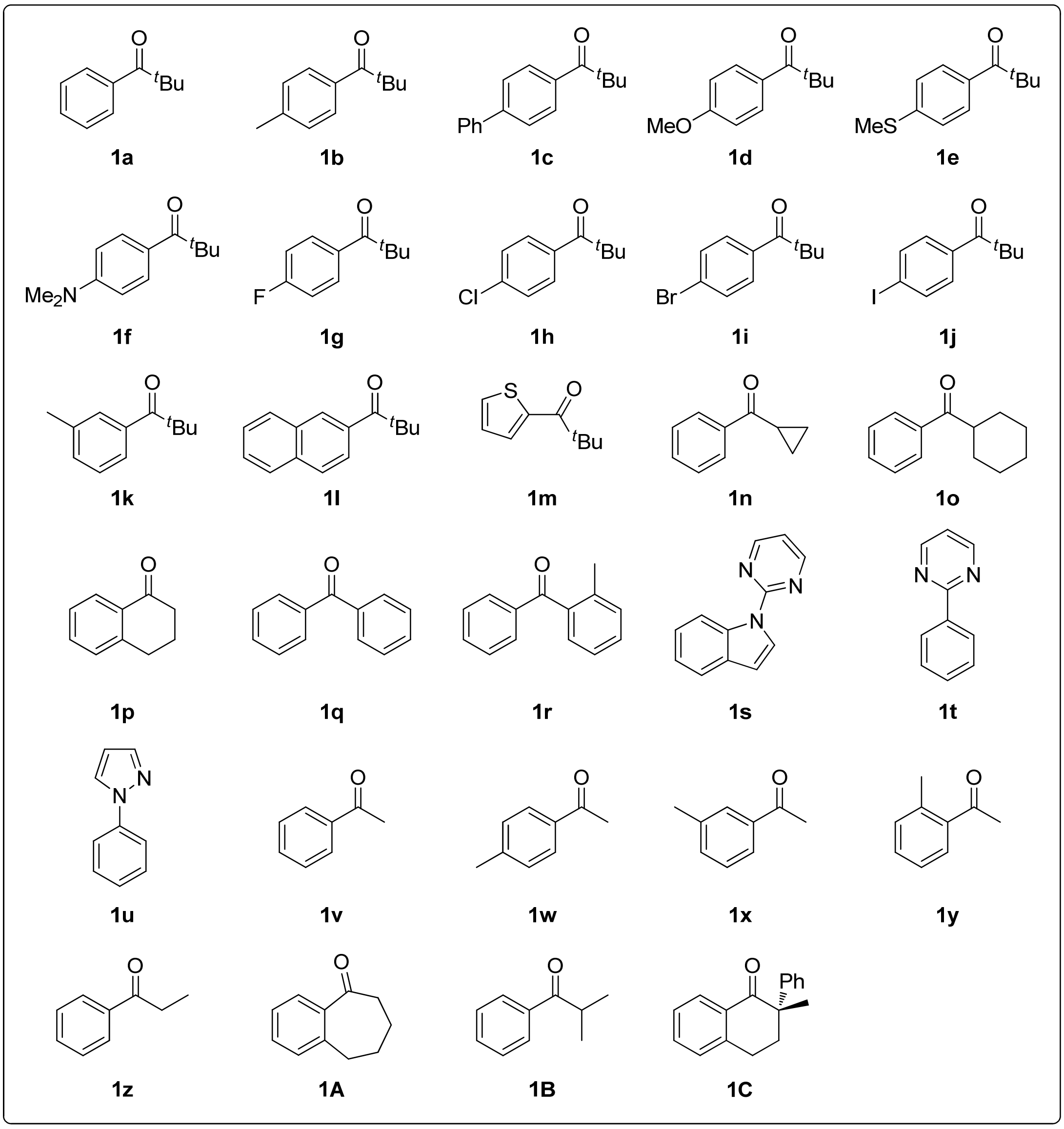

Supplementary Figure 1. Scope of ketones

Substrates **1n**, **1o**, **1p**, **1q**, **1u**, **1v**, **1w**, **1x**, **1y**, **1z**, **1A**, **1B** are commercially available. Other substrates are prepared according to known literatures.

General procedure A:[1]

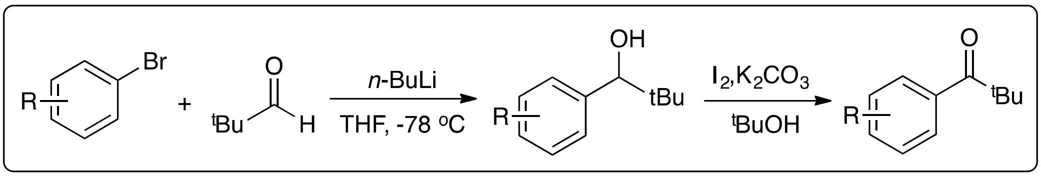

Supplementary Figure 2. Synthesis of ketones from aldehyde

To a solution of aryl bromide (20 mmol) in THF (30 mL) was added drop-wise *n*-butyllithium (22 mmol, 2.5 M in hexane) at -78 °C for 30 min. Pivaldehyde (21 mmol) was added and the obtained mixture was stirred at room temperature for further 1 h. After removal of the solvent, I_2 (32 mmol), K_2CO_3 (60 mmol), and *t*-BuOH (30 mL) were added and the mixture was refluxed for 3 h. Then the reaction mixture was quenched with saturated Na_2SO_3 (25 mL) and extracted with $CHCl_3$ (3*30 mL). The organic layer was washed with brine and dried over anhydrous Na_2SO_4. The residue was further purified by flash column chromatography.

General procedure B:[2]

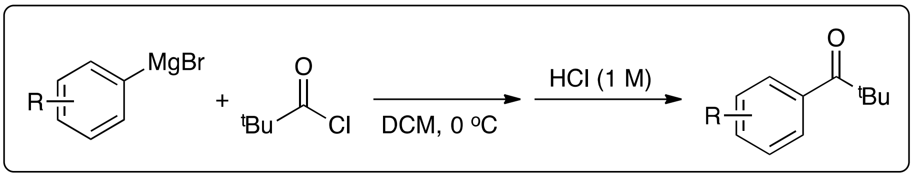

Supplementary Figure 3. Synthesis of ketones from acyl chloride

Under the atmosphere of N_2, aryl Grignard Reagent (15 mmol) was added slowly to a solution of pivaloyl chloride (10 mmol) in DCM (5 mL) at 0 °C. After stirring for 3 hours, the mixture was diluted with HCl (15 mL, 1 M) and extracted with ethyl acetate (3*20 mL). The combined organic layer was washed with saturated Na_2CO_3 (20 mL), brine (20 mL) and dried over anhydrous Na_2SO_4. The residue was further purified by flash column chromatography.

Substrates **1b**[1], **1c**[3a], **1d**[1], **1g**[4], **1h**[1], **1i**[5], **1j**[6], **1k**[7] and **1m**[8] were prepared following the *general procedure A*. **1a**[9], **1f**[10] and **1l**[3] were prepared following the *general procedure B*. All of these are known compounds and the NMR spectroscopy was consisted with those known data. Phenyl(*o*-tolyl)methanone (**1r**)[11], 1-(pyrimidin-2-yl)-1*H*-indole (**1s**)[12] and 2-phenylpyrimidine (**1t**)[13] were synthesized

according to the known literatures and the NMR spectroscopy was consisted with those data.

2,2-dimethyl-1-(4-(methylthio)phenyl)propan-1-one (1e)[3b]

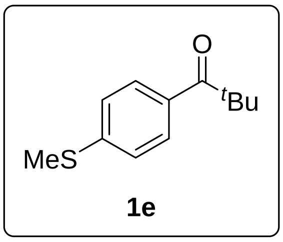

Isolated in 68% yield according to the general procedure A.

^{1}H NMR ($CDCl_3$, 500 MHz) δ 7.73 (d, J = 8.5 Hz, 2H), 7.23 (d, J = 8.5 Hz, 2H), 2.51 (s, 3H), 1.36 (s, 9H);

^{13}C NMR ($CDCl_3$, 125 MHz) δ 207.4, 143.4, 134.2, 129.1, 125.0, 44.2, 28.4, 15.1;

(S)-2-methyl-2-phenyl-3,4-dihydronaphthalen-1(2H)-one (1C)

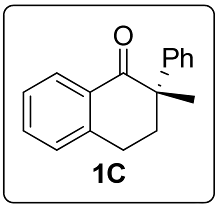

According to a known procedure with a slight modification (60 °C, 12h).[14]

^{1}H NMR ($CDCl_3$, 400 MHz) δ 8.15 (d, J = 8.0 Hz, 1H), 7.40 (td, J_1 = 7.2 Hz, J_2 = 1.2 Hz, 1H), 7.32-7.25 (m, 3H), 7.22-7.17 (m, 3H), 7.10 (d, J = 8.0 Hz, 1H), 2.84-2.80 (m, 2H), 2.61 (dt, J_1 = 16.0 Hz, J_2= 4.0 Hz, 1H), 2.30-2.22 (m, 1H), 1.53 (s, 3H);

^{13}C NMR ($CDCl_3$, 100 MHz) δ 201.5, 143.7, 142.3, 133.2, 132.9, 128.8, 128.7, 128.1, 126.8, 126.7, 126.5, 50.6, 36.4, 27.2, 26.3.

The *ee* value of the ketone was determined to be 96% by chiral HPLC analysis (Daicel CHIRALCEL AD-H; hexanes: *i*-PrOH = 98:2; detection wavelengths = 254 nm; flow rate = 1.0 mL/min). TR = 6.5 min (major) and 7.5 min (minor).

a

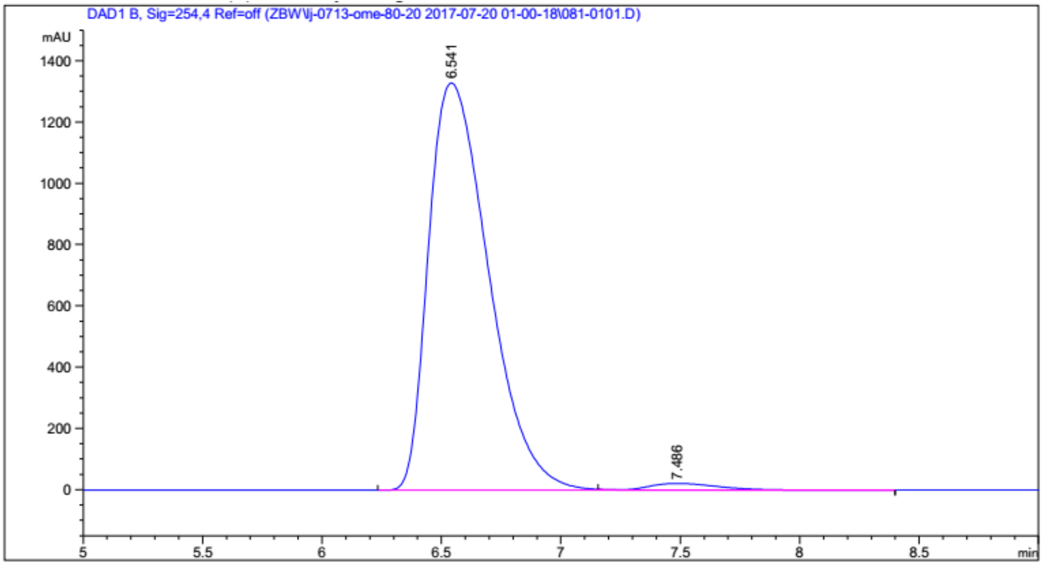

Peak #	RetTime [min]	Type	Width [min]	Area [mAU*s]	Height [mAU]	Area %
1	6.541	BV R	0.2762	2.32686e4	1329.64746	98.0782
2	7.486	VB E	0.3130	455.94125	22.44745	1.9218

b

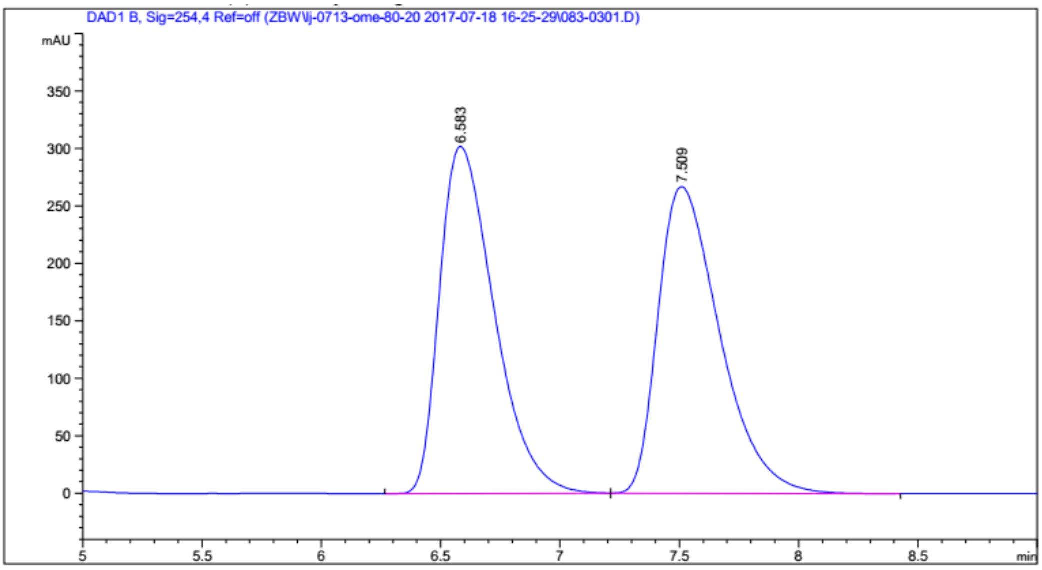

Peak #	RetTime [min]	Type	Width [min]	Area [mAU*s]	Height [mAU]	Area %
1	6.583	BB	0.2477	4805.53027	301.94675	50.0775
2	7.509	BB	0.2775	4790.65137	266.79788	49.9225

Supplementary Figure 4. Measurement of *ee* for ketone 1C.

(a) HPLC spectrum of 1C. (b) HPLC spectrum of a racemic sample

General procedure for the preparation of sulfonamides

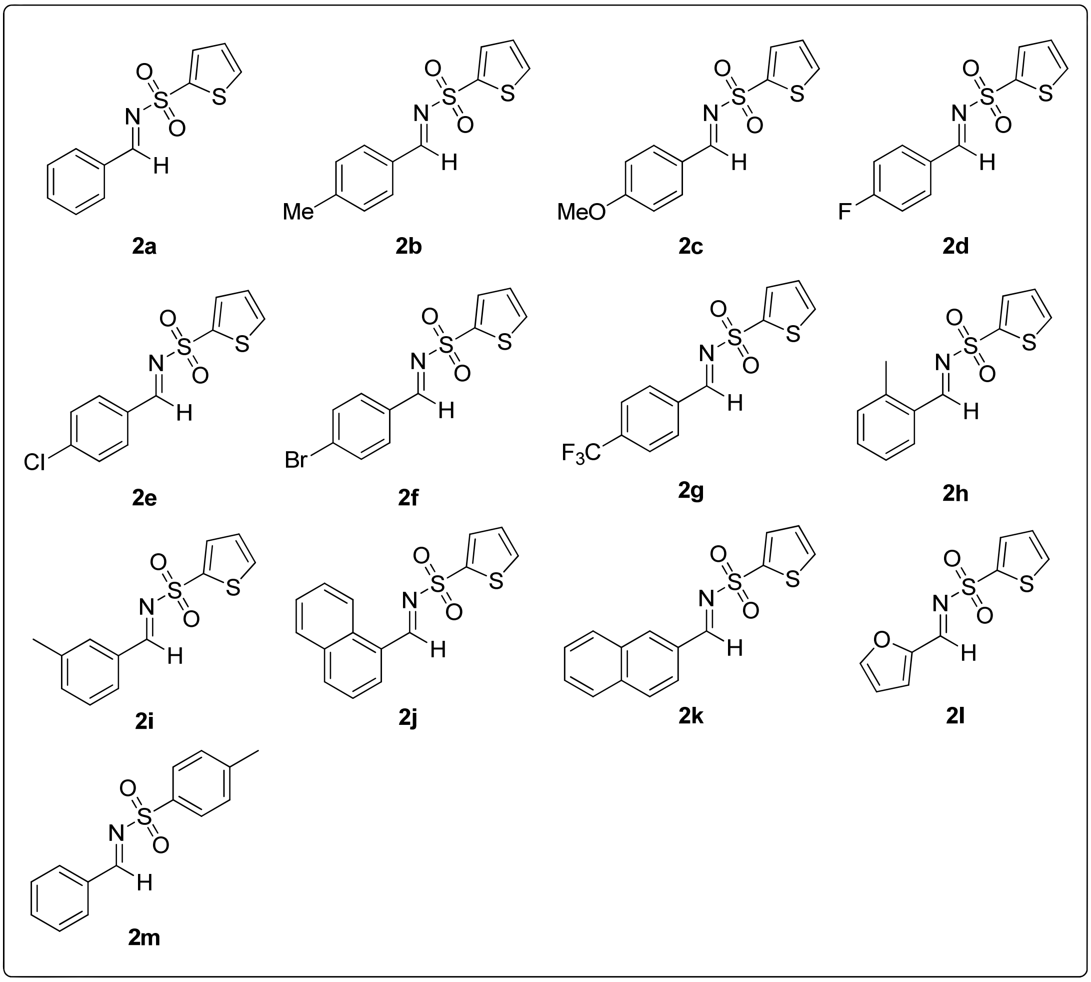

Supplementary Figure 5. Scope of imines

General procedure A:[15]

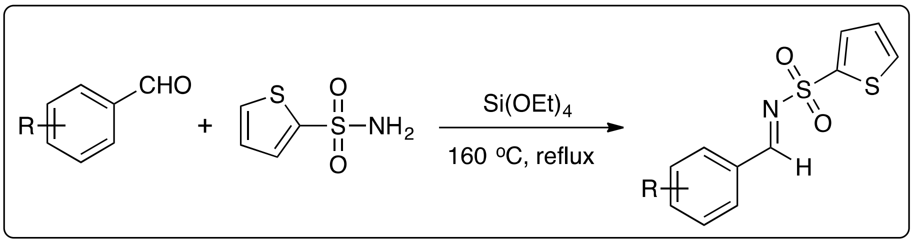

Supplementary Figure 6. Synthesis of imines from aldehydes

A 50 mL round bottom flask containing thiophene-2-sulfonamide (5 mmol), $Si(OEt)_4$ (5.5 mmol) and aldehyde (5.5 mmol) was fitted with a Dean-Stark trap under the atmosphere of nitrogen. The mixture was heated to 160 °C and stirred for further 3

hours. After cooling, the pure product was recrystallized from hexane and DCM.

General procedure B:[16]

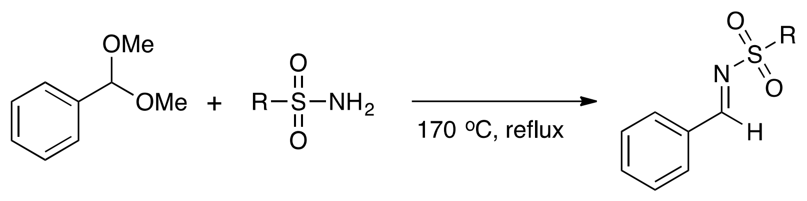

Supplementary Figure 7. Synthesis of imines from acetal

A 50 mL round bottom flask containing sulfonamide (20 mmol), benzaldehyde dimethylacetal (30 mmol) was fitted with a Dean-Stark trap under the atmosphere of nitrogen. The mixture was heated to 170 °C for 5 hours. After cooling, the volatile substrates were removed under vacuum resulting in a white solid. The pure product was recrystallized from hexane and DCM.

Sulfonamides **2b**[17], **2c**[18], **2d**[18], **2e**[19a], **2g**[19], **2h**[18], **2j**[18], **2k**[18], **2l**[18] were prepared according to *General procedure A* except **2a**[18] and **2m**[21] which were synthesized according to G*eneral procedure B.* The NMR spectroscopy was consisted with those known data.

(*E*)-*N*-(4-bromobenzylidene)thiophene-2-sulfonamide (2f)[20]

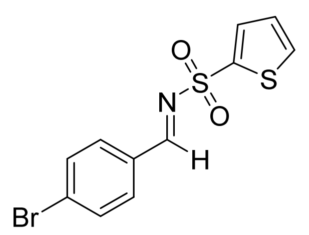

2f

Isolated in 78% yield according to *General procedure A*.

^{1}H NMR (CDCl$_3$, 500 MHz) δ 8.96 (s, 1H), 7.82-7.80 (m, 3H), 7.73 (dd, J_1 = 5.0 Hz, J_2 = 1.0 Hz, 1H), 7.66 (d, J = 8.0 Hz, 2H), 7.17-7.14 (m, 1H);

^{13}C NMR (CDCl$_3$, 125 MHz) δ 169.1, 138.4, 134.5, 134.4, 132.8, 132.7, 131.2, 130.7, 127.9;

(*E*)-*N*-(3-methylbenzylidene)thiophene-2-sulfonamide (2i)

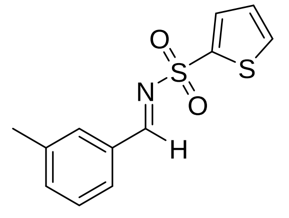

2i

Isolated in 67% yield according to *General procedure A.*

^{1}H NMR (CDCl$_3$, 500 MHz) δ 8.98 (s, 1H), 7.81-7.79 (m, 2H), 7.73 (d, J = 8.0 Hz, 1H), 7.70 (dd, J_1 = 5.0 Hz, J_2 = 1.0 Hz, 1H), 7.45 (d, J = 7.5 Hz, 1H), 7.40 (t, J = 7.5 Hz, 1H), 7.15 (t, J = 4.5 Hz, 1H), 2.41 (s, 3H);

^{13}C NMR ($CDCl_3$, 125 MHz) δ 170.7, 139.4, 138.8, 136.3, 134.2, 134.1, 132.3, 131.6, 129.3, 129.2, 127.9, 21.3;

HRMS(ESI) Calculated for $C_{12}H_{12}O_2NS_2^+$ ($[M+H]^+$): 266.03040, found: 266.03026.

Optimization of reaction conditions

Supplementary Table 1. Survey of the reaction parameters[a]

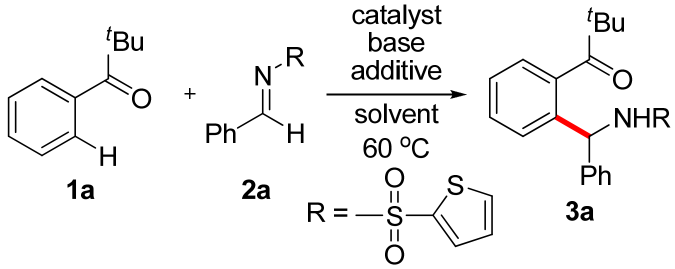

Entry	Catalyst/mol%	Base/equiv	Additive/equiv	Solvent	**1a**:**2a**	Yield/%[b]
1	$MnBr(CO)_5$/10	Me_2Zn/1.0	$ZnBr_2$/0.5	Et_2O	1:1	34
2	$MnBr(CO)_5$/10	Me_2Zn/1.0	$ZnBr_2$/0.5	THF	1:1	28
3	$MnBr(CO)_5$/10	Me_2Zn/1.0	$ZnBr_2$/0.5	Dioxane	1:1	24
4	$MnBr(CO)_5$/10	Me_2Zn/1.0	$ZnBr_2$/0.5	Toluene	1:1	0
5	$MnBr(CO)_5$/10	Me_2Zn/1.0	$ZnBr_2$/0.5	DCM	1:1	50
6	$MnBr(CO)_5$/10	Me_2Zn/1.0	$ZnBr_2$/0.5	DCE	1:1	52
7	$MnBr(CO)_5$/10	-	$ZnBr_2$/0.5	DCE	1:1	0
8	$MnBr(CO)_5$/10	Me_2Zn/0.5	$ZnBr_2$/0.5	DCE	1:1	13
9	$MnBr(CO)_5$/10	Me_2Zn/1.5	$ZnBr_2$/0.5	DCE	1:1	56
10	$MnBr(CO)_5$/10	Me_2Zn/2.0	$ZnBr_2$/0.5	DCE	1:1	48
11	$MnBr(CO)_5$/10	MeMgBr/1.5	$ZnBr_2$/0.5	DCE	1:1	0
12	$MnBr(CO)_5$/10	PhMgBr/1.5	$ZnBr_2$/0.5	DCE	1:1	0
13	$MnBr(CO)_5$/10	*n*-BuLi/1.5	$ZnBr_2$/0.5	DCE	1:1	0
14	$MnBr(CO)_5$/10	Me_2Zn/1.5	$MgBr_2$/0.5	DCE	1:1	12
15	$MnBr(CO)_5$/10	Me_2Zn/1.5	$ZnCl_2$/0.5	DCE	1:1	41
16	$MnBr(CO)_5$/10	Me_2Zn/1.5	$FeBr_2$/0.5	DCE	1:1	52
17	$MnBr(CO)_5$/10	Me_2Zn/1.5	$CuBr_2$/0.5	DCE	1:1	51
18	$MnBr(CO)_5$/10	Me_2Zn/1.5	AgOAc/0.5	DCE	1:1	0
19	MnBr(CO)5/10	Me_2Zn/1.5	$ZnBr_2$/1.0	DCE	1:1	59
20	MnBr(CO)5/10	Me_2Zn/1.5	-	DCE	1:1	16
21	$Mn_2(CO)_{10}$/10	Me_2Zn/1.5	$ZnBr_2$/1.0	DCE	1:1	17
22	$ReBr(CO)_5$10	Me_2Zn/1.5	$ZnBr_2$/1.0	DCE	1:1	0
23	$Re_2(CO)_{10}$/10	Me_2Zn/1.5	$ZnBr_2$/1.0	DCE	1:1	0
24	$Fe_2(CO)_9$/10	Me_2Zn/1.5	$ZnBr_2$/1.0	DCE	1:1	0
25	$Ru(CO)_6$/10	Me_2Zn/1.5	$ZnBr_2$/1.0	DCE	1:1	0
26	$Mo(CO)_6$/10	Me_2Zn/1.5	$ZnBr_2$/1.0	DCE	1:1	0
27	$MnBr(CO)_5$/5	Me_2Zn/1.5	$ZnBr_2$/1.0	DCE	1:1	53
28	$MnBr(CO)_5$/10	Me_2Zn/1.5	$ZnBr_2$/1.0	DCE	1:2	53
29	$MnBr(CO)_5$/10	Me_2Zn/1.5	$ZnBr_2$/1.0	DCE	2:1	71
30	$MnBr(CO)_5$/10	Me_2Zn/1.5	$ZnBr_2$/1.0	DCE	3:1	81(80)[c]
31	$MnBr(CO)_5$/10	Me_2Zn/1.5	$ZnBr_2$/0.5	DCE	3:1	53

[a] Reaction conditions unless otherwise noted: **1a** (0.2 mmol), **2a** (0.2 mmol), catalyst (0.02

mmol), base (0.2 mmol), additive (0.1 mmol), solvent (0.5 mL), 60 °C, 10 h under N_2 atmosphere. [b] Yields determined by ^{1}H NMR analysis with 1,3,5-trimethoxybenzene as an internal standard. [c] Isolated yield on 0.5 mmol scale was shown in the parentheses. MeMgBr: 1M in THF. PhMgBr: 1M in THF. Me_2Zn: 1.2 M in toluene.

Experimental details and characterization of products

To a 25 ml flame-dried Schlenk tube was added $ZnBr_2$ (0.5 mmol, 112.5 mg, stored in glove box), $MnBr(CO)_5$ (0.05 mmol, 10.0 mol%, 13.8 mg), DCE (1.25 mL), 2,2-dimethyl-1-phenylpropan-1-one **1a** (1.5 mmol, 243.0 mg), (*E*)-*N*-benzylidenethiophene-2-sulfonamide **2a** (0.5 mmol, 125.5 mg) and Me_2Zn (0.75 mmol, 1.2 M in toluene, 0.625 mL) sequentially under nitrogen. The tube was sealed and stirred at 60 °C for 10 h. After completion, the reaction mixture was diluted with ethyl acetate (5.0 mL) and filtered through a short pad silica gel washing with ethyl acetate (20 mL). The filtrate was concentrated and purified by silica gel column chromatography to provide the product **3a** in 80% yield.

***N*-(phenyl(2-pivaloylphenyl)methyl)thiophene-2-sulfonamide (3a)**

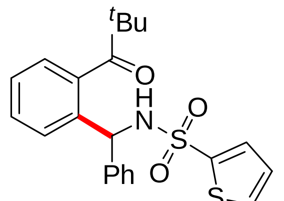

3a

^{1}H NMR ($CDCl_3$, 400 MHz) δ 7.44-7.39 (m, 3H), 7.32-7.28 (m, 1H), 7.27-7.23 (m, 2H), 7.21-7.14 (m, 3H), 7.09 (d, *J* = 7.2 Hz, 2H), 6.89-6.84 (m, 2H), 5.76 (d, *J* = 8.4 Hz, 1H), 0.88 (s, 9H);

^{13}C NMR ($CDCl_3$, 100 MHz) δ 214.9, 142.3, 139.9, 139.5, 138.3, 132.0, 131.5, 131.0, 130.0, 128.3, 127.4, 127.0 (overlap), 126.9, 60.7, 44.2, 28.1;

HRMS(ESI) Calculated for $C_{22}H_{23}O_3NNaS_2^+$ ($[M+Na]^+$): 436.10029, found: 436.10063.

***N*-((5-methyl-2-pivaloylphenyl)(phenyl)methyl)thiophene-2-sulfonamide (3b)**

Following a general procedure: To a 25 ml flame-dried Schlenk tube was added $ZnBr_2$ (0.5 mmol, 112.5 mg, stored in glove box), $MnBr(CO)_5$ (0.05 mmol, 10.0 mol%, 13.8 mg), DCE (1.25 mL), 2,2-dimethyl-1-*p* -tolylpropan-1-one **1b** (1.5 mmol, 264.0 mg),

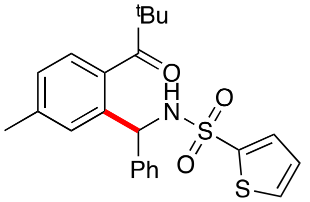

3b

(*E*)-*N*-benzylidenethiophene-2-sulfonamide **2a** (0.5 mmol, 125.5 mg) and Me_2Zn (0.75 mmol, 1.2 M in toluene, 0.625 mL) sequentially under nitrogen. The tube was sealed and stirred at 60 ^oC for 10 h. After completion, the reaction mixture was diluted with ethyl acetate (5.0 mL) and filtered through a short pad silica gel washing with ethyl acetate (20 mL). The filtrate was concentrated and purified by silica gel column chromatography to provide the product **3b** in 63% yield.

^{1}H NMR ($CDCl_3$, 400 MHz) δ 7.41-7.38 (m, 2H), 7.34 (d, *J* = 8.0 Hz, 1H), 7.22-7.09 (m, 5H), 7.06-7.02 (m, 3H), 6.89-6.86 (m, 1H), 5.69 (d, *J* = 9.2 Hz, 1H), 2.25 (s, 3H), 0.85 (s, 9H);

^{13}C NMR ($CDCl_3$, 100 MHz) δ 214.9, 142.6, 140.3, 140.0, 139.7, 135.4, 132.2, 131.9, 131.3, 128.2, 127.4, 127.3, 127.2, 126.9 (overlap), 61.0, 44.1, 28.1, 21.2;

HRMS(ESI) Calculated for $C_{23}H_{25}O_3NNaS_2^+$ ($[M+Na]^+$): 450.11681, found: 450.11612.

***N*-(phenyl(4-pivaloylbiphenyl-3-yl)methyl)thiophene-2-sulfonamide (3c)**

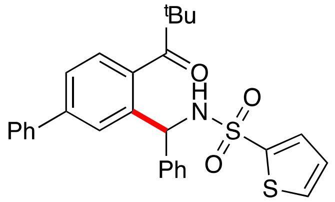

3c

Following a general procedure: To a 25 ml flame-dried Schlenk tube was added $ZnBr_2$ (0.5 mmol, 112.5 mg, stored in glove box), $MnBr(CO)_5$ (0.05 mmol, 10.0 mol%, 13.8 mg), DCE (1.25 mL), 1-(biphenyl-4-yl)-2,2 -dimethylpropan-1-one **1c** (1.5 mmol, 357.0 mg), (*E*)-*N*-benzylidenethiophene-2-sulfonamide **2a** (0.5 mmol, 125.5 mg) and Me_2Zn (0.75 mmol, 1.2 M in toluene, 0.625 mL) sequentially under nitrogen. The tube was sealed and stirred at 60 ^oC for 10 h. After completion, the reaction mixture was diluted with ethyl acetate (5.0 mL) and filtered through a short pad silica gel washing with ethyl acetate (20 mL). The filtrate was concentrated and purified by silica gel column chromatography to provide the product **3c** in 74% yield.

^{1}H NMR ($CDCl_3$, 400 MHz) δ 7.55-7.39 (m, 9H), 7.28-7.13 (m, 6H), 6.98 (d, *J* = 9.6

Hz, 1H), 6.77 (t, J = 4.0 Hz, 1H), 5.84 (d, J = 9.2 Hz, 1H), 0.89 (s, 9H);

^{13}C NMR ($CDCl_3$, 100 MHz) δ 214.7, 142.8, 142.7, 140.5, 139.9, 139.3, 137.0, 131.9, 131.4, 130.2, 129.0, 128.4, 128.3, 128.1, 127.5, 127.2, 127.1, 127.0, 125.3, 61.3, 44.4, 28.2;

HRMS(ESI) Calculated for $C_{28}H_{27}O_3NNaS_2^+$ ($[M+Na]^+$): 512.13246, found: 512.13199.

***N*-((5-methoxy-2-pivaloylphenyl)(phenyl)methyl)thiophene-2-sulfonamide (3d)**

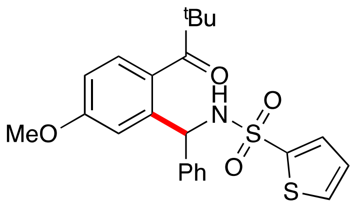

3d

Following a general procedure: To a 25 ml flame-dried Schlenk tube was added $ZnBr_2$ (0.5 mmol, 112.5 mg, stored in glove box), $MnBr(CO)_5$ (0.05 mmol, 10.0 mol%, 13.8 mg), DCE (1.25 mL), 1-(4-methoxyphenyl)-2,2-dimethylpropan-1-one **1d** (1.5 mmol, 288.0 mg), (*E*)-*N*-benzylidenethiophene-2-sulfonamide **2a** (0.5 mmol, 125.5 mg) and Me_2Zn (0.75 mmol, 1.2 M in toluene, 0.625 mL) sequentially under nitrogen. The tube was sealed and stirred at 60 ^{o}C for 10 h. After completion, the reaction mixture was diluted with ethyl acetate (5.0 mL) and filtered through a short pad silica gel washing with ethyl acetate (20 mL). The filtrate was concentrated and purified by silica gel column chromatography to provide the product **3d** in 73% yield.

^{1}H NMR ($CDCl_3$, 400 MHz) δ 7.45-7.39 (m, 3H), 7.22-7.13 (m, 4H), 7.10 (d, J = 7.2 Hz, 2H), 6.90-6.87 (m, 1H), 6.82 (d, J = 2.8 Hz, 1H), 6.74 (dd, J_1 = 8.4 Hz, J_2 = 2.8 Hz, 1H), 5.73 (d, J = 9.2 Hz, 1H), 3.76 (s, 3H), 0.85 (s, 9H);

^{13}C NMR ($CDCl_3$, 100 MHz) δ 213.8, 160.4, 142.6, 142.2, 139.8, 131.8, 131.4, 130.5, 129.4, 128.1, 127.3, 127.0, 126.9, 117.2, 111.5, 61.0, 55.4, 44.1, 28.2;

HRMS(ESI) Calculated for $C_{23}H_{25}O_4NNaS_2^+$ ($[M+Na]^+$): 466.11172, found: 466.11113.

***N*-((5-(methylthio)-2-pivaloylphenyl)(phenyl)methyl)thiophene-2-sulfonamide (3e)**

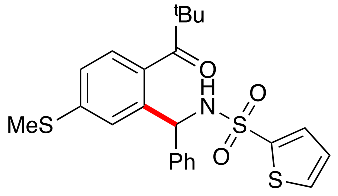

3e

Following a general procedure: To a 25 ml flame-dried Schlenk tube was added $ZnBr_2$ (0.5 mmol, 112.5 mg, stored in glove box), $MnBr(CO)_5$ (0.05 mmol, 10.0 mol%, 13.8 mg), DCE (1.25 mL), 2,2-dimethyl-1-(4-(methylthio)phenyl)propan-1-one **1e** (1.5 mmol, 312.0 mg), (*E*)-*N*-benzylidene -thiophene-2-sulfonamide **2a** (0.5 mmol, 125.5 mg) and Me_2Zn (0.75 mmol, 1.2 M in toluene, 0.625 mL) sequentially under nitrogen. The tube was sealed and stirred at 60 °C for 10 h. After completion, the reaction mixture was diluted with ethyl acetate (5.0 mL) and filtered through a short pad silica gel washing with ethyl acetate (20 mL). The filtrate was concentrated and purified by silica gel column chromatography to provide the product **3e** in 61% yield.

^{1}H NMR ($CDCl_3$, 400 MHz) δ 7.44-7.42 (m, 2H), 7.37 (d, *J* = 8.8 Hz, 1H), 7.24-7.17 (m, 3H), 7.12-7.05 (m, 4H), 7.02 (d, *J* = 9.2 Hz, 1H), 6.93-6.90 (m, 1H), 5.71 (d, *J* = 9.2 Hz, 1H), 2.46 (s, 3H), 0.84 (s, 9H);

^{13}C NMR ($CDCl_3$, 100 MHz) δ 214.2, 142.5, 142.2, 140.6, 139.7, 134.3, 132.0, 131.5, 128.3, 128.0, 127.5, 127.1, 127.0, 123.4, 61.2, 44.2, 28.2, 14.9;

HRMS(ESI) Calculated for $C_{23}H_{25}O_3NNaS_3^+$ ($[M+Na]^+$): 482.08888, found: 482.08815.

N-((5-(dimethylamino)-2-pivaloylphenyl)(phenyl)methyl)thiophene-2-sulfonamide (3f)

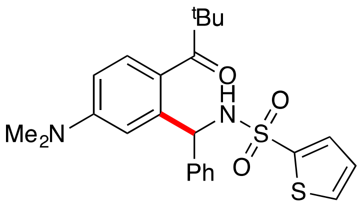

3f

Following a general procedure: To a 25 ml flame-dried Schlenk tube was added $ZnBr_2$ (0.5 mmol, 112.5 mg, stored in glove box), $MnBr(CO)_5$ (0.05 mmol, 10.0 mol%, 13.8 mg), DCE (1.25 mL), 1-(4-(dimethylamino)phenyl)-2,2-dimethylpropan-1-one **1f** (1.5 mmol, 312.0 mg), (*E*)-*N*-benzylidenethiophene-2-sulfonamide **2a** (0.5 mmol, 125.5 mg) and Me_2Zn (0.75 mmol, 1.2 M in toluene, 0.625 mL) sequentially under nitrogen. The tube was sealed and stirred at 60 °C for 10 h. After completion,

the reaction mixture was diluted with ethyl acetate (5.0 mL) and filtered through a short pad silica gel washing with ethyl acetate (20 mL). The filtrate was concentrated and purified by silica gel column chromatography to provide the product **3f** in 42% yield.

^{1}H NMR ($CDCl_3$, 400 MHz) δ 7.66 (d, J = 1.0 Hz, 1H), 7.44-7.36 (m, 3H), 7.21-7.13 (m, 5H), 6.86 (t, J = 4.0 Hz, 1H), 6.51 (d, J = 2.4 Hz, 1H), 6.45 (dd, J_1 = 8.8 Hz, J_2 = 2.4 Hz, 1H), 5.67 (d, J = 1.0 Hz, 1H), 2.96 (s, 6H), 0.83 (s, 9H);

^{13}C NMR ($CDCl_3$, 100 MHz) δ 213.2, 151.0, 143.3, 142.4, 140.4, 131.5, 130.9, 130.2, 127.9, 127.0, 126.9, 126.7, 124.9, 115.1, 108.6, 62.4, 43.9, 40.0, 28.5;

HRMS(ESI) Calculated for $C_{24}H_{28}O_3N_2FNaS_2^+$ ($[M+Na]^+$): 479.14336, found: 479.14323.

***N*-((5-fluoro-2-pivaloylphenyl)(phenyl)methyl)thiophene-2-sulfonamide (3g)**

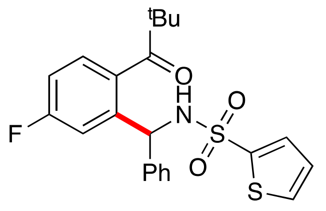

3g

Following a general procedure: To a 25 ml flame-dried Schlenk tube was added $ZnBr_2$ (0.5 mmol, 112.5 mg, stored in glove box), $MnBr(CO)_5$ (0.05 mmol, 10.0 mol%, 13.8 mg), DCE (1.25 mL), 1-(4-fluorophenyl)-2,2-dimethylpropan-1-one **1g** (1.5 mmol, 270.0 mg), (*E*)-*N*-benzylidenethiophene-2-sulfonamide **2a** (0.5 mmol, 125.5 mg) and Me_2Zn (0.75 mmol, 1.2 M in toluene, 0.625 mL) sequentially under nitrogen. The tube was sealed and stirred at 60 °C for 10 h. After completion, the reaction mixture was diluted with ethyl acetate (5.0 mL) and filtered through a short pad silica gel washing with ethyl acetate (20 mL). The filtrate was concentrated and purified by silica gel column chromatography to provide the product **3g** in 60% yield.

^{1}H NMR ($CDCl_3$, 400 MHz) δ 7.50-7.43 (m, 3H), 7.24-7.18 (m, 3H), 7.10 (dd, J_1 = 9.2 Hz, J_2 = 2.4 Hz, 1H), 7.06-7.03 (m, 2H), 6.98-6.92 (m, 2H), 6.61 (d, J = 8.8 Hz, 1H), 5.76 (d, J = 8.8 Hz, 1H), 0.88 (s, 9H);

^{13}C NMR ($CDCl_3$, 100 MHz) δ 213.5, 162.9 (d, $^1J_{C-F}$= 250.7 Hz), 143.1 (d, $^3J_{C-F}$=

6.9 Hz), 142.2, 139.4, 134.5 (d, $^{4}J_{C-F}$ = 3.6 Hz), 132.3, 131.8, 129.3 (d, $^{3}J_{C-F}$ = 8.4 Hz), 128.6, 127.8, 127.2, 127.2, 118.3 (d, $^{2}J_{C-F}$ = 22.3 Hz), 113.7 (d, $^{2}J_{C-F}$ = 21.1 Hz), 60.3, 44.4, 28.2;

^{19}F NMR ($CDCl_3$, 565 MHz) δ -109.2;

HRMS(ESI) Calculated for $C_{22}H_{22}O_3NFNaS_2^+$ ($[M+Na]^+$): 454.09173, found: 454.09133.

N-((5-chloro-2-pivaloylphenyl)(phenyl)methyl)thiophene-2-sulfonamide (3h)

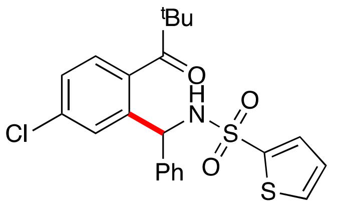

3h

Following a general procedure: To a 25 ml flame-dried Schlenk tube was added $ZnBr_2$ (0.5 mmol, 112.5 mg, stored in glove box), $MnBr(CO)_5$ (0.05 mmol, 10.0 mol%, 13.8 mg), DCE (1.25 mL), 1-(4-chlorophenyl)-2,2-dimethylpropan-1-one **1h** (1.5 mmol, 294.0 mg), (*E*)-*N*-benzylidenethiophene-2-sulfonamide **2a** (0.5 mmol, 125.5 mg) and Me_2Zn (0.75 mmol, 1.2 M in toluene, 0.625 mL) sequentially under nitrogen. The tube was sealed and stirred at 60 °C for 10 h. After completion, the reaction mixture was diluted with ethyl acetate (5.0 mL) and filtered through a short pad silica gel washing with ethyl acetate (20 mL). The filtrate was concentrated and purified by silica gel column chromatography to provide the product **3h** in 64% yield.

^{1}H NMR ($CDCl_3$, 400 MHz) δ 7.48-7.45 (m, 2H), 7.37 (d, *J* = 8.4 Hz, 1H), 7.29 (d, *J* = 2.0 Hz, 1H), 7.25-7.18 (m, 4H), 7.08-7.05 (m, 2H), 6.97-6.93 (m, 1H), 6.67 (d, *J* = 9.2 Hz, 1H), 5.71 (d, *J* = 9.2 Hz, 1H), 0.86 (s, 9H);

^{13}C NMR ($CDCl_3$, 100 MHz) δ 213.8, 142.1, 141.7, 139.2, 136.6, 136.0, 132.3, 131.9, 131.3, 128.6, 128.5, 127.8, 127.2, 127.1, 127.0, 60.4, 44.4, 28.1;

HRMS(ESI) Calculated for $C_{22}H_{22}O_3NClNaS_2^+$ ($[M+Na]^+$): 470.06218, found: 470.06175.

N-((5-bromo-2-pivaloylphenyl)(phenyl)methyl)thiophene-2-sulfonamide (3i)

Following a general procedure: To a 25 ml flame-dried Schlenk tube was added $ZnBr_2$

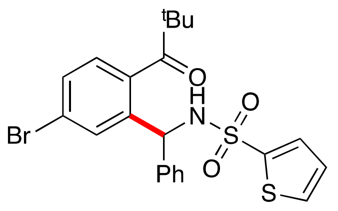

3i

(0.5 mmol, 112.5 mg, stored in glove box), $MnBr(CO)_5$ (0.05 mmol, 10.0 mol%, 13.8 mg), DCE (1.25 mL), 1-(4-bromophenyl)- 2,2-dimethylpropan-1-one **1i** (1.5 mmol, 360.0 mg), (*E*)-*N*-benzylidenethiophene-2-sulfonamide **2a** (0.5 mmol, 125.5 mg) and Me_2Zn (0.75 mmol, 1.2 M in toluene, 0.625 mL) sequentially under nitrogen. The tube was sealed and stirred at 60 °C for 10 h. After completion, the reaction mixture was diluted with ethyl acetate (5.0 mL) and filtered through a short pad silica gel washing with ethyl acetate (20 mL). The filtrate was concentrated and purified by silica gel column chromatography to provide the product **3i** in 55% yield.

^{1}H NMR ($CDCl_3$, 400 MHz) δ 7.47 (d, J = 4.0 Hz, 2H), 7.43 (d, J = 2.0 Hz, 1H), 7.39 (dd, J_1 = 8.0 Hz, J_2 = 2.0 Hz, 1H), 7.29 (d, J = 8.0 Hz, 1H), 7.25-7.19 (m, 3H), 7.07 (d, J = 6.8 Hz, 2H), 6.95 (t, J = 4.0 Hz, 1H), 6.67 (d, J = 9.2 Hz, 1H), 5.70 (d, J = 9.2 Hz, 1H), 0.85 (s, 9H);

^{13}C NMR ($CDCl_3$, 100 MHz) δ 214.0, 142.1, 141.7, 139.2, 137.1, 134.1, 132.3, 131.9, 130.0, 128.6, 128.6, 127.8, 127.3, 127.1, 124.4, 60.4, 44.4, 28.1;

HRMS(ESI) Calculated for $C_{22}H_{22}O_3NBrNaS_2^+$ ($[M+Na]^+$): 514.01167, found: 514.01112.

***N*-((5-iodo-2-pivaloylphenyl)(phenyl)methyl)thiophene-2-sulfonamide (3j)**

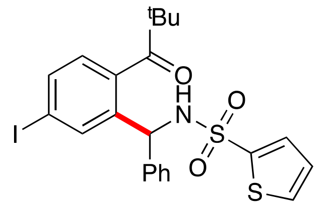

3j

Following a general procedure: To a 25 ml flame-dried Schlenk tube was added $ZnBr_2$ (0.5 mmol, 112.5 mg, stored in glove box), $MnBr(CO)_5$ (0.05 mmol, 10.0 mol%, 13.8 mg), DCE (1.25 mL), 1-(4-iodophenyl)-2,2-dimethylpropan-1-one **1j** (1.5 mmol, 432.0 mg), (*E*)-*N*-benzylidenethiophene-2-sulfonamide **2a** (0.5 mmol, 125.5 mg) and Me_2Zn (0.75 mmol, 1.2 M in toluene, 0.625 mL) sequentially under nitrogen. The tube was sealed and stirred at 60 °C for 10 h. After completion, the reaction mixture was diluted with ethyl acetate (5.0 mL) and filtered through a short pad silica gel washing with

ethyl acetate (20 mL). The filtrate was concentrated and purified by silica gel column chromatography to provide the product **3j** in 60% yield.

^{1}H NMR ($CDCl_3$, 400 MHz) δ 7.60-7.57 (m, 2H), 7.49-7.44 (m, 2H), 7.26-7.19 (m, 3H), 7.14 (d, *J* = 8.0 Hz, 1H), 7.08 (d, *J* = 7.2 Hz, 2H), 6.98-6.95 (m, 1H), 6.73 (d, *J* = 9.2 Hz, 1H), 5.66 (d, *J* = 9.2 Hz, 1H), 0.84 (s, 9H);

^{13}C NMR ($CDCl_3$, 100 MHz) δ 214.2, 142.1, 141.4, 140.0, 139.2, 137.6, 136.0, 132.2, 132.0, 128.5 (overlap), 127.8, 127.3, 127.1, 96.8, 60.4, 44.3, 28.1;

HRMS(ESI) Calculated for $C_{22}H_{22}O_3NINaS_2^+$ ([M+Na]$^+$): 561.99780, found: 561.99747.

***N*-((4-methyl-2-pivaloylphenyl)(phenyl)methyl)thiophene-2-sulfonamide (3k)**

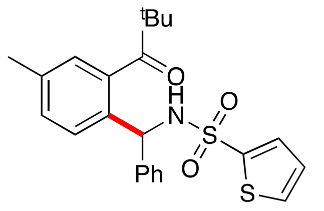

3k

Following a general procedure: To a 25 ml flame-dried Schlenk tube was added $ZnBr_2$ (0.5 mmol, 112.5 mg, stored in glove box), $MnBr(CO)_5$ (0.05 mmol, 10.0 mol%, 13.8 mg), DCE (1.25 mL), 2,2-dimethyl-1-*m*-tolylpropan-1-one **1k** (1.5 mmol, 264.0 mg), (*E*)-*N*-benzylidenethiophene-2-sulfonamide **2a** (0.5 mmol, 125.5 mg) and Me_2Zn (0.75 mmol, 1.2 M in toluene, 0.625 mL) sequentially under nitrogen. The tube was sealed and stirred at 60 ^{o}C for 10 h. After completion, the reaction mixture was diluted with ethyl acetate (5.0 mL) and filtered through a short pad silica gel washing with ethyl acetate (20 mL). The filtrate was concentrated and purified by silica gel column chromatography to provide the product **3k** in 63% yield.

^{1}H NMR ($CDCl_3$, 400 MHz) δ 7.42-7.40 (m, 2H), 7.21-7.12 (m, 5H), 7.10-7.04 (m, 3H), 6.89 (d, *J* = 4.0 Hz, 1H), 6.79 (d, *J* = 8.8 Hz, 1H), 5.70 (d, *J* = 9.2 Hz, 1H), 2.32 (s, 3H), 0.89 (s, 9H);

^{13}C NMR ($CDCl_3$, 100 MHz) δ 215.0, 142.5, 140.2, 138.4, 136.8, 136.7, 132.0, 131.4, 131.1, 130.6, 128.3, 127.6, 127.3, 127.1, 127.0, 60.6, 44.2, 28.2, 21.2;

HRMS(ESI) Calculated for $C_{23}H_{25}O_3NNaS_2^+$ ([M+Na]$^+$): 450.11681, found:

450.11648.

***N*-(phenyl(3-pivaloylnaphthalen-2-yl)methyl)thiophene-2-sulfonamide (3l)**

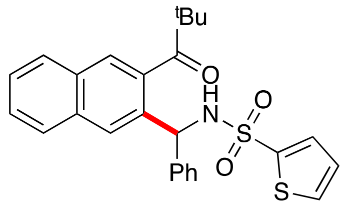

3l

Following a general procedure: To a 25 ml flame-dried Schlenk tube was added $ZnBr_2$ (0.5 mmol, 112.5 mg, stored in glove box), $MnBr(CO)_5$ (0.05 mmol, 10.0 mol%, 13.8 mg), DCE (1.25 mL), 2,2-dimethyl-1-(naphthalen-2-yl)propan-1-one **1l** (1.5 mmol, 318.0 mg), (*E*)-*N*-benzylidenethiophene-2-sulfonamide **2a** (0.5 mmol, 125.5 mg) and Me_2Zn (0.75 mmol, 1.2 M in toluene, 0.625 mL) sequentially under nitrogen. The tube was sealed and stirred at 60 °C for 10 h. After completion, the reaction mixture was diluted with ethyl acetate (5.0 mL) and filtered through a short pad silica gel washing with ethyl acetate (20 mL). The filtrate was concentrated and purified by silica gel column chromatography to provide the product **3l** in 59% yield.

^{1}H NMR ($CDCl_3$, 400 MHz) δ 7.92 (s, 1H), 7.82-7.79 (m, 1H), 7.74-7.71 (m, 2H), 7.58-7.51 (m, 2H), 7.42 (dd, J_1 = 3.6 Hz, J_2 = 1.2 Hz, 1H), 7.25-7.21 (m, 4H), 7.14-7.11 (m, 2H), 7.05 (d, J = 9.2 Hz, 1H), 6.75-6.72 (m, 1H), 5.96 (d, J = 9.6 Hz, 1H), 0.91 (s, 9H);

^{13}C NMR ($CDCl_3$, 100 MHz) δ 214.7, 142.5, 140.0, 136.0, 135.5, 133.2, 132.0, 131.4, 131.0, 130.8, 128.4, 128.3, 128.2, 128.0, 127.9, 127.5, 127.4, 127.2, 126.9, 61.4, 44.5, 28.5;

HRMS(ESI) Calculated for $C_{26}H_{25}O_3NNaS_2^+$ ([M+Na]$^+$): 486.11681, found: 486.11652.

***N*-(phenyl(4-pivaloylthiophen-3-yl)methyl)thiophene-2-sulfonamide (3m)**

Following a general procedure: To a 25 ml flame-dried Schlenk tube was added $ZnBr_2$ (0.5 mmol, 112.5 mg, stored in glove box), $MnBr(CO)_5$ (0.05 mmol, 10.0 mol%, 13.8 mg), DCE (1.25 mL), 2,2-dimethyl-1-(thiophen-2-yl) propan-1-one **1m** (1.5 mmol, 252.0 mg), (*E*)-*N*-benzylidene thiophene-2-sulfonamide **2a** (0.5 mmol, 125.5 mg) and

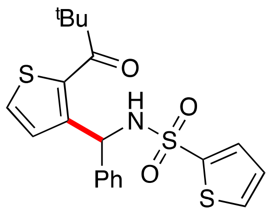
3m

Me_2Zn (0.75 mmol, 1.2 M in toluene, 0.625 mL) sequentially under nitrogen. The tube was sealed and stirred at 60 °C for 10 h. After completion, the reaction mixture was diluted with ethyl acetate (5.0 mL) and filtered through a short pad silica gel washing with ethyl acetate (20 mL). The filtrate was concentrated and purified by silica gel column chromatography to provide the product **3m** in 42% yield.

^{1}H NMR ($CDCl_3$, 400 MHz) δ 7.45-7.41 (m, 2H), 7.33 (d, *J* = 4.8 Hz, 1H), 7.20-7.15 (m, 3H), 7.12 (d, *J* = 7.2 Hz, 2H), 7.05 (d, *J* = 4.8 Hz, 1H), 6.92 (t, *J* = 4.0 Hz, 1H), 6.81 (d, *J* = 9.2 Hz, 1H), 6.08 (d, *J* = 9.2 Hz, 1H), 1.1 (s, 9H);

^{13}C NMR ($CDCl_3$, 100 MHz) δ 202.3, 148.6, 142.2, 139.6, 133.4, 132.0, 131.7, 130.1, 128.2, 128.2, 127.4, 127.2, 126.7, 56.3, 44.7, 27.5;

HRMS(ESI) Calculated for $C_{20}H_{21}O_3NNaS_3^+$ ([M+Na]$^+$): 442.05758, found: 442.05713.

***N*-((2-(cyclopropanecarbonyl)phenyl)(phenyl)methyl)thiophene-2-sulfonamide (3n)**

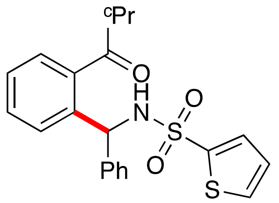
3n

Following a general procedure: To a 25 ml flame-dried Schlenk tube was added $ZnBr_2$ (0.5 mmol, 112.5 mg, stored in glove box), $MnBr(CO)_5$ (0.05 mmol, 10.0 mol%, 13.8 mg), DCM (5.0 mL), cyclopropyl(phenyl)methanone **1n** (2.0 mmol, 292.0 mg), (*E*)-*N*-benzylidenethiophene-2-sulfonamide **2a** (0.5 mmol, 125.5 mg) and Me_2Zn (0.75 mmol, 1.2 M in toluene, 0.625 mL) sequentially under nitrogen. The tube was sealed and stirred at r.t. for 16 h. After completion, the reaction mixture was diluted with ethyl acetate (5.0 mL) and filtered through a short pad silica gel washing with ethyl acetate (20 mL). The filtrate was concentrated and purified by silica gel column chromatography to provide the product **3n** in 71% yield.

^{1}H NMR ($CDCl_3$, 400 MHz) δ 7.70-7.67 (m, 2H), 7.40-7.28 (m, 5H), 7.19-7.13 (m,

4H), 7.08-7.05 (m, 2H), 6.86 (dd, J = 4.8 Hz, 1H), 5.86 (d, J = 9.6 Hz, 1H), 2.12-2.04 (m, 1H), 1.06-0.99 (m, 1H), 0.95-0.85 (m, 1H), 0.62-0.54 (m, 1H), 0.41-0.34 (m, 1H);

^{13}C NMR ($CDCl_3$, 100 MHz) δ 206.9, 142.7, 140.1, 139.6, 139.3, 131.8, 131.4, 131.2, 131.1, 129.5, 128.0, 127.9, 127.1, 127.0, 126.7, 60.8, 21.0, 12.8;

HRMS(ESI) Calculated for $C_{21}H_{19}O_3NNaS_2^+$ ($[M+Na]^+$): 420.06986, found: 420.06958.

***N*-((2-(cyclohexanecarbonyl)phenyl)(phenyl)methyl)thiophene-2-sulfonamide (3o)**

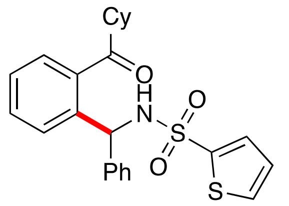

3o

Following a general procedure: To a 25 ml flame-dried Schlenk tube was added $ZnBr_2$ (0.5 mmol, 112.5 mg, stored in glove box), $MnBr(CO)_5$ (0.05 mmol, 10.0 mol%, 13.8 mg), DCM (5.0 mL), cyclohexyl(phenyl)methanone **1o** (2.0 mmol, 376.0 mg), (*E*)-*N*-benzylidenethiophene-2-sulfonamide **2a** (0.5 mmol, 125.5 mg) and Me_2Zn (0.75 mmol, 1.2 M in toluene, 0.625 mL) sequentially under nitrogen. The tube was sealed and stirred at r.t. for 1 h. After completion, the reaction mixture was diluted with ethyl acetate (5.0 mL) and filtered through a short pad silica gel washing with ethyl acetate (20 mL). The filtrate was concentrated and purified by silica gel column chromatography to provide the product **3o** in 58% yield.

^{1}H NMR ($CDCl_3$, 500 MHz) δ 7.57 (d, J = 7.5 Hz, 1H), 7.45-7.32 (m, 5H), 7.18-7.11 (m, 4H), 7.03 (d, J = 7.5 Hz, 2H), 6.90 (t, J = 4.5 Hz, 1H), 5.91 (d J = 9.0 Hz, 1H), 2.80 (t, J = 11.5 Hz, 1H), 1.69 (d, J = 8.5 Hz, 2H), 1.57-1.48 (m, 2H), 1.16-0.98 (m, 5H), 0.36-0.27 (m, 1H);

^{13}C NMR ($CDCl_3$, 125 MHz) δ 209.4, 142.8, 141.3, 140.0, 137.6, 131.9, 131.7, 131.6, 131.4, 129.5, 128.1, 127.9, 127.2, 127.1, 127.0, 61.1, 48.5, 28.5, 28.4, 25.9, 25.7, 25.5;

HRMS(ESI) Calculated for $C_{24}H_{25}O_3NNaS_2^+$ ($[M+Na]^+$): 462.11681, found:

462.11626.

***N*-((8-oxo-5,6,7,8-tetrahydronaphthalen-1-yl)(phenyl)methyl)thiophene-2-sulfonamide (3p)**

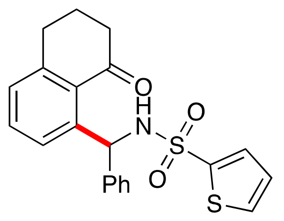
3p

Following a general procedure: To a 25 ml flame-dried Schlenk tube was added $ZnBr_2$ (0.5 mmol, 112.5 mg, stored in glove box), $MnBr(CO)_5$ (0.05 mmol, 10.0 mol%, 13.8 mg), DCM (5.0 mL), 3,4-dihydronaphthalen-1(*2H*)-one **1p** (2.0 mmol, 292.0 mg), (*E*)-*N*-benzylidenethiophene-2-sulfonamide **2a** (0.5 mmol, 125.5 mg) and Me_2Zn (0.75 mmol, 1.2 M in toluene, 0.625 mL) sequentially under nitrogen. The tube was sealed and stirred at 40 ^oC for 1 h. After completion, the reaction mixture was diluted with ethyl acetate (5.0 mL) and filtered through a short pad silica gel washing with ethyl acetate (20 mL). The filtrate was concentrated and purified by silica gel column chromatography to provide the product **3p** in 53% yield.

^{1}H NMR ($CDCl_3$, 500 MHz) δ 7.44 (m, 4H), 7.23-7.14 (m, 4H), 7.02 (d, J = 7.0 Hz, 2H), 6.91 (t, J = 4.5 Hz, 1H), 6.70 (br, 1H), 6.22 (br, 1H), 2.97-2.83 (m, 2H), 2.43 (t, J = 7.0 Hz, 2H), 1.98-1.95 (m, 2H);

^{13}C NMR ($CDCl_3$, 125 MHz) δ 200.6, 147.3, 142.5, 141.7, 140.5, 133.0, 132.1, 131.5, 130.5, 130.3, 129.5, 128.2, 127.1 (overlap), 126.5, 61.2, 40.6, 31.0, 22.6;

HRMS(ESI) Calculated for $C_{21}H_{19}O_3NNaS_2^+$ ($[M+Na]^+$): 420.06986, found: 420.06941.

***N*-((2-benzoylphenyl)(phenyl)methyl)thiophene-2-sulfonamide (3q)**

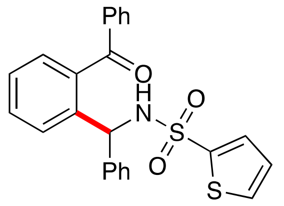
3q

Following a general procedure: To a 25 ml flame-dried Schlenk tube was added $ZnBr_2$ (0.5 mmol, 112.5 mg, stored in glove box), $MnBr(CO)_5$ (0.05 mmol, 10.0 mol%, 13.8 mg), DCE (1.25 mL), benzophenone **1q** (1.5 mmol, 252.0 mg), (*E*)-*N*-benzylidenethiophene-2-sulfonamide **2a** (0.5

mmol, 125.5 mg) and Me_2Zn (0.75 mmol, 1.2 M in toluene, 0.625 mL) sequentially under nitrogen. The tube was sealed and stirred at 60 °C for 10 h. After completion, the reaction mixture was diluted with ethyl acetate (5.0 mL) and filtered through a short pad silica gel washing with ethyl acetate (20 mL). The filtrate was concentrated and purified by silica gel column chromatography to provide the product **3q** in 65% yield.

^{1}H NMR ($CDCl_3$, 400 MHz) δ 7.46-7.40 (m, 7H), 7.30-7.23 (m, 4H), 7.06-6.68 (m, 4H), 6.94-6.87 (m, 2H), 7.69 (d, J = 8.8 Hz, 1H), 5.92 (d, J = 8.8 Hz, 1H);

^{13}C NMR ($CDCl_3$, 100 MHz) δ 199.3, 142.4, 141.6, 139.8, 137.8, 137.0, 133.3, 132.2, 131.6, 131.0, 130.6, 130.5, 130.4, 128.1, 128.1, 127.2, 127.1, 127.1, 60.5;

HRMS(ESI) Calculated for $C_{24}H_{19}O_3NNaS_2^+$ ($[M+Na]^+$): 456.06986, found: 456.06958.

***N*-((2-(2-methylbenzoyl)phenyl)(phenyl)methyl)thiophene-2-sulfonamide (3r)**

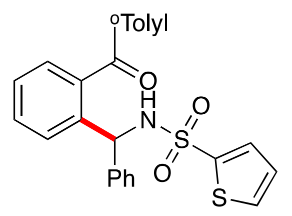

3r

Following a general procedure: To a 25 ml flame-dried Schlenk tube was added $ZnBr_2$ (0.5 mmol, 112.5 mg, stored in glove box), $MnBr(CO)_5$ (0.05 mmol, 10.0 mol%, 13.8 mg), DCE (1.25 mL), phenyl(*o*-tolyl)methanone **1r** (1.5 mmol, 294.0 mg), (*E*)-*N*-benzylidenethiophene-2-sulfonamide **2a** (0.5 mmol, 125.5 mg) and Me_2Zn (0.75 mmol, 1.2 M in toluene, 0.625 mL) sequentially under nitrogen. The tube was sealed and stirred at 60 °C for 10 h. After completion, the reaction mixture was diluted with ethyl acetate (5.0 mL) and filtered through a short pad silica gel washing with ethyl acetate (20 mL). The filtrate was concentrated and purified by silica gel column chromatography to provide the product **3r** in 82% yield.

^{1}H NMR ($CDCl_3$, 400 MHz) δ 7.53 (d, J = 7.6 Hz, 1H), 7.48 (dd, J_1 = 3.6 Hz, J_2 = 1.2 Hz, 1H), 7.45-7.41 (m, 2H), 7.29-7.22 (m, 2H), 7.19-6.96 (m, 9H), 6.93-6.89 (m, 1H), 6.77 (d, J = 7.2 Hz, 1H), 6.05 (d, J = 9.2 Hz, 1H), 2.18 (s, 3H);

^{13}C NMR (CDCl$_3$, 100 MHz) δ 201.3, 142.4, 141.8, 139.9, 138.4, 138.3, 138.1, 132.5, 132.2, 131.9, 131.6, 131.3, 131.2, 130.9, 130.4, 128.2, 127.5, 127.3, 127.2, 127.0, 125.2, 60.7, 20.5;

HRMS(ESI) Calculated for $C_{25}H_{21}O_3NNaS_2^+$ ([M+Na]$^+$): 470.08551, found: 470.08539.

***N*-(phenyl(1-(pyrimidin-2-yl)-1H-indol-2-yl)methyl)thiophene-2-sulfonamide (3s)**

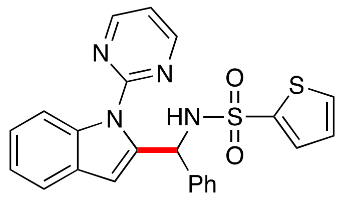

3s

Following a general procedure: To a 25 ml flame-dried Schlenk tube was added $ZnBr_2$ (0.5 mmol, 112.5 mg, stored in glove box), $MnBr(CO)_5$ (0.05 mmol, 10.0 mol%, 13.8 mg), DCE (1.25 mL), 1-(pyrimidin-2-yl)-1*H*-indole **1s** (1.5 mmol, 292.5 mg), (*E*)-*N*-benzylidenethiophene-2-sulfonamide **2a** (0.5 mmol, 125.5 mg) and Me_2Zn (0.75 mmol, 1.2 M in toluene, 0.625 mL) sequentially under nitrogen. The tube was sealed and stirred at 60 ^{o}C for 10 h. After completion, the reaction mixture was diluted with ethyl acetate (5.0 mL) and filtered through a short pad silica gel washing with ethyl acetate (20 mL). The filtrate was concentrated and purified by silica gel column chromatography to provide the product **3s** in 58% yield.

^{1}H NMR (CDCl$_3$, 400 MHz) δ 8.51 (d, J = 4.8 Hz, 2H), 8.21 (d, J = 8.4 Hz, 1H), 7.79 (d, J = 9.6 Hz, 1H), 7.51-7.46 (m, 2H), 7.31-7.25 (m, 2H), 7.20 (t, J = 7.2 Hz, 1H), 7.14 (d, J = 7.2 Hz, 2H), 7.07-7.00 (m, 3H), 6.92 (t, J = 4.8 Hz, 1H), 6.81-6.78 (m, 1H), 6.50 (s, 1H), 6.30 (d, J = 9.6 Hz, 1H);

^{13}C NMR (CDCl$_3$, 100 MHz) δ 158.0, 157.2, 142.5, 139.2, 137.4, 136.7, 132.1, 131.5, 128.4, 128.2, 127.2, 126.9, 126.1, 124.3, 122.5, 120.8, 117.1, 114.6, 111.4, 56.2;

HRMS(ESI) Calculated for $C_{23}H_{18}O_2N_4NaS_2^+$ ([M+Na]$^+$): 469.07634, found: 469.07587.

***N*-(phenyl(2-(pyrimidin-2-yl)phenyl)methyl)thiophene-2-sulfonamide (3t)**

Following a general procedure: To a 25 ml flame-dried Schlenk tube was added $ZnBr_2$

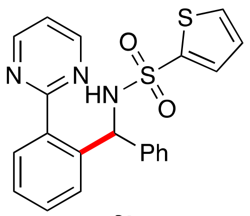

3t

(0.5 mmol, 112.5 mg, stored in glove box), $MnBr(CO)_5$ (0.05 mmol, 10.0 mol%, 13.8 mg), DCE (1.25 mL), 2-phenylpyrimidine **1t** (1.5 mmol, 237.0 mg), (*E*)-*N*-benzylidenethiophene-2-sulfonamide **2a** (0.5 mmol, 125.5 mg) and Me_2Zn (0.75 mmol, 1.2 M in toluene, 0.625 mL) sequentially under nitrogen. The tube was sealed and stirred at 60 °C for 10 h. After completion, the reaction mixture was diluted with ethyl acetate (5.0 mL) and filtered through a short pad silica gel washing with ethyl acetate (20 mL). The filtrate was concentrated and purified by silica gel column chromatography to provide the product **3t** in 61% yield.

^{1}H NMR ($CDCl_3$, 400 MHz) δ 8.60-8.56 (m, 3H), 7.82 (d, *J* = 7.6 Hz, 1H), 7.41-7.36 (m, 3H), 7.29-7.25 (m, 1H), 7.20 (d, *J* = 7.2 Hz, 1H), 7.04 (d, *J* = 7.2 Hz, 2H), 7.00-6.93 (m, 4H), 6.85 (t, *J* = 4.0 Hz, 1H), 6.00 (d, *J* = 9.6 Hz, 1H);

^{13}C NMR ($CDCl_3$, 100 MHz) δ 166.5, 156.6, 143.0, 140.4, 139.4, 137.4, 132.6, 131.8, 131.5, 131.3, 129.8, 128.3, 127.7, 126.9, 126.6, 126.2, 118.6, 61.4;

HRMS(ESI) Calculated for $C_{21}H_{17}O_2N_3NaS_2^+$ ([M+Na]$^+$): 430.06544, found: 430.06494.

N-((2-(1*H*-pyrazol-1-yl)phenyl)(phenyl)methyl)thiophene-2-sulfonamide (3u)

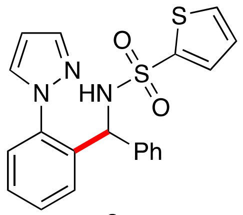

3u

Following a general procedure: To a 25 ml flame-dried Schlenk tube was added $ZnBr_2$ (0.5 mmol, 112.5 mg, stored in glove box), $MnBr(CO)_5$ (0.05 mmol, 10.0 mol%, 13.8 mg), DCE (1.25 mL), 1-phenyl-1*H*-pyrazole **1u** (1.5 mmol, 216.0 mg), (*E*)-*N*-benzylidenethiophene-2-sulfonamide **2a** (0.5 mmol, 125.5 mg) and Me_2Zn (0.75 mmol, 1.2 M in toluene, 0.625 mL) sequentially under nitrogen. The tube was sealed and stirred at 60 °C for 10 h. After completion, the reaction mixture was diluted with ethyl acetate (5.0 mL) and filtered through a short pad silica gel washing with ethyl acetate (20 mL). The filtrate was concentrated and purified by silica gel column chromatography to provide the product

3u in 52% yield.

^{1}H NMR (CDCl$_3$, 400 MHz) δ 8.16 (d, *J* = 9.2 Hz, 1H), 7.56 (s, 1H), 7.43 (d, *J* = 2.8 Hz, 1H), 7.36 (d, *J* = 4.8 Hz, 1H), 7.33-7.29 (m, 2H), 7.24 (d, *J* = 6.8 Hz, 1H), 7.12 (d, *J* = 7.6 Hz, 1H), 7.02-7.01 (m, 4H), 6.92-6.90 (m, 2H), 6.84 (t, *J* = 4.0 Hz, 1H), 6.08 (s, 1H), 5.79 (d, *J* = 9.6 Hz, 1H);

^{13}C NMR (CDCl$_3$, 100 MHz) δ 142.6, 140.4, 139.2, 138.7, 136.5, 131.9, 131.4, 131.3, 131.0, 129.0, 128.6, 127.8, 127.1, 127.0, 126.8, 125.4, 106.9, 59.9;

HRMS(ESI) Calculated for $C_{20}H_{17}O_2N_3NaS_2^+$ ([M+Na]$^+$): 418.06544, found: 418.06491.

***N*-((2-pivaloylphenyl)(p-tolyl)methyl)thiophene-2-sulfonamide (3v)**

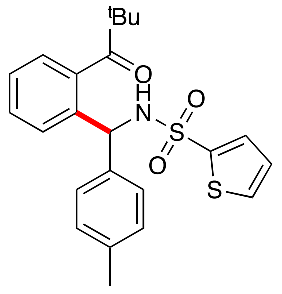

3v

Following a general procedure: To a 25 ml flame-dried Schlenk tube was added $ZnBr_2$ (0.5 mmol, 112.5 mg, stored in glove box), $MnBr(CO)_5$ (0.05 mmol, 10.0 mol%, 13.8 mg), DCE (1.25 mL), 2,2-dimethyl-1-phenylpropan-1-one **1a** (1.5 mmol, 243.0 mg), (*E*)-*N*-(4-methylbenzylidene)thiophene-2-sulfonamide **2b** (0.5 mmol, 132.5 mg) and Me_2Zn (0.75 mmol, 1.2 M in toluene, 0.625 mL) sequentially under nitrogen. The tube was sealed and stirred at 60 ^{o}C for 10 h. After completion, the reaction mixture was diluted with ethyl acetate (5.0 mL) and filtered through a short pad silica gel washing with ethyl acetate (20 mL). The filtrate was concentrated and purified by silica gel column chromatography to provide the product **3v** in 64% yield.

^{1}H NMR (CDCl$_3$, 400 MHz) δ 7.43-7.39 (m, 3H), 7.34-7.31(m, 1H), 7.26-7.23 (m, 2H), 7.00 (d, *J* = 8.4 Hz, 2H), 6.94 (d, *J* = 8.0 Hz, 2H), 6.90-6.87 (m, 1H), 6.65 (d, *J* = 8.8 Hz, 1H), 5.71 (d, *J* = 8.8 Hz, 1H), 2.25 (s, 3H), 0.91 (s, 9H);

^{13}C NMR (CDCl$_3$, 100 MHz) δ 214.8, 142.4, 139.7, 138.5, 137.2, 137.1, 132.0, 131.5, 130.8, 129.9, 129.0, 127.1, 126.9 (overlap), 126.8, 60.5, 44.3, 28.1, 21.0;

HRMS(ESI) Calculated for $C_{23}H_{25}O_3NNaS_2^+$ ([M+Na]$^+$): 450.11681, found: 450.11642.

***N*-((4-methoxyphenyl)(2-pivaloylphenyl)methyl)thiophene-2-sulfonamide (3w)**

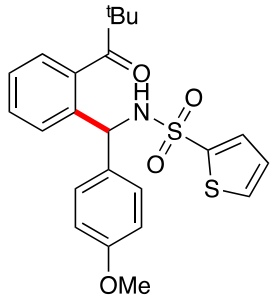

3w

Following a general procedure: To a 25 ml flame-dried Schlenk tube was added $ZnBr_2$ (0.5 mmol, 112.5 mg, stored in glove box), $MnBr(CO)_5$ (0.05 mmol, 10.0 mol%, 13.8 mg), DCE (1.25 mL), 2,2-dimethyl- 1-phenylpropan-1-one **1a** (1.5 mmol, 243.0 mg), (*E*)-*N*-(4-methoxybenzylidene) thiophene-2-sulfonamide **2c** (0.5 mmol, 140.5 mg) and Me_2Zn (0.75 mmol, 1.2 M in toluene, 0.625 mL) sequentially under nitrogen. The tube was sealed and stirred at 60 °C for 10 h. After completion, the reaction mixture was diluted with ethyl acetate (5.0 mL) and filtered through a short pad silica gel washing with ethyl acetate (20 mL). The filtrate was concentrated and purified by silica gel column chromatography to provide the product **3w** in 76% yield.

^{1}H NMR ($CDCl_3$, 400 MHz) δ 7.43-7.38 (m, 3H), 7.34-7.31 (m, 1H), 7.26-7.23 (m, 2H), 6.97 (d, J = 8.4 Hz, 2H), 6.89 (t, J = 4.0 Hz, 1H), 6.72 (d, J = 8.4 Hz, 2H), 6.63 (d, J = 8.8 Hz, 1H), 5.70 (d, J = 8.8 Hz, 1H), 3.72 (s, 3H), 0.94 (s, 9H);

^{13}C NMR ($CDCl_3$, 100 MHz) δ 214.8, 158.9, 142.2, 139.5, 138.4, 132.1, 132.1, 131.6, 130.5, 129.9, 128.4, 127.1, 126.8, 126.8, 113.7, 60.0, 55.4, 44.3, 28.1;

HRMS(ESI) Calculated for $C_{23}H_{25}O_4NNaS_2^+$ ([M+Na]$^+$): 466.11172, found: 466.11139.

***N*-((4-fluorophenyl)(2-pivaloylphenyl)methyl)thiophene-2-sulfonamide (3x)**

Following a general procedure: To a 25 ml flame-dried Schlenk tube was added $ZnBr_2$ (0.5 mmol, 112.5 mg, stored in glove box), $MnBr(CO)_5$ (0.05 mmol, 10.0 mol%, 13.8 mg), DCE (1.25 mL), 2,2-dimethyl- 1-phenylpropan-1-one **1a** (1.5 mmol, 243.0 mg), (*E*)-*N*-(4-fluorobenzylidene) thiophene-2-sulfonamide **2d** (0.5 mmol, 135.0 mg) and Me_2Zn (0.75 mmol, 1.2 M in toluene, 0.625 mL) sequentially under nitrogen. The

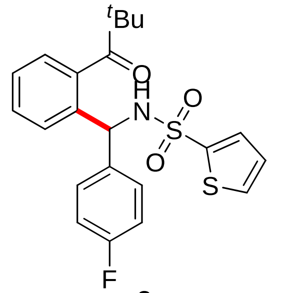

3x

tube was sealed and stirred at 60 ^{o}C for 10 h. After completion, the reaction mixture was diluted with ethyl acetate (5.0 mL) and filtered through a short pad silica gel washing with ethyl acetate (20 mL). The filtrate was concentrated and purified by silica gel column chromatography to provide the product **3x** in 56% yield.

^{1}H NMR ($CDCl_3$, 400 MHz) δ 7.46-7.42 (m, 3H), 7.27 (d, J = 2.4 Hz, 3H), 7.10-7.06 (m, 2H), 6.90 (t, J = 8.0 Hz, 3H), 6.80 (d, J = 8.8 Hz, 1H), 5.70 (d, J = 8.8 Hz, 1H), 0.93 (s, 9H);

^{13}C NMR ($CDCl_3$, 100 MHz) δ 214.9, 162.1 (d, $^{1}J_{C\text{-}F}$ = 245.2 Hz), 142.4, 139.5, 138.3, 135.9 (d, $^{4}J_{C\text{-}F}$ = 3.0 Hz), 132.1, 131.7, 131.1, 130.2, 128.8 (d, $^{3}J_{C\text{-}F}$ = 8.1 Hz), 127.3, 127.2, 115.2 (d, $^{2}J_{C\text{-}F}$ = 21.4 Hz), 60.4, 44.4, 28.2;

^{19}F NMR ($CDCl_3$, 565 MHz) δ -115.1;

HRMS(ESI) Calculated for $C_{22}H_{22}O_3NFNaS_2^+$ ([M+Na]$^+$): 454.09173, found: 454.09128.

***N*-((4-chlorophenyl)(2-pivaloylphenyl)methyl)thiophene-2-sulfonamide (3y)**

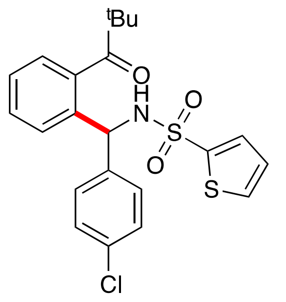

3y

Following a general procedure: To a 25 ml flame-dried Schlenk tube was added $ZnBr_2$ (0.5 mmol, 112.5 mg, stored in glove box), $MnBr(CO)_5$ (0.05 mmol, 10.0 mol%, 13.8 mg), DCE (1.25 mL), 2,2-dimethyl-1-phenylpropan-1-one **1a** (1.5 mmol, 243.0 mg), (*E*)-*N*-(4-chlorobenzylidene)thiophene-2-sulfonamide **2e** (0.5 mmol, 142.5 mg) and Me_2Zn (0.75 mmol, 1.2 M in toluene, 0.625 mL) sequentially under nitrogen. The tube was sealed and stirred at 60 ^{o}C for 10 h. After completion, the reaction mixture was diluted with ethyl acetate (5.0 mL) and filtered through a short pad silica gel washing with ethyl acetate (20 mL). The filtrate was concentrated and purified by silica gel column chromatography to provide the product **3y** in 45% yield.

^{1}H NMR (CDCl$_3$, 400 MHz) δ 7.46-7.43 (m, 3H), 7.30-7.27 (m, 3H), 7.19 (d, *J* = 8.0 Hz, 2H), 7.06 (d, *J* = 8.0 Hz, 2H), 6.91 (t, *J* = 4.0 Hz, 1H), 6.82 (d, *J* = 9.2 Hz, 1H), 5.69 (d, *J* = 8.8 Hz, 1H), 0.93 (s, 9H);

^{13}C NMR (CDCl$_3$, 100 MHz) δ 214.9, 142.4, 139.3, 138.7, 138.3, 133.4, 132.2, 131.7, 131.2, 130.3, 128.5, 128.4, 127.4, 127.2, 127.2, 60.5, 44.4, 28.2;

HRMS(ESI) Calculated for $C_{22}H_{22}O_3NClNaS_2^+$ ($[M+Na]^+$): 470.06218, found: 470.06174.

***N*-((4-bromophenyl)(2-pivaloylphenyl)methyl)thiophene-2-sulfonamide (3z)**

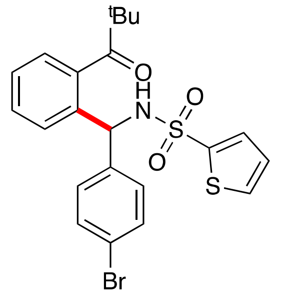

3z

Following a general procedure: To a 25 ml flame-dried Schlenk tube was added $ZnBr_2$ (0.5 mmol, 112.5 mg, stored in glove box), $MnBr(CO)_5$ (0.05 mmol, 10.0 mol%, 13.8 mg), DCE (1.25 mL), 2,2-dimethyl-1-phenylpropan-1-one **1a** (1.5 mmol, 243.0 mg), (*E*)-*N*-(4-bromobenzylidene)thiophene-2-sulfonamide **2f** (0.5 mmol, 164.5 mg) and Me_2Zn (0.75 mmol, 1.2 M in toluene, 0.625 mL) sequentially under nitrogen. The tube was sealed and stirred at 60 °C for 10 h. After completion, the reaction mixture was diluted with ethyl acetate (5.0 mL) and filtered through a short pad silica gel washing with ethyl acetate (20 mL). The filtrate was concentrated and purified by silica gel column chromatography to provide the product **3z** in 55% yield.

^{1}H NMR (CDCl$_3$, 400 MHz) δ 7.46-7.40 (m, 3H), 7.33 (d, *J* = 8.4 Hz, 2H), 7.28-7.25 (m, 3H), 6.98 (d, *J* = 8.4 Hz, 2H), 6.91-6.88 (m, 1H), 6.79 (d, *J* = 9.2 Hz, 1H), 5.66 (d, *J* = 9.2 Hz, 1H), 0.92 (s, 9H);

^{13}C NMR (CDCl$_3$, 100 MHz) δ 214.8, 142.4, 139.2, 138.3, 132.2, 131.7, 131.4, 131.2, 130.3, 128.9, 127.4, 127.3, 127.2, 121.5, 60.5, 44.4, 28.2;

HRMS(ESI) Calculated for $C_{22}H_{22}O_3NBrNaS_2^+$ ($[M+Na]^+$): 514.01167, found: 514.01114.

***N*-((2-pivaloylphenyl)(4-(trifluoromethyl)phenyl)methyl)thiophene-2-sulfonamide (3A)**

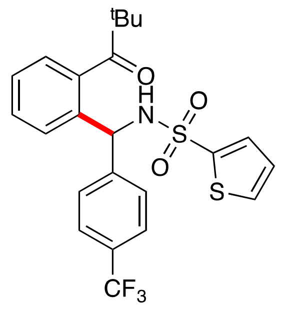

3A

Following a general procedure: To a 25 ml flame-dried Schlenk tube was added $ZnBr_2$ (0.5 mmol, 112.5 mg, stored in glove box), $MnBr(CO)_5$ (0.05 mmol, 10.0 mol%, 13.8 mg), DCE (1.25 mL), 2,2-dimethyl-1-phenylpropan-1-one **1a** (1.5 mmol, 243.0 mg), (*E*)-*N*-(4-(trifluoromethyl)benzylidene)thiophene-2-sulfonamide **2g** (0.5 mmol, 159.5 mg) and Me_2Zn (0.75 mmol, 1.2 M in toluene, 0.625 mL) sequentially under nitrogen. The tube was sealed and stirred at 60 °C for 10 h. After completion, the reaction mixture was diluted with ethyl acetate (5.0 mL) and filtered through a short pad silica gel washing with ethyl acetate (20 mL). The filtrate was concentrated and purified by silica gel column chromatography to provide the product **3A** in 42% yield.

^{1}H NMR ($CDCl_3$, 400 MHz) δ 7.49-7.42 (m, 5H), 7.31-7.26 (m, 5H), 6.95-6.90 (m, 2H), 5.77 (d, *J* = 9.2 Hz, 1H), 0.90 (s, 9H);

^{13}C NMR ($CDCl_3$, 100 MHz) δ 214.7, 144.2, 142.4, 139.2, 138.2, 132.2, 131.8, 131.5, 130.5, 129.8 (q, $^2J_{C-F}$ = 32.3 Hz), 127.6, 127.5, 127.5, 127.2, 125.3 (q, $^3J_{C-F}$ = 3.7 Hz), 124.1 (q, $^1J_{C-F}$ = 270.3 Hz), 60.8, 44.4, 28.2;

^{19}F NMR ($CDCl_3$, 565 MHz) δ -62.6;

HRMS(ESI) Calculated for $C_{23}H_{22}O_3NF_3NaS_2^+$ ($[M+Na]^+$): 504.08854, found: 504.08798.

***N*-((2-pivaloylphenyl)(o-tolyl)methyl)thiophene-2-sulfonamide (3B)**

Following a general procedure: To a 25 ml flame-dried Schlenk tube was added $ZnBr_2$ (0.5 mmol, 112.5 mg, stored in glove box), $MnBr(CO)_5$ (0.05 mmol, 10.0 mol%, 13.8 mg), DCE (1.25 mL), 2,2-dimethyl-1- phenylpropan-1-one **1a** (1.5 mmol, 243.0 mg), (*E*)-*N*-(2-methylbenzylidene) thiophene-2-sulfonamide **2h** (0.5 mmol, 132.5 mg) and

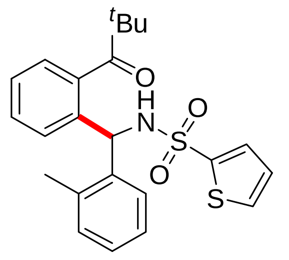

3B

Me_2Zn (0.75 mmol, 1.2 M in toluene, 0.625 mL) sequentially under nitrogen. The tube was sealed and stirred at 60 °C for 10 h. After completion, the reaction mixture was diluted with ethyl acetate (5.0 mL) and filtered through a short pad silica gel washing with ethyl acetate (20 mL). The filtrate was concentrated and purified by silica gel column chromatography to provide the product **3B** in 63% yield.

^{1}H NMR ($CDCl_3$, 400 MHz) δ 7.50-7.42 (m, 3H), 7.36 (d, *J* = 7.2 Hz, 1H), 7.29-7.20 (m, 2H), 7.12-6.99 (m, 3H), 6.95-6.88 (m, 2H), 6.09 (d, *J* = 7.6 Hz, 1H), 5.77 (d, *J* = 7.6 Hz, 1H), 2.06 (s, 3H), 0.91 (s, 9H);

^{13}C NMR ($CDCl_3$, 100 MHz) δ 213.8, 141.9, 139.0, 138.2, 137.7, 136.5, 132.5, 131.7, 131.0, 129.8, 129.4, 128.5, 128.1, 127.3, 126.5, 126.2, 126.2, 56.6, 44.4, 28.0, 19.6;

HRMS(ESI) Calculated for $C_{23}H_{25}O_3NNaS_2^+$ ($[M+Na]^+$): 450.11681, found: 450.11630.

_N_-((2-pivaloylphenyl)(m-tolyl)methyl)thiophene-2-sulfonamide (3C)

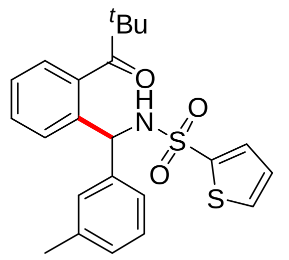

3C

Following a general procedure: To a 25 ml flame-dried Schlenk tube was added $ZnBr_2$ (0.5 mmol, 112.5 mg, stored in glove box), $MnBr(CO)_5$ (0.05 mmol, 10.0 mol%, 13.8 mg), DCE (1.25 mL), 2,2-dimethyl-1-phenylpropan-1-one **1a** (1.5 mmol, 243.0 mg), (*E*)-*N*-(3-methylbenzylidene)thiophene-2-sulfonamide **2i** (0.5 mmol, 132.5 mg) and Me_2Zn (0.75 mmol, 1.2 M in toluene, 0.625 mL) sequentially under nitrogen. The tube was sealed and stirred at 60 °C for 10 h. After completion, the reaction mixture was diluted with ethyl acetate (5.0 mL) and filtered through a short pad silica gel washing with ethyl acetate (20 mL). The filtrate was concentrated and purified by silica gel column chromatography to provide the product **3C** in 77% yield.

^{1}H NMR ($CDCl_3$, 400 MHz) δ 7.43-7.39 (m, 3H), 7.31-7.23 (m, 3H), 7.07 (t, *J* = 8.0

Hz, 1H), 6.96 (d, *J* = 7.6 Hz, 1H), 6.89-6.85 (m, 3H), 6.79 (d, *J* = 8.8 Hz, 1H), 5.73 (d, *J* = 9.2 Hz, 1H), 2.20 (s, 3H), 0.88 (s, 9H);

^{13}C NMR ($CDCl_3$, 100 MHz) δ 214.9, 142.5, 139.8, 139.6, 138.4, 137.8, 131.9, 131.4, 131.0, 130.0, 128.2, 128.1, 127.7, 127.0, 127.0, 126.9, 124.2, 60.7, 44.2, 28.0, 21.3;

HRMS(ESI) Calculated for $C_{23}H_{25}O_3NNaS_2^+$ ($[M+Na]^+$): 450.11681, found: 450.11634.

***N*-(naphthalen-1-yl(2-pivaloylphenyl)methyl)thiophene-2-sulfonamide (3D)**

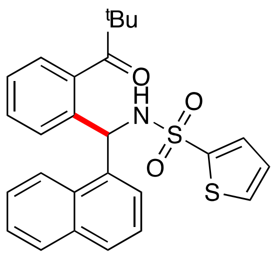

3D

Following a general procedure: To a 25 ml flame-dried Schlenk tube was added $ZnBr_2$ (0.5 mmol, 112.5 mg, stored in glove box), $MnBr(CO)_5$ (0.05 mmol, 10.0 mol%, 13.8 mg), DCE (1.25 mL), 2,2-dimethyl-1-phenylpropan-1-one **1a** (1.5 mmol, 243.0 mg), (*E*)-*N*-(naphthalen-1-ylmethylene)thiophene-2-sulfonamide **2j** (0.5 mmol, 150.5 mg) and Me_2Zn (0.75 mmol, 1.2 M in toluene, 0.625 mL) sequentially under nitrogen. The tube was sealed and stirred at 60 ^oC for 10 h. After completion, the reaction mixture was diluted with ethyl acetate (5.0 mL) and filtered through a short pad silica gel washing with ethyl acetate (20 mL). The filtrate was concentrated and purified by silica gel column chromatography to provide the product **3D** in 68% yield.

^{1}H NMR ($CDCl_3$, 400 MHz) δ 7.79 (t, *J* = 7.6 Hz, 2H), 7.71 (d, *J* = 8.0 Hz, 1H), 6.61 (d, *J* = 8.0 Hz, 1H), 7.78-7.24 (m, 8H), 7.12 (d, *J* = 7.2 Hz, 1H), 6.90 (t, *J* = 4.4 Hz, 1H), 6.69 (d, *J* = 7.6 Hz, 1H), 5.81 (d, *J* = 7.2 Hz, 1H), 0.75 (s, 9H);

^{13}C NMR ($CDCl_3$, 100 MHz) δ 213.9, 141.8, 139.4, 138.6, 135.0, 134.0, 132.8, 131.9, 130.7, 129.8, 129.6, 129.1, 128.8, 127.3, 127.1, 126.9, 126.7, 126.1, 125.9, 125.1, 123.4, 56.1, 44.3, 27.9;

HRMS(ESI) Calculated for $C_{26}H_{25}O_3NNaS_2^+$ ($[M+Na]^+$): 486.11681, found: 486.11624.

N-(naphthalen-2-yl(2-pivaloylphenyl)methyl)thiophene-2-sulfonamide (3E)

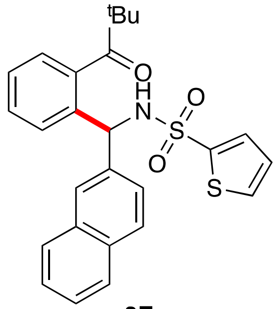
3E

Following a general procedure: To a 25 ml flame-dried Schlenk tube was added $ZnBr_2$ (0.5 mmol, 112.5 mg, stored in glove box), $MnBr(CO)_5$ (0.05 mmol, 10.0 mol%, 13.8 mg), DCE (1.25 mL), 2,2-dimethyl-1-phenylpropan-1-one **1a** (1.5 mmol, 243.0 mg), (*E*)-*N*-(naphthalen-2-ylmethylene)thiophene-2-sulfonamide **2k** (0.5 mmol, 150.5 mg) and Me_2Zn (0.75 mmol, 1.2 M in toluene, 0.625 mL) sequentially under nitrogen. The tube was sealed and stirred at 60 °C for 10 h. After completion, the reaction mixture was diluted with ethyl acetate (5.0 mL) and filtered through a short pad silica gel washing with ethyl acetate (20 mL). The filtrate was concentrated and purified by silica gel column chromatography to provide the product **3E** in 74% yield.

^{1}H NMR (CDCl$_3$, 400 MHz) δ 7.74-7.71 (m, 1H), 7.68-7.64 (m, 2H), 7.56 (s, 1H), 7.45-7.37 (m, 6H), 7.31-7.24 (m, 2H), 7.14 (dd, J_1 = 8.4 Hz, J_2 = 1.2 Hz, 1H), 6.90-6.85 (m, 2H), 5.92 (d, J = 9.2 Hz, 1H), 0.77 (s, 9H);

^{13}C NMR (CDCl$_3$, 100 MHz) δ 214.9, 142.5, 139.6, 138.6, 137.2, 133.0, 132.6, 132.1, 131.6, 131.2, 130.2, 128.1, 128.1, 127.6, 127.1, 127.1, 127.1, 126.5, 126.2, 126.0, 125.2, 61.0, 44.3, 28.1;

HRMS(ESI) Calculated for $C_{26}H_{25}O_3NNaS_2^+$ ([M+Na]$^+$): 486.11681, found: 486.11636.

N-(furan-2-yl(2-pivaloylphenyl)methyl)thiophene-2-sulfonamide (3F)

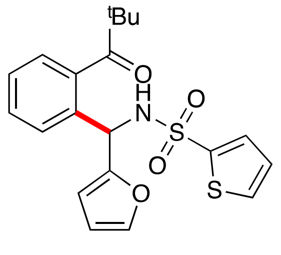
3F

Following a general procedure: To a 25 ml flame-dried Schlenk tube was added $ZnBr_2$ (0.5 mmol, 112.5 mg, stored in glove box), $MnBr(CO)_5$ (0.05 mmol, 10.0 mol%, 13.8 mg), DCE (1.25 mL), 2,2-dimethyl-1-phenylpropan-1-one **1a** (1.5 mmol, 243.0 mg),

(*E*)-*N*-(furan-2-ylmethylene)thiophene-2-sulfonamide **2l** (0.5 mmol, 120.5 mg) and Me_2Zn (0.75 mmol, 1.2 M in toluene, 0.625 mL) sequentially under nitrogen. The tube was sealed and stirred at 60 °C for 10 h. After completion, the reaction mixture was diluted with ethyl acetate (5.0 mL) and filtered through a short pad silica gel washing with ethyl acetate (20 mL). The filtrate was concentrated and purified by silica gel column chromatography to provide the product **3F** in 68% yield.

^{1}H NMR ($CDCl_3$, 400 MHz) δ 7.45-7.42 (m, 3H), 7.37-7.34 (m, 1H), 7.29-7.26 (m, 2H), 7.18 (s, 1H), 6.90 (t, *J* = 4.4 Hz, 1H), 6.54 (d, *J* = 8.4 Hz, 1H), 6.21 (s, 1H), 6.07 (d, *J* = 2.8 Hz, 1H), 5.70 (d, *J* = 8.4 Hz, 1H), 1.13 (s, 9H);

^{13}C NMR ($CDCl_3$, 100 MHz) δ 214.4, 152.2, 142.3, 141.9, 138.8, 136.6, 132.2, 131.8, 130.3, 130.1, 127.3, 127.1, 126.4, 110.7, 108.5, 55.3, 44.7, 28.2;

HRMS(ESI) Calculated for $C_{20}H_{21}O_4NNaS_2^+$ ([M+Na]$^+$): 426.08042, found: 426.07999.

4-methyl-*N*-(phenyl(2-pivaloylphenyl)methyl)benzenesulfonamide (3G)

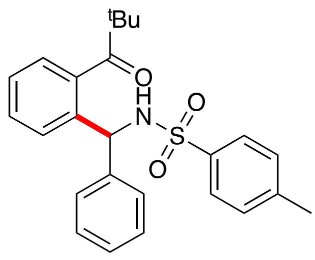

3G

Following a general procedure: To a 25 ml flame-dried Schlenk tube was added $ZnBr_2$ (0.5 mmol, 112.5 mg, stored in glove box), $MnBr(CO)_5$ (0.05 mmol, 10.0 mol%, 13.8 mg), DCE (1.25 mL), 2,2-dimethyl-1-phenylpropan-1-one **1a** (1.5 mmol, 243.0 mg), (*E*)-*N*-benzylidene-4-methylbenzenesulfonamide **2m** (0.5 mmol, 129.5 mg) and Me_2Zn (0.75 mmol, 1.2 M in toluene, 0.625 mL) sequentially under nitrogen. The tube was sealed and stirred at 60 °C for 10 h. After completion, the reaction mixture was diluted with ethyl acetate (5.0 mL) and filtered through a short pad silica gel washing with ethyl acetate (20 mL). The filtrate was concentrated and purified by silica gel column chromatography to provide the product **3G** in 70% yield.

^{1}H NMR ($CDCl_3$, 400 MHz) δ 7.58 (d, *J* = 10.8 Hz, 2H), 7.38 (dd, J_1 = 10.4 Hz, J_2 = 2.4 Hz, 1H), 7.22-7.13 (m, 6H), 7.10-7.06 (m, 4H), 6.66 (d, *J* = 12.4 Hz, 1H), 5.66 (d,

J = 12.4 Hz, 1H), 2.33 (s, 3H), 0.88 (s, 9H);

^{13}C NMR ($CDCl_3$, 100 MHz) δ 214.9, 142.9, 140.2, 139.7, 138.4, 138.2, 131.2, 129.8, 129.3, 128.2, 127.3, 127.2, 127.0, 126.9, 126.6, 60.5, 44.2, 28.1, 21.5;

HRMS(ESI) Calculated for $C_{25}H_{27}O_3NNaS_2^+$ ([M+Na]$^+$): 444.16039, found: 444.15991.

Of note, we have also tested several imines bearing varied substituents on nitrogen. It turned out that the sulfonyl groups are crucial for the success of the desired reactions with substrates containing other protecting groups failed in the reaction. It was proposed that the strong electron-withdrawing property of the sulfonyl groups accelerated the expected reactions much more than that of the *N*-sulfinyl or phenyl groups.

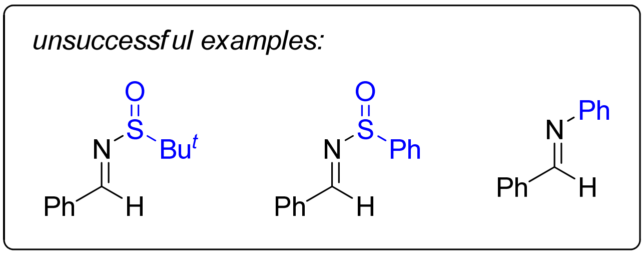

In addition, the ketimine was shown ineffective in the reaction, presumably due to its enhanced steric hindrance.

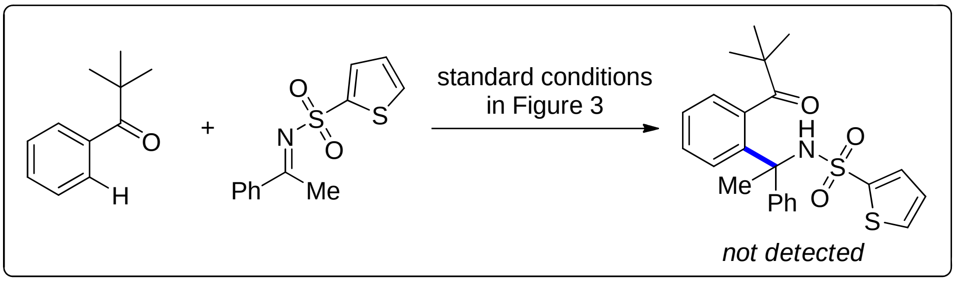

N-(((S)-7-methyl-8-oxo-7-phenyl-5,6,7,8-tetrahydronaphthalen-1-yl)(phenyl)methyl)thiophene-2-sulfonamide (3H)

Following a general procedure: To a 25 ml flame-dried Schlenk tube was added $ZnBr_2$ (0.5 mmol, 112.5 mg, stored in glove box), $MnBr(CO)_5$ (0.05 mmol, 10.0 mol%, 13.8 mg), DCM (5.0 mL), (S)-2-methyl-2-phenyl-3,4- dihydronaphthalen-1(2H)-one **1C**

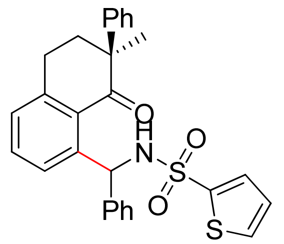

3H

(2.0 mmol, 292.0 mg, 96% ee), (*E*)-*N*-benzylidenethiophene- 2-sulfonamide **2a** (0.5 mmol, 125.5 mg) and Me_2Zn (0.75 mmol, 1.2 M in toluene, 0.625 mL) sequentially under nitrogen. The tube was sealed and stirred at 40 °C for 1 h. After completion, the reaction mixture was diluted with ethyl acetate (5.0 mL) and filtered through a short pad silica gel washing with ethyl acetate (20 mL). The filtrate was concentrated and purified by silica gel column chromatography to provide the diastereoisomers **3H** in 66% combined yield with a *dr* value of 9.4.

Characterization of the major diastereoisomer

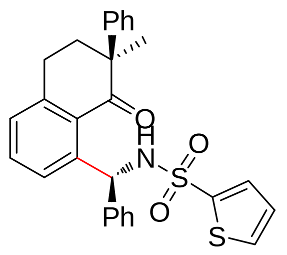

3H-major: 96% *ee*

The isolation of diastereoisomers was performed on a preparative HPLC LC3000 using CH_2Cl_2 as an eluent. The structure of major diastereoisomer was confirmed by NMR, HRMS and single-crystal X-ray diffraction analysis. **^{1}H NMR ($CDCl_3$, 500 MHz)** δ 7.73 (d, *J* = 6.5 Hz, 1H), 7.62 (d, *J* = 3.5 Hz, 1H), 7.54 (d, *J* = 5.0 Hz, 1H), 7.41 (t, *J* = 7.5 Hz, 1H), 7.23-7.16 (m, 3H), 7.10-7.03 (m, 3H), 6.99 (t, *J* = 7.5 Hz, 2H), 6.91 (d, *J* = 7.0 Hz, 2H), 6.84 (br, 1H), 6.53 (d, *J* = 6.0 Hz, 2H), 5.88 (br, 1H), 2.91-2.78 (m, 2H), 2.44 (dt, J_1 = 14.0 Hz, J_2 = 4.0 Hz, 1H), 2.16-2.09 (m, 1H), 1.19 (s, 3H);

^{13}C NMR ($CDCl_3$, 125 MHz) δ 203.6, 145.8, 142.8, 142.5, 141.9, 140.5, 132.6, 132.5, 131.9, 130.5, 129.1, 128.5, 128.4, 127.9, 127.5, 127.4, 126.5, 126.2, 59.8, 51.6, 35.3, 27.2, 27.0;

HRMS(ESI) Calculated for $C_{28}H_{25}O_3NNaS_2^+$ ($[M+Na]^+$): 510.11681, found: 510.11657.

The ee value of the major product was determined to be 96% by chiral HPLC analysis (Daicel CHIRALCEL ID; hexanes: *i*-PrOH = 70:30; detection wavelengths = 254 nm; flow rate = 1.0 mL/min). TR = 11.3 min (minor) and 15.8 min (major).

a

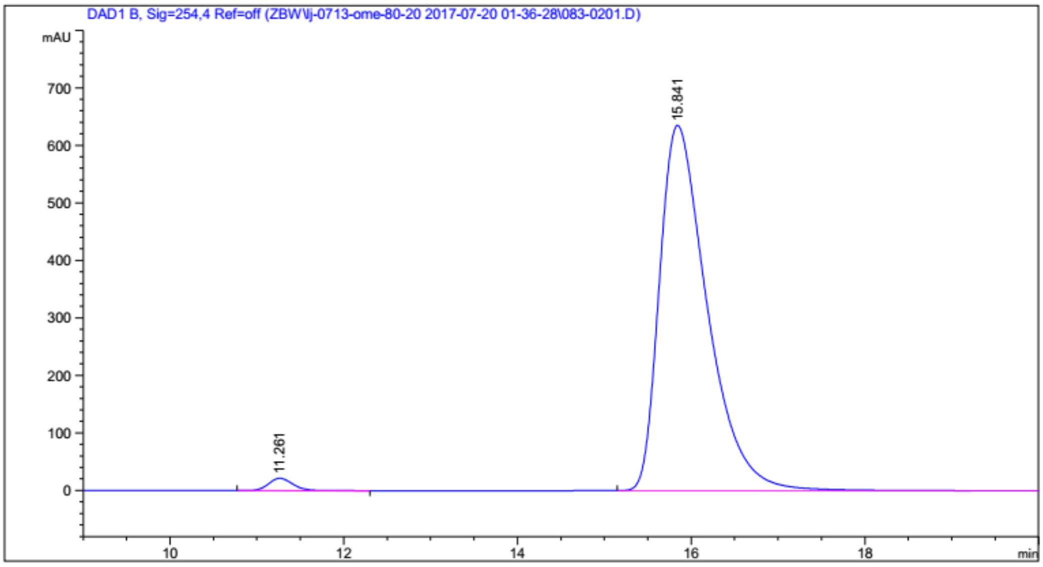

Peak #	RetTime [min]	Type	Width [min]	Area [mAU*s]	Height [mAU]	Area %
1	11.261	BB	0.3026	423.75754	21.62508	1.7172
2	15.841	BB	0.5815	2.42537e4	635.17731	98.2828

b

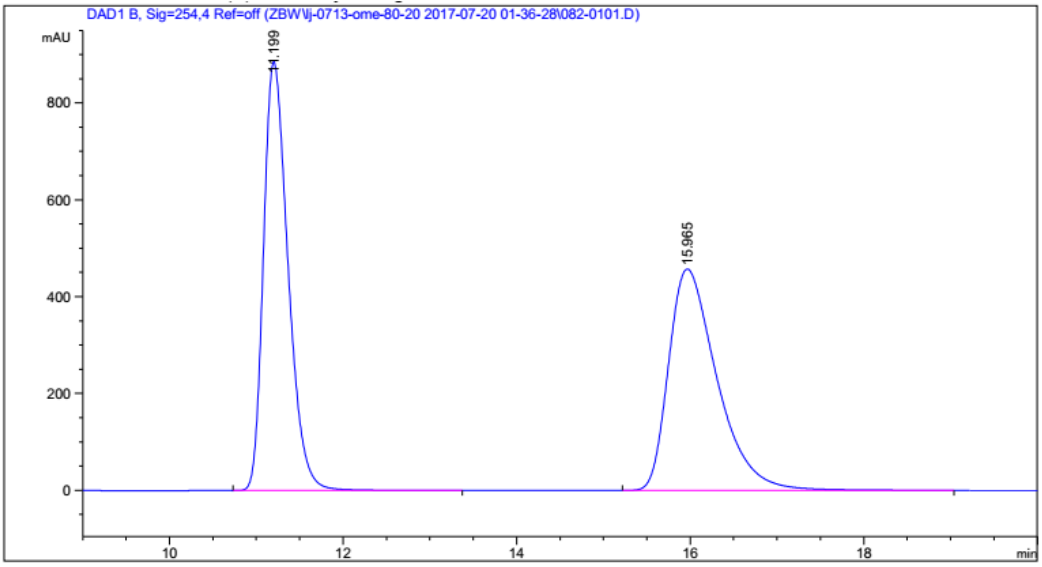

Peak #	RetTime [min]	Type	Width [min]	Area [mAU*s]	Height [mAU]	Area %
1	11.199	BB	0.3024	1.73421e4	885.64349	49.4907
2	15.965	BB	0.5897	1.76990e4	457.16632	50.5093

Supplementary Figure 8. Chiral separation for major diastereoisomer

(a) HPLC spectrum of 3H-major. (b) HPLC spectrum of a racemic sample

Characterization of the minor diastereoisomer

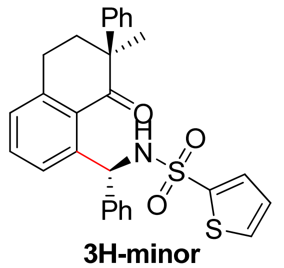

3H-minor

^{1}H NMR ($CDCl_3$, 500 MHz) δ 7.40-7.37 (m, 2H), 7.31-7.28 (m, 2H), 7.25-7.23 (m, 3H), 7.16-7.14 (m, 2H), 7.08-7.00 (m, 4H), 6.86 (t, J = 4.5 Hz, 1H), 6.41 (br, 2H), 6.06 (br, 1H), 2.94-2.79 (m, 2H), 2.53 (dt, J_1 = 14.0 Hz, J_2 = 4.0 Hz, 1H), 2.15-2.08 (m, 1H), 1.26 (s, 3H);

^{13}C NMR ($CDCl_3$, 125 MHz) δ 203.8, 146.4, 143.0, 142.1, 141.6, 139.5, 132.7, 131.9, 131.3, 130.9, 129.7, 128.7, 128.1, 127.0, 126.9, 126.8, 126.6, 125.9, 62.4, 51.6, 34.8, 28.1, 27.3;

HRMS(ESI) Calculated for $C_{28}H_{25}O_3NNaS_2^+$ ([M+Na]$^+$): 510.11681, found: 510.11657.

1-methylene-3-phenyl-2-(thiophen-2-ylsulfonyl)isoindoline (4a)

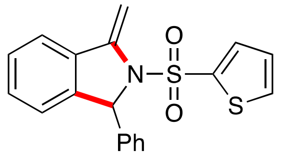

4a

Following a general procedure: To a Schlenk tube was added $ZnBr_2$ (0.5 mmol, 112.5 mg), $MnBr(CO)_5$ (0.05 mmol, 10.0 mol%, 13.8 mg), DCM (5.0 mL), acetophenone **1v** (2.0 mmol, 240.0 mg), (*E*)-*N*-benzylidene thiophene-2-sulfonamide **2a** (0.5 mmol, 125.5 mg) and Me_2Zn (0.75 mmol, 1.2 M in toluene, 0.625 mL) sequentially under nitrogen. The tube was sealed and stirred at 60 °C for 2 h. After completion, the reaction mixture was diluted with ethyl acetate (10 mL) and filtered through a short pad silica gel washing with ethyl acetate (20 mL). The filtrate was concentrated and purified by silica gel column chromatography to provide **4a** in 66% yield.

^{1}H NMR ($CDCl_3$, 400 MHz) δ 7.50-7.42 (m, 3H), 7.31-7.19 (m, 7H), 6.99-6.93 (m, 2H), 6.12 (s, 1H), 5.44 (d, J = 1.6 Hz, 1H), 5.19 (d, J = 1.6 Hz, 1H);

^{13}C NMR ($CDCl_3$, 100 MHz) δ 143.9, 140.8, 140.5, 138.1, 133.8, 132.7, 132.3, 129.9, 128.7, 128.4, 128.2, 127.4, 127.1, 123.5, 120.6, 89.4, 70.6;

HRMS(ESI) Calculated for $C_{19}H_{16}O_2NS_2^+$ ([M+H]$^+$): 354.06170, found: 354.06133.

5-methyl-1-methylene-3-phenyl-2-(thiophen-2-ylsulfonyl)isoindoline (4b)

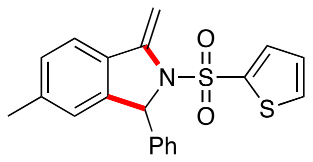

4b

Following a general procedure: To a Schlenk tube was added $ZnBr_2$ (0.5 mmol, 112.5 mg), $MnBr(CO)_5$ (0.05 mmol, 10.0 mol%, 13.8 mg), DCM (5.0 mL), 1-*p*-tolylethanone **1w** (2.0 mmol, 268.0 mg), (*E*)-*N*-benzylidenethiophene-2-sulfonamide **2a** (0.5 mmol, 125.5 mg) and Me_2Zn (0.75 mmol, 1.2 M in toluene, 0.625 mL) sequentially under nitrogen. The tube was sealed and stirred at 60 °C for 2 h. After completion, the reaction mixture was diluted with ethyl acetate (10 mL) and filtered through a short pad silica gel washing with ethyl acetate (20 mL). The filtrate was concentrated and purified by silica gel column chromatography to provide **4b** in 62 % yield.

^{1}H NMR ($CDCl_3$, 400 MHz) δ 7.48 (dd, J_1 = 5.2 Hz, J_2 = 1.2 Hz, 1H), 7.44 (dd, J_1 = 3.6 Hz, J_2 = 1.2 Hz, 1H), 7.37 (d, J = 8.0 Hz, 1H), 7.32-7.25 (m, 5H), 7.09 (d, J = 8.0 Hz, 1H), 6.98-6.95 (m, 1H), 6.78 (s, 1H), 6.07 (s, 1H), 5.39 (d, J = 1.6 Hz, 1H), 5.12 (d, J = 1.6 Hz, 1H), 2.25 (s, 3H);

^{13}C NMR ($CDCl_3$, 100 MHz) δ 144.0, 141.0, 140.7, 140.2, 138.2, 132.7, 132.2, 131.3, 129.5, 128.8, 128.2, 127.5, 127.1, 123.7, 120.4, 88.6, 70.5, 21.6;

HRMS(ESI) Calculated for $C_{20}H_{18}O_2NS_2^+$ ($[M+H]^+$): 368.07735, found: 368.07718.

5-methyl-3-methylene-1-phenyl-2-(thiophen-2-ylsulfonyl)isoindoline (4c)

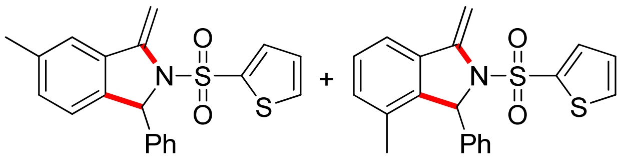

4c (major) 4c'

Following a general procedure: To a Schlenk tube was added $ZnBr_2$ (0.5 mmol, 112.5 mg), $MnBr(CO)_5$ (0.05 mmol, 10.0 mol%, 13.8 mg), DCM (5.0 mL), 1-*m*-tolylethanone **1x** (2.0 mmol, 268.0 mg), (*E*)-*N*-benzylidene -thiophene-2-sulfonamide **2a** (0.5 mmol, 125.5 mg) and Me_2Zn (0.75 mmol, 1.2 M in toluene, 0.625 mL) sequentially under nitrogen. The tube was sealed and stirred at 60 °C for 2 h. After completion, the reaction mixture was diluted with ethyl acetate (10 mL) and filtered through a short pad silica gel washing with ethyl acetate (20 mL). The filtrate was concentrated and subjected to crude ^{1}H NMR

analysis. The ratio of two regioisomers was obtained (**4c**:**4c'** = 3.9:1). The product was purified by silica gel column chromatography and obtained as a mixture in 53% yield.

^{1}H NMR ($CDCl_3$, 400 MHz) δ 7.45-7.43 (m, 2H), 7.28-7.26 (m, 6H), 7.03 (d, *J* = 7.6 Hz, 1H), 6.95-6.92 (m, 1H), 6.85 (d, *J* = 8.0 Hz, 1H), 6.07 (s, 1H), 5.42 (d, *J* = 2.0 Hz, 1H), 5.16 (d, *J* = 2.0 Hz, 1H), 2.31 (s, 3H);

^{13}C NMR ($CDCl_3$, 100 MHz) δ 144.0, 141.0, 138.3, 138.0, 137.9, 133.8, 132.6, 132.3, 131.0, 128.7, 128.1, 127.3, 127.1, 123.1, 120.8, 89.2, 70.4, 21.4;

HRMS(ESI) Calculated for $C_{20}H_{18}O_2NS_2^+$ ($[M+H]^+$): 368.07735, found: 368.07686.

4-methyl-3-methylene-1-phenyl-2-(thiophen-2-ylsulfonyl)isoindoline (4d)

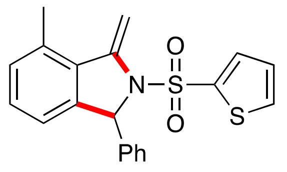

4d

Following a general procedure: To a Schlenk tube was added $ZnBr_2$ (0.5 mmol, 112.5 mg), $MnBr(CO)_5$ (0.05 mmol, 10.0 mol%, 13.8 mg), DCM (5.0 mL), 1-*o*-tolylethanone **1y** (2.0 mmol, 268.0 mg), (*E*)-*N*-benzylidenethiophene-2-sulfonamide **2a** (0.5 mmol, 125.5 mg) and Me_2Zn (0.75 mmol, 1.2 M in toluene, 0.625 mL) sequentially under nitrogen. The tube was sealed and stirred at 60 °C for 2 h. After completion, the reaction mixture was diluted with ethyl acetate (10 mL) and filtered through a short pad silica gel washing with ethyl acetate (20 mL). The filtrate was concentrated and purified by silica gel column chromatography to provide **4d** in 43% yield.

^{1}H NMR ($CDCl_3$, 400 MHz) δ 7.47-7.44 (m, 2H), 7.30-7.27 (m, 5H), 7.11 (t, *J* = 7.2 Hz, 1H), 7.06 (d, *J* = 7.2 Hz, 1H), 6.97-6.94 (m, 1H), 6.85 (d, *J* = 7.2 Hz, 1H), 6.11 (s, 1H), 5.66 (d, *J* = 2.0 Hz, 1H), 5.28 (d, *J* = 1.6 Hz, 1H), 2.51 (s, 3H);

^{13}C NMR ($CDCl_3$, 100 MHz) δ 145.1, 141.6, 141.4, 138.2, 134.1, 132.8, 132.3, 131.3, 131.0, 129.2, 128.8, 128.1, 127.4, 127.0, 121.2, 95.5, 69.9, 21.4;

HRMS(ESI) Calculated for $C_{20}H_{18}O_2NS_2^+$ ($[M+H]^+$): 368.07735, found: 368.07706.

(*E*)-1-ethylidene-3-phenyl-2-(thiophen-2-ylsulfonyl)isoindoline (4e)

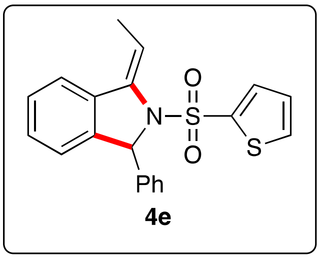

Following a general procedure: To a Schlenk tube was added $ZnBr_2$ (0.5 mmol, 112.5 mg), $MnBr(CO)_5$ (0.05 mmol, 10.0 mol%, 13.8 mg), DCM (5.0 mL), propiophenone **1z** (2.0 mmol, 268.0 mg), (*E*)-*N*-benzylidenethiophene-2-sulfonamide **2a** (0.5 mmol, 125.5 mg) and Me_2Zn (0.75 mmol, 1.2 M in toluene, 0.625 mL) sequentially under nitrogen. The tube was sealed and stirred at 60 °C for 2 h. After completion, the reaction mixture was diluted with ethyl acetate (10 mL) and filtered through a short pad silica gel washing with ethyl acetate (20 mL). The filtrate was concentrated and purified by silica gel column chromatography to provide **4e** in 42% yield.

^{1}H NMR ($CDCl_3$, 400 MHz) δ 7.62 (d, J = 7.6 Hz, 1H), 7.46 (d, J = 3.2 Hz, 1H), 7.41 (d, J = 4.4 Hz, 1H), 7.30-7.22 (m, 6H), 7.17 (t, J = 7.6 Hz, 1H), 7.03 (d, J = 7.6 Hz, 1H), 6.92 (t, J = 4.8 Hz, 1H), 6.29 (q, J = 7.6 Hz, 1H), 6.10 (s, 1H), 2.13 (d, J = 7.6 Hz, 3H);

^{13}C NMR ($CDCl_3$, 100 MHz) δ 141.4, 137.9, 137.7, 134.2, 132.5, 132.2, 128.8, 128.6, 128.1, 128.1, 127.3, 126.9, 124.0, 123.5, 110.3, 69.5, 13.3;

HRMS(ESI) Calculated for $C_{20}H_{18}O_2NS_2^+$ ($[M+H]^+$): 368.07735, found: 368.07725.

2-phenyl-1-(thiophen-2-ylsulfonyl)-1,2,6,7-tetrahydrobenzo[*cd*]indole (4f)

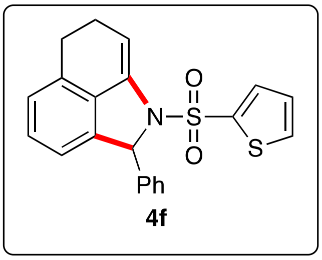

Following a general procedure: To a Schlenk tube was added $ZnBr_2$ (0.5 mmol, 112.5 mg), $MnBr(CO)_5$ (0.05 mmol, 10.0 mol%, 13.8 mg), DCM (5.0 mL), 3,4-dihydronaphthalen-1(2*H*)-one **1p** (2.0 mmol, 292.0 mg), (*E*)-*N*-benzylidenethiophene-2-sulfonamide **2a** (0.5 mmol, 125.5 mg) and Me_2Zn (1.0 mmol, 1.2 M in toluene, 0.83 mL) sequentially under nitrogen. The tube was sealed and stirred at 100 °C for 10 h. After completion, the reaction mixture was diluted with ethyl acetate (10 mL) and filtered through a short pad silica gel washing with ethyl acetate (20 mL). The filtrate was concentrated and purified by silica gel column chromatography to provide **4f** in 61% yield.

^{1}H NMR (CDCl$_3$, 400 MHz) δ 7.48-7.45 (m, 2H), 7.30-7.24 (m, 5H), 7.08 (t, *J* = 7.6 Hz, 1H), 7.02-7.96 (m, 2H), 6.75 (d, *J* = 7.2 Hz, 1H), 6.05 (s, 1H), 5.80 (t, *J* = 4.4 Hz, 1H), 2.95-2.79 (m, 2H), 2.69-2.61 (m, 2H);

^{13}C NMR (CDCl$_3$, 100 MHz) δ 140.5, 137.9, 137.0, 137.0, 133.0, 132.5, 132.3, 131.5, 129.6, 128.7, 128.2, 127.5, 127.1, 126.2, 120.6, 101.8, 72.0, 24.6, 23.6;

HRMS(ESI) Calculated for $C_{21}H_{18}O_2NS_2^+$ ([M+H]$^+$): 380.07735, found: 380.07660.

2-phenyl-1-(thiophen-2-ylsulfonyl)-2,6,7,8-tetrahydro-1*H*-cyclohepta[*cd*]isoindole (4g)

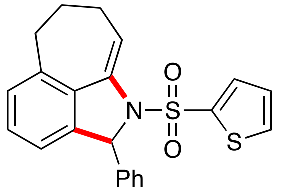

4g

Following a general procedure: To a Schlenk tube was added $ZnBr_2$ (0.5 mmol, 112.5 mg), $MnBr(CO)_5$ (0.05 mmol, 10.0 mol%, 13.8 mg), DCM (5.0 mL), 6,7,8,9-tetrahydro-5*H*-benzo[7]annulen-5-one **1A** (2.0 mmol, 320.0 mg), (*E*)-*N*-benzylidenethiophene-2-sulfonamide **2a** (0.5 mmol, 125.5 mg) and Me_2Zn (1.0 mmol, 1.2 M in toluene, 0.83 mL) sequentially under nitrogen. The tube was sealed and stirred at 100 °C for 10 h. After completion, the reaction mixture was diluted with ethyl acetate (10 mL) and filtered through a short pad silica gel washing with ethyl acetate (20 mL). The filtrate was concentrated and purified by silica gel column chromatography to provide **4g** in 47% yield.

^{1}H NMR (CDCl$_3$, 400 MHz) δ 7.48 (d, *J* = 2.8 Hz, 1H), 7.42 (d, *J* = 4.8 Hz, 1H), 7.33-7.23 (m, 5H), 7.03 (t, *J* = 7.2 Hz, 1H), 6.97-6.92 (m, 2H), 6.81 (d, *J* = 7.2 Hz, 1H), 6.42 (t, *J* = 4.8 Hz, 1H), 6.09 (s, 1H), 2.97-2.86 (m, 2H), 2.59 (d, *J* = 4.8 Hz, 2H), 1.95 (t, *J* = 4.8 Hz, 2H);

^{13}C NMR (CDCl$_3$, 100 MHz) δ 141.7, 141.1, 138.4, 138.1, 137.5, 132.5, 132.2, 128.7, 128.5, 128.5, 128.0, 127.2, 126.8, 120.8, 115.6, 69.6, 36.2, 30.2, 24.2;

HRMS(ESI) Calculated for $C_{22}H_{20}O_2NS_2^+$ ([M+H]$^+$): 394.09300, found: 394.09221.

1-methylene-2-(thiophen-2-ylsulfonyl)-3-p-tolylisoindoline (4h)

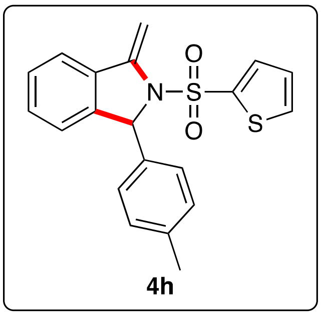

Following a general procedure: To a Schlenk tube was added $ZnBr_2$ (0.5 mmol, 112.5 mg), $MnBr(CO)_5$ (0.05 mmol, 10.0 mol%, 13.8 mg), DCM (5.0 mL), acetophenone **1v** (2.0 mmol, 240.0 mg), (*E*)-*N*-(4-methylbenzylidene)thiophene-2-sulfonamide **2b** (0.5 mmol, 132.5 mg) and Me_2Zn (0.75 mmol, 1.2 M in toluene, 0.625 mL) sequentially under nitrogen. The tube was sealed and stirred at 60 ^{o}C for 2 h. After completion, the reaction mixture was diluted with ethyl acetate (10 mL) and filtered through a short pad silica gel washing with ethyl acetate (20 mL). The filtrate was concentrated and purified by silica gel column chromatography to provide **4h** in 53% yield.

^{1}H NMR ($CDCl_3$, 500 MHz) δ 7.49-7.45 (m, 3H), 7.29-7.20 (m, 2H), 7.15 (d, *J* = 8.0 Hz, 2H), 7.10 (d, *J* = 8.0 Hz, 2H), 6.99-6.95 (m, 2H), 6.09 (s, 1H), 5.43 (s, 1H), 5.18 (s, 1H), 2.32 (s, 3H);

^{13}C NMR ($CDCl_3$, 125 MHz) δ 143.9, 140.7, 138.2, 138.0, 138.0, 133.8, 132.7, 132.3, 129.8, 129.4, 128.3, 127.4, 127.1, 123.5, 120.6, 89.4, 70.5, 21.3;

HRMS(ESI) Calculated for $C_{20}H_{18}O_2NS_2^+$ ($[M+H]^+$): 368.07735, found: 368.07729.

1-(4-methoxyphenyl)-3-methylene-2-(thiophen-2-ylsulfonyl)isoindoline (4i)

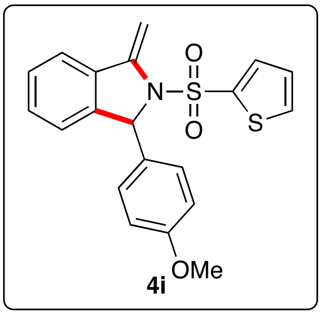

Following a general procedure: To a Schlenk tube was added $ZnBr_2$ (0.5 mmol, 112.5 mg), $MnBr(CO)_5$ (0.05 mmol, 10.0 mol%, 13.8 mg), DCM (5.0 mL), acetophenone **1v** (2.0 mmol, 240.0 mg), (*E*)-*N*-(4-methoxybenzylidene)thiophene-2-sulfonamide **2c** (0.5 mmol, 140.5 mg) and Me_2Zn (0.75 mmol, 1.2 M in toluene, 0.625 mL) sequentially under nitrogen. The tube was sealed and stirred at 60 ^{o}C for 2 h. After completion, the reaction mixture was diluted with ethyl acetate (10 mL) and filtered through a short pad silica gel washing with ethyl acetate (20 mL). The filtrate was concentrated and purified by silica gel column chromatography to

provide **4i** in 43% yield.

^{1}H NMR (CDCl$_3$, 400 MHz) δ 7.50-7.42 (m, 3H), 7.30-7.21 (m, 2H), 7.16 (d, J = 8.8 Hz, 2H), 6.98-6.94 (m, 2H), 6.81 (d, J = 8.4 Hz, 2H), 6.11 (s, 1H), 5.41 (d, J = 1.6 Hz, 1H), 5.17 (d, J = 1.6 Hz, 1H), 3.77 (s, 3H);

^{13}C NMR (CDCl$_3$, 100 MHz) δ 159.6, 143.8, 140.8, 138.4, 133.9, 133.0, 132.6, 132.2, 129.8, 129.0, 128.3, 127.0, 123.5, 120.6, 114.1, 89.3, 70.2, 55.4;

HRMS(ESI) Calculated for $C_{20}H_{18}O_3NS_2^+$ ([M+H]$^+$): 384.07226, found: 384.07177.

1-(4-fluorophenyl)-3-methylene-2-(thiophen-2-ylsulfonyl)isoindoline (4j)

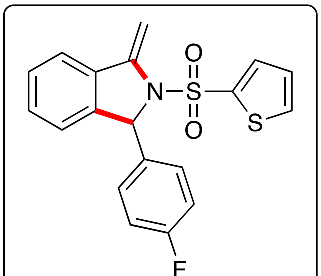

4j

Following a general procedure: To a Schlenk tube was added ZnBr$_2$ (0.5 mmol, 112.5 mg), MnBr(CO)$_5$ (0.05 mmol, 10.0 mol%, 13.8 mg), DCM (5.0 mL), acetophenone **1v** (2.0 mmol, 240.0 mg), (*E*)-*N*-(4-fluorobenzylidene)thiophene-2-sulfonamide **2d** (0.5 mmol, 134.5 mg) and Me$_2$Zn (0.75 mmol, 1.2 M in toluene, 0.625 mL) sequentially under nitrogen. The tube was sealed and stirred at 60 ^{o}C for 2 h. After completion, the reaction mixture was diluted with ethyl acetate (10 mL) and filtered through a short pad silica gel washing with ethyl acetate (20 mL). The filtrate was concentrated and purified by silica gel column chromatography to provide **4j** in 67% yield.

^{1}H NMR (CDCl$_3$, 400 MHz) δ 7.50-7.47 (m, 3H), 7.29 (t, J = 7.2 Hz, 1H), 7.26-7.21 (m, 3H), 7.00-6.94 (m, 4H), 6.11 (s, 1H), 5.44 (d, J = 2.0 Hz, 1H), 5.19 (d, J = 2.0 Hz, 1H);

^{13}C NMR (CDCl$_3$, 100 MHz) δ 162.6 (d, $^1J_{C\text{-}F}$ = 245.1 Hz), 143.7, 140.2, 138.0, 136.7 (d, $^4J_{C\text{-}F}$ = 3.3 Hz), 133.8, 132.8, 132.5, 129.9, 129.3 (d, $^3J_{C\text{-}F}$ = 8.2 Hz), 128.6, 127.1, 123.4, 120.7, 115.6 (d, $^2J_{C\text{-}F}$ = 21.7 Hz), 89.7, 69.8;

^{19}F NMR (CDCl$_3$, 565 MHz) δ -113.8;

HRMS(ESI) Calculated for $C_{19}H_{15}O_2NFS_2^+$ ([M+H]$^+$): 372.05227, found: 372.05198.

1-(4-bromophenyl)-3-methylene-2-(thiophen-2-ylsulfonyl)isoindoline (4k)

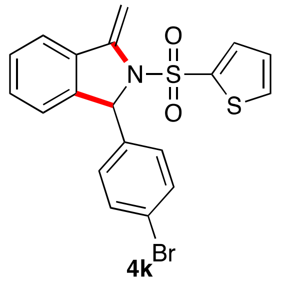

4k

Following a general procedure: To a Schlenk tube was added $ZnBr_2$ (0.5 mmol, 112.5 mg), $MnBr(CO)_5$ (0.05 mmol, 10.0 mol%, 13.8 mg), DCM (5.0 mL), acetophenone **1v** (2.0 mmol, 240.0 mg), (*E*)-*N*-(4-bromobenzylidene)thiophene-2-sulfonamide **2f** (0.5 mmol, 164.5 mg) and Me_2Zn (0.75 mmol, 1.2 M in toluene, 0.625 mL) sequentially under nitrogen. The tube was sealed and stirred at 60 °C for 2 h. After completion, the reaction mixture was diluted with ethyl acetate (10 mL) and filtered through a short pad silica gel washing with ethyl acetate (20 mL). The filtrate was concentrated and purified by silica gel column chromatography to provide **4k** in 42% yield.

^{1}H NMR (CDCl$_3$, 400 MHz) δ 7.52-7.47 (m, 3H), 7.42 (d, *J* = 7.6 Hz, 2H), 7.30 (t, *J* = 7.2 Hz, 1H), 7.24 (t, *J* = 7.2 Hz, 1H), 7.16 (d, *J* = 8.4 Hz, 2H), 7.00 (t, *J* = 4.4 Hz, 1H), 6.96 (d, *J* = 7.6 Hz, 1H), 6.07 (s, 1H), 5.46 (d, *J* = 1.6 Hz, 1H), 5.20 (d, *J* = 1.6 Hz, 1H);

^{13}C NMR (CDCl$_3$, 100 MHz) δ 143.7, 140.0, 139.9, 137.7, 133.8, 132.9, 132.6, 131.9, 130.0, 129.2, 128.7, 127.2, 123.4, 122.3, 120.8, 89.8, 69.9;

HRMS(ESI) Calculated for $C_{19}H_{15}O_2NBrS_2^+$ ($[M+H]^+$): 431.97221, found: 431.97250.

1-methylene-2-(thiophen-2-ylsulfonyl)-3-m-tolylisoindoline (4l)

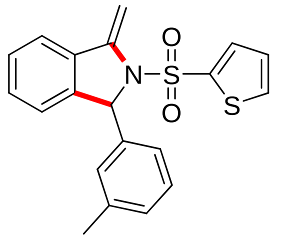

4l

Following a general procedure: To a Schlenk tube was added $ZnBr_2$ (0.5 mmol, 112.5 mg), $MnBr(CO)_5$ (0.05 mmol, 10.0 mol%, 13.8 mg), DCM (5.0 mL), acetophenone **1v** (2.0 mmol, 240.0 mg), (*E*)-*N*-(3-methylbenzylidene)thiophene-2-sulfonamide **2i** (0.5 mmol, 132.5 mg) and Me_2Zn (0.75 mmol, 1.2 M in toluene, 0.625 mL) sequentially under nitrogen. The tube was sealed and stirred at 60 °C for 2 h. After

completion, the reaction mixture was diluted with ethyl acetate (10 mL) and filtered through a short pad silica gel washing with ethyl acetate (20 mL). The filtrate was concentrated and purified by silica gel column chromatography to provide **4l** in 62% yield.

^{1}H NMR ($CDCl_3$, 400 MHz) δ 7.50-7.45 (m, 2H), 7.44 (d, *J* = 3.6 Hz, 1H), 7.27 (t, *J* = 7.2 Hz, 1H), 7.23 (d, *J* = 8.0 Hz, 1H), 7.18 (t, *J* = 7.2 Hz, 1H), 7.07 (t, *J* = 6.8 Hz, 2H), 7.03 (s, 1H), 6.98 (d, *J* = 7.6 Hz, 1H), 6.95 (t, *J* = 4.4 Hz, 1H), 6.08 (s, 1H), 5.45 (d, *J* = 1.6 Hz, 1H), 5.19 (d, *J* = 1.6 Hz, 1H), 2.27 (s, 3H);

^{13}C NMR ($CDCl_3$, 100 MHz) δ 143.9, 140.7, 140.6, 138.4, 138.0, 133.7, 132.7, 132.3, 129.8, 129.0, 128.6, 128.3, 127.9, 127.0, 124.5, 123.4, 120.6, 89.3, 70.6, 21.5;

HRMS(ESI) Calculated for $C_{20}H_{18}O_2NS_2^+$ ($[M+H]^+$): 368.07735, found: 368.07710.

1-methylene-3-phenyl-2-tosylisoindoline (4m)

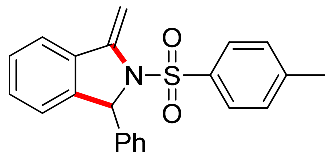

4m

Following a general procedure: To a Schlenk tube was added $ZnBr_2$ (0.5 mmol, 112.5 mg), $MnBr(CO)_5$ (0.05 mmol, 10.0 mol%, 13.8 mg), DCM (5.0 mL), acetophenone **1v** (2.0 mmol, 240.0 mg), (*E*)-*N*-benzylidene-4-methylbenzenesulfonamide **2m** (0.5 mmol, 129.5 mg) and Me_2Zn (0.75 mmol, 1.2 M in toluene, 0.625 mL) sequentially under nitrogen. The tube was sealed and stirred at 60 °C for 2 h. After completion, the reaction mixture was diluted with ethyl acetate (10 mL) and filtered through a short pad silica gel washing with ethyl acetate (20 mL). The filtrate was concentrated and purified by silica gel column chromatography to provide **4m** in 65% yield.

^{1}H NMR ($CDCl_3$, 400 MHz) δ 7.56 (d, *J* = 8.4 Hz, 2H), 7.45 (d, *J* = 7.2 Hz, 1H), 7.30-7.17 (m, 7H), 7.14 (d, *J* = 8.0 Hz, 2H), 6.96 (d, *J* = 7.6 Hz, 1H), 6.17 (s, 1H), 5.33 (s, 1H), 5.06 (d, *J* = 1.6 Hz, 1H), 2.32 (s, 3H);

^{13}C NMR ($CDCl_3$, 100 MHz) δ 144.2, 143.9, 141.2, 140.7, 135.8, 134.0, 129.7, 129.5, 128.7, 128.3, 128.1, 127.5, 127.5, 123.4, 120.5, 88.1, 70.4, 21.6;

HRMS(ESI) Calculated for $C_{22}H_{19}O_2NNaS^+$ ($[M+Na]^+$): 384.10287, found: 384.10236.

1-ethyl-1-methyl-3-phenyl-2-(thiophen-2-ylsulfonyl)isoindoline (5a)

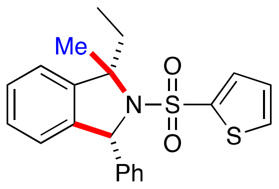

5a

Following a general procedure: To a Schlenk tube was added $ZnBr_2$ (0.5 mmol, 112.5 mg), $MnBr(CO)_5$ (0.05 mmol, 10.0 mol%, 13.8 mg), DCM (5.0 mL), propiophenone **1z** (2.0 mmol, 276.0 mg), (*E*)-*N*-benzylidenethiophene-2-sulfonamide **2a** (0.5 mmol, 125.5 mg) and Me_2Zn (1.0 mmol, 1.2 M in toluene, 0.83 mL) sequentially under nitrogen. The tube was sealed and stirred at 100 °C for 10 h. After completion, the reaction mixture was diluted with ethyl acetate (10 mL) and filtered through a short pad silica gel washing with ethyl acetate (20 mL). The filtrate was concentrated and purified by silica gel column chromatography to provide **5a** in 63% yield (*dr* = 3.1 :1).

^{1}H NMR (CDCl$_3$, 400 MHz) δ 7.33 (dd, J_1 = 5.2 Hz, J_2 = 1.2 Hz, 1H), 7.28 (t, J = 7.6 Hz, 1H), 7.24-7.19 (m, 5H), 7.14 (t, J = 7.6 Hz, 1H), 7.09 (d, J = 7.6 Hz, 1H), 7.01 (dd, J_1 = 4.0 Hz, J_2 = 1.2 Hz, 1H), 6.75-6.70 (m, 2H), 5.93 (s, 1H), 2.77-2.67 (m, 1H), 2.16-2.05 (m, 1H), 1.83 (s, 3H), 0.93 (t, J = 7.6 Hz, 3H);

^{13}C NMR (CDCl$_3$, 100 MHz) δ 143.7, 143.0, 140.9, 139.8, 132.7, 131.3, 129.3, 128.3, 128.3, 128.1, 128.0, 126.5, 123.7, 121.1, 76.4, 68.9, 33.1, 29.9, 10.1;

HRMS(ESI) Calculated for $C_{21}H_{21}O_2NNaS_2^+$ ($[M+Na]^+$): 406.09059, found: 406.09025.

1-isopropyl-1-methyl-3-phenyl-2-(thiophen-2-ylsulfonyl)isoindoline (5b)

Following a general procedure: To a Schlenk tube was added $ZnBr_2$ (0.5 mmol, 112.5 mg), $MnBr(CO)_5$ (0.05 mmol, 10.0 mol%, 13.8 mg), DCM (5.0 mL), 2-methyl-1-phenylpropan-1-one **1B** (2.0 mmol, 296.0 mg), (*E*)-*N*-benzylidenethiophene-2-sulfonamide **2a** (0.5 mmol, 125.5 mg) and Me_2Zn (1.0

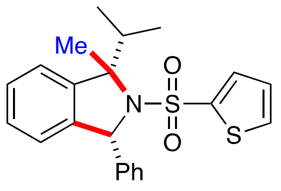

5b

mmol, 1.2 M in toluene, 0.83 mL) sequentially under nitrogen. The tube was sealed and stirred at 100 °C for 10 h. After completion, the reaction mixture was diluted with ethyl acetate (10 mL) and filtered through a short pad silica gel washing with ethyl acetate (20 mL). The filtrate was concentrated and purified by silica gel column chromatography to provide **5b** in 53% yield (*dr* = 9:1).

^{1}H NMR (CDCl$_3$, 400 MHz) δ 7.32-7.15 (m, 9H), 6.88 (dd, J_1 = 3.6 Hz, J_2 = 1.2 Hz, 1H), 6.76 (d, J = 7.6 Hz, 1H), 6.72-6.68 (m, 1H), 6.00 (s, 1H), 3.02-2.94 (m, 1H), 1.94 (s, 3H), 1.33 (d, J = 7.2 Hz, 3H), 0.92 (d, J = 6.8 Hz, 3H);

^{13}C NMR (CDCl$_3$, 100 MHz) δ 143.7, 143.3, 140.4, 139.7, 132.3, 131.1, 130.0, 128.2 (overlap), 128.0, 127.9, 126.4, 123.8, 122.5, 79.7, 69.0, 36.6, 28.0, 19.6, 18.9;

HRMS(ESI) Calculated for $C_{22}H_{23}O_2NNaS_2^+$ ([M+Na]$^+$): 420.10624, found: 420.10586.

1-cyclopropyl-1-methyl-3-phenyl-2-(thiophen-2-ylsulfonyl)isoindoline (5c)

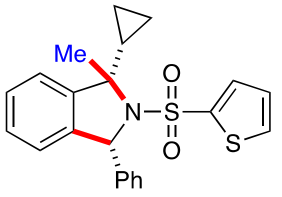

5c

Following a general procedure: To a Schlenk tube was added $ZnBr_2$ (0.5 mmol, 112.5 mg), MnBr(CO)$_5$ (0.05 mmol, 10.0 mol%, 13.8 mg), DCM (5.0 mL), cyclopropyl(phenyl)methanone **1n** (2.0 mmol, 292.0 mg), (*E*)-*N*-benzylidenethiophene-2-sulfonamide **2a** (0.5 mmol, 125.5 mg) and Me_2Zn (1.0 mmol, 1.2 M in toluene, 0.83 mL) sequentially under nitrogen. The tube was sealed and stirred at 100 °C for 10 h. After completion, the reaction mixture was diluted with ethyl acetate (10 mL) and filtered through a short pad silica gel washing with ethyl acetate (20 mL). The filtrate was concentrated and purified by silica gel column chromatography to provide **5c** in 44 % yield (*dr* = 3.3:1).

^{1}H NMR (CDCl$_3$, 400 MHz) δ 7.33 (dd, J_1 = 5.2 Hz, J_2 = 1.2 Hz, 1H), 7.29-7.25 (m, 3H), 7.21-7.15 (m, 5H), 7.05 (dd, J_1 = 4.0 Hz, J_2 = 1.2 Hz, 1H), 6.84 (d, J = 7.6 Hz, 1H), 6.73 (t, J = 4.0 Hz, 1H), 6.01 (s, 1H), 1.81-1.73 (m, 4H), 1.02-0.95 (m, 1H),

0.80-0.72 (m, 1H), 0.70-0.62 (m, 1H), 0.47-0.39 (m, 1H);

^{13}C NMR ($CDCl_3$, 100 MHz) δ 144.0, 143.8, 141.3, 139.2, 132.4, 130.9, 128.6, 128.3, 128.0, 127.8, 126.4, 123.7, 122.2, 75.0, 69.6, 25.1, 22.9, 3.7, 3.3;

HRMS(ESI) Calculated for $C_{22}H_{21}O_2NNaS_2^+$ ($[M+Na]^+$): 418.09059, found: 418.08978.

1-cyclohexyl-1-methyl-3-phenyl-2-(thiophen-2-ylsulfonyl)isoindoline (5d)

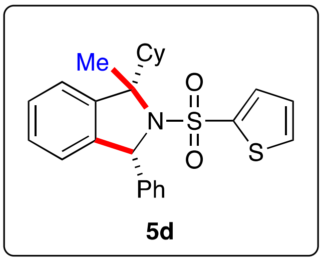

Following a general procedure: To a Schlenk tube was added $ZnBr_2$ (0.5 mmol, 112.5 mg), $MnBr(CO)_5$ (0.05 mmol, 10.0 mol%, 13.8 mg), DCM (5.0 mL), cyclohexyl(phenyl)methanone **1o** (2.0 mmol, 376.0 mg), (*E*)-*N*-benzylidenethiophene-2-sulfonamide **2a** (0.5 mmol, 125.5 mg) and Me_2Zn (1.0 mmol, 1.2 M in toluene, 0.83 mL) sequentially under nitrogen. The tube was sealed and stirred at 100 °C for 10 h. After completion, the reaction mixture was diluted with ethyl acetate (10 mL) and filtered through a short pad silica gel washing with ethyl acetate (20 mL). The filtrate was concentrated and purified by silica gel column chromatography to provide **5d** in 65 % yield (*dr* = 7.6:1).

^{1}H NMR ($CDCl_3$, 400 MHz) δ 7.30-7.14 (m, 9H), 6.86 (dd, J_1 = 3.6 Hz, J_2 = 1.2 Hz, 1H), 6.75 (d, J = 7.6 Hz, 1H), 6.69 (t, J = 4.4 Hz, 1H), 6.03 (s, 1H), 2.55-2.48 (m, 1H), 2.18 (d, J = 12.0 Hz, 1H), 1.92-1.85 (m, 5H), 1.68-1.64 (m, 2H), 1.54 (dq, J_1 = 12.0 Hz, J_2 = 3.2 Hz, 1H), 1.38-1.27 (m, 1H), 1.21-1.08(m, 2H), 0.82 (dq, J_1 = 12.4 Hz, J_2 = 3.2 Hz, 1H);

^{13}C NMR ($CDCl_3$, 100 MHz) δ 144.0, 143.9, 140.3, 139.5, 132.2, 131.0, 130.2, 128.2, 128.2, 127.9, 127.8, 126.3, 123.8, 122.7, 79.6, 68.9, 46.6, 29.3, 28.9, 27.9, 27.4, 26.5;

HRMS(ESI) Calculated for $C_{25}H_{27}O_2NNaS_2^+$ ($[M+Na]^+$): 460.13754, found: 460.13663.

1-isopropyl-1-methyl-3-phenyl-2-tosylisoindoline (5e)

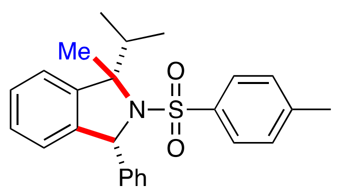

5e

Following a general procedure: To a Schlenk tube was added $ZnBr_2$ (0.5 mmol, 112.5 mg), $MnBr(CO)_5$ (0.05 mmol, 10.0 mol%, 13.8 mg), DCM (5.0 mL), cyclohexyl(phenyl)methanone **1B** (2.0 mmol, 296.0 mg), *N*-benzylidene-4-methylbenzenesulfonamide **2m** (0.5 mmol, 129.5 mg) and Me_2Zn (1.0 mmol, 1.2 M in toluene, 0.83 mL) sequentially under nitrogen. The tube was sealed and stirred at 100 °C for 10 h. After completion, the reaction mixture was diluted with ethyl acetate (10 mL) and filtered through a short pad silica gel washing with ethyl acetate (20 mL). The filtrate was concentrated and purified by silica gel column chromatography to provide **5e** in 41% yield (*dr* = 16:1).

^{1}H NMR (CDCl$_3$, 400 MHz) δ 7.28 (d, *J* = 7.6 Hz, 1H), 7.25-7.08 (m, 9H), 6.91 (d, *J* = 8.0 Hz, 2H), 6.68 (d, *J* = 7.2 Hz, 1H), 5.93 (s, 1H), 3.11-3.03 (m, 1H), 2.25 (s, 3H), 1.96 (s, 3H), 1.35 (d, *J* = 6.8 Hz, 3H), 0.90 (d, *J* = 6.8 Hz, 3H);

^{13}C NMR (CDCl$_3$, 100 MHz) δ 143.3, 142.2, 140.4, 140.1, 139.1, 129.7, 128.7, 128.1, 127.8, 127.8, 127.7, 127.5, 123.7, 122.3, 79.2, 68.7, 36.5, 28.2, 21.4, 19.5, 18.9;

HRMS(ESI) Calculated for $C_{25}H_{27}O_2NNaS^+$ ([M+Na]$^+$): 428.16547, found: 428.16449.

1-cyclohexyl-3-(4-methoxyphenyl)-1-methyl-2-(thiophen-2-ylsulfonyl)isoindoline (5f)

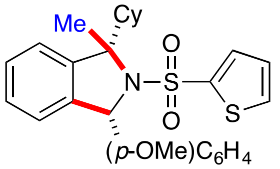

5f

Following a general procedure: To a Schlenk tube was added $ZnBr_2$ (0.5 mmol, 112.5 mg), $MnBr(CO)_5$ (0.05 mmol, 10.0 mol%, 13.8 mg), DCM (5.0 mL), cyclohexyl(phenyl)methanone **1o** (2.0 mmol, 376.0 mg), (*E*)-N-(4-methoxybenzylidene)thiophene-2-sulfonamide **2c** (0.5 mmol, 140.5 mg) and Me_2Zn (1.0 mmol, 1.2 M in toluene, 0.83 mL) sequentially under nitrogen. The tube was sealed and stirred at 100 °C for 10 h. After completion, the reaction mixture was diluted with ethyl acetate (10 mL) and filtered through a

short pad silica gel washing with ethyl acetate (20 mL). The filtrate was concentrated and purified by silica gel column chromatography to provide **5f** in 57% yield (*dr* = 5.8:1).

^{1}H NMR ($CDCl_3$, 400 MHz) δ 7.31 (dd, J_1 = 4.8 Hz, J_2 = 1.2 Hz, 1H), 7.28-7.23 (m, 2H), 7.21-7.14 (m, 3H), 6.91 (dd, J_1 = 4.0 Hz, J_2 = 1.2 Hz, 1H), 6.77-6.71 (m, 4H), 6.01 (s, 1H), 3.79 (s, 3H), 2.54-2.46 (m, 1H), 2.16 (d, J = 12.0 Hz, 1H), 1.90 (s, 3H), 1.90-1.80 (m, 2H), 1.67-1.61 (m, 2H), 1.52 (dq, J_1 = 12.4 Hz, J_2 = 3.2 Hz, 1H), 1.37-1.25 (m, 1H), 1.21-1.02 (m, 2H), 0.78 (dq, J_1 = 12.4 Hz, J_2 = 3.2 Hz, 1H);

^{13}C NMR ($CDCl_3$, 125 MHz) δ 159.6, 144.2, 143.8, 139.6, 132.4, 132.0, 131.5, 131.0, 127.8, 127.7, 126.3, 123.8, 122.6, 113.5, 79.3, 68.4, 55.4, 46.5, 29.3, 28.8, 28.0, 27.3, 27.0, 26.4;

HRMS(ESI) Calculated for $C_{26}H_{29}O_3NNaS_2^+$ ($[M+Na]^+$): 490.14811, found: 490.14749.

1-cyclohexyl-3-(4-fluorophenyl)-1-methyl-2-(thiophen-2-ylsulfonyl)isoindoline (5g)

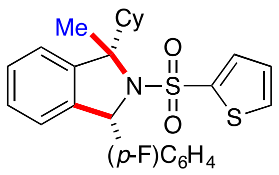

5g

Following a general procedure: To a Schlenk tube was added $ZnBr_2$ (0.5 mmol, 112.5 mg), $MnBr(CO)_5$ (0.05 mmol, 10.0 mol%, 13.8 mg), DCM (5.0 mL), cyclohexyl(phenyl)methanone **1o** (2.0 mmol, 296.0 mg), (*E*)-N-(4-fluorobenzylidene)thiophene-2-sulfonamide **2d** (0.5 mmol, 129.5 mg) and Me_2Zn (1.0 mmol, 1.2 M in toluene, 0.83 mL) sequentially under nitrogen. The tube was sealed and stirred at 100 °C for 10 h. After completion, the reaction mixture was diluted with ethyl acetate (10 mL) and filtered through a short pad silica gel washing with ethyl acetate (20 mL). The filtrate was concentrated and purified by silica gel column chromatography to provide **5g** in 41% yield (*dr* = 9.8:1).

^{1}H NMR ($CDCl_3$, 500 MHz) δ 7.33 (dd, J_1 = 5.0 Hz, J_2 = 1.0 Hz, 1H), 7.31-7.24 (m, 4H), 7.18 (dt, J_1 = 7.5 Hz, J_2 = 1.5 Hz, 1H), 6.97 (dd, J_1 = 4.0 Hz, J_2 = 1.5 Hz, 1H),

6.90 (t, J = 8.5 Hz, 2H), 6.77-6.72 (m, 2H), 6.02 (s, 1H), 2.50 (m, 1H), 2.17 (m, 1H), 1.90 (s, 3H), 1.90-1.81 (m, 2H), 1.68-1.64 (m, 2H), 1.53 (dq, J_1 = 12.5 Hz, J_2 = 3.5 Hz, 1H), 1.32 (tq, J_1 = 12.5 Hz, J_2 = 3.5 Hz, 1H), 1.24-1.05 (m, 2H), 0.81 (dq, J_1 = 12.5 Hz, J_2 = 3.5 Hz, 1H);

^{13}C NMR ($CDCl_3$, 125 MHz) δ 162.7 (d, $^1J_{C-F}$ = 245.6 Hz), 144.0, 143.8, 139.2, 136.3 (d, $^4J_{C-F}$ = 3.4 Hz), 132.2, 131.9 (d, $^3J_{C-F}$ = 8.3 Hz), 131.3, 128.1, 127.9, 126.3, 123.7, 122.8, 115.1 (d, $^2J_{C-F}$ = 21.3 Hz), 79.7, 68.1, 46.6, 29.4, 28.8, 27.9, 27.3, 26.5, 26.5;

^{19}F NMR ($CDCl_3$, 565 MHz) δ -117.4;

HRMS(ESI) Calculated for $C_{25}H_{27}O_2NFS_2^+$ ($[M+H]^+$): 456.14618, found: 456.14593.

Supplementary Discussion

Explorations on possible reaction intermediates

To probe the possible reaction mechanism, a series of stoichiometric reactions were carried out. We investigated the reaction of 2,2-dimethyl-1-phenylpropan-1-one **1a** with a stoichiometric amount of $MnBr(CO)_5$ in the presence of Me_2Zn at 60 °C in DCE. A five-membered manganacycle **Mn-I** was isolated in 28% yield. In contrast, no reaction occurred in the absence of Me_2Zn or in the presence of $ZnBr_2$. We proposed that Me_2Zn first reacted with $MnBr(CO)_5$ to give $MnMe(CO)_5$, which promoted the formation of five-member manganacycle **Mn-I** via the C-H bond activation.[22]

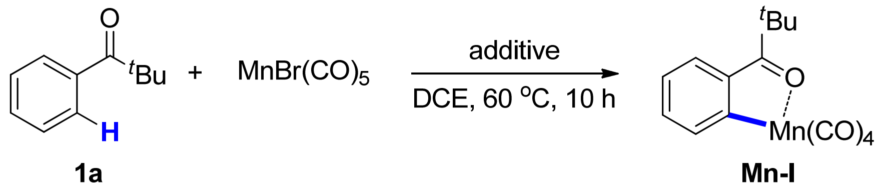

additive	isolated yield
-	n.r.
$ZnBr_2$ (1.0 equiv.)	n.r.
Me_2Zn (1.5 equiv.)	28%

Supplementary Figure 9. Preparation of manganacycle Mn-I

Experimental procedure:

MnBr(CO)$_5$ (0.2 mmol, 55 mg) was added into an oven-dried reaction vessel with Teflon screw cap under a nitrogen atmosphere. DCE (5.0 mL), 2,2-dimethyl-1-phenylpropan-1-one **1a** (0.8 mmol, 129.6 mg) and Me$_2$Zn (0.3 mmol, 1.2 M in toluene) were then injected into the reaction tube. The reaction mixture was stirred at 60 ^{o}C for 10 h. After the completion, the mixture was diluted with ethyl acetate (20 mL) and filtered through a short pad of silica gel. The solvent was removed by rotary evaporation and the residue was purified by silica gel column chromatography to afford the five-membered manganacycle **Mn-I** in 28% yield.

^{1}H NMR (CDCl$_3$, 400 MHz) δ 8.16 (d, J = 6.8 Hz, 1H), 8.09 (d, J = 6.4 Hz, 1H), 7.35 (s, 1H), 7.13 (s, 1H), 1.41 (s, 9H);

^{13}C NMR (CDCl$_3$, 100 MHz) δ 213.2, 211.8, 194.8, 143.2, 142.0, 132.8, 132.4, 123.2, 44.6, 28.8;

HRMS(EI) Calculated for C$_{15}$H$_{13}$O$_5$Mn: 328.0143, found: 328.0141.

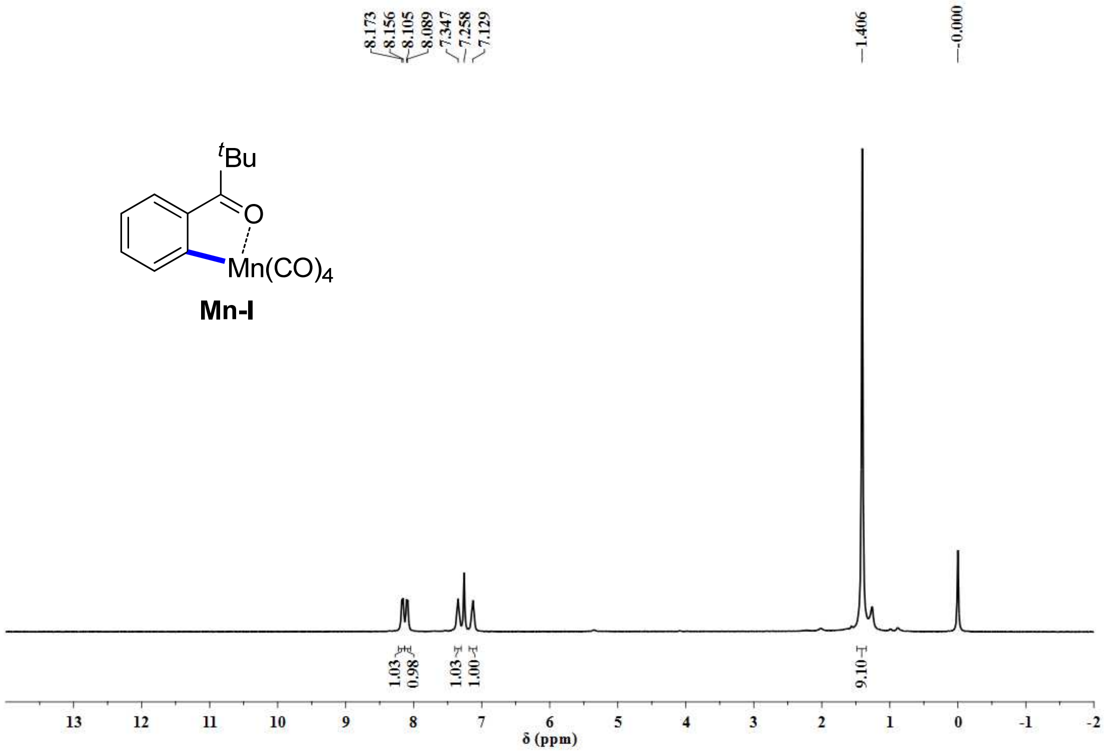

Supplementary Figure 10. ^{1}H NMR spectrum of Mn-I

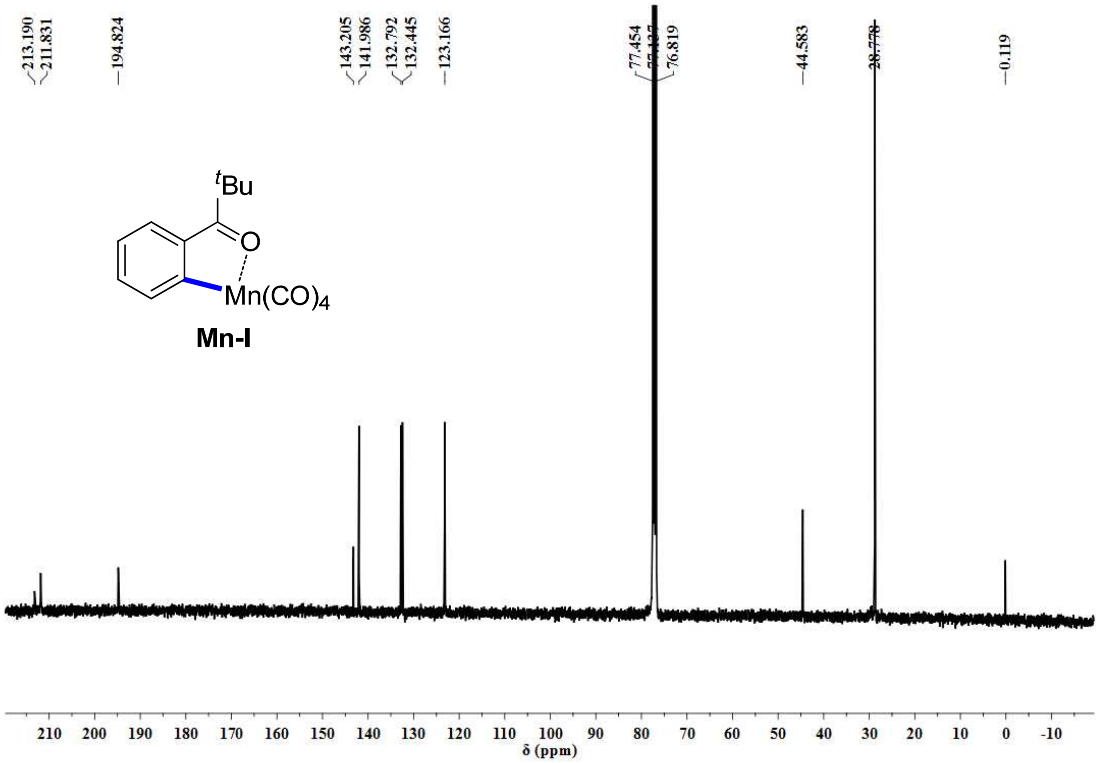

Supplementary Figure 11. ^{13}C NMR spectrum of Mn-I

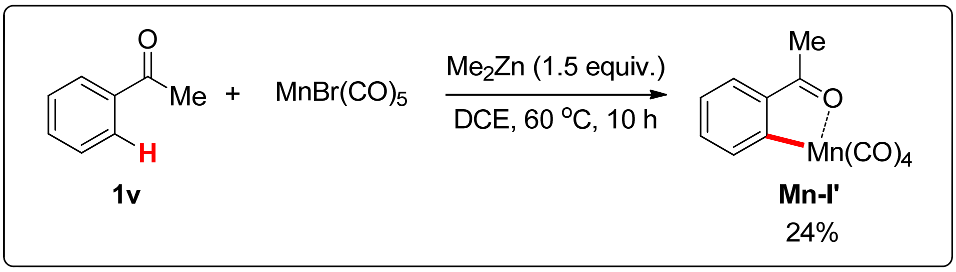

Supplementary Figure 12. Preparation of manganacycle Mn-I'

Experimental procedure:

$MnBr(CO)_5$ (0.2 mmol, 55 mg) was added into an oven-dried reaction vessel with Teflon screw cap under a nitrogen atmosphere. DCE (0.5 mL), acetophenone **1v** (0.2 mmol, 24 mg) and Me_2Zn (0.3 mmol, 1.2 M in toluene) were then injected into the reaction tube. The reaction mixture was stirred at 60 °C for 6 h. After the completion, the mixture was diluted with ethyl acetate (20 mL) and filtered through a short pad of silica gel. The solvent was removed by rotary evaporation and the residue was purified by silica gel column chromatography to afford the five-membered manganacycle **Mn-I'** in 24% yield.

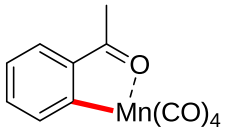

Mn-I′

^{1}H NMR ($CDCl_3$, 400 MHz) δ 8.10 (d, J = 7.2 Hz, 1H), 7.85 (dd, J_1 = 7.6 Hz, J_2 = 0.8 Hz, 1H), 7.43 (dt, J_1 = 7.6 Hz, J_2 = 1.2 Hz, 1H), 7.18 (dt, J_1 = 7.6 Hz, J_2 = 1.2 Hz, 1H), 2.62 (s, 3H);

^{13}C NMR ($CDCl_3$, 100 MHz) δ 216.7, 193.5, 145.4, 141.6, 134.0, 131.7, 124.0, 24.7;

HRMS(EI) Calculated for $C_{12}H_7O_5Mn$: 285.9674, found: 285.9678.

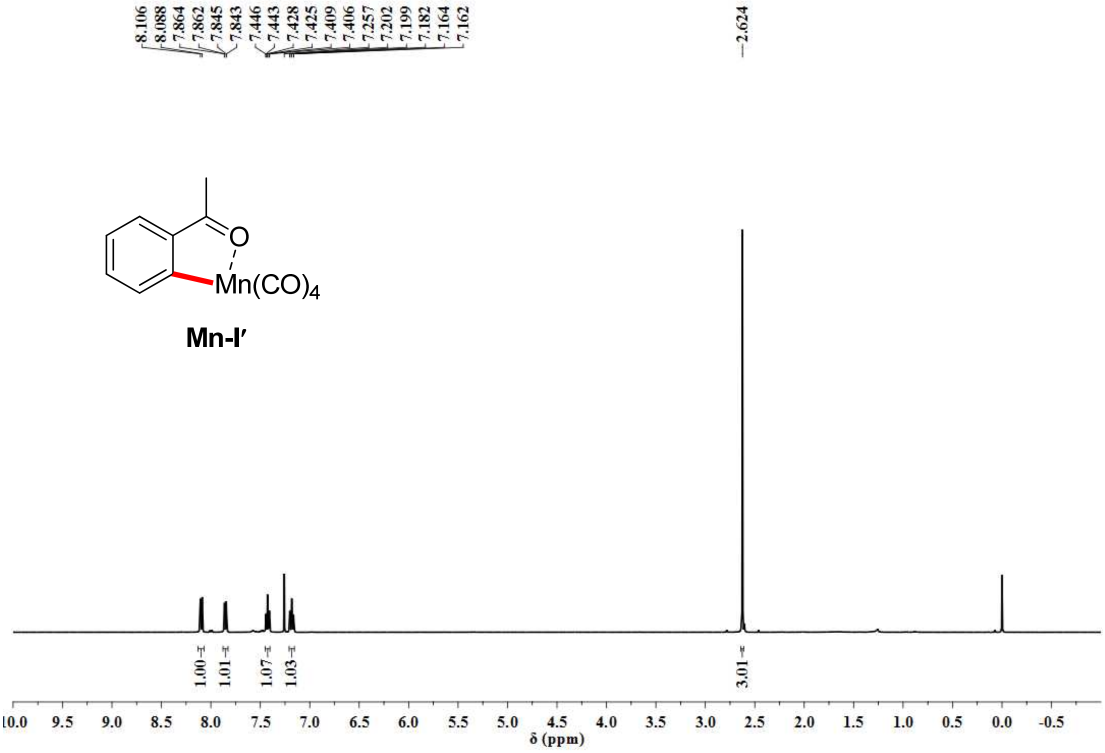

Supplementary Figure 13. ^{1}H NMR spectrum of Mn-I’

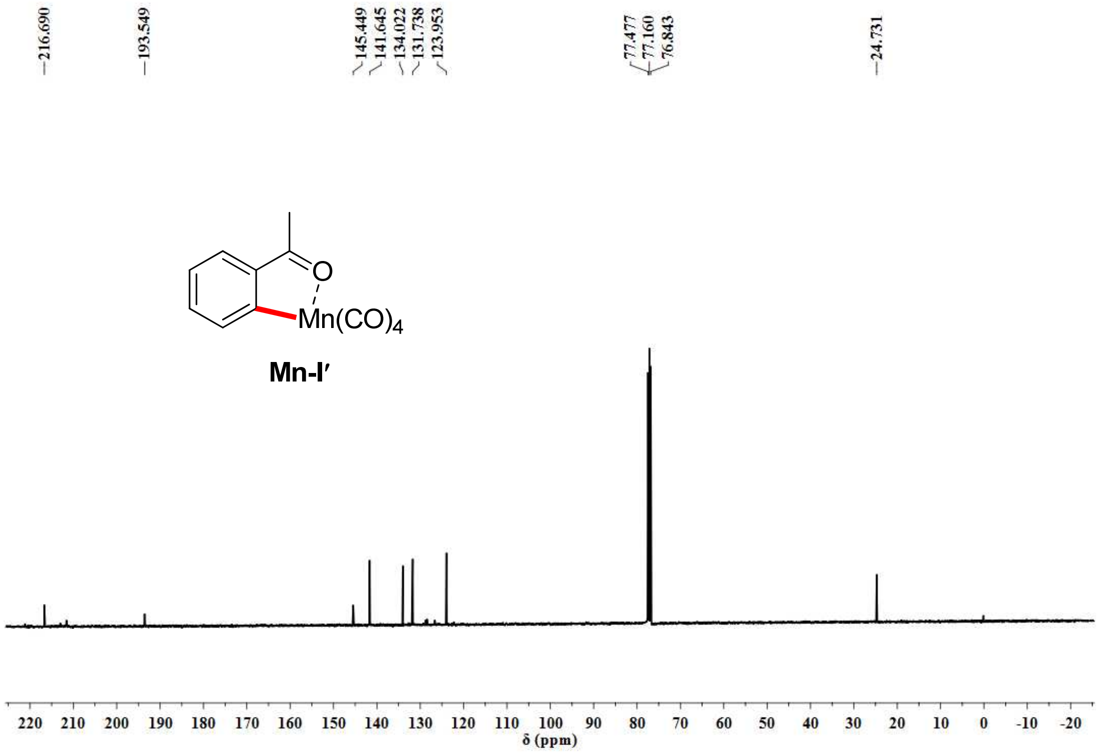

Supplementary Figure 14. ^{13}C NMR spectrum of Mn-I'

Next, we examined the stoichiometric reaction of manganacycle **Mn-I** with (*E*)-*N*-benzylidenethiophene-2-sulfonamide **2a** at 60 °C in DCE. The product **3a** was obtained in 27% NMR yield after 10 h. Adding Me_2Zn or $ZnBr_2$ could further improve the product formation, providing **3a** in 54% and 68% yield, respectively.

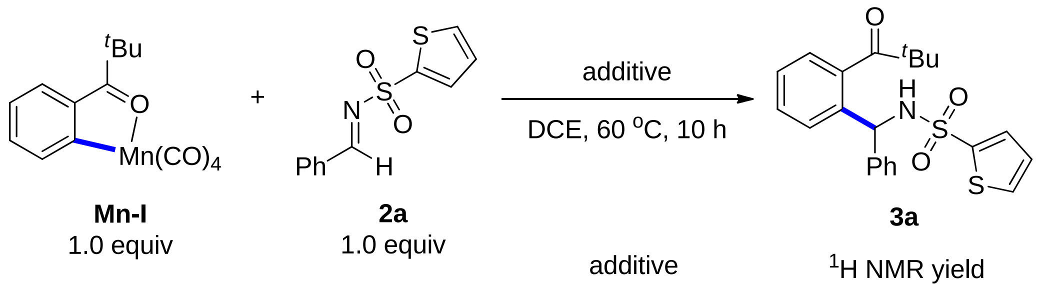

additive	^{1}H NMR yield
-	27%
Me_2Zn (1.5 equiv)	54%
$ZnBr_2$ (1.0 equiv)	68%

Supplementary Figure 15. Reaction of Mn-I with imine

Experimental procedure:

Mn-I (0.04 mmol, 13.1 mg), Me_2Zn (0.06 mmol, 1.2 M in toluene), or $ZnBr_2$ (0.04 mmol, 9.0 mg) were added as indicated in the above scheme into an oven-dried reaction vessel with Teflon screw cap under a nitrogen atmosphere. DCE (0.5 mL) and (*E*)-*N*-benzylidenethiophene-2-sulfonamide (0.04 mmol, 10.0 mg) were then injected into the reaction tube. The reaction mixture was stirred at 60 °C for 10 h. After the completion, the mixture was diluted with ethyl acetate (20 mL) and filtered through a short pad of silica gel. The solvent was removed by rotary evaporation. The yield of **3a** was determined by ^{1}H NMR analysis of the crude product using 1,3,5-trimethoxybenzene as an internal standard.

Finally, we examined the reaction of 2,2-dimethyl-1-phenylpropan-1-one **1a** with (*E*)-*N*-benzylidenethiophene-2-sulfonamide **2a** using the manganacycle **Mn-I** or $MnMe(CO)_5$ as a catalyst under the standard conditions. Gratifyingly, the reactions proceeded smoothly to provide the corresponding product **3a** in 74% and 80% NMR yield, respectively. It indicated that manganacycle **Mn-I** and $MnMe(CO)_5$ might be the key reaction intermediates involved in the catalytic reaction.

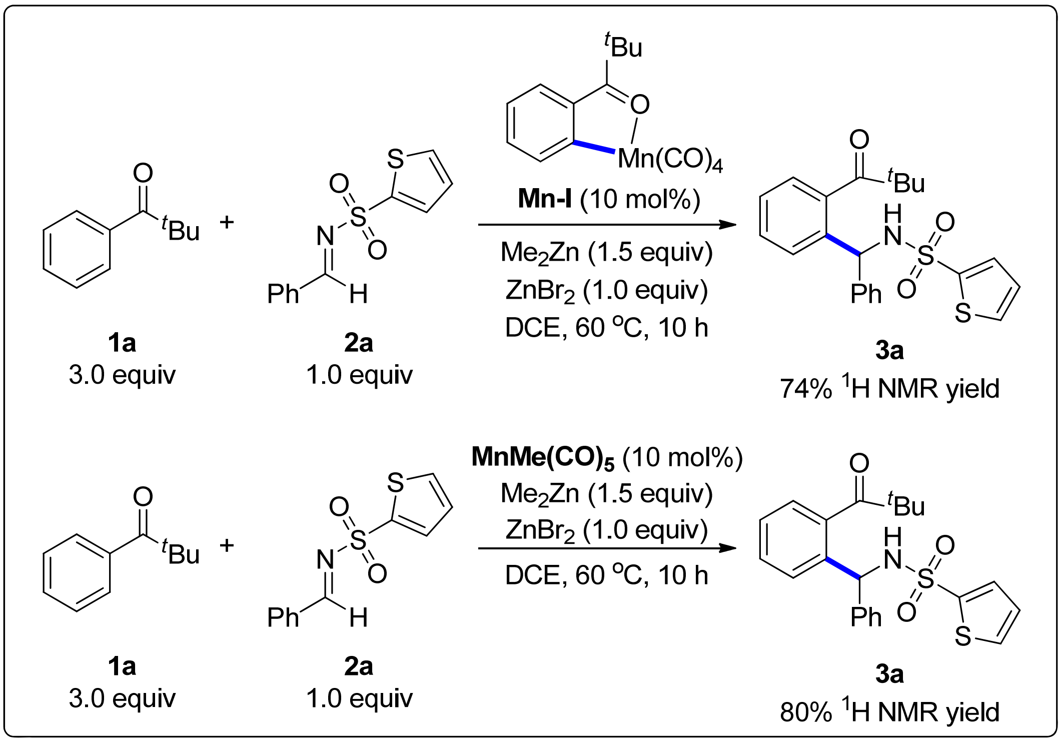

Supplementary Figure 16. Catalytic reactions with Mn-I and $MnMe(CO)_5$

Experimental procedure:

To a 25 ml flame-dried Schlenk tube was added $ZnBr_2$ (0.1 mmol, 22.5 mg), **Mn-I** or $MnMe(CO)_5$ (0.01 mmol, 10. mol%), DCE (1.0 mL), (*E*)-*N*-benzylidene -thiophene-2-sulfonamide **2a** (0.1 mmol, 25.1 mg), 2,2-dimethyl-1-phenylpropan-1-one **1a** (0.3 mmol, 48.6 mg) and Me_2Zn (0.15 mmol, 1.2 M in toluene) sequentially under nitrogen. The tube was sealed and stirred at 60 °C for 10 h. After completion, the reaction mixture was diluted with ethyl acetate (5.0 mL) and filtered through a short pad silica gel washing with ethyl acetate (20 mL). The filtrate was concentrated and the yield of **3a** was determined by ^1H NMR analysis of the crude product using 1,3,5-trimethoxybenzene as an internal standard.

Deuterium-labeling experiments

tert-Butyl(pentaduteriophenyl)methone **1a-*d*5** was prepared from commercially available bromobenzene-*d5* (>99% D) according to the *procedure B*.

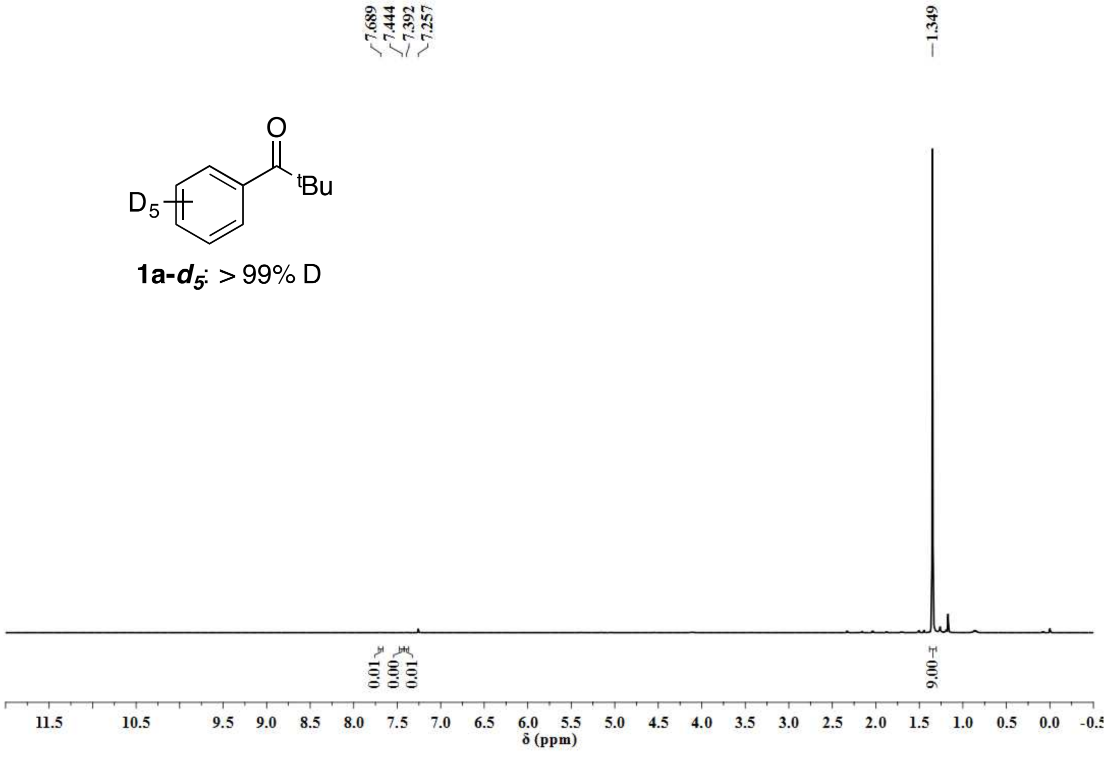

Supplementary Figure 17. ^1H NMR spectrum of 1a-*d*$_5$

In order to acquire more details about the C–H bond cleavage, we carried out a series of deuterium-labeling experiments. First, the *tert*-butyl(pentadeuteriophenyl)-methone **1a-*d*$_5$** was subjected to the standard conditions in the absence of (*E*)-*N*-benzylidenethiophene-2-sulfonamide **2a**. After 10 hours, a negligible loss of deuterium was observed at the *ortho* position of **1a-*d*$_5$** by ^{1}H NMR analysis. It indicated that the C–H activation step might be an irreversible process.

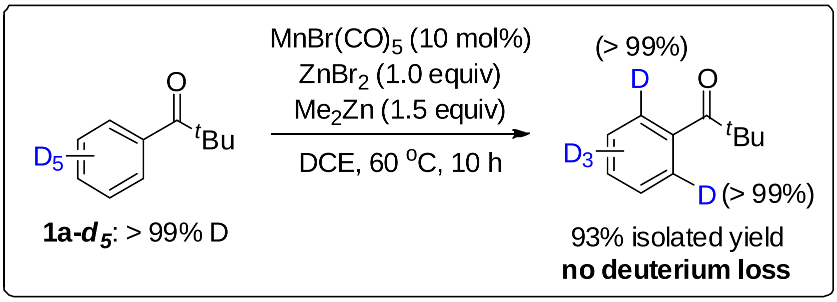

Supplementary Figure 18. Reaction of 1a-*d*$_5$

Experimental procedure:

tert-Butyl(pentaduteriophenyl)methone **1a-*d*$_5$** (0.5 mmol, 83.5 mg), $MnBr(CO)_5$ (0.05 mmol, 13.8 mg) and $ZnBr_2$ (0.5 mmol, 112.5 mg) were added into an oven-dried reaction vessel with Teflon screw cap under a nitrogen atmosphere. DCE (5.0 mL) and Me_2Zn (0.75 mmol, 1.2 M in toluene) were then injected into the reaction tube. The reaction mixture was stirred at 60 ^{o}C for 10 h. After completion, the reaction mixture was filtered through a short pad of silica gel and washed with ethyl acetate. The filtrate was evaporated in vacuo and the residue was purified by silica gel column chromatography (93% isolated yield), which was analyzed by ^{1}H NMR in $CDCl_3$.

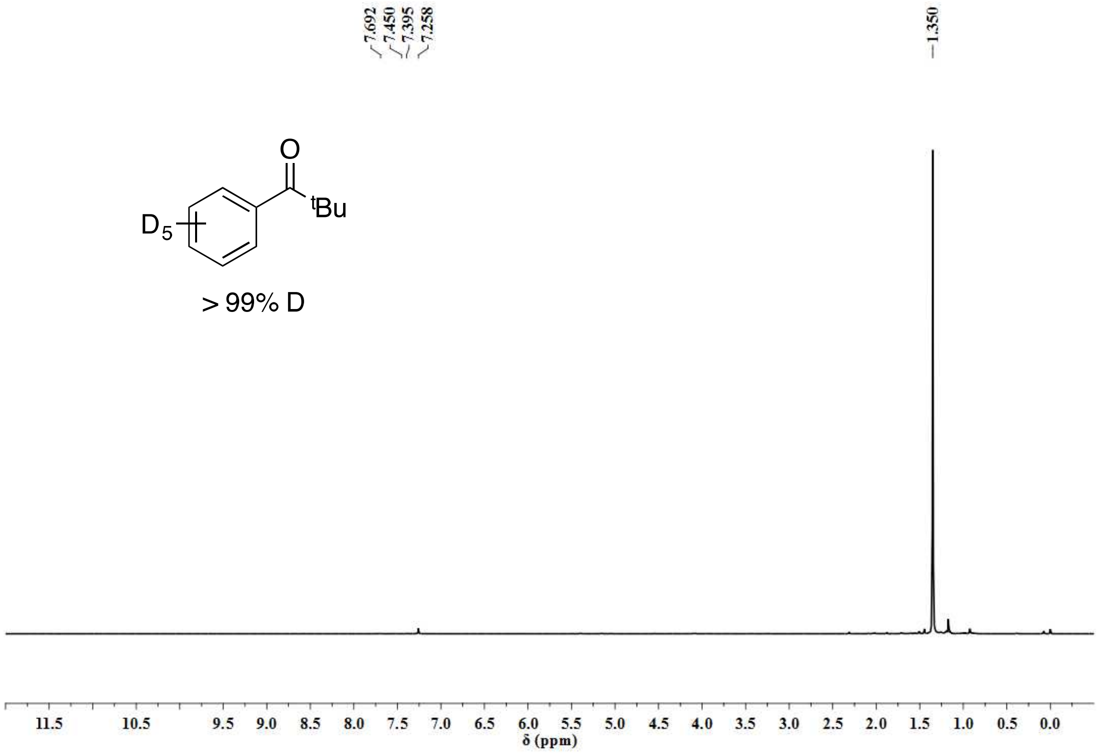

Supplementary Figure 19. ^{1}**H NMR spectrum of 1a-*d*$_5$ after reaction**

Second, two parallel reactions of **1a** and **1a-*d*$_5$** with **2a** respectively were performed to examine the corresponding k_H and k_D value. The KIE value was measured to be 3.2. The result implied that the C-H cleavage was involved in the turnover-limiting step in the reaction.

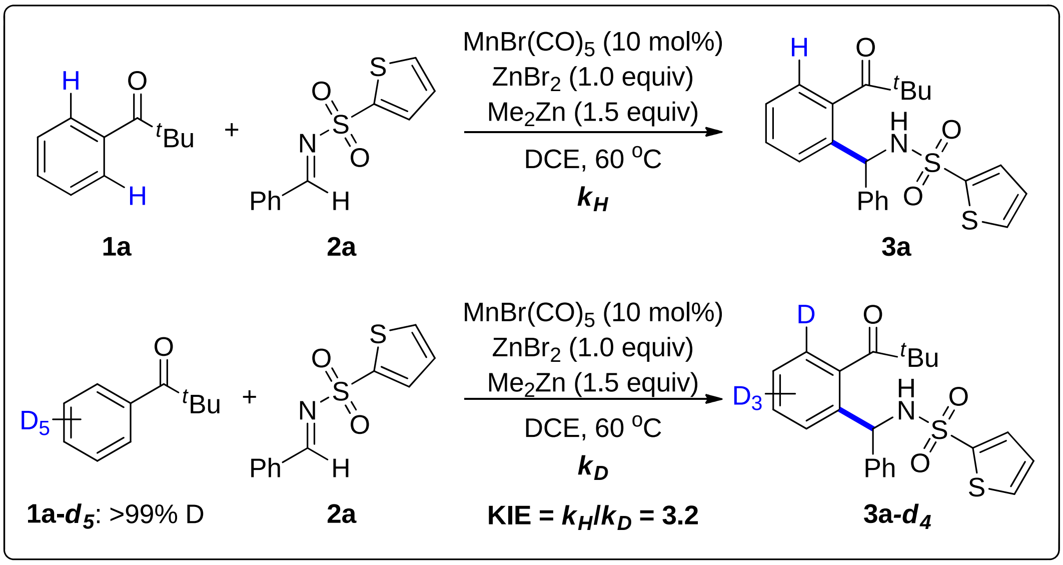

Supplementary Figure 20. Parallel KIE experiments

Experimental procedure:

1a (0.6 mmol, 97.2 mg) and **1a-*d*$_5$** (0.6 mmol, 100.2 mg) were placed in a flame-dried Schlenk tube respectively, and then treated with the same mixture of **2a** (0.2 mmol, 50.2 mg), $MnBr(CO)_5$ (0.02 mmol, 5.5 mg), $ZnBr_2$ (0.2 mmol, 45.0 mg), Me_2Zn (0.3 mmol, 1.2 M in toluene), 1,3,5-trimethoxybenzene (internal standard, 0.067 mmol, 11.2 mg) in DCE (2.0 mL) at 60 °C under N_2 atmosphere. Each reaction was sampled at the following indicated points and analyzed by GC. The GC yields were calculated after calibrating the response of GC.

Time (min)	0	2	4	6	8	10
GC yield of **3a** (%)	0	1.6	12.1	24.9	38.2	45.4

Time (min)	0	2	4	8	12	16
GC yield of **3a-*d*$_5$** (%)	0	1.2	3.6	10.2	19.5	24.9

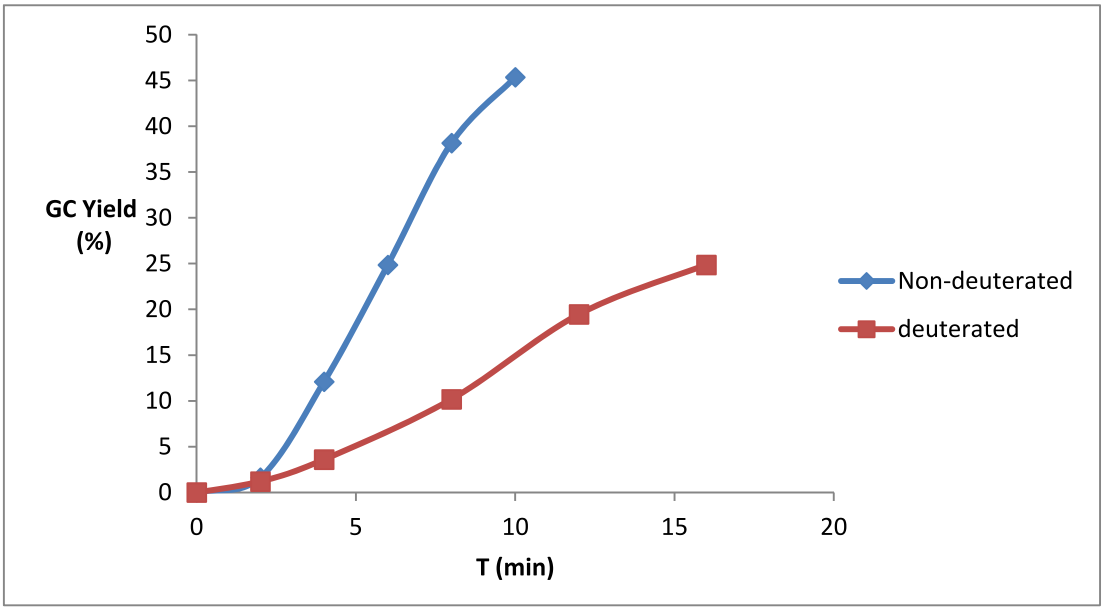

Supplementary Figure 21. Plot of 1a for KIE experiments

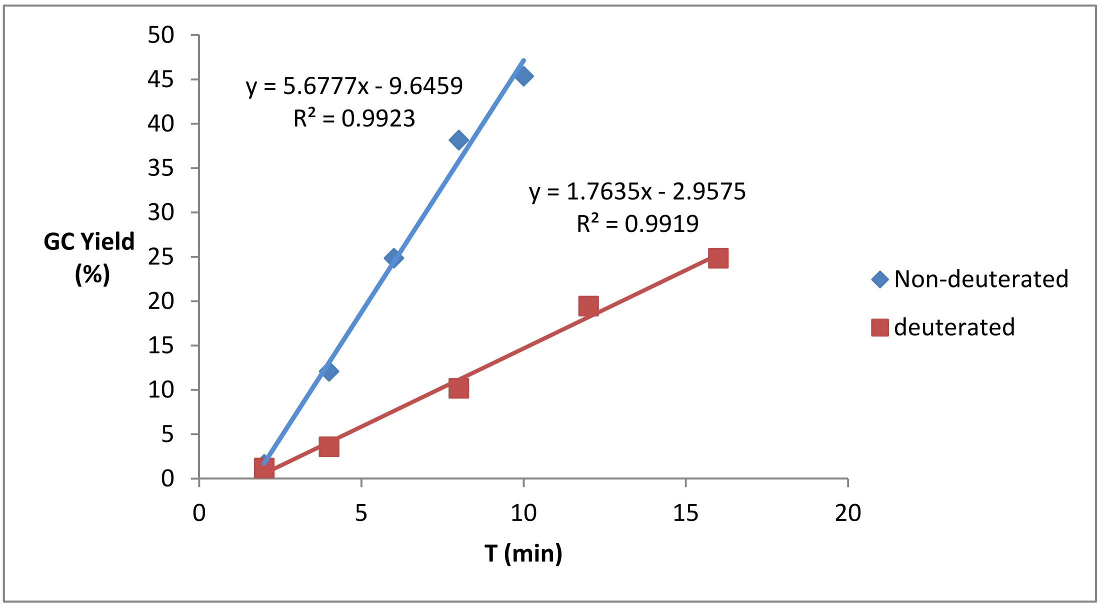

Supplementary Figure 22. Plot of 1a-d_5 for KIE experiments

X-Ray crystallography data for 3H, 4a, 4e, 5c, 5d, Mn-I'

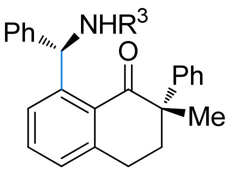

3H

CCDC 1563929

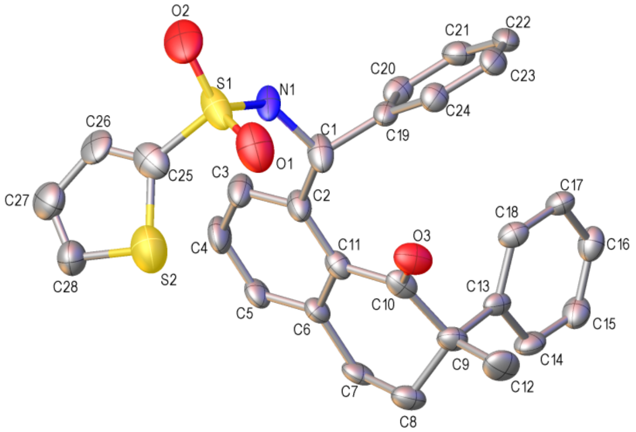

Supplementary Figure 23. X-Ray structure of 3H

Supplementary Table 2. Crystal data and structure refinement for 3H.

Identification code	**3H**	
Empirical formula	C28 H25 N O3 S2	
Formula weight	487.61	
Temperature	173.15 K	
Wavelength	0.71073 Å	
Crystal system	Monoclinic	
Space group	C 1 2 1	
Unit cell dimensions	a = 18.8409(14) Å	α= 90°.
	b = 6.1715(4) Å	β= 111.489(8)°.
	c = 22.9464(18) Å	γ = 90°.
Volume	2482.7(3) Å^3	
Z	4	
Density (calculated)	1.305 Mg/m^3	
Absorption coefficient	0.245 mm^{-1}	
F(000)	1024	
Crystal size	0.5 x 0.18 x 0.16 mm^3	

Theta range for data collection	2.164 to 25.190°.
Index ranges	-22<=h<=21, -7<=k<=7, -27<=l<=27
Reflections collected	8700
Independent reflections	4104 [R(int) = 0.0458]
Completeness to theta = 25.190°	99.8 %
Absorption correction	Semi-empirical from equivalents
Max. and min. transmission	1.0000 and 0.789
Refinement method	Full-matrix least-squares on F^2
Data / restraints / parameters	4104 / 301 / 390
Goodness-of-fit on F^2	1.042
Final R indices [I>2sigma(I)]	R1 = 0.0842, wR2 = 0.2104
R indices (all data)	R1 = 0.1156, wR2 = 0.2397
Absolute structure parameter	0.19(10)
Extinction coefficient	n/a
Largest diff. peak and hole	0.441 and -0.403 e.$Å^{-3}$

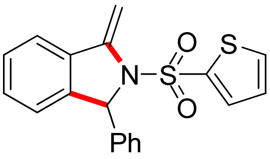

4a

CCDC: 1532722

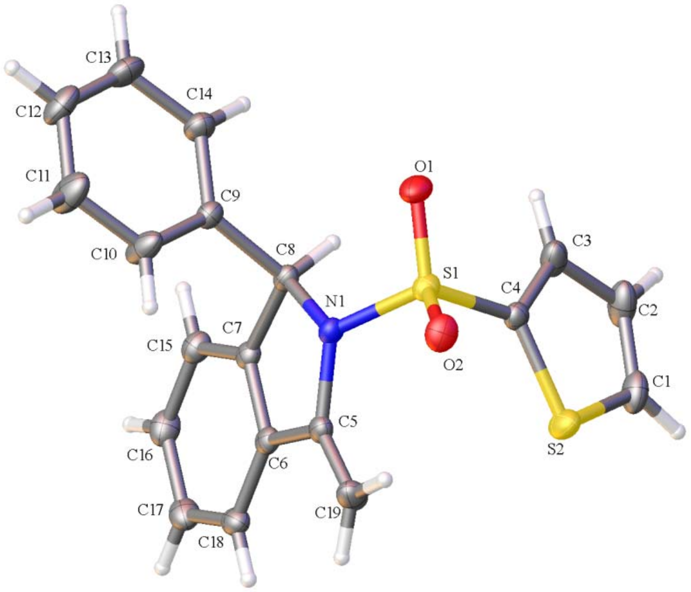

Supplementary Figure 24. X-Ray structure of 4a

Supplementary Table 3. Crystal data and structure refinement for 4a

Identification code	**4a**	
Empirical formula	C19 H15 N O2 S2	
Formula weight	353.44	
Temperature	173.1500 K	
Wavelength	0.71073 Å	
Crystal system	Monoclinic	
Space group	P 1 21/c 1	
Unit cell dimensions	a = 16.000(3) Å	a= 90°.
	b = 9.1080(18) Å	b= 101.81(3)°.
	c = 11.856(2) Å	g = 90°.
Volume	1691.1(6) Å^3	
Z	4	
Density (calculated)	1.388 Mg/m^3	
Absorption coefficient	0.326 mm^{-1}	
F(000)	736	
Crystal size	0.213 x 0.186 x 0.174 mm^3	

Theta range for data collection	2.601 to 27.481°.
Index ranges	-20<=h<=20, -11<=k<=11, -15<=l<=15
Reflections collected	18407
Independent reflections	3858 [R(int) = 0.0345]
Completeness to theta = 26.000°	99.5 %
Absorption correction	Semi-empirical from equivalents
Max. and min. transmission	1.0000 and 0.8753
Refinement method	Full-matrix least-squares on F^2
Data / restraints / parameters	3858 / 0 / 217
Goodness-of-fit on F^2	1.221
Final R indices [I>2sigma(I)]	R1 = 0.0487, wR2 = 0.1090
R indices (all data)	R1 = 0.0500, wR2 = 0.1097
Extinction coefficient	n/a
Largest diff. peak and hole	0.285 and -0.239 e.Å^{-3}

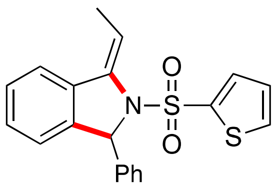

4e

CCDC: 1532723

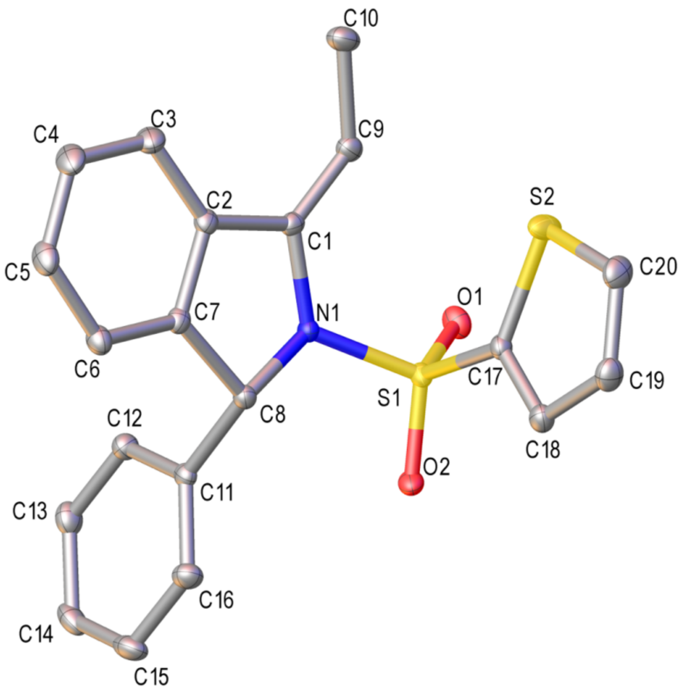

Supplementary Figure 25. X-Ray structure of 4e

Supplementary Table 4. Crystal data and structure refinement for 4e.

Identification code	**4e**	
Empirical formula	C20 H17 N O2 S2	
Formula weight	367.46	
Temperature	173.1500 K	
Wavelength	0.71073 Å	
Crystal system	Monoclinic	
Space group	P 1 21/n 1	
Unit cell dimensions	a = 6.0055(11) Å	α= 90°.
	b = 19.774(4) Å	β= 95.394(2)°.
	c = 14.612(3) Å	γ = 90°.
Volume	1727.5(6) Å^3	
Z	4	

Density (calculated)	1.413 Mg/m^3
Absorption coefficient	0.322 mm^{-1}
F(000)	768
Crystal size	0.529 x 0.506 x 0.483 mm^3
Theta range for data collection	1.738 to 27.477°.
Index ranges	-7<=h<=7, -25<=k<=25, -18<=l<=18
Reflections collected	10648
Independent reflections	3915 [R(int) = 0.0235]
Completeness to theta = 26.000°	98.9 %
Absorption correction	Semi-empirical from equivalents
Max. and min. transmission	1.0000 and 0.7472
Refinement method	Full-matrix least-squares on F^2
Data / restraints / parameters	3915 / 0 / 227
Goodness-of-fit on F^2	1.085
Final R indices [I>2sigma(I)]	R1 = 0.0361, wR2 = 0.0899
R indices (all data)	R1 = 0.0380, wR2 = 0.0919
Extinction coefficient	n/a
Largest diff. peak and hole	0.259 and -0.379 e.$Å^{-3}$

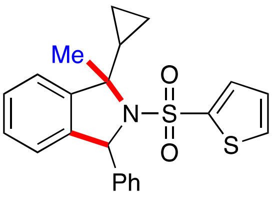

5c

CCDC: 1532725

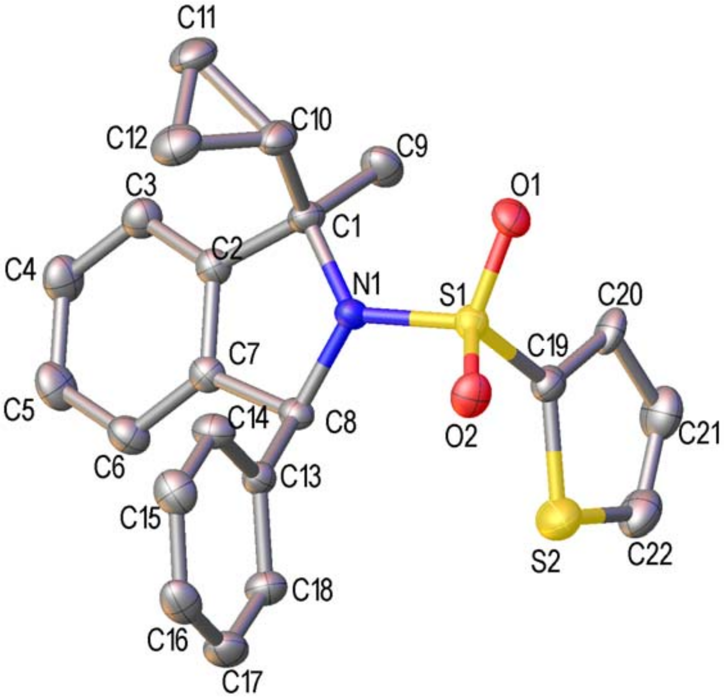

Supplementary Figure 26. X-Ray structure of 5c

Supplementary Table 5. Crystal data and structure refinement for 5c.

Identification code	**5c**	
Empirical formula	C22 H21 N O2 S2	
Formula weight	395.52	
Temperature	173.1500 K	
Wavelength	0.71073 Å	
Crystal system	Monoclinic	
Space group	P 1 21/c 1	
Unit cell dimensions	a = 9.498(2) Å	α= 90°.
	b = 15.525(4) Å	β= 95.666(2)°.
	c = 12.999(3) Å	γ = 90°.
Volume	1907.4(7) Å^3	

Z	4
Density (calculated)	1.377 Mg/m^3
Absorption coefficient	0.297 mm^{-1}
F(000)	832
Crystal size	0.41 x 0.26 x 0.14 mm^3
Theta range for data collection	2.049 to 27.489°.
Index ranges	-12<=h<=12, -20<=k<=20, -16<=l<=16
Reflections collected	13352
Independent reflections	4319 [R(int) = 0.0312]
Completeness to theta = 26.000°	99.0 %
Absorption correction	Semi-empirical from equivalents
Max. and min. transmission	1.0000 and 0.8354
Refinement method	Full-matrix least-squares on F^2
Data / restraints / parameters	4319 / 1 / 245
Goodness-of-fit on F^2	1.124
Final R indices [I>2sigma(I)]	R1 = 0.0543, wR2 = 0.1291
R indices (all data)	R1 = 0.0586, wR2 = 0.1326
Extinction coefficient	n/a
Largest diff. peak and hole	0.605 and -0.555 e.Å^{-3}

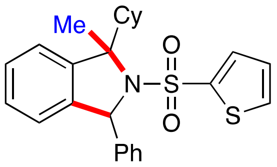

5d

CCDC: 1532724

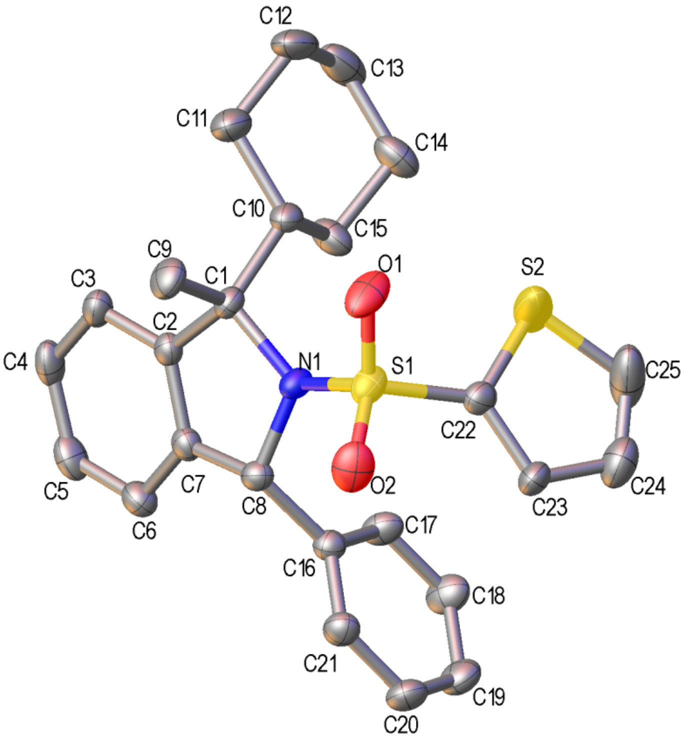

Supplementary Figure 27. X-Ray structure of 5d

Supplementary Table 6. Crystal data and structure refinement for 5d.

Identification code	**5d**	
Empirical formula	C25 H27 N O2 S2	
Formula weight	437.59	
Temperature	173.1500 K	
Wavelength	0.71073 Å	
Crystal system	Monoclinic	
Space group	P 1 21/c 1	
Unit cell dimensions	a = 10.498(2) Å	α= 90°.
	b = 12.404(3) Å	β= 93.234(4)°.
	c = 17.077(4) Å	γ = 90°.

Volume	2220.2(9) $Å^3$
Z	4
Density (calculated)	1.309 Mg/m^3
Absorption coefficient	0.262 mm^{-1}
F(000)	928
Crystal size	0.57 x 0.49 x 0.38 mm^3
Theta range for data collection	2.899 to 27.502°.
Index ranges	-13<=h<=13, -16<=k<=16, -21<=l<=22
Reflections collected	14571
Independent reflections	5053 [R(int) = 0.0263]
Completeness to theta = 26.000°	99.4 %
Absorption correction	Semi-empirical from equivalents
Max. and min. transmission	1.0000 and 0.8745
Refinement method	Full-matrix least-squares on F^2
Data / restraints / parameters	5053 / 0 / 272
Goodness-of-fit on F^2	1.125
Final R indices [I>2sigma(I)]	R1 = 0.0523, wR2 = 0.1279
R indices (all data)	R1 = 0.0556, wR2 = 0.1301
Extinction coefficient	n/a
Largest diff. peak and hole	0.335 and -0.497 e.$Å^{-3}$

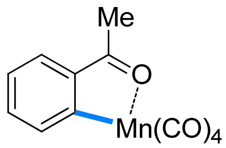

Mn-I'

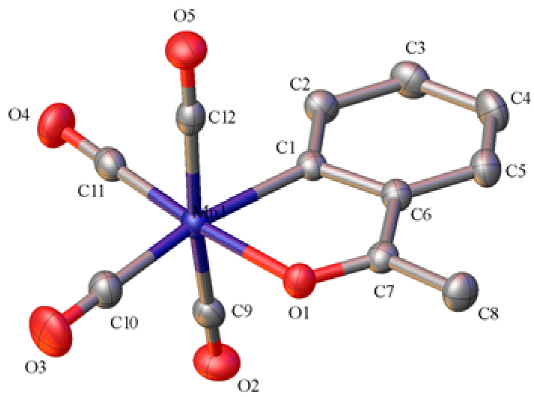

Supplementary Figure 28. X-Ray structure of Mn-I'

Supplementary Table 7. Crystal data and structure refinement for Mn-I'

Identification code	**Mn-I'**	
Empirical formula	C12 H7 Mn O5	
Formula weight	286.12	
Temperature	173.1500 K	
Wavelength	0.71073 Å	
Crystal system	Monoclinic	
Space group	C 1 2/c 1	
Unit cell dimensions	a = 25.862(7) Å	α= 90°.
	b = 5.9069(14) Å	β= 115.909(2)°.
	c = 16.952(4) Å	γ = 90°.
Volume	2329.4(10) $Å^3$	
Z	8	
Density (calculated)	1.632 Mg/m^3	
Absorption coefficient	1.142 mm^{-1}	
F(000)	1152	
Crystal size	0.24 x 0.17 x 0.05 mm^3	
Theta range for data collection	2.672 to 27.501°.	
Index ranges	-33<=h<=32, -7<=k<=7, -22<=l<=22	
Reflections collected	8733	
Independent reflections	2664 [R(int) = 0.0338]	

Completeness to theta = 26.000°	99.6 %
Absorption correction	Semi-empirical from equivalents
Max. and min. transmission	1.0000 and 0.7229
Refinement method	Full-matrix least-squares on F^2
Data / restraints / parameters	2664 / 0 / 164
Goodness-of-fit on F^2	1.120
Final R indices [I>2sigma(I)]	R1 = 0.0339, wR2 = 0.0824
R indices (all data)	R1 = 0.0356, wR2 = 0.0836
Extinction coefficient	n/a
Largest diff. peak and hole	0.376 and -0.256 e.Å^{-3}

Supplementary Figure 29. Characterization of product 3a. (a) ^{1}H NMR spectrum. (b) ^{13}C-NMR spectrum.

a

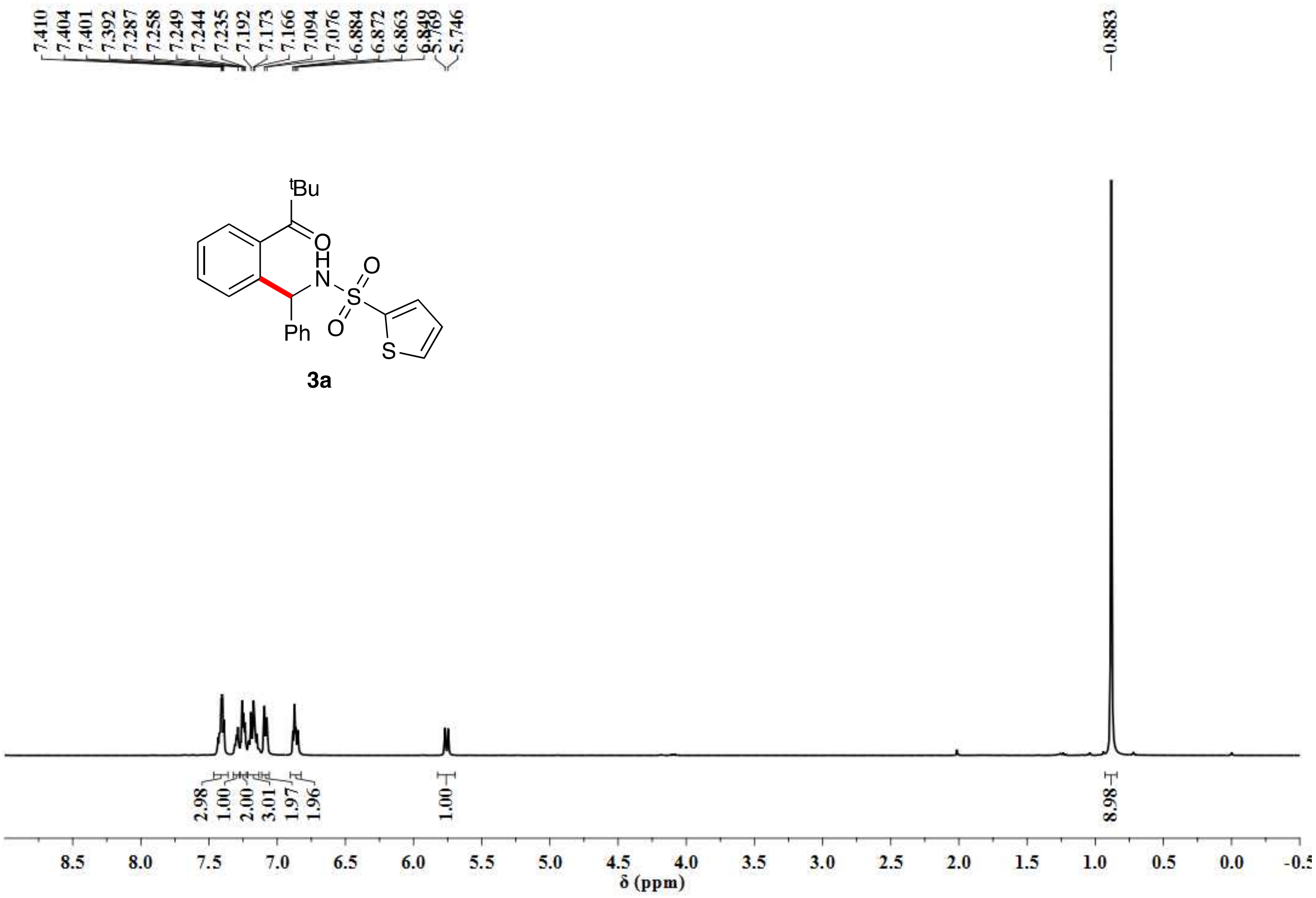

b

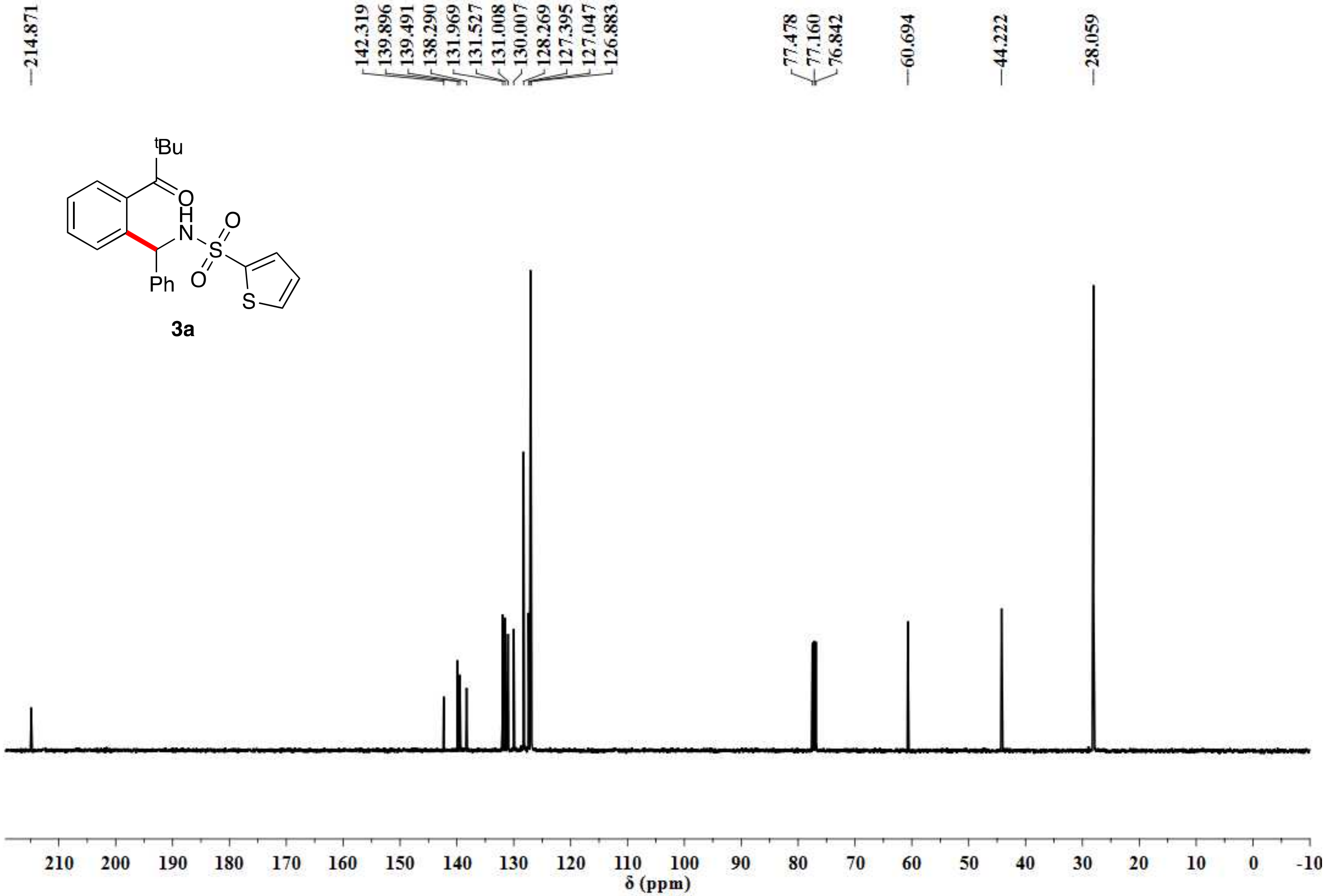
214.871
142.319
139.896
139.491
138.290
131.969
131.527
131.008
130.007
128.269
127.395
127.047
126.883
77.478
77.160
76.842
60.694
44.222
28.059
tBu
O
H
N
S
O
O
Ph
S
3a
210 200 190 180 170 160 150 140 130 120 110 100 90 80 70 60 50 40 30 20 10 0 -10
δ (ppm)

Supplementary Figure 30. Characterization of product 3b. (a) ^{1}H NMR spectrum. (b) ^{13}C-NMR spectrum.

a

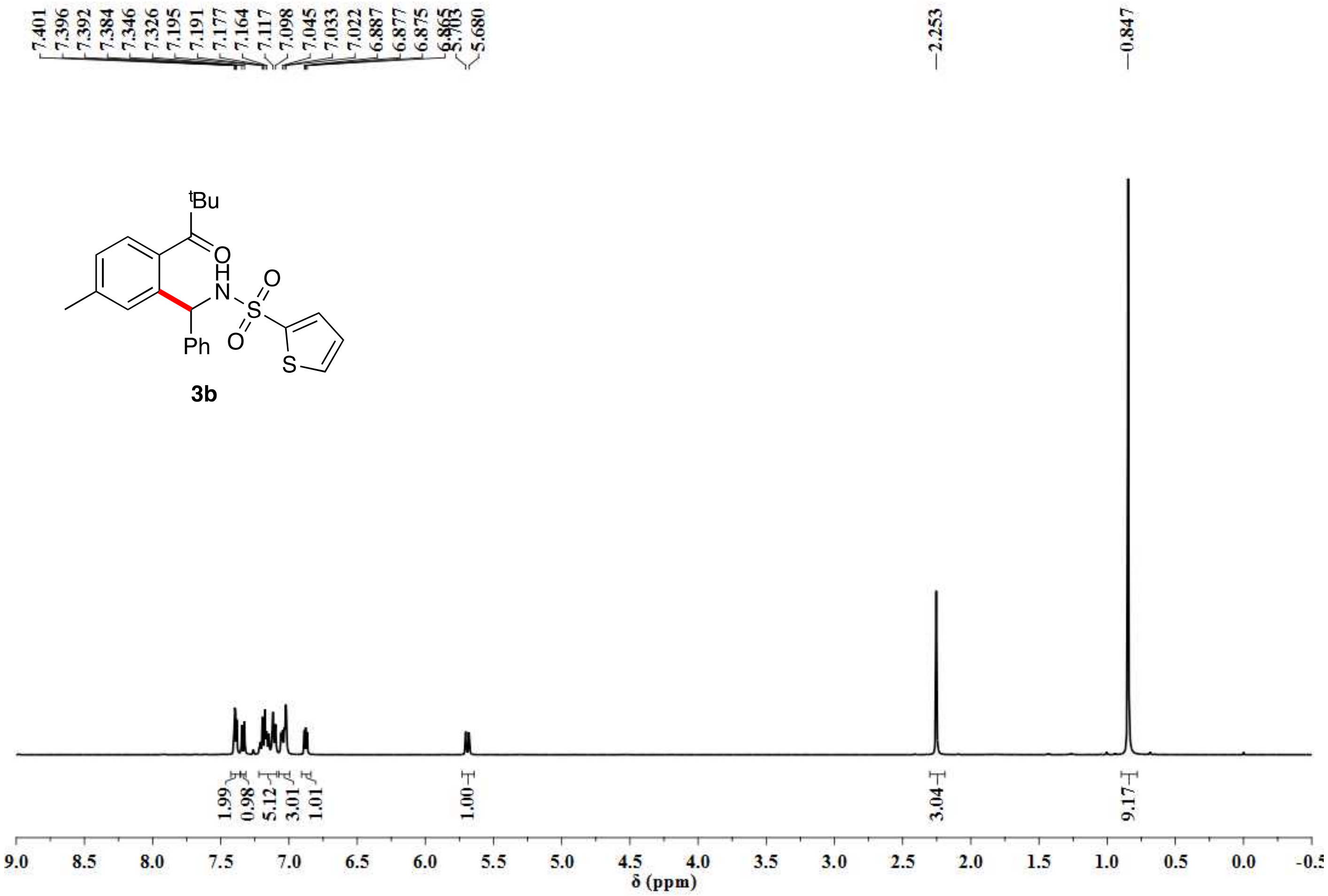

b

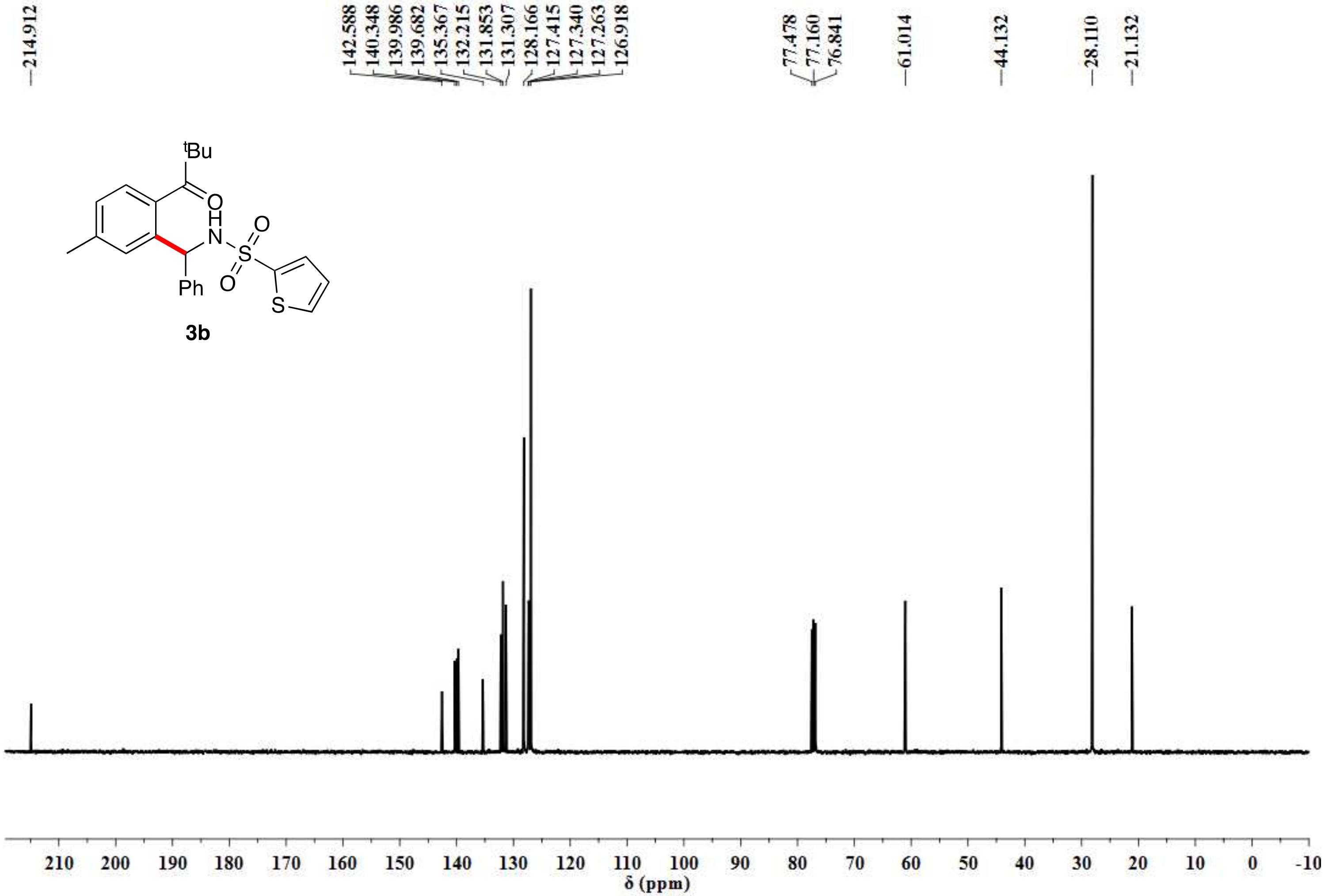
214.912
142.588
140.348
139.986
139.682
135.367
132.215
131.853
131.307
128.166
127.415
127.340
127.263
126.918
77.478
77.160
76.841
61.014
44.132
28.110
21.132
tBu
O
H
N
S
O
O
Ph
S
3b
210 200 190 180 170 160 150 140 130 120 110 100 90 80 70 60 50 40 30 20 10 0 -10
δ (ppm)

Supplementary Figure 31. Characterization of product 3c. (a) ^{1}H NMR spectrum. (b) ^{13}C-NMR spectrum.

a

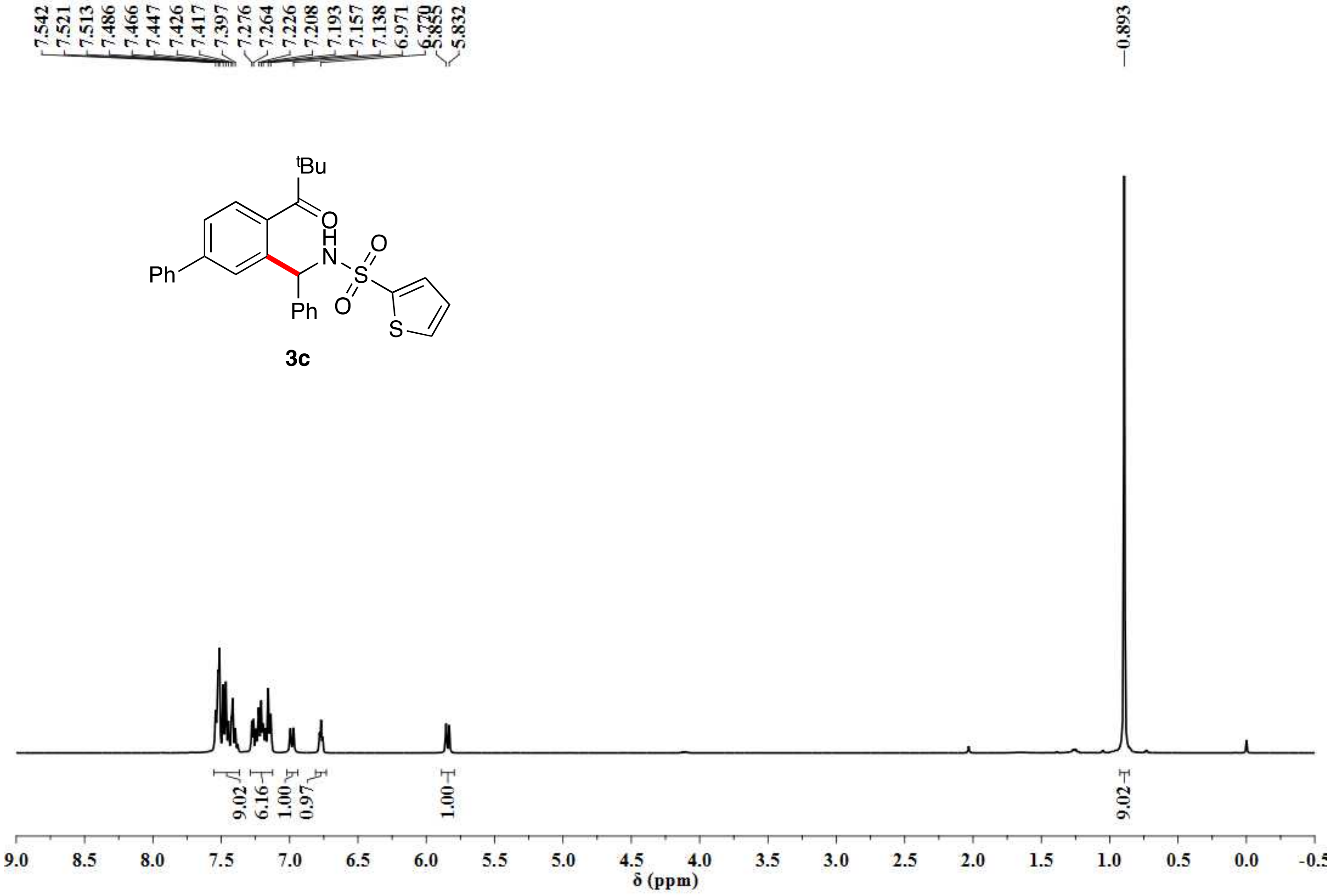

b

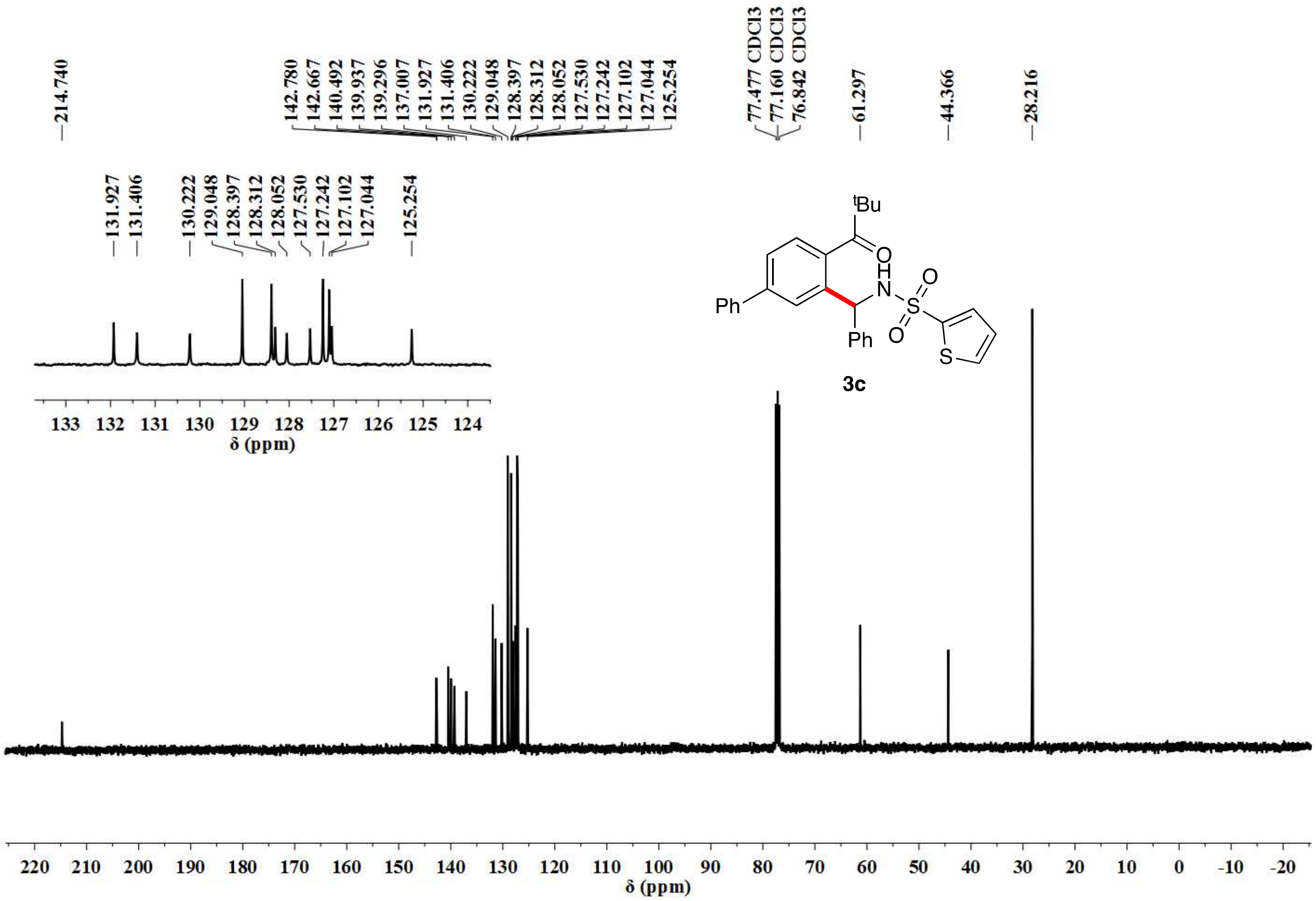

214.740
142.780
142.667
140.492
139.937
139.296
137.007
131.927
131.406
130.222
129.048
128.397
128.312
128.052
127.530
127.242
127.102
127.044
125.254
77.477 CDCl3
77.160 CDCl3
76.842 CDCl3
61.297
44.366
28.216
δ (ppm)
tBu
O
H
N
S
O
O
Ph
Ph
S
3c

Supplementary Figure 32. Characterization of product 3d. (a) ^{1}H NMR spectrum. (b) ^{13}C-NMR spectrum.

a

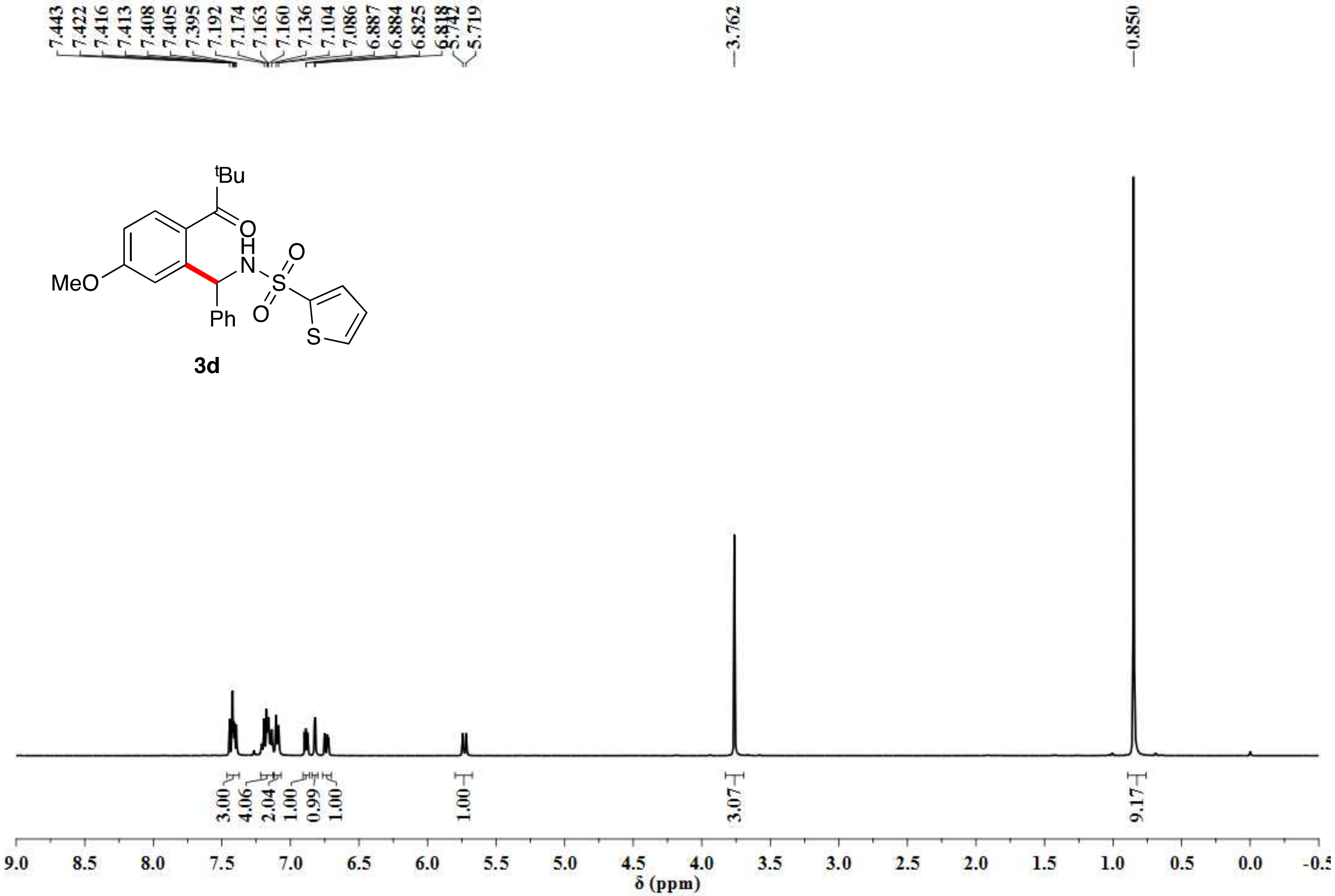

b

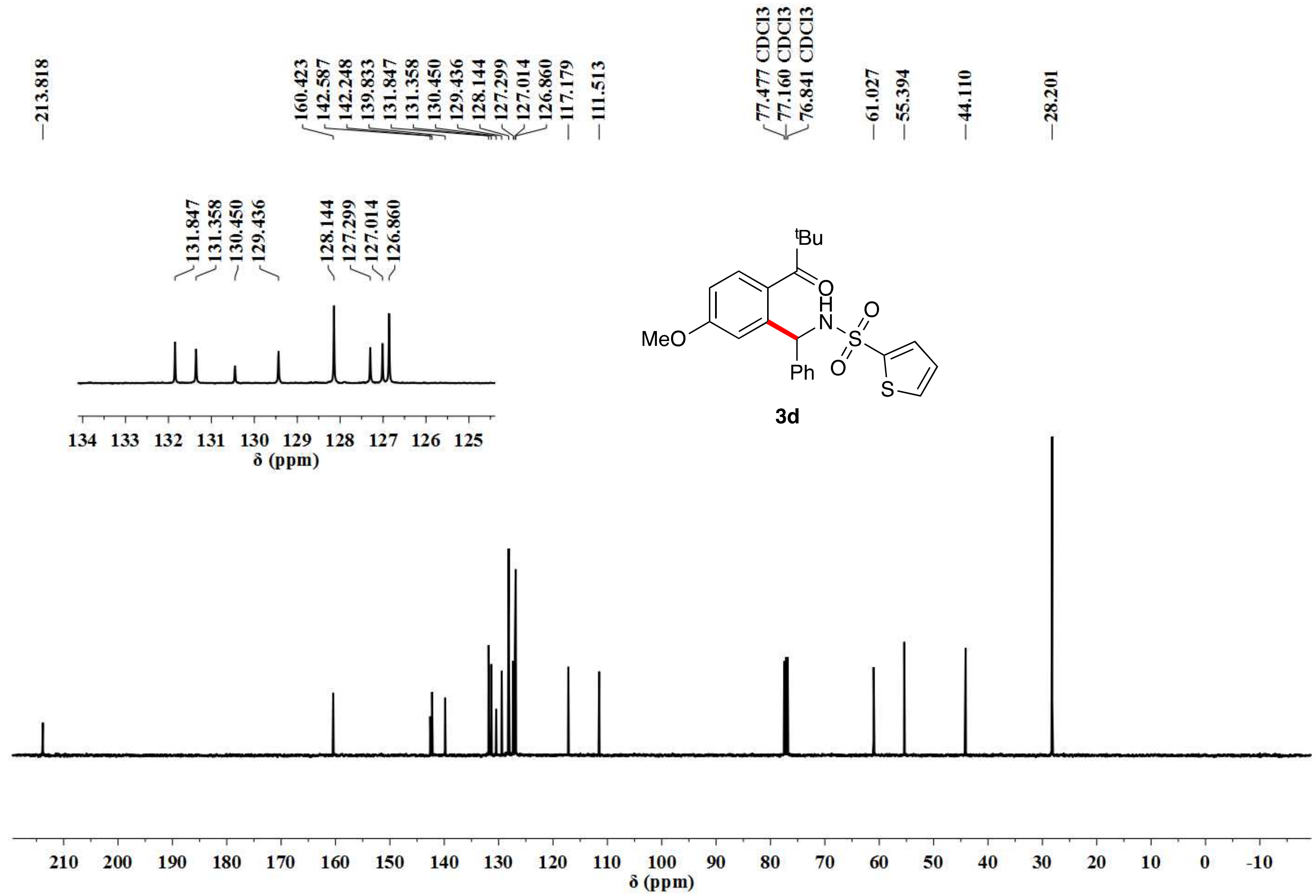
213.818
160.423
142.587
142.248
139.833
131.847
131.358
130.450
129.436
128.144
127.299
127.014
126.860
117.179
111.513
77.477 CDCl3
77.160 CDCl3
76.841 CDCl3
61.027
55.394
44.110
28.201
131.847
131.358
130.450
129.436
128.144
127.299
127.014
126.860
134 133 132 131 130 129 128 127 126 125
δ (ppm)
tBu
O
H
N
O
S
O
MeO
Ph
S
3d
210 200 190 180 170 160 150 140 130 120 110 100 90 80 70 60 50 40 30 20 10 0 -10
δ (ppm)

Supplementary Figure 33. Characterization of product 3e. (a) ^{1}H NMR spectrum. (b) ^{13}C-NMR spectrum.

a

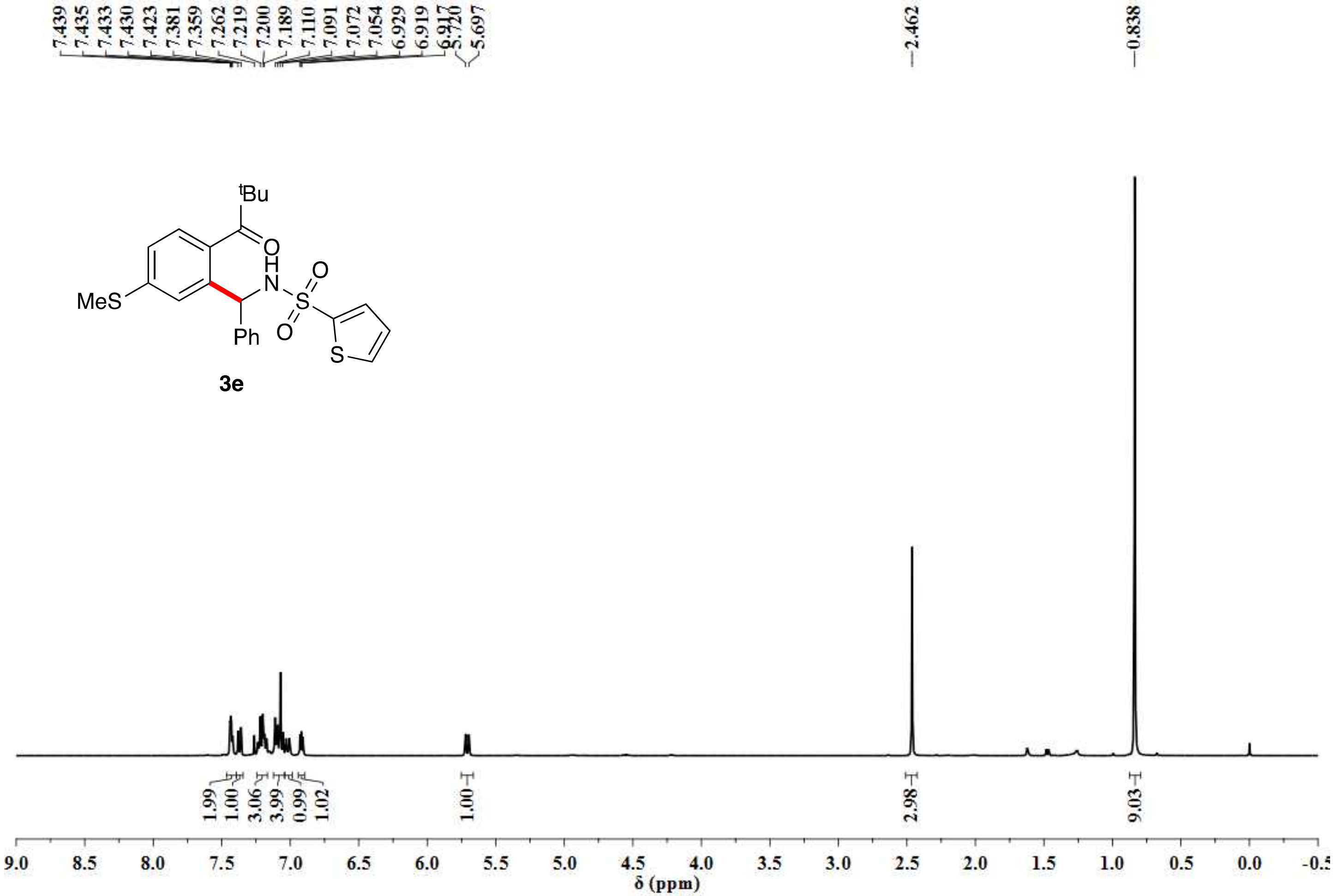

b

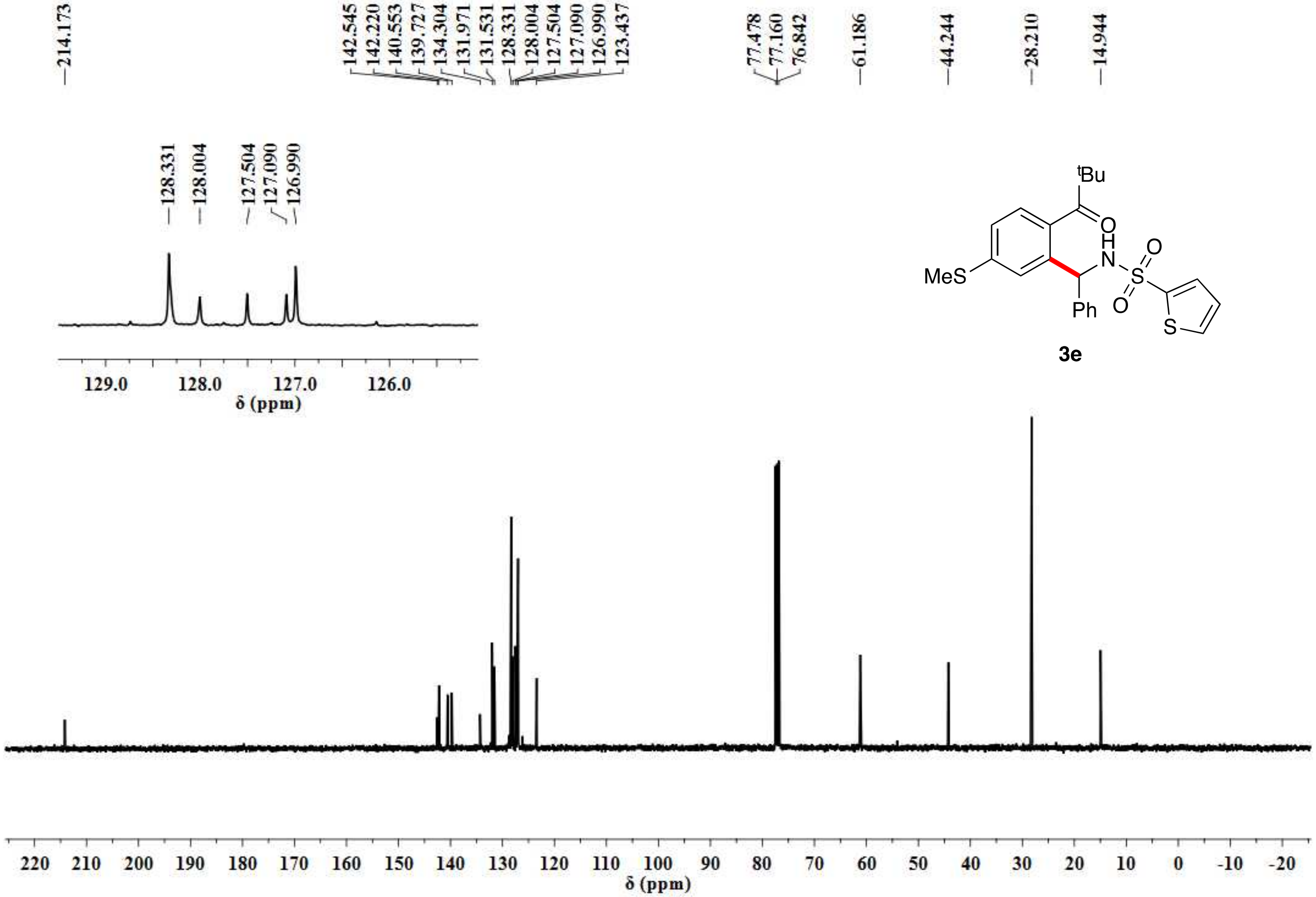
214.173
142.545
142.220
140.553
139.727
134.304
131.971
131.531
128.331
128.004
127.504
127.090
126.990
123.437
77.478
77.160
76.842
61.186
44.244
28.210
14.944
128.331
128.004
127.504
127.090
126.990
129.0 128.0 127.0 126.0
δ (ppm)
tBu
O
H
N
O
S
O
MeS
Ph
S
220 210 200 190 180 170 160 150 140 130 120 110 100 90 80 70 60 50 40 30 20 10 0 -10 -20
δ (ppm)

3e

Supplementary Figure 34. Characterization of product 3f. (a) ^{1}H NMR spectrum. (b) ^{13}C-NMR spectrum.

a

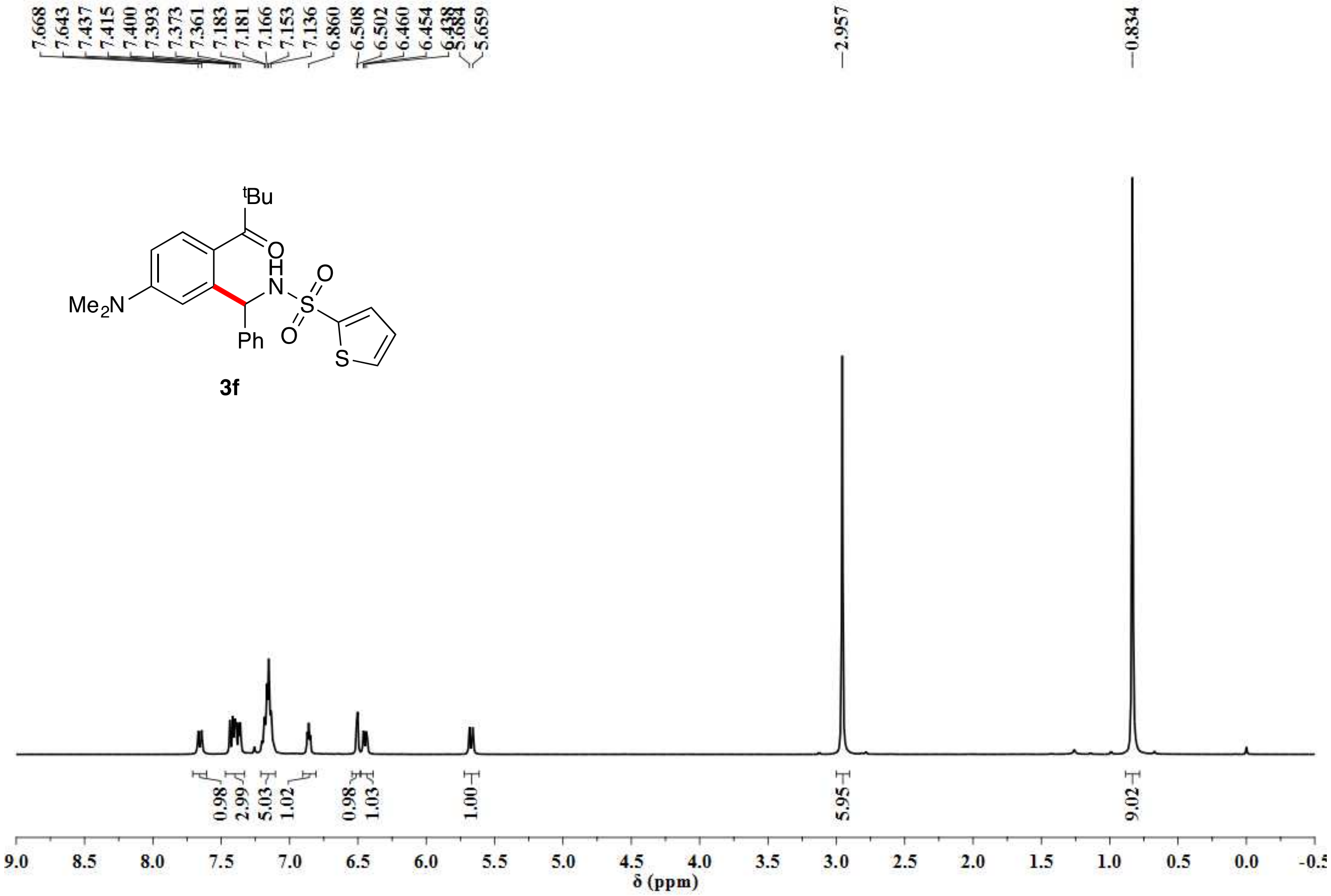

b

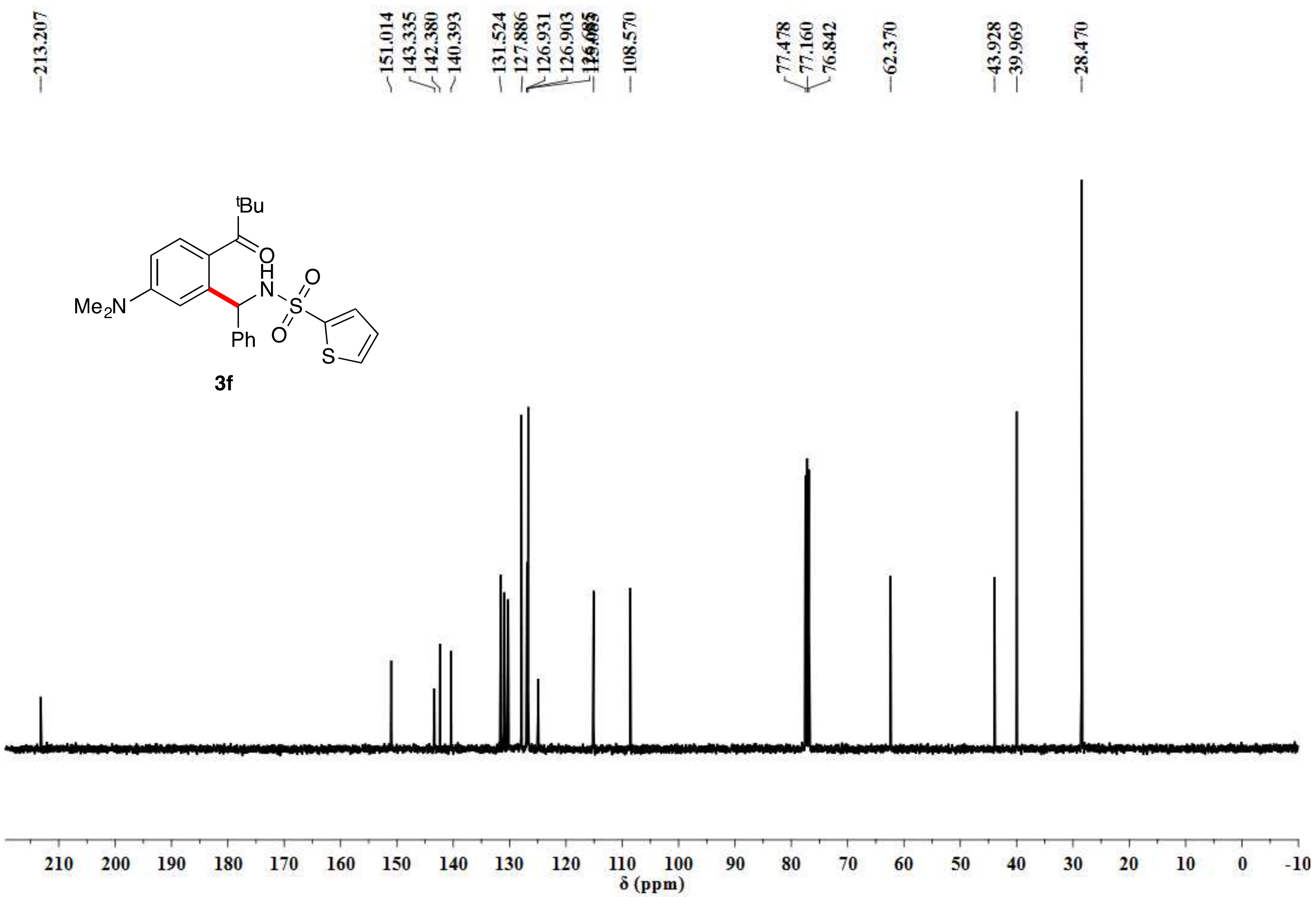

213.207
151.014
143.335
142.380
140.393
131.524
127.886
126.931
126.903
108.570
77.478
77.160
76.842
62.370
43.928
39.969
28.470
tBu
O
H
N
O
S
O
Me2N
Ph
S
3f
210 200 190 180 170 160 150 140 130 120 110 100 90 80 70 60 50 40 30 20 10 0 -10
δ (ppm)

Supplementary Figure 35. Characterization of product 3g. (a) ^{1}H NMR spectrum. (b) ^{13}C-NMR spectrum. (c) ^{19}F-NMR spectrum.

a

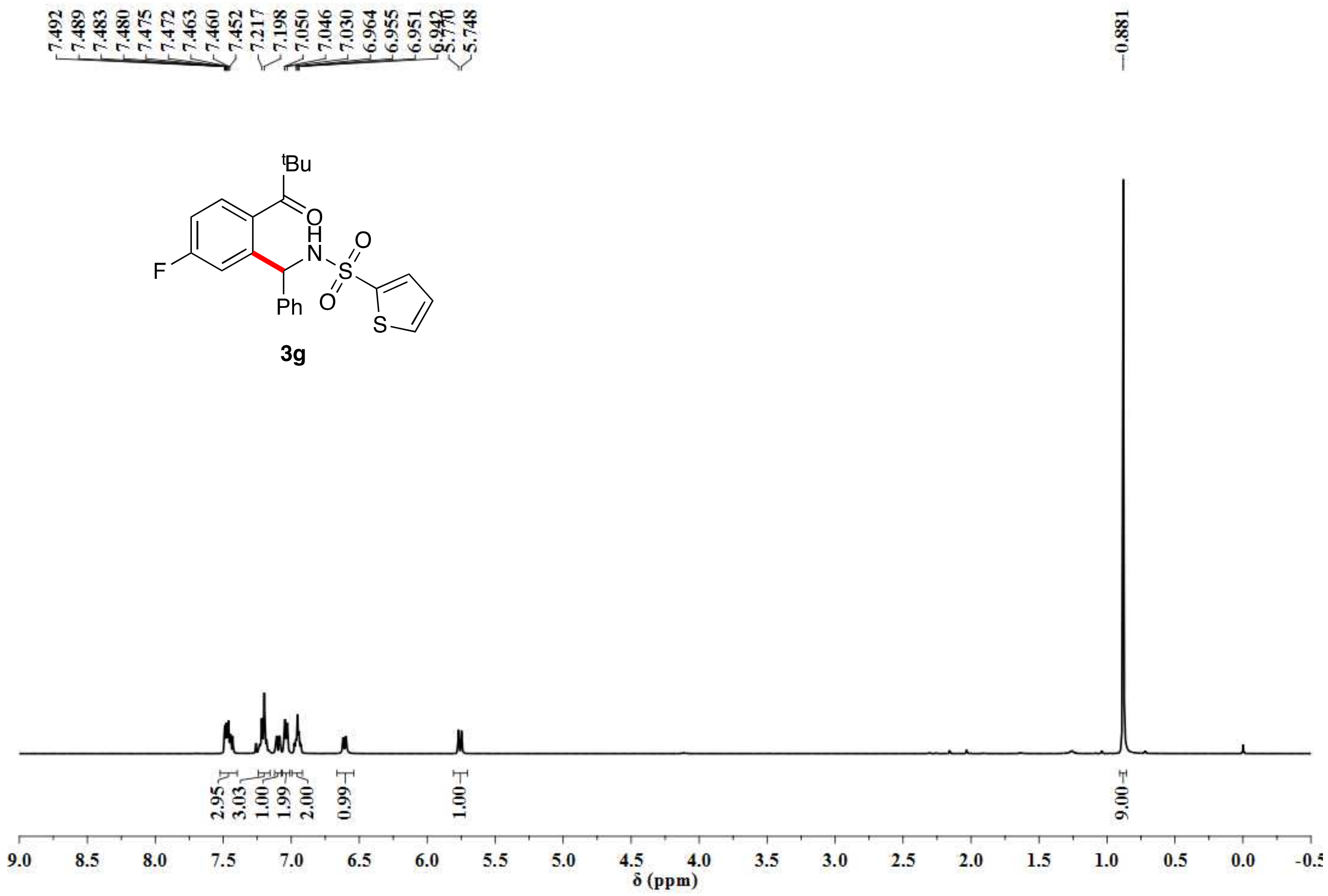

b

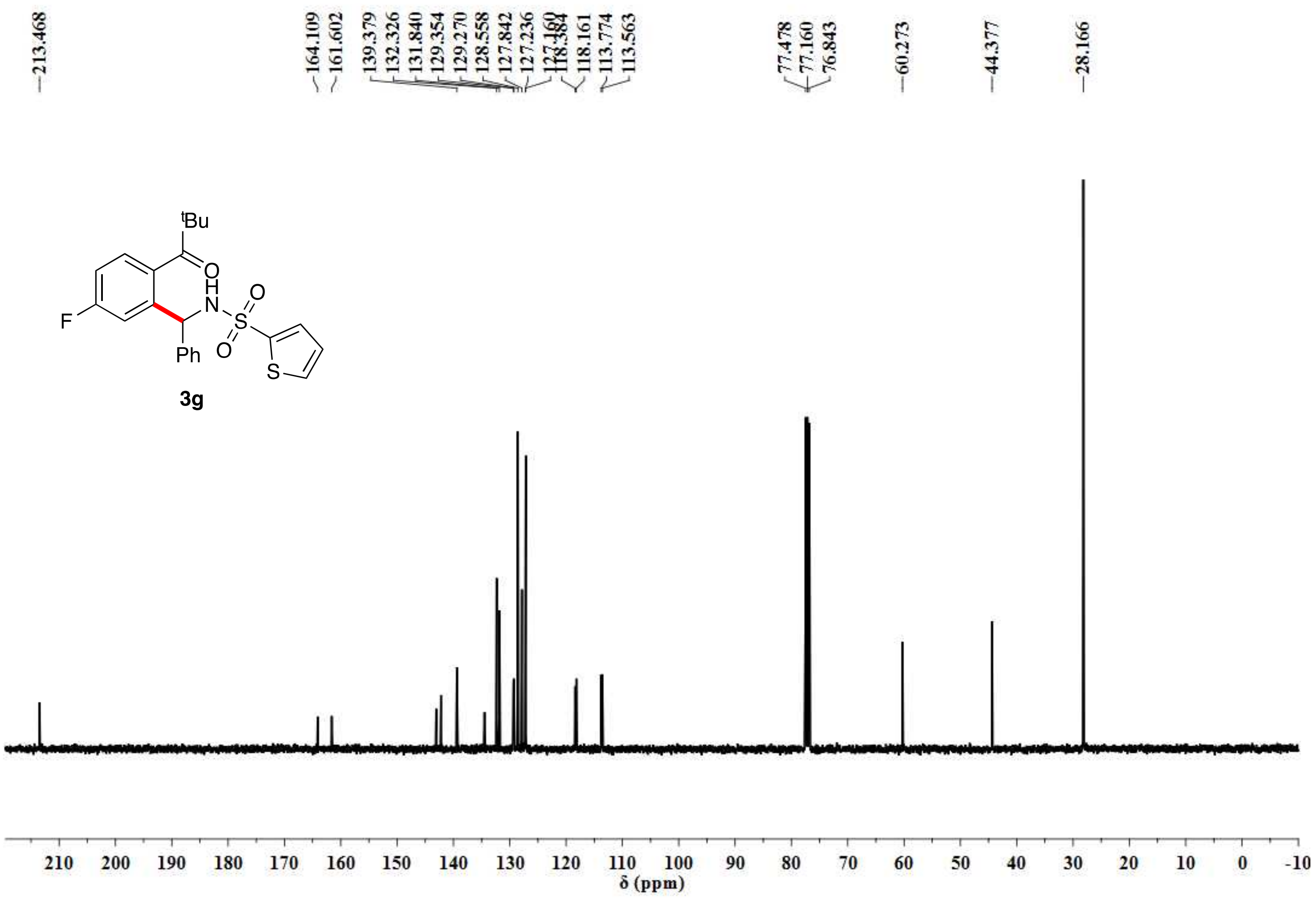

213.468
164.109
161.602
139.379
132.326
131.840
129.354
129.270
128.558
127.842
127.236
127.160
118.384
118.161
113.774
113.563
77.478
77.160
76.843
60.273
44.377
28.166
tBu
O
H
N
O
S
O
F
Ph
S
3g
210
200
190
180
170
160
150
140
130
120
110
100
90
80
70
60
50
40
30
20
10
0
-10
δ (ppm)

c

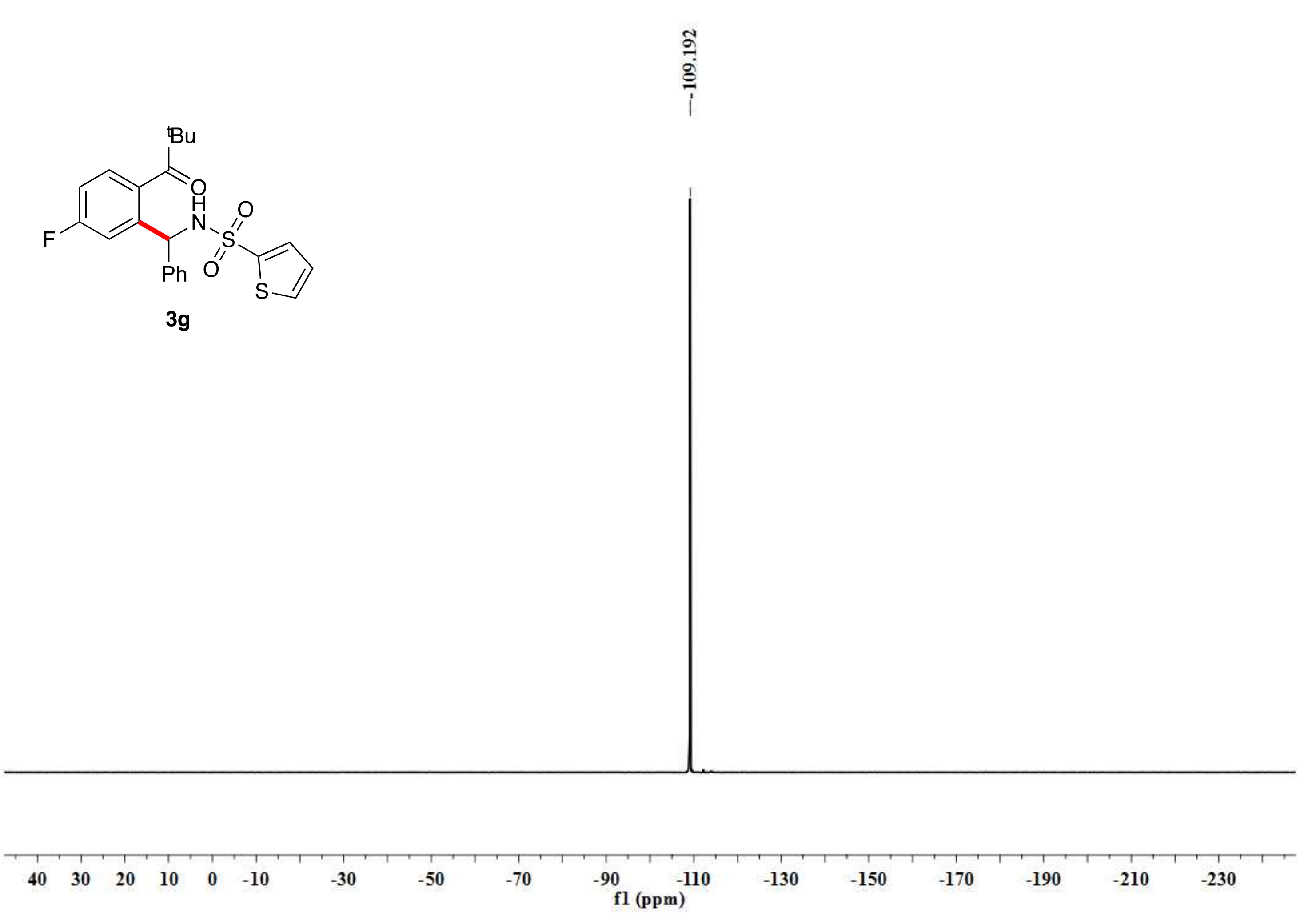

-109.192
tBu
O
H
N
O
S
O
F
Ph
S
3g
40 30 20 10 0 -10 -30 -50 -70 -90 -110 -130 -150 -170 -190 -210 -230
f1 (ppm)

Supplementary Figure 36. Characterization of product 3h. (a) ^{1}H NMR spectrum. (b) ^{13}C-NMR spectrum.

a

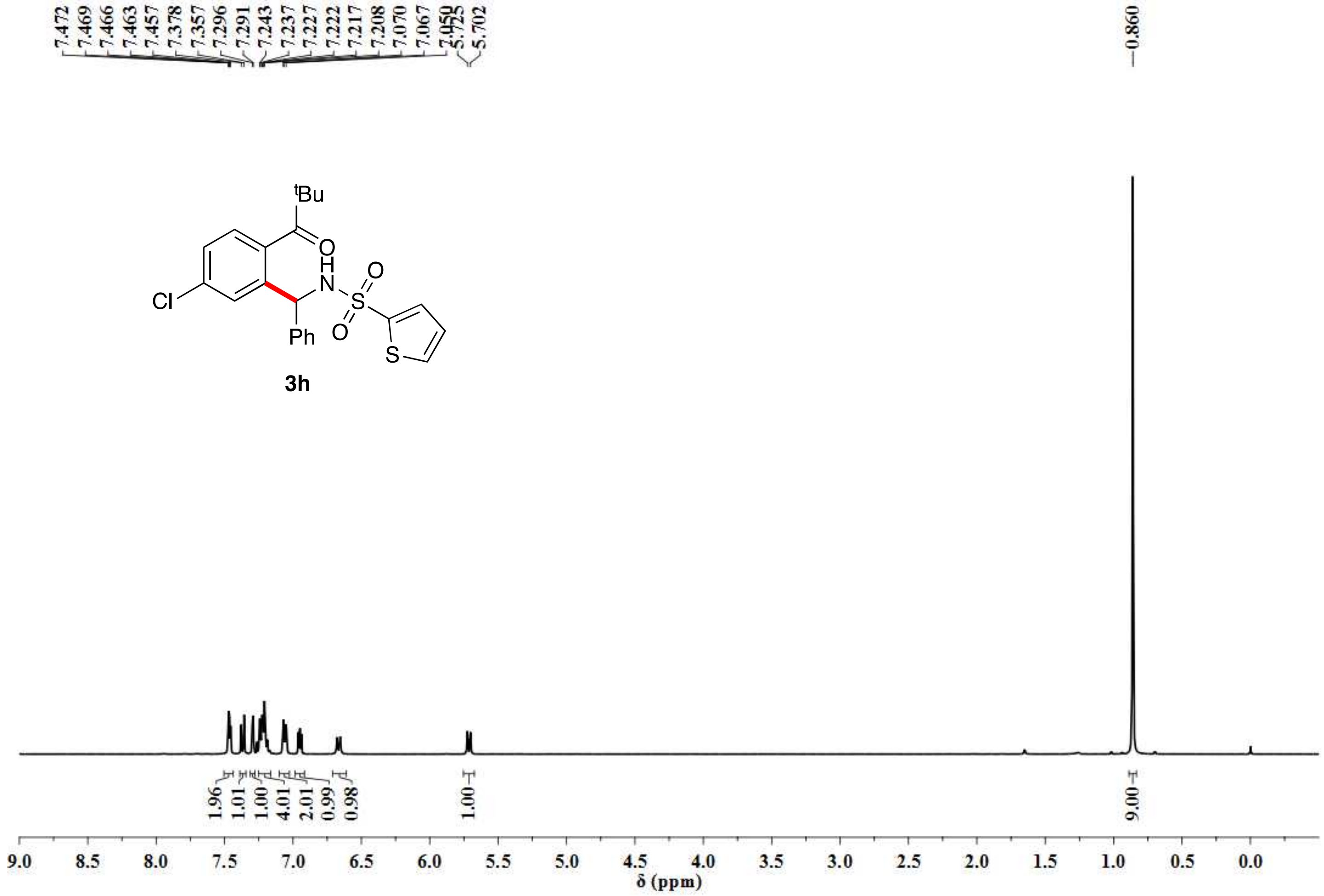

b

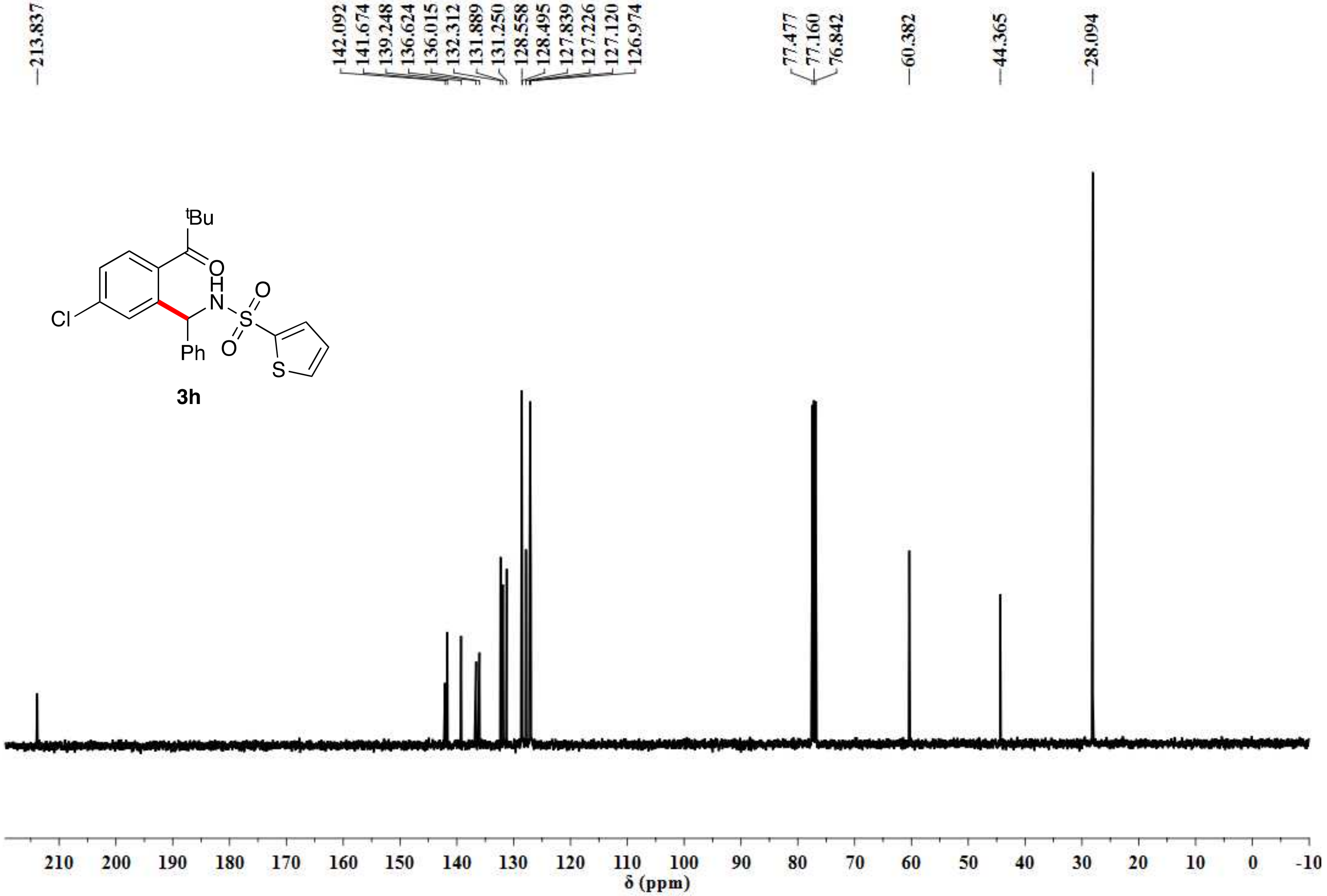
3h
213.837
142.092
141.674
139.248
136.624
136.015
132.312
131.889
131.250
128.558
128.495
127.839
127.226
127.120
126.974
77.477
77.160
76.842
60.382
44.365
28.094
210 200 190 180 170 160 150 140 130 120 110 100 90 80 70 60 50 40 30 20 10 0 -10
δ (ppm)

Supplementary Figure 37. Characterization of product 3i. (a) ^{1}H NMR spectrum. (b) ^{13}C-NMR spectrum.

a

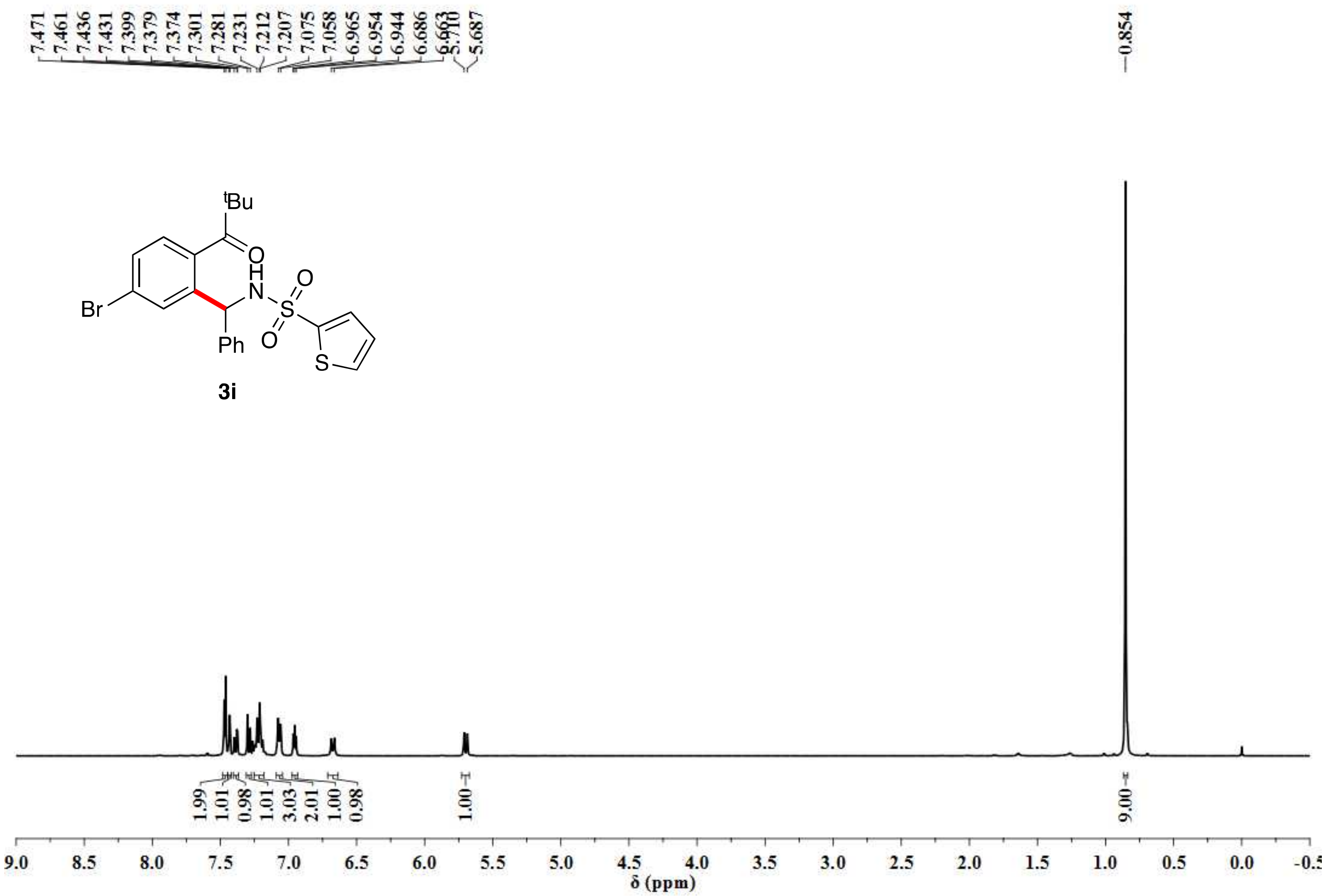

b

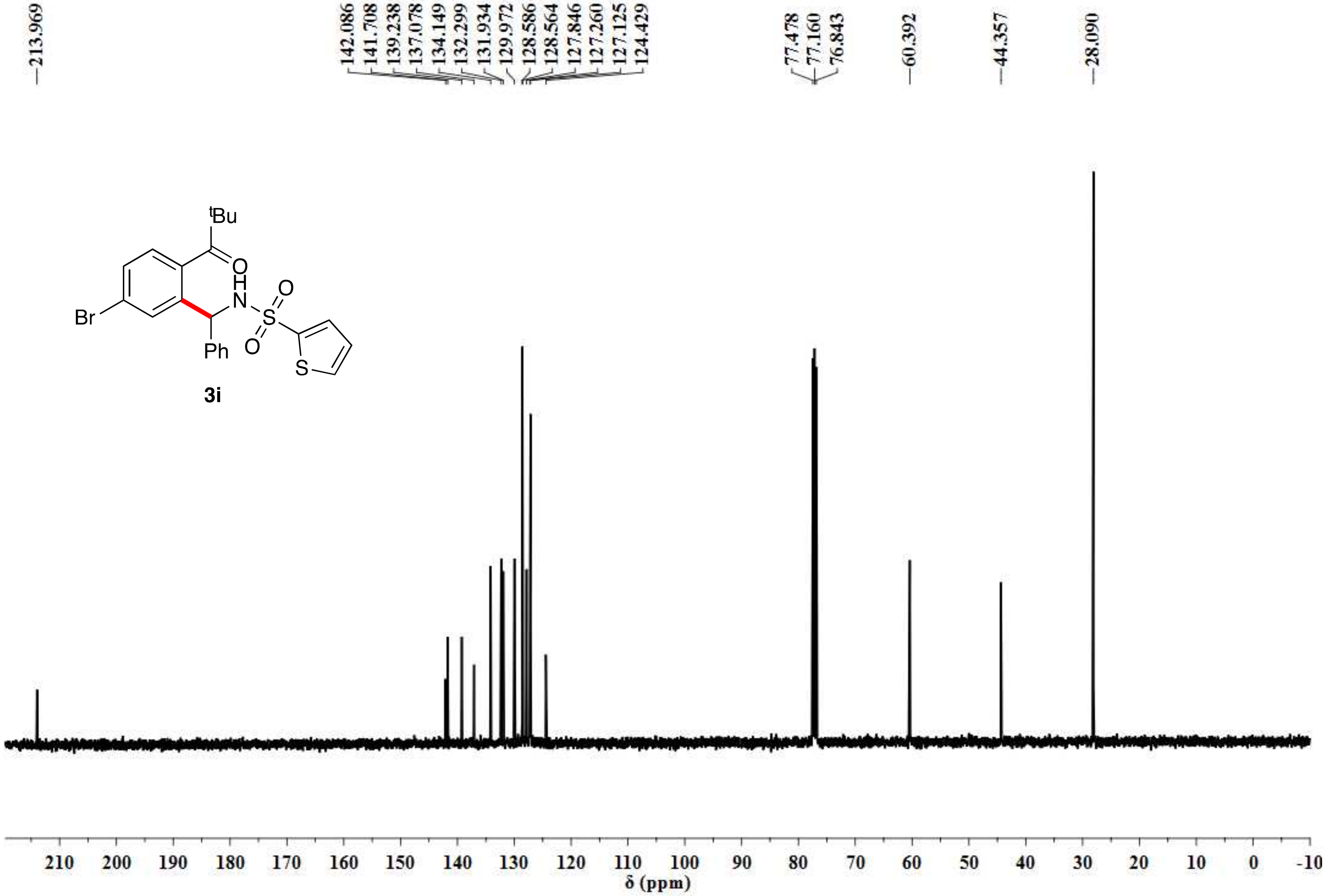

213.969
142.086
141.708
139.238
137.078
134.149
132.299
131.934
129.972
128.586
128.564
127.846
127.260
127.125
124.429
77.478
77.160
76.843
60.392
44.357
28.090
tBu
O
Br
H
N
S
O
O
Ph
S
3i
210 200 190 180 170 160 150 140 130 120 110 100 90 80 70 60 50 40 30 20 10 0 -10
δ (ppm)

Supplementary Figure 38. Characterization of product 3j. (a) ^{1}H NMR spectrum. (b) ^{13}C-NMR spectrum.

a

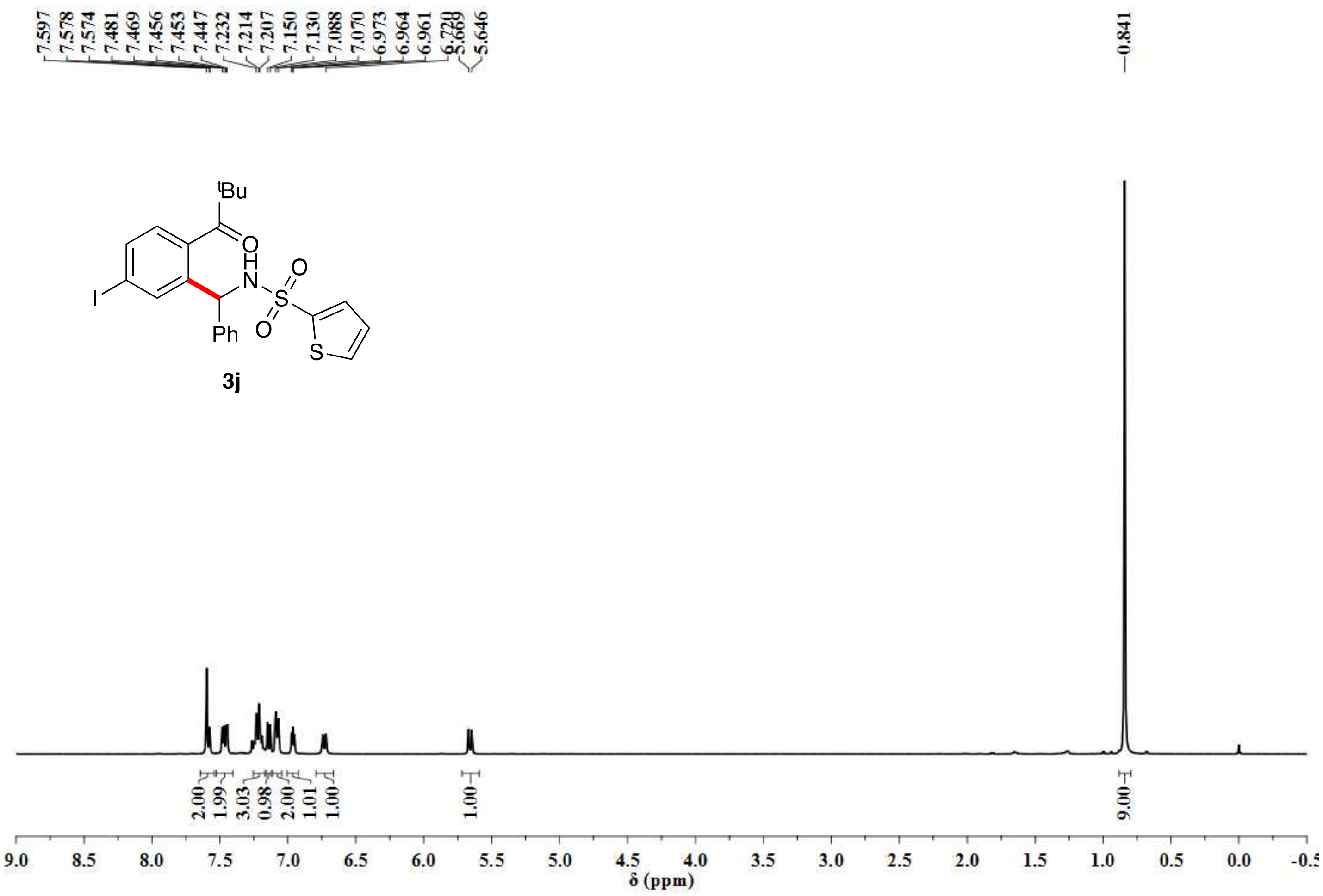

b

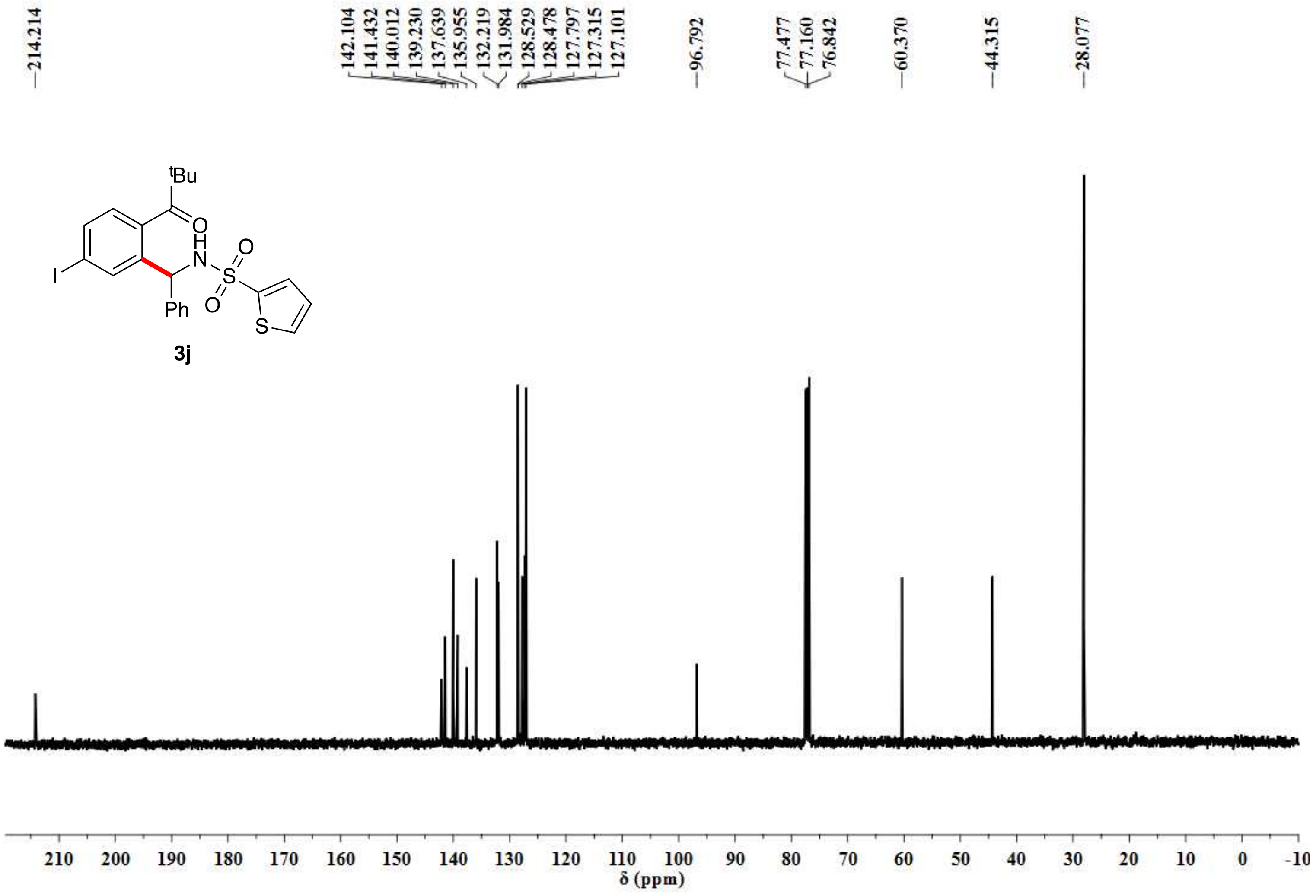
214.214
142.104
141.432
140.012
139.230
137.639
135.955
132.219
131.984
128.529
128.478
127.797
127.315
127.101
96.792
77.477
77.160
76.842
60.370
44.315
28.077
tBu
O
H
N
S
O
O
I
Ph
S
3j
210 200 190 180 170 160 150 140 130 120 110 100 90 80 70 60 50 40 30 20 10 0 -10
δ (ppm)

Supplementary Figure 39. Characterization of product 3k. (a) ^{1}H NMR spectrum. (b) ^{13}C-NMR spectrum.

a

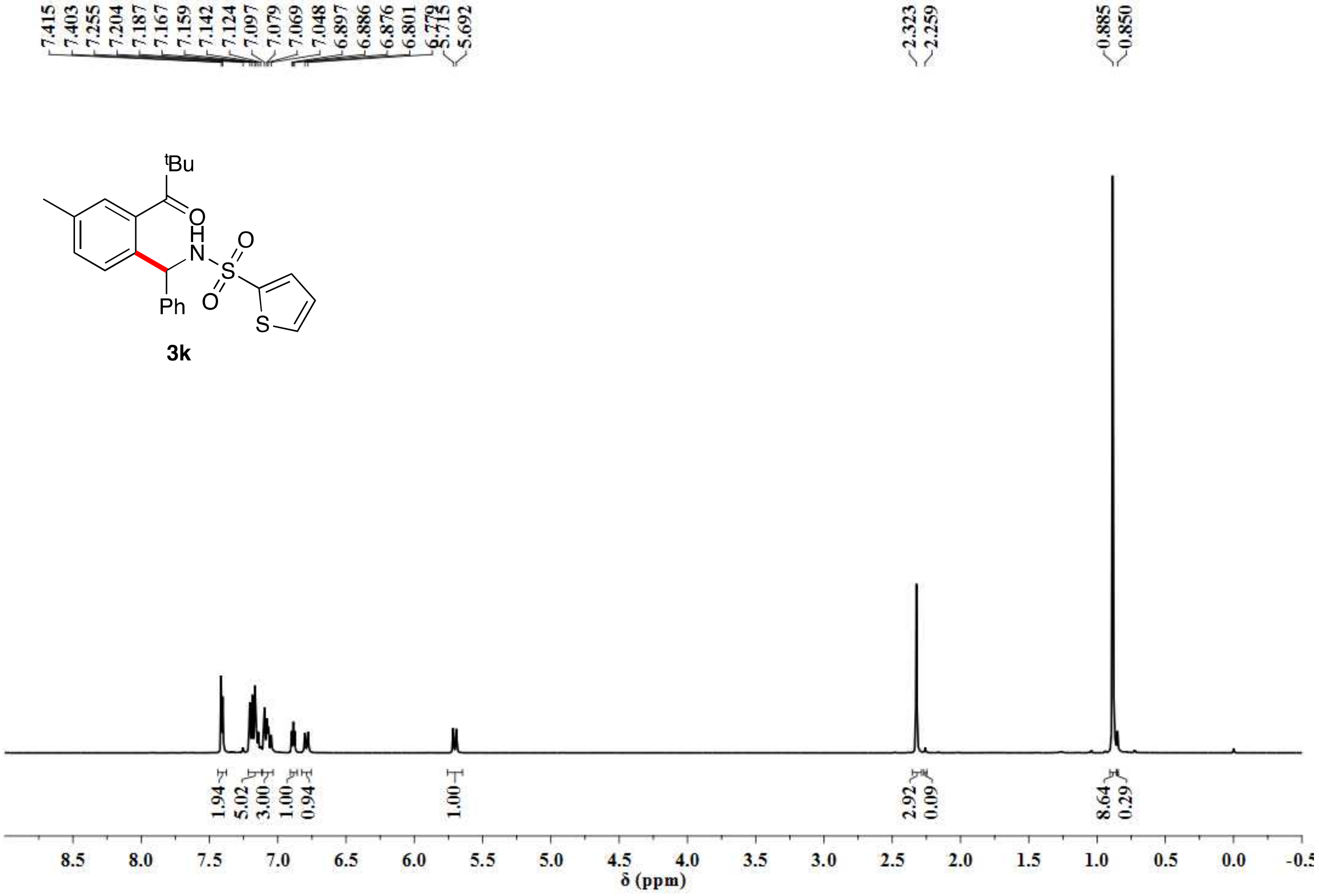

b

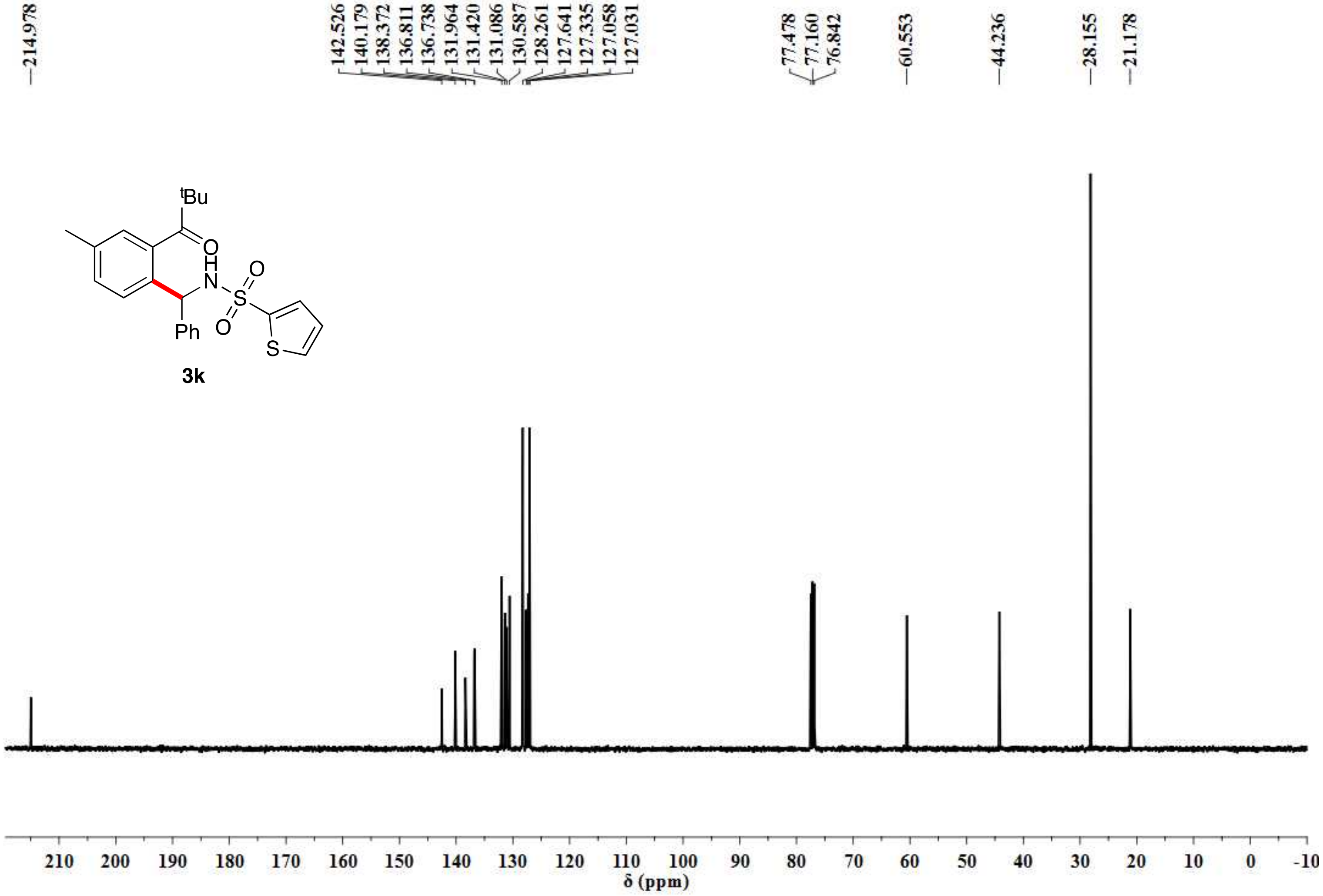

214.978
142.526
140.179
138.372
136.811
136.738
131.964
131.420
131.086
130.587
128.261
127.641
127.335
127.058
127.031
77.478
77.160
76.842
60.553
44.236
28.155
21.178
tBu
O
H
N
S
O
O
Ph
S
3k
210 200 190 180 170 160 150 140 130 120 110 100 90 80 70 60 50 40 30 20 10 0 -10
δ (ppm)

Supplementary Figure 40. Characterization of product 3l. (a) ^{1}H NMR spectrum. (b) ^{13}C-NMR spectrum.

a

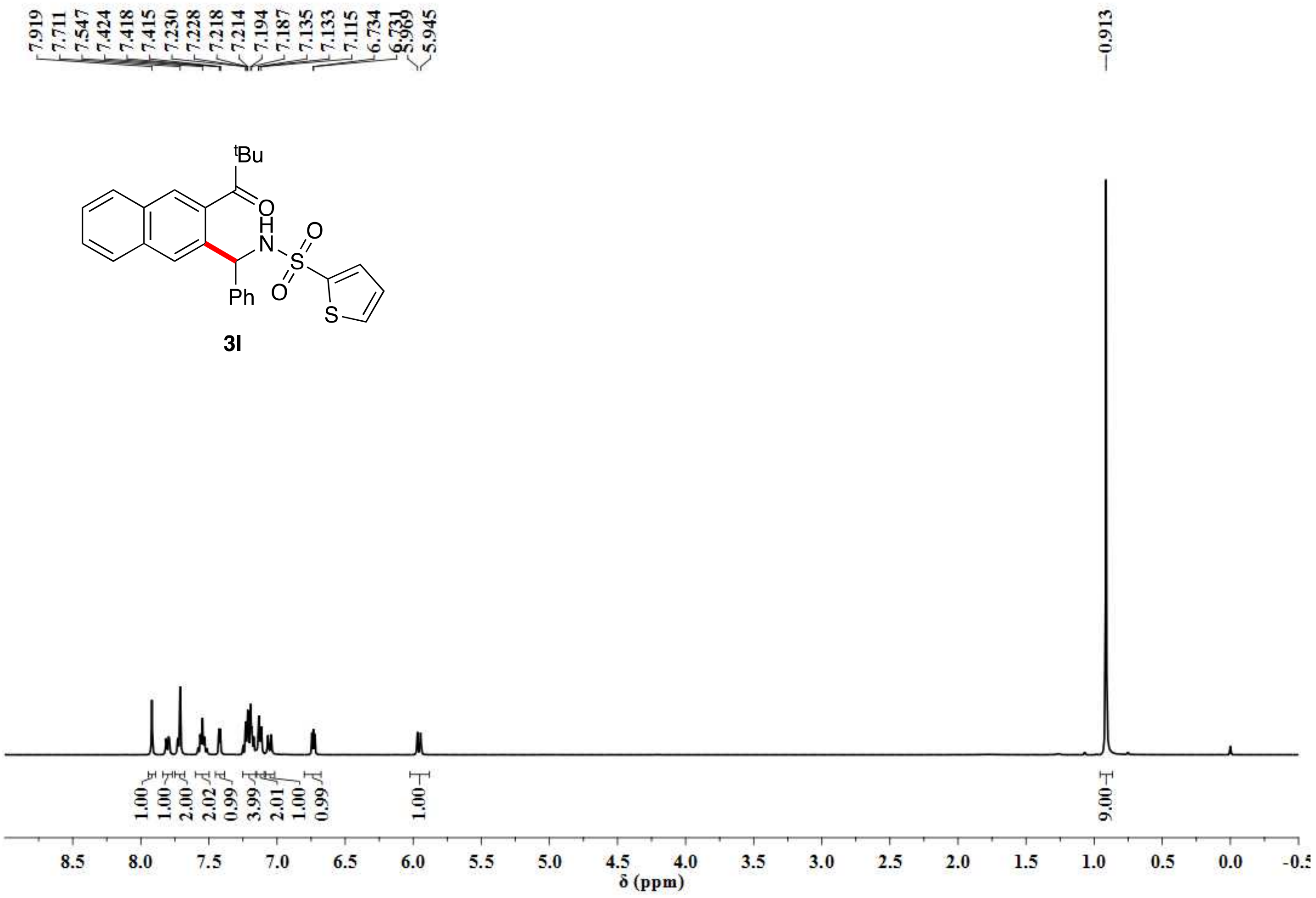

b

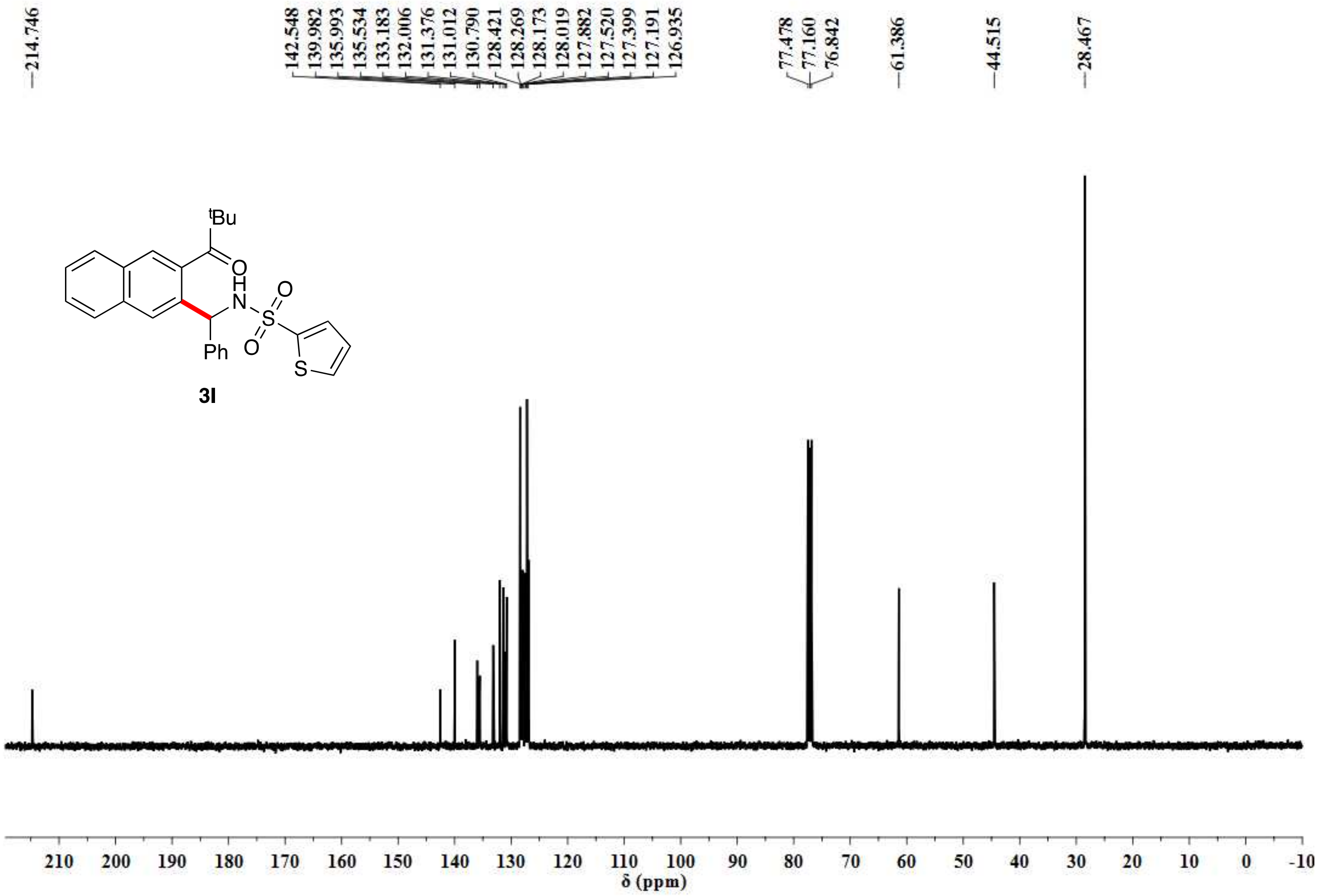
214.746
142.548
139.982
135.993
135.534
133.183
132.006
131.376
131.012
130.790
128.421
128.269
128.173
128.019
127.882
127.520
127.399
127.191
126.935
77.478
77.160
76.842
61.386
44.515
28.467
tBu
O
H
N
S
O
O
Ph
S
3l
210 200 190 180 170 160 150 140 130 120 110 100 90 80 70 60 50 40 30 20 10 0 -10
δ (ppm)

Supplementary Figure 41. Characterization of product 3m. (a) ^{1}H NMR spectrum. (b) ^{13}C-NMR spectrum.

a

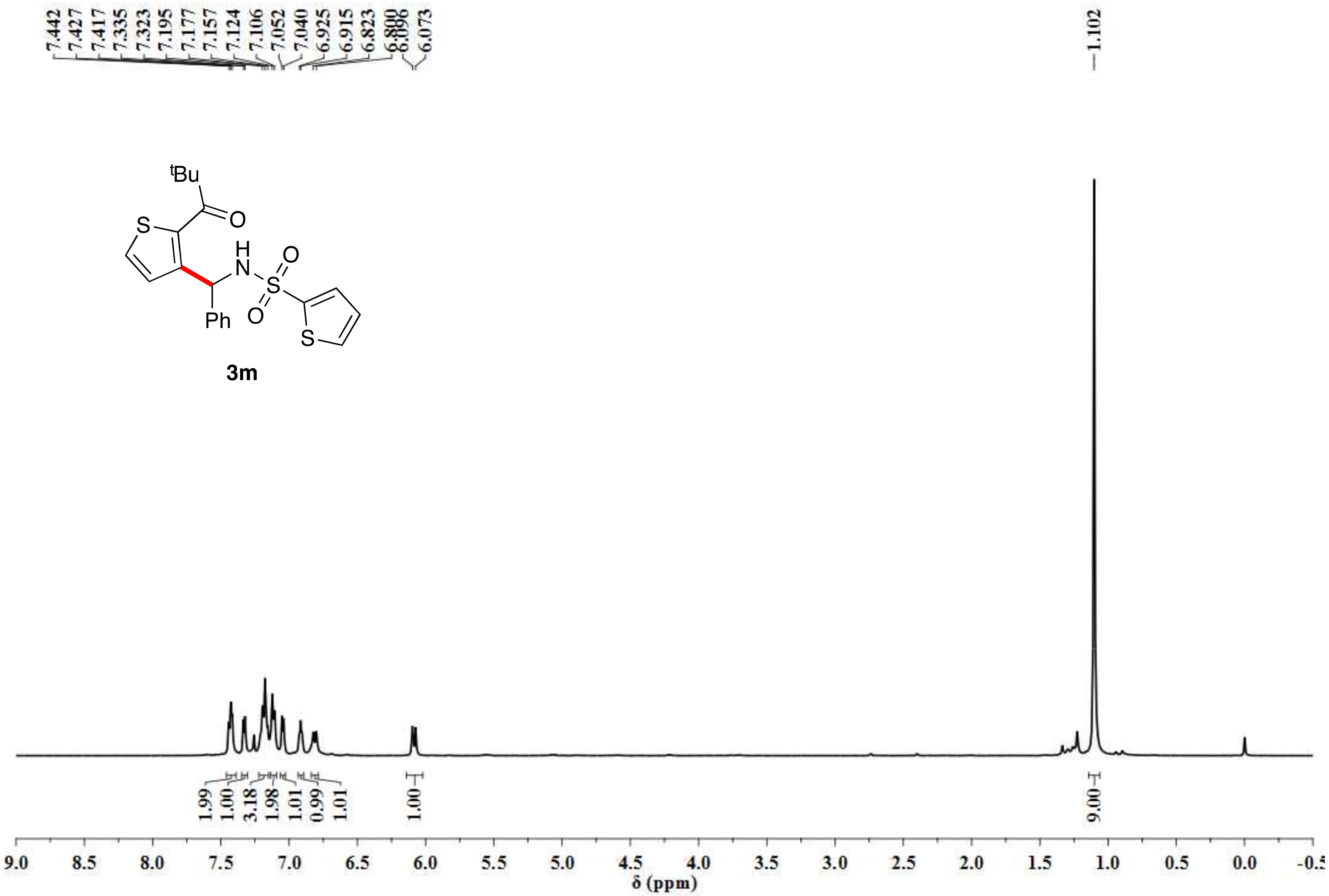

b

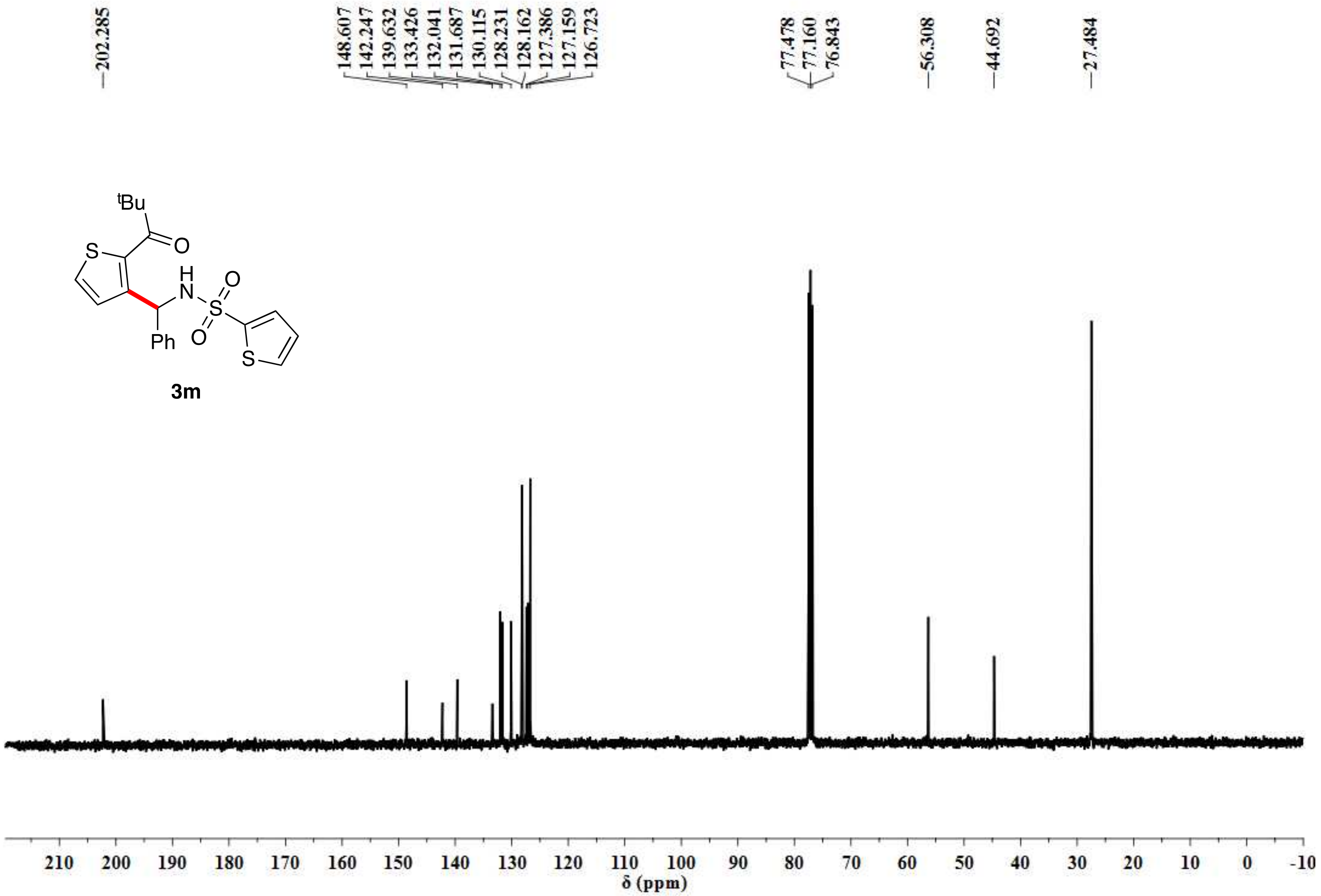
202.285
148.607
142.247
139.632
133.426
132.041
131.687
130.115
128.231
128.162
127.386
127.159
126.723
77.478
77.160
76.843
56.308
44.692
27.484
tBu
S
O
H
N
S
O
O
Ph
S
3m
210 200 190 180 170 160 150 140 130 120 110 100 90 80 70 60 50 40 30 20 10 0 -10
δ (ppm)

Supplementary Figure 42. Characterization of product 3n. (a) ^{1}H NMR spectrum. (b) ^{13}C-NMR spectrum.

a

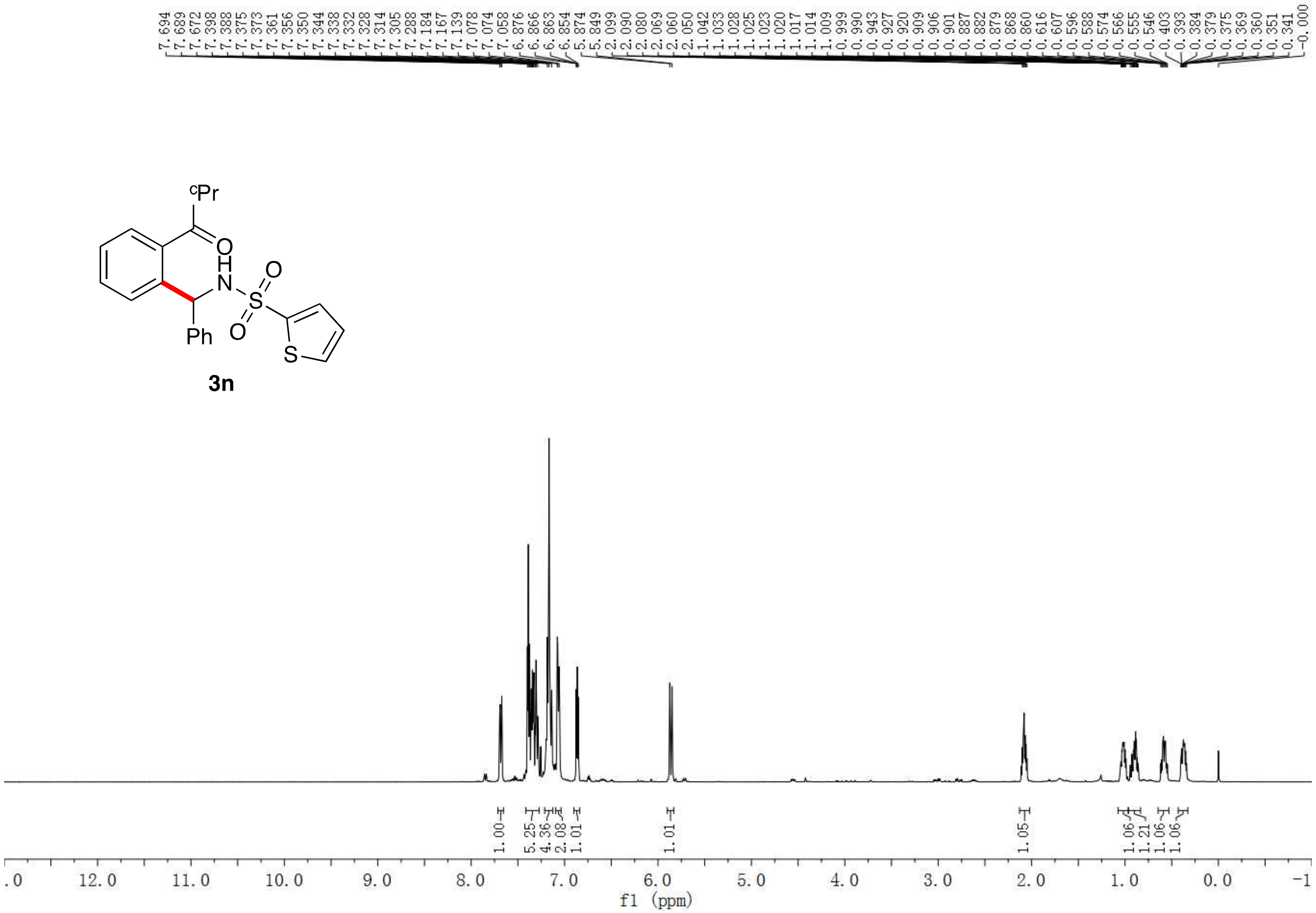

b

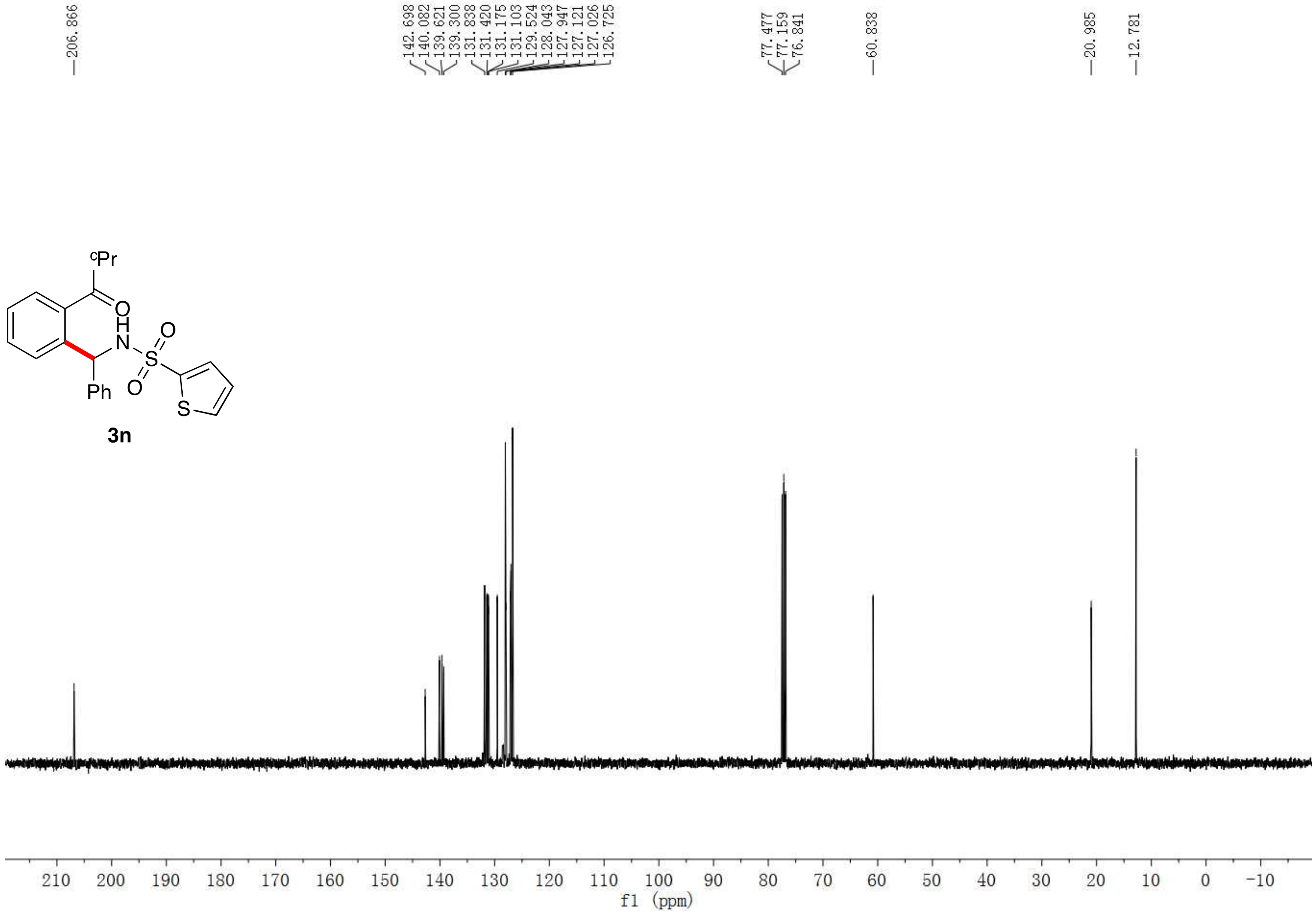
206.866
142.698
140.082
139.621
139.300
131.838
131.420
131.175
131.103
129.524
128.043
127.947
127.121
127.026
126.725
77.477
77.159
76.841
60.838
20.985
12.781
cPr
O
H
N
O
S
O
S
Ph
3n
210 200 190 180 170 160 150 140 130 120 110 100 90 80 70 60 50 40 30 20 10 0 −10
f1 (ppm)

Supplementary Figure 43. Characterization of product 3o. (a) ^{1}HNMR spectrum. (b) ^{13}C NMR spectrum.

a

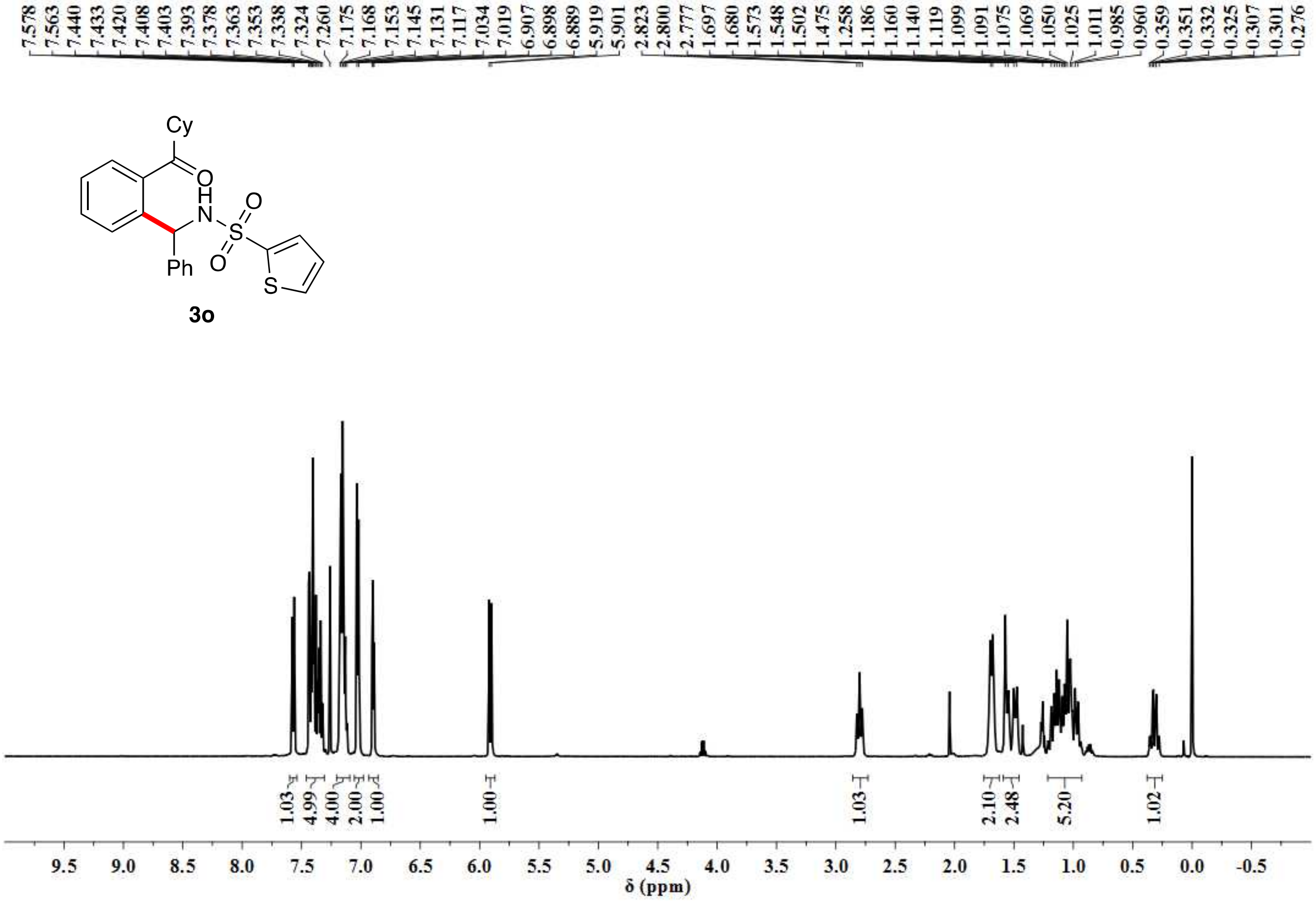

b

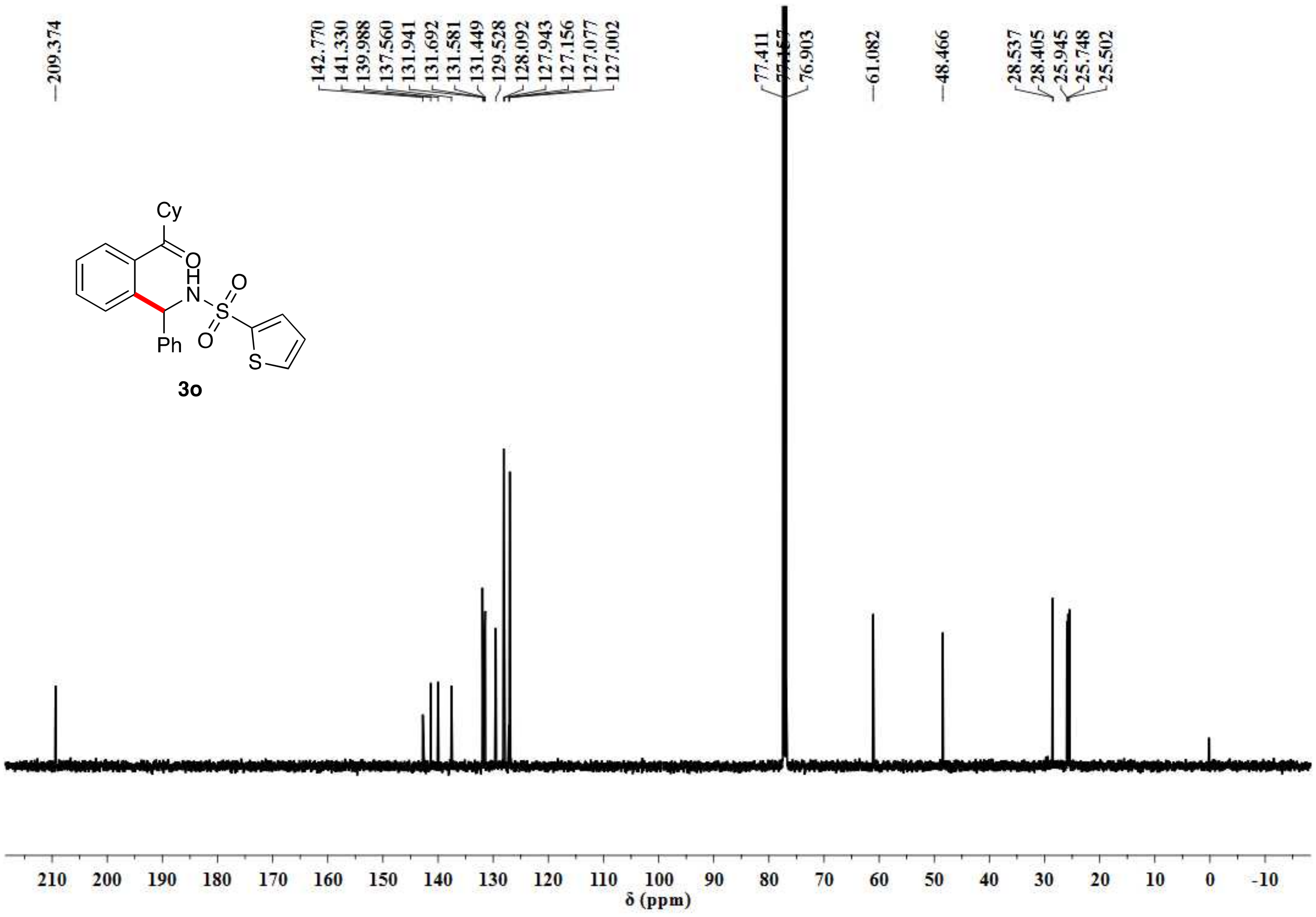

209.374
142.770
141.330
139.988
137.560
131.941
131.692
131.581
131.449
129.528
128.092
127.943
127.156
127.077
127.002
77.411
77.157
76.903
61.082
48.466
28.537
28.405
25.945
25.748
25.502
Cy
O
H
N
S
O
O
Ph
S
3o
210
200
190
180
170
160
150
140
130
120
110
100
90
80
70
60
50
40
30
20
10
0
-10
δ (ppm)

Supplementary Figure 44. Characterization of product 3p. (a) ^{1}HNMR spectrum. (b) ^{13}C NMR spectrum.

a

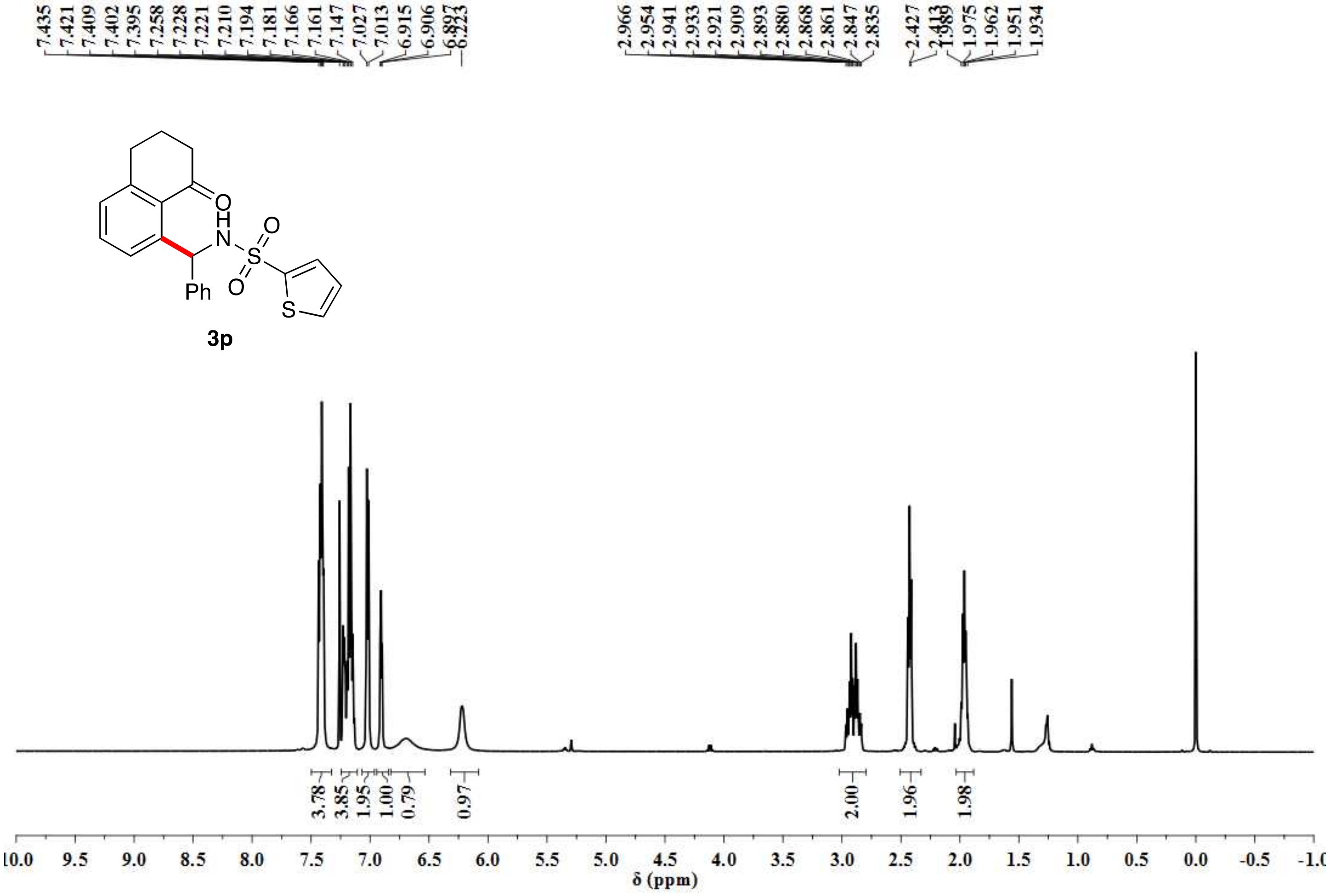

b

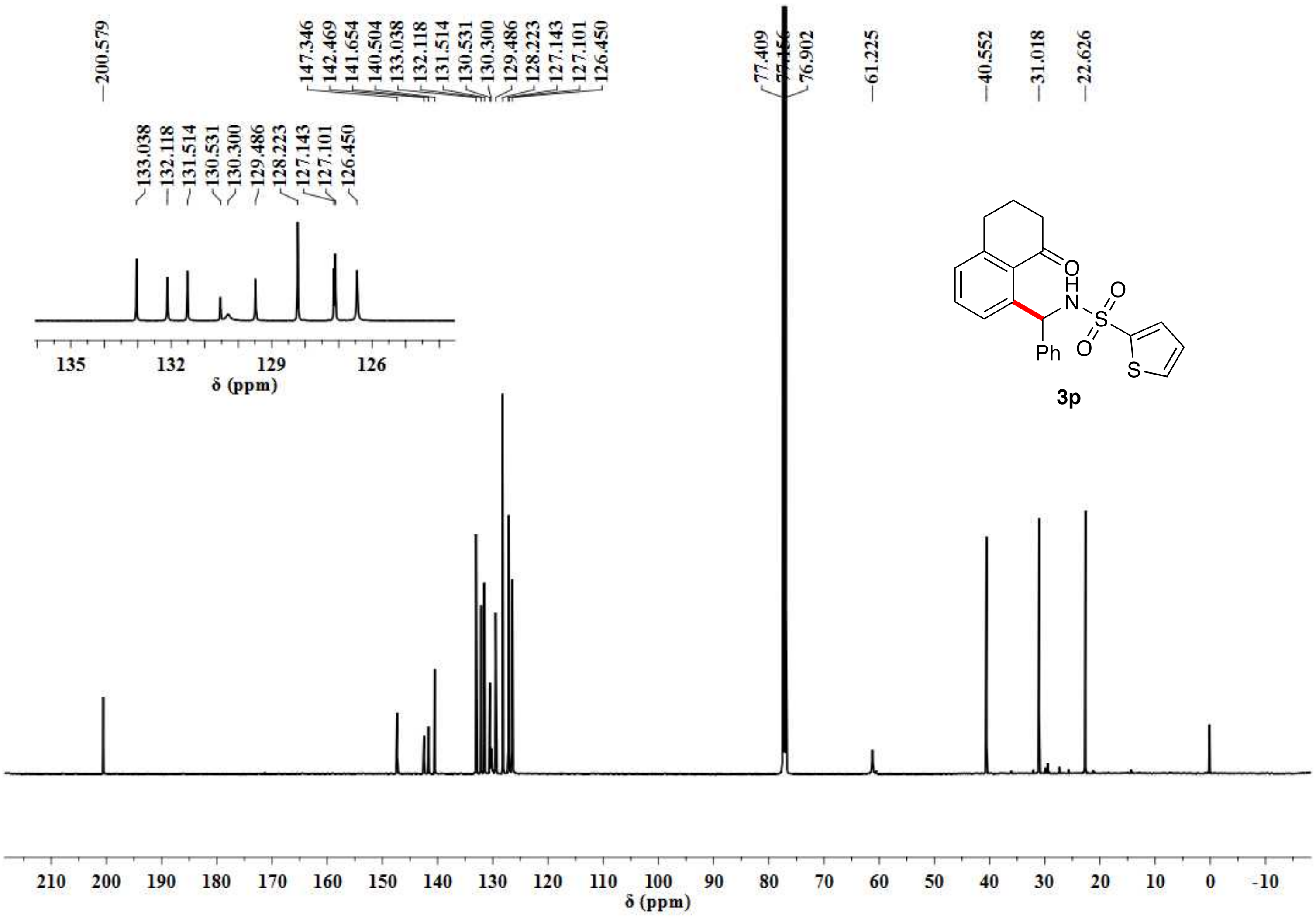
200.579
147.346
142.469
141.654
140.504
133.038
132.118
131.514
130.531
130.300
129.486
128.223
127.143
127.101
126.450
77.409
76.902
61.225
40.552
31.018
22.626
135
132
129
126
δ (ppm)
O
H
N
S
O
O
Ph
S
3p
210
200
190
180
170
160
150
140
130
120
110
100
90
80
70
60
50
40
30
20
10
0
-10
δ (ppm)

Supplementary Figure 45. Characterization of product 3q. (a) ^{1}HNMR spectrum. (b) ^{13}C NMR spectrum.

a

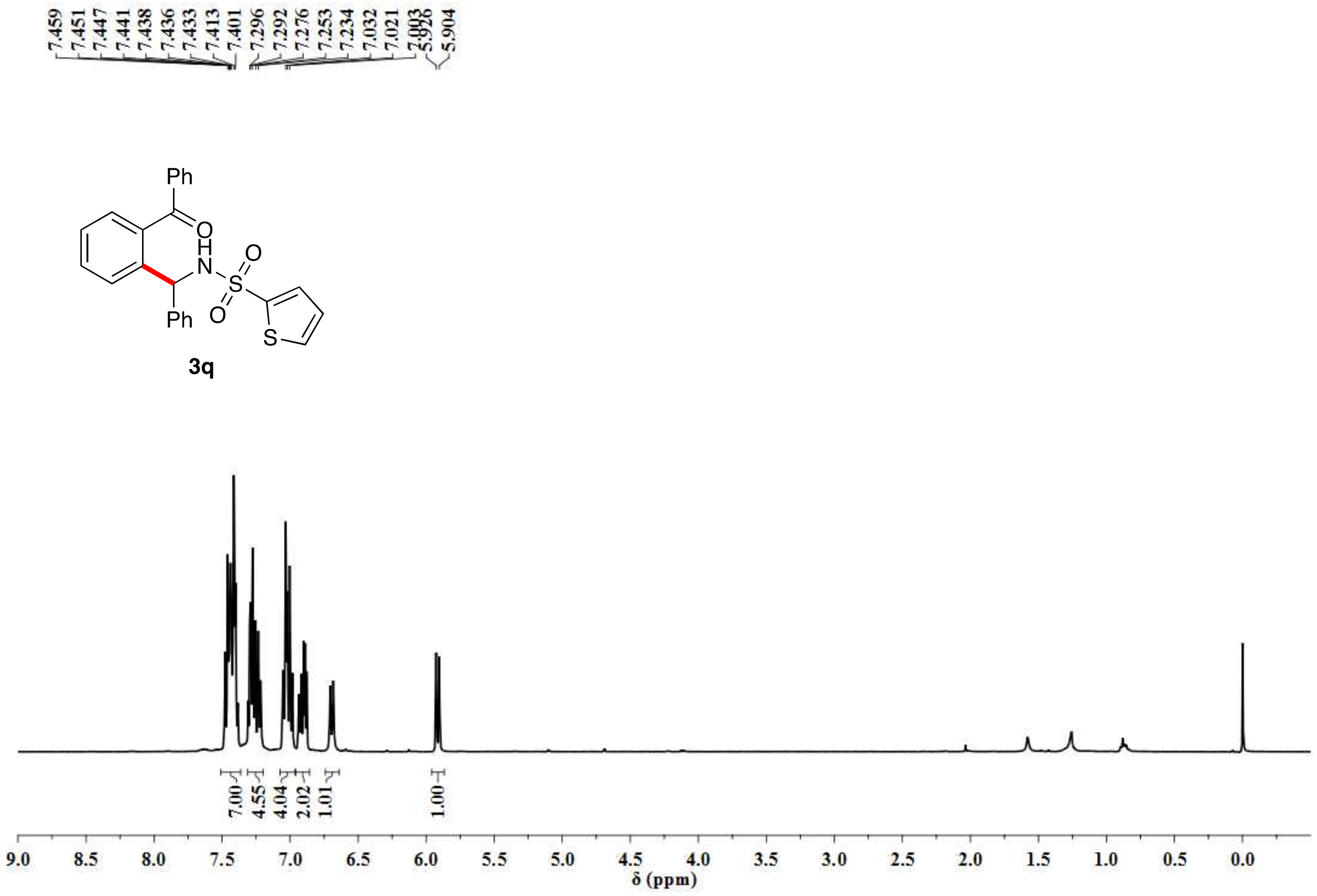

b

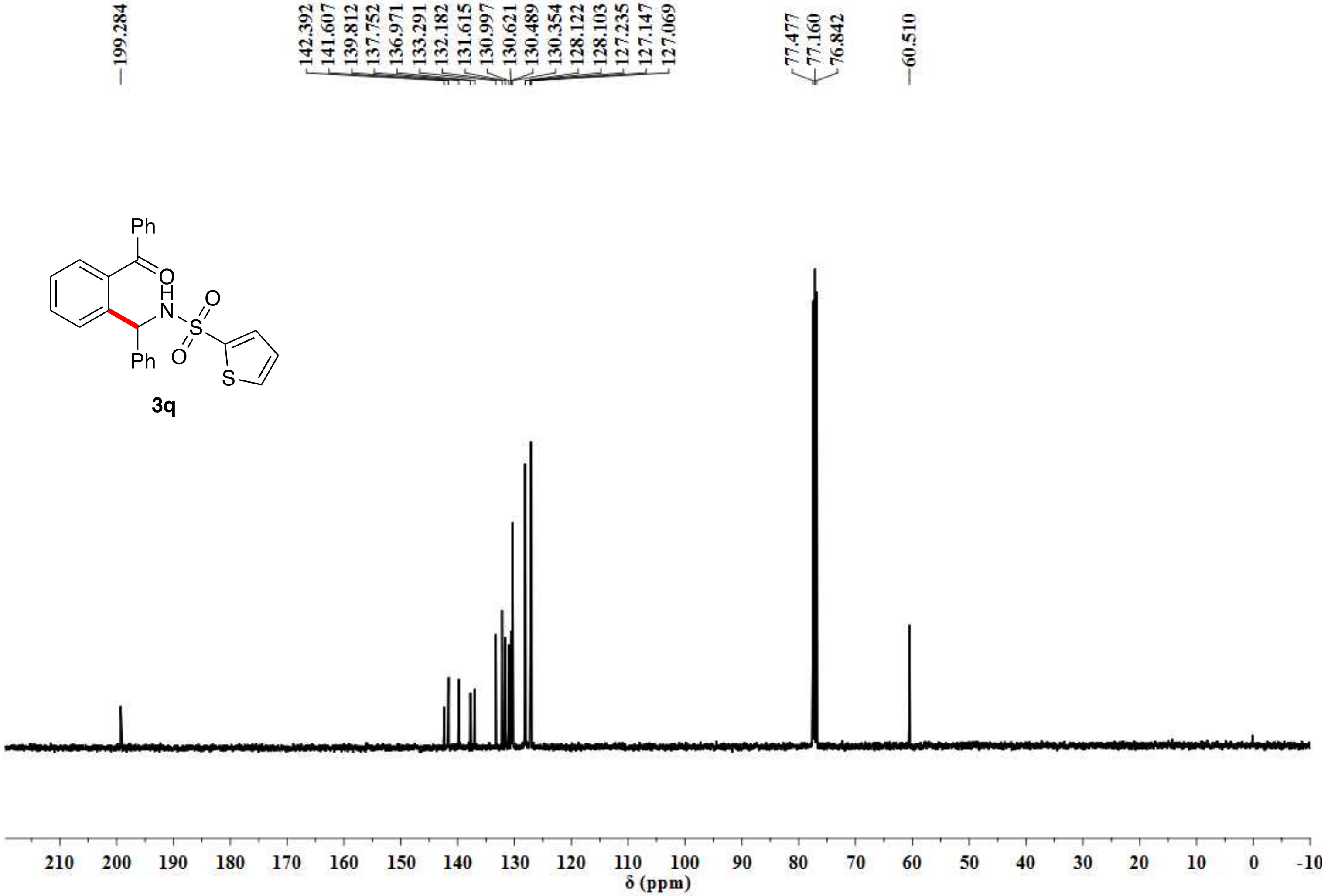
3q
199.284
142.392
141.607
139.812
137.752
136.971
133.291
132.182
131.615
130.997
130.621
130.489
130.354
128.122
128.103
127.235
127.147
127.069
77.477
77.160
76.842
60.510
210 200 190 180 170 160 150 140 130 120 110 100 90 80 70 60 50 40 30 20 10 0 -10
δ (ppm)

Supplementary Figure 46. Characterization of product 3r. (a) ^{1}HNMR spectrum. (b) ^{13}C NMR spectrum.

a

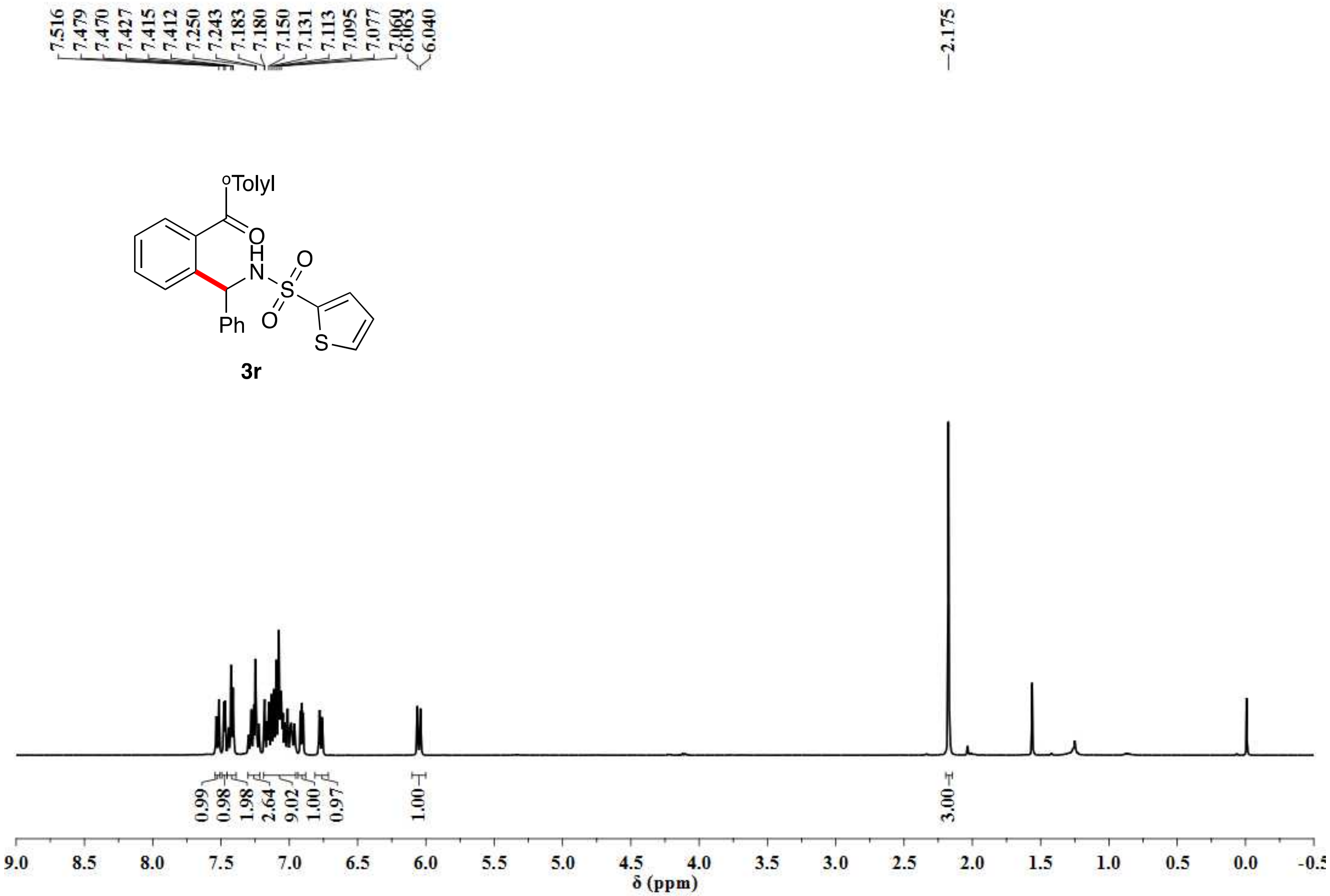

b

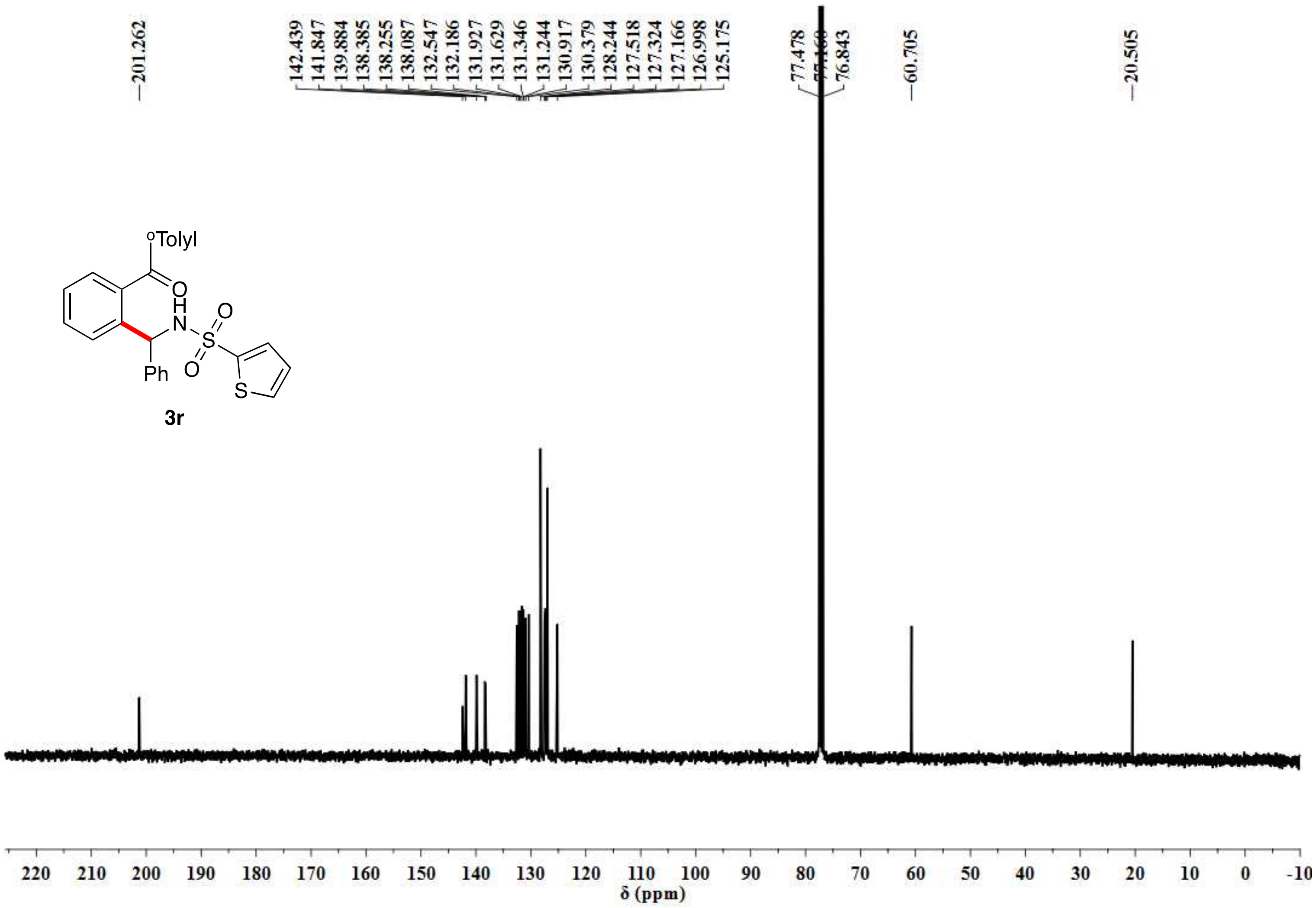

201.262
142.439
141.847
139.884
138.385
138.255
138.087
132.547
132.186
131.927
131.629
131.346
131.244
130.917
130.379
128.244
127.518
127.324
127.166
126.998
125.175
77.478
77.160
76.843
60.705
20.505
oTolyl
O
H
N
S
O
O
S
Ph
3r
220 210 200 190 180 170 160 150 140 130 120 110 100 90 80 70 60 50 40 30 20 10 0 -10
δ (ppm)

Supplementary Figure 47. Characterization of product 3s. (a) ^{1}HNMR spectrum. (b) ^{13}C NMR spectrum.

a

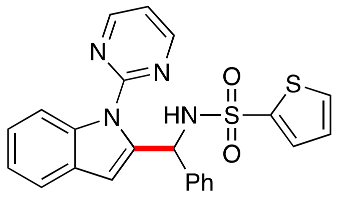

3s

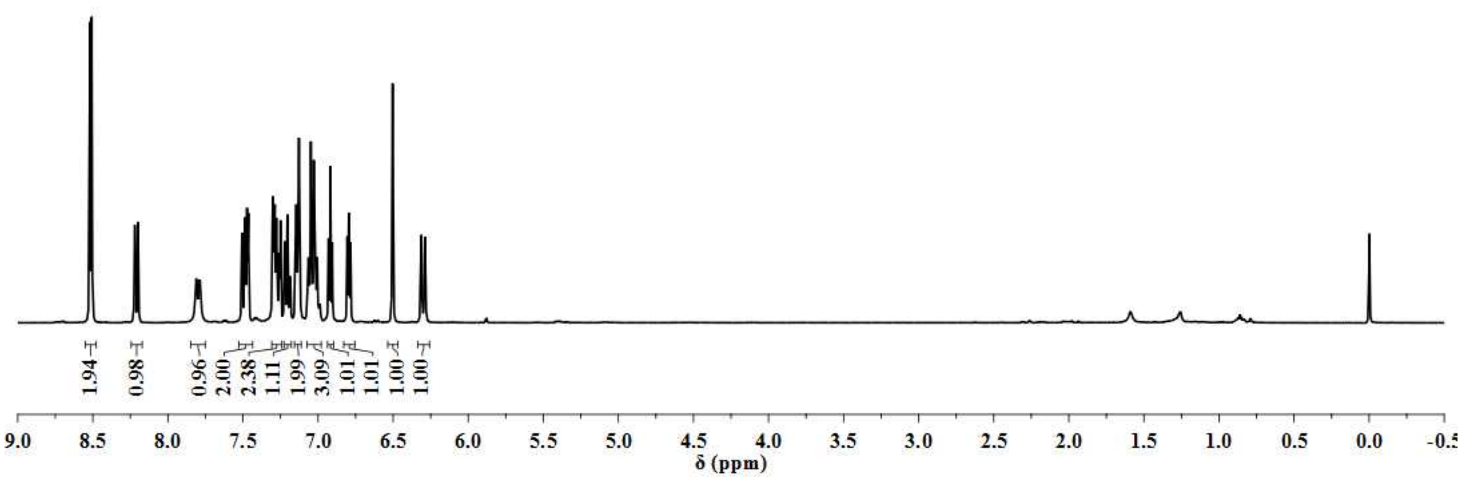

b

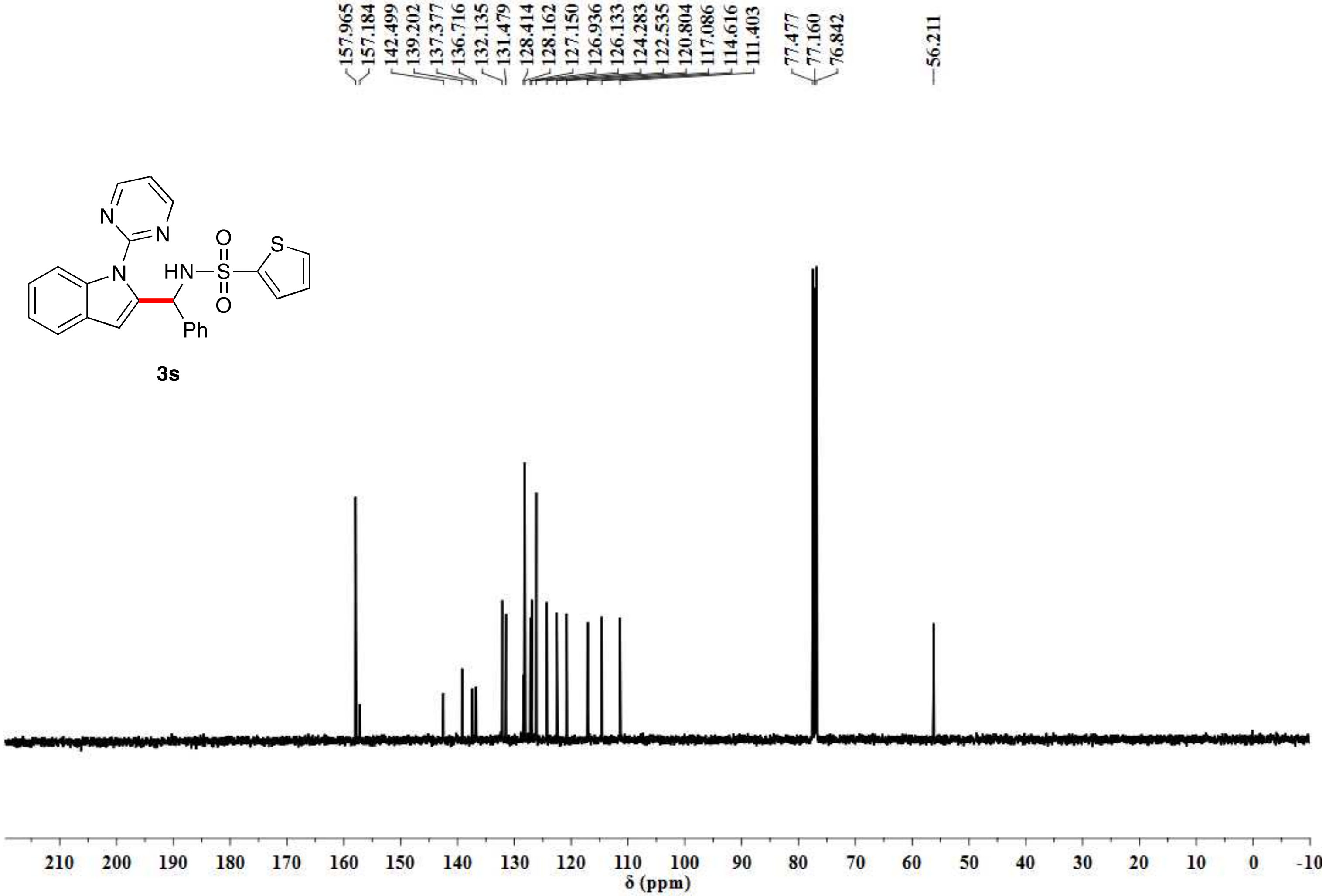
N
N
N
HN
S
O
O
S
Ph
3s
157.965
157.184
142.499
139.202
137.377
136.716
132.135
131.479
128.414
128.162
127.150
126.936
126.133
124.283
122.535
120.804
117.086
114.616
111.403
77.477
77.160
76.842
56.211
210 200 190 180 170 160 150 140 130 120 110 100 90 80 70 60 50 40 30 20 10 0 -10
δ (ppm)

Supplementary Figure 48. Characterization of product 3t. (a) ^{1}HNMR spectrum. (b) ^{13}C NMR spectrum.

a

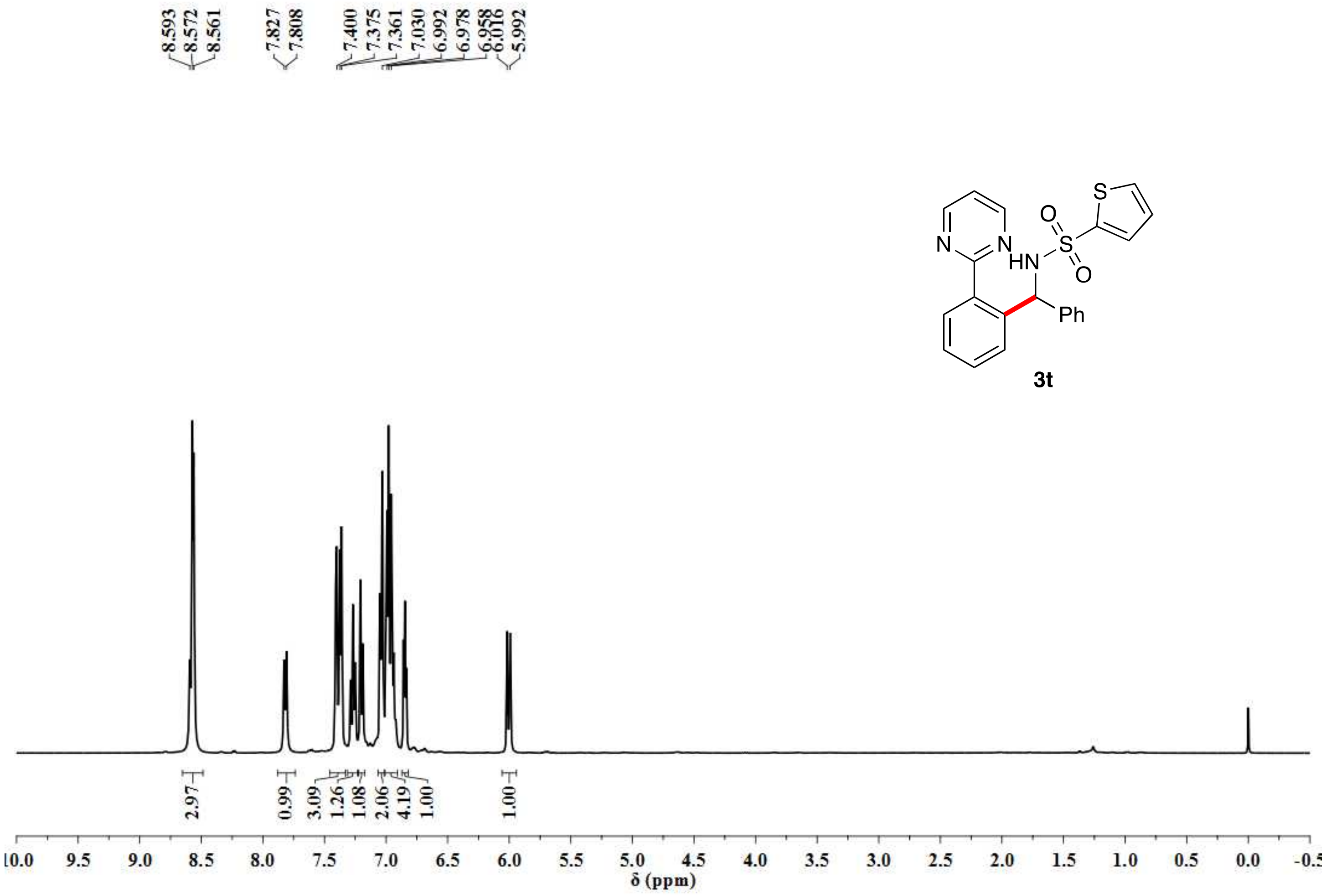

3t

b

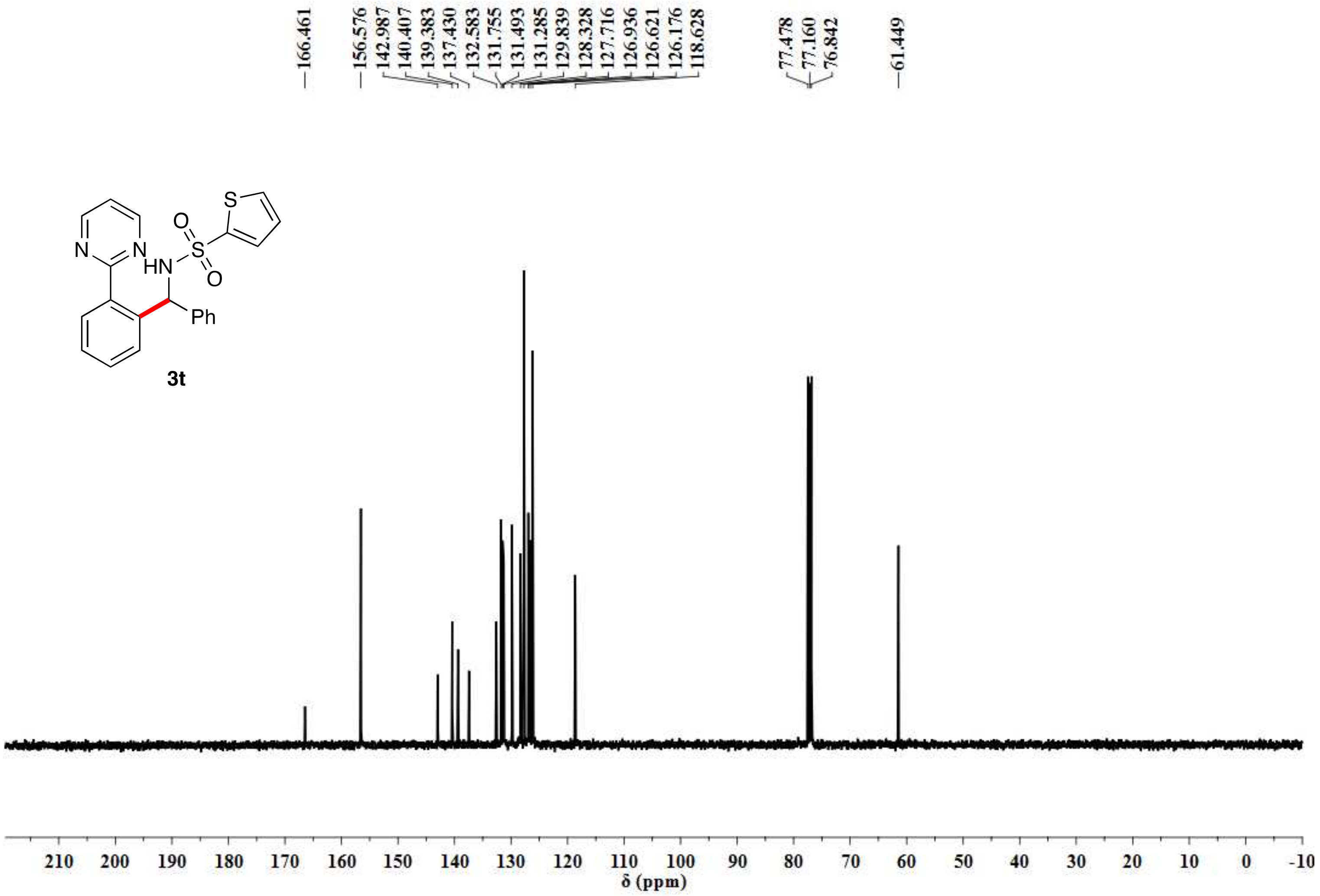
166.461
156.576
142.987
140.407
139.383
137.430
132.583
131.755
131.493
131.285
129.839
128.328
127.716
126.936
126.621
126.176
118.628
77.478
77.160
76.842
61.449
N
N
HN
S
O
O
S
Ph
3t
210 200 190 180 170 160 150 140 130 120 110 100 90 80 70 60 50 40 30 20 10 0 -10
δ (ppm)

Supplementary Figure 49. Characterization of product 3u. (a) ^{1}HNMR spectrum. (b) ^{13}C NMR spectrum.

a

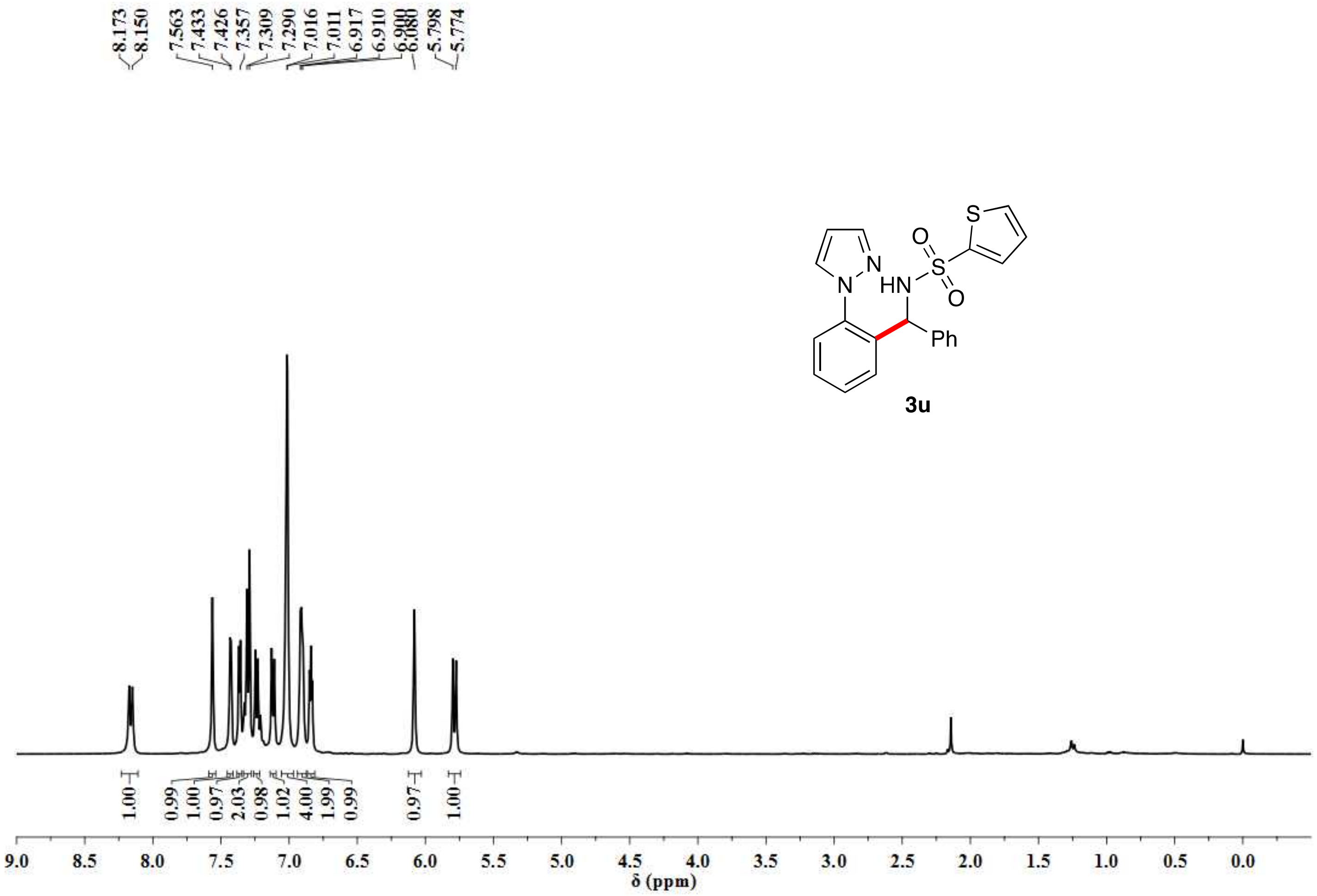

b

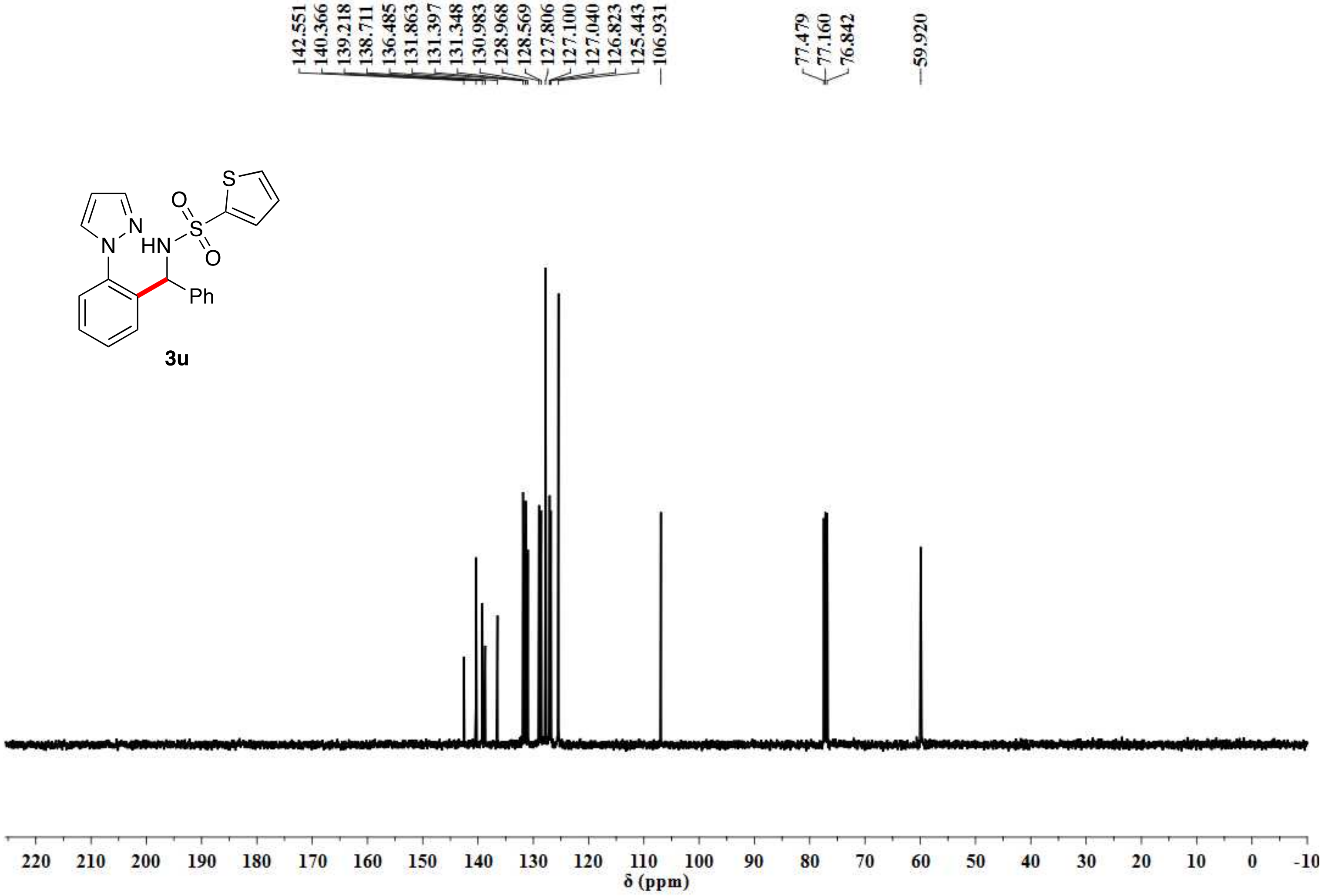
142.551
140.366
139.218
138.711
136.485
131.863
131.397
131.348
130.983
128.968
128.569
127.806
127.100
127.040
126.823
125.443
106.931
77.479
77.160
76.842
59.920
S
O
N
S
N
HN
O
Ph
3u
220 210 200 190 180 170 160 150 140 130 120 110 100 90 80 70 60 50 40 30 20 10 0 -10
δ (ppm)

Supplementary Figure 50. Characterization of product 3v. (a) ^{1}HNMR spectrum. (b) ^{13}C NMR spectrum.

a

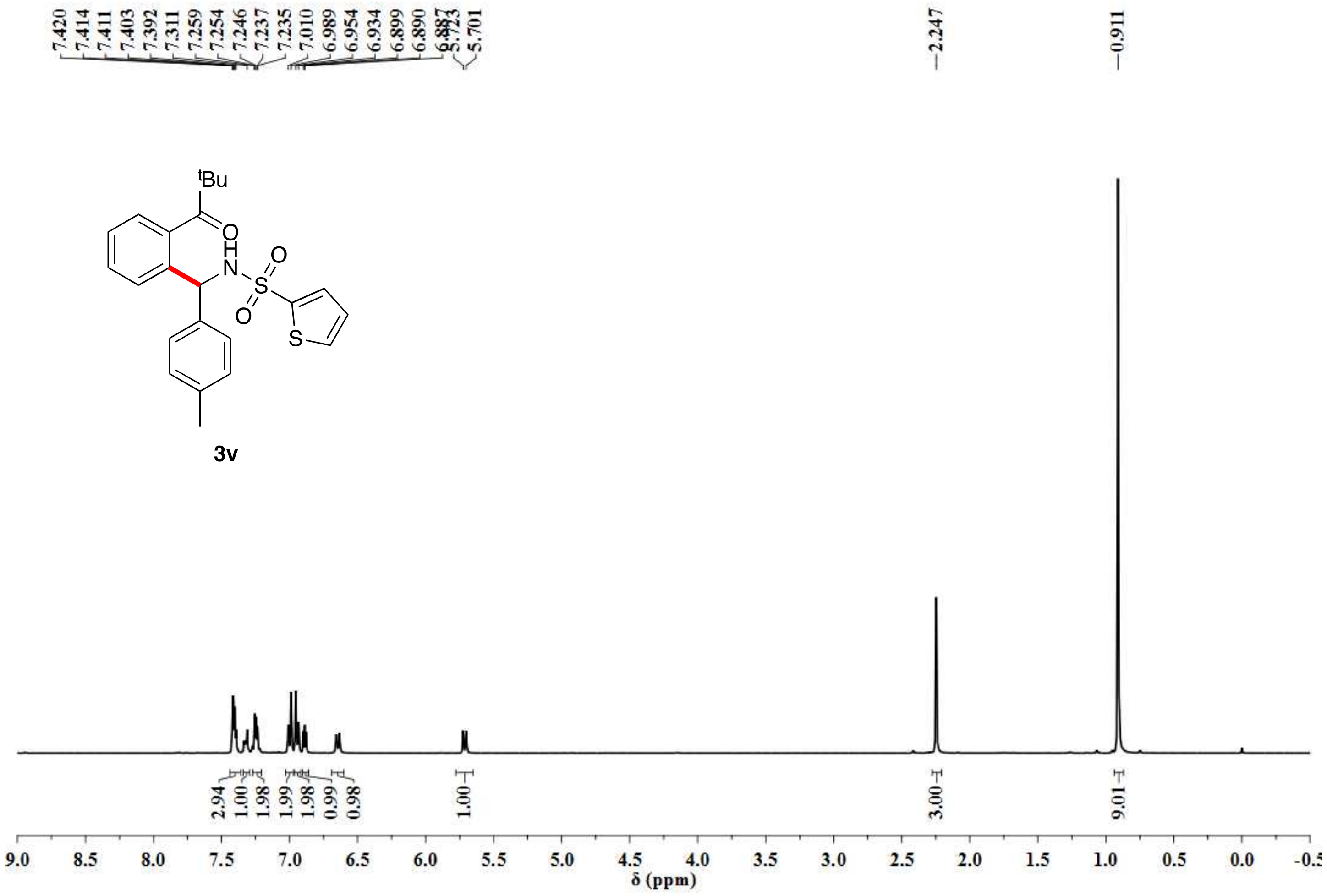

b

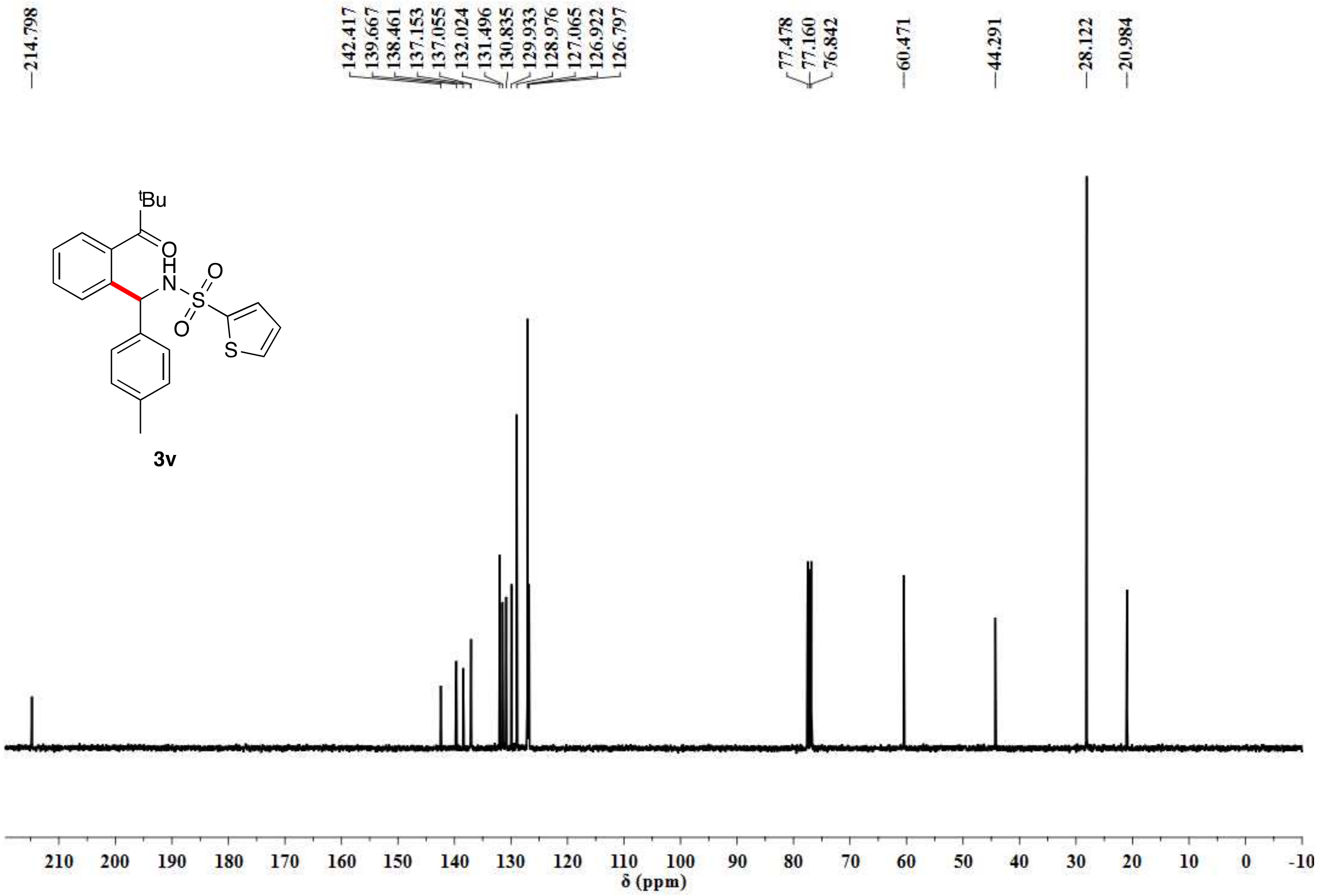
214.798
142.417
139.667
138.461
137.153
137.055
132.024
131.496
130.835
129.933
128.976
127.065
126.922
126.797
77.478
77.160
76.842
60.471
44.291
28.122
20.984
tBu
O
H
N
S
O
O
S
3v
210 200 190 180 170 160 150 140 130 120 110 100 90 80 70 60 50 40 30 20 10 0 -10
δ (ppm)

Supplementary Figure 51. Characterization of product 3w. (a) ^{1}HNMR spectrum. (b) ^{13}C NMR spectrum.

a

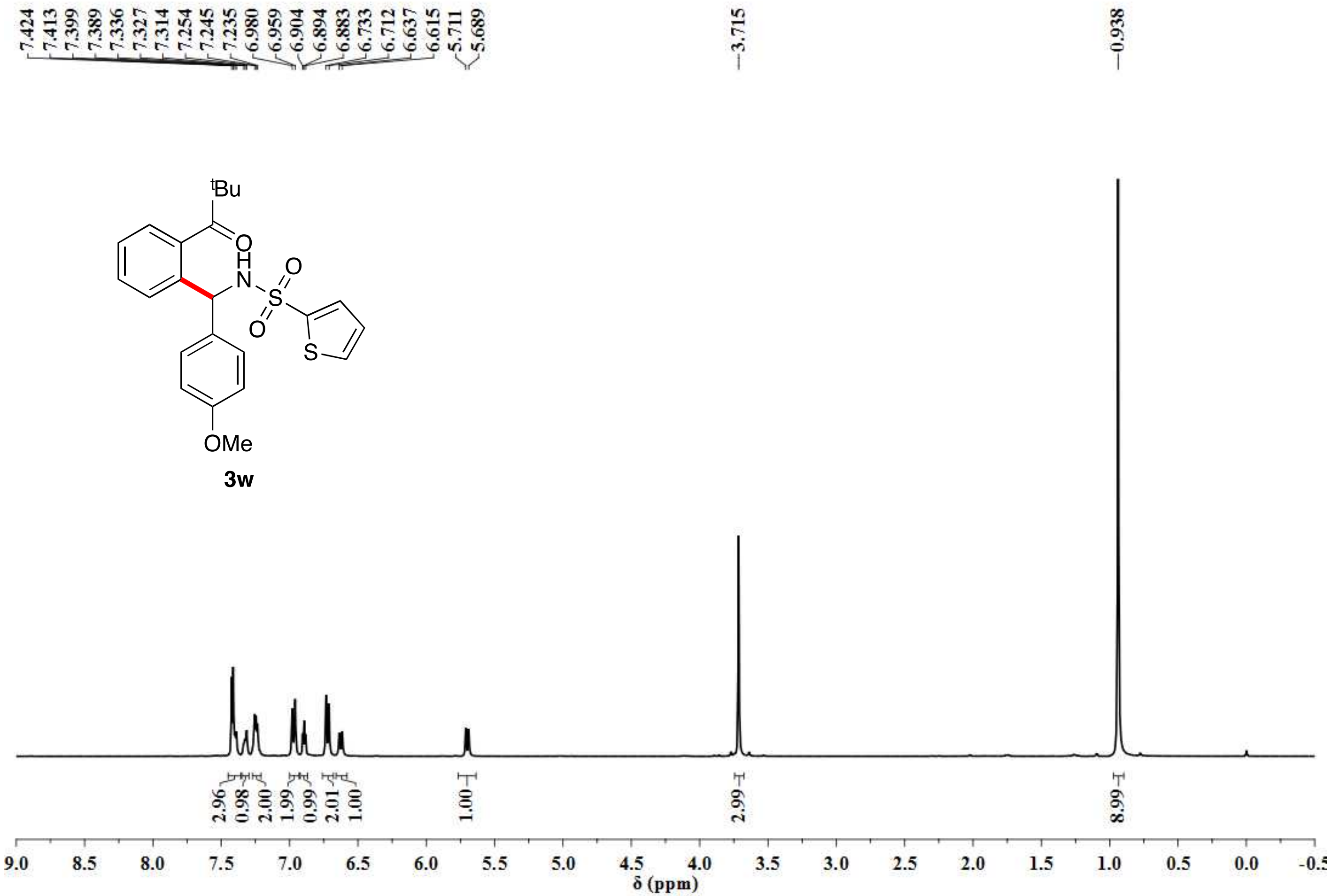

b

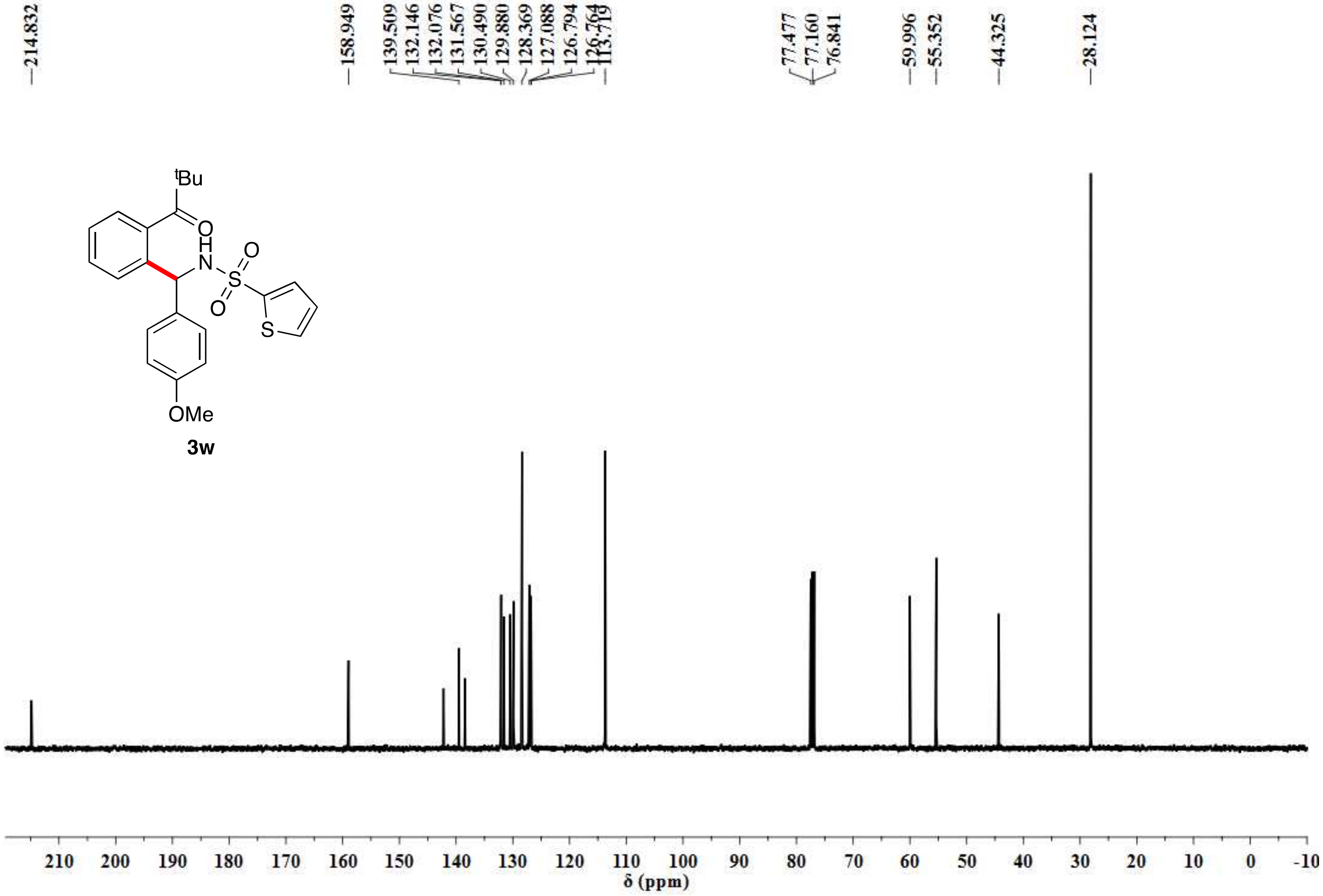

214.832
158.949
139.509
132.146
132.076
131.567
130.490
129.880
128.369
127.088
126.794
126.764
113.719
77.477
77.160
76.841
59.996
55.352
44.325
28.124
tBu
O
H
N
S
O
O
S
OMe
210 200 190 180 170 160 150 140 130 120 110 100 90 80 70 60 50 40 30 20 10 0 -10
δ (ppm)

3w

Supplementary Figure 52. Characterization of product 3x. (a) ^{1}HNMR spectrum. (b) ^{13}C NMR spectrum. (c) ^{19}F NNR spectrum

a

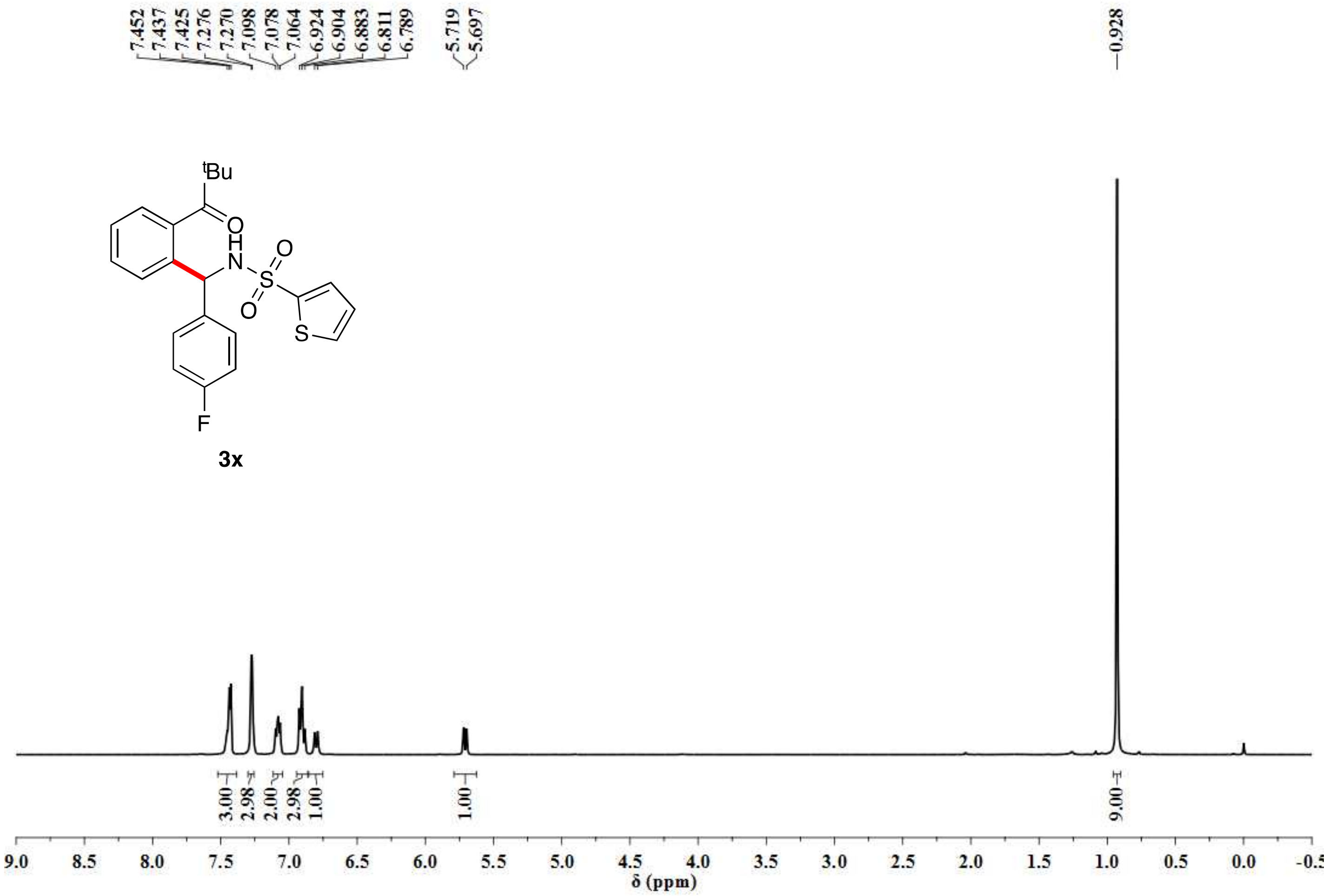

b

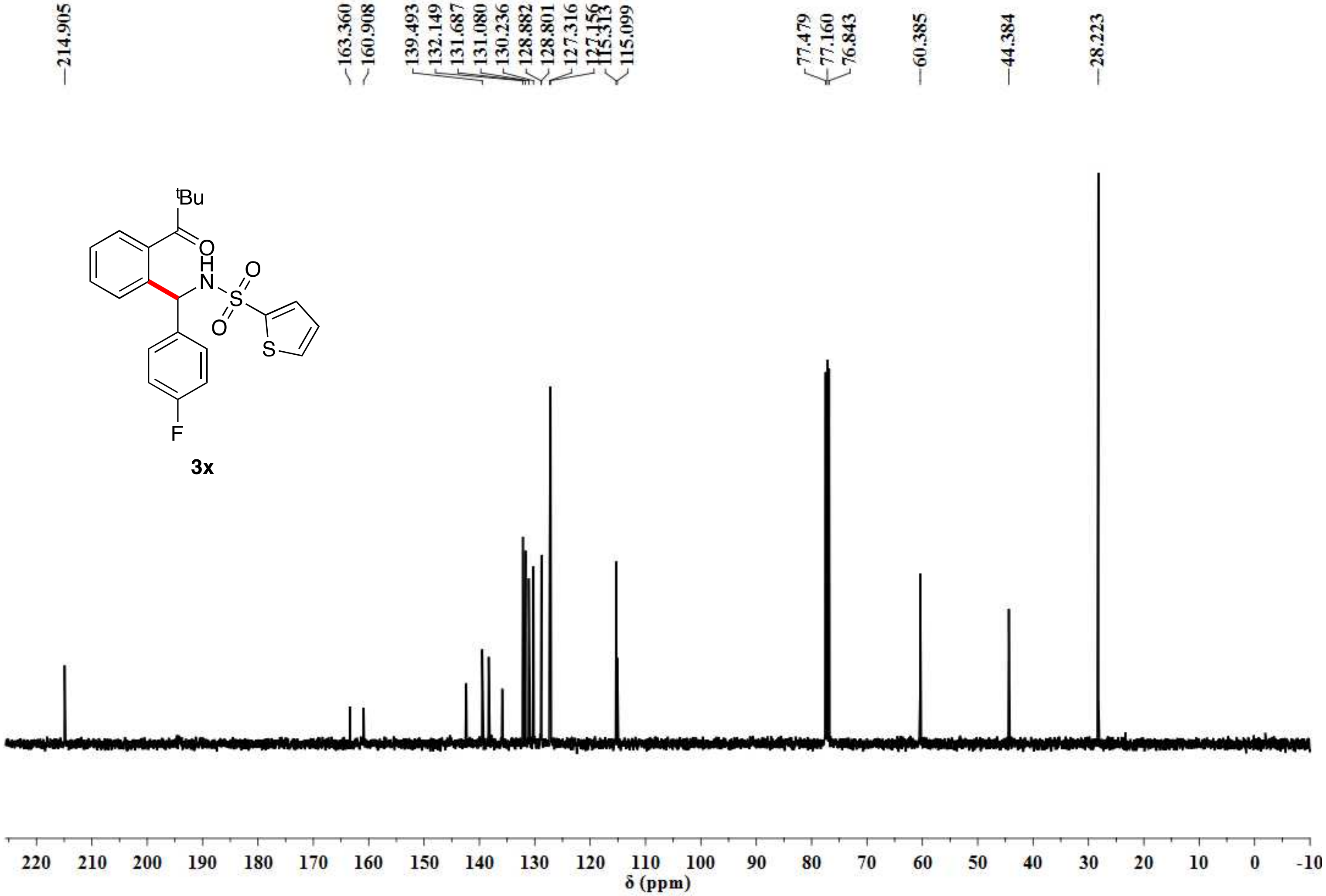
214.905
163.360
160.908
139.493
132.149
131.687
131.080
130.236
128.882
128.801
127.316
127.156
115.313
115.099
77.479
77.160
76.843
60.385
44.384
28.223
tBu
O
H
N
O
S
O
S
F
3x
220 210 200 190 180 170 160 150 140 130 120 110 100 90 80 70 60 50 40 30 20 10 0 -10
δ (ppm)

c

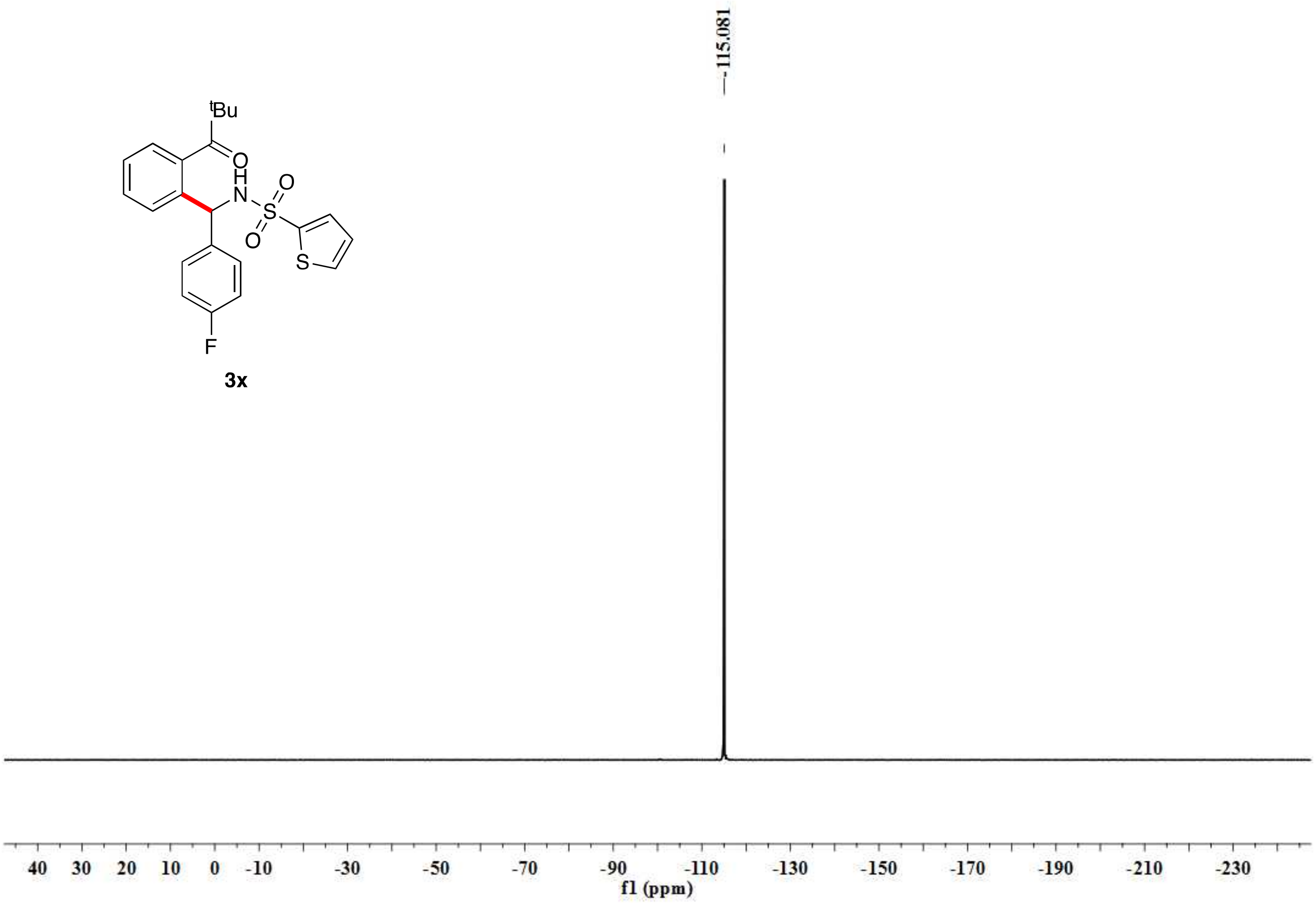
-115.081
tBu
O
H
N
S
O
O
S
F
3x
40 30 20 10 0 -10 -30 -50 -70 -90 -110 -130 -150 -170 -190 -210 -230
f1 (ppm)

Supplementary Figure 53. Characterization of product 3y. (a) ^{1}HNMR spectrum. (b) ^{13}C NMR spectrum.

a

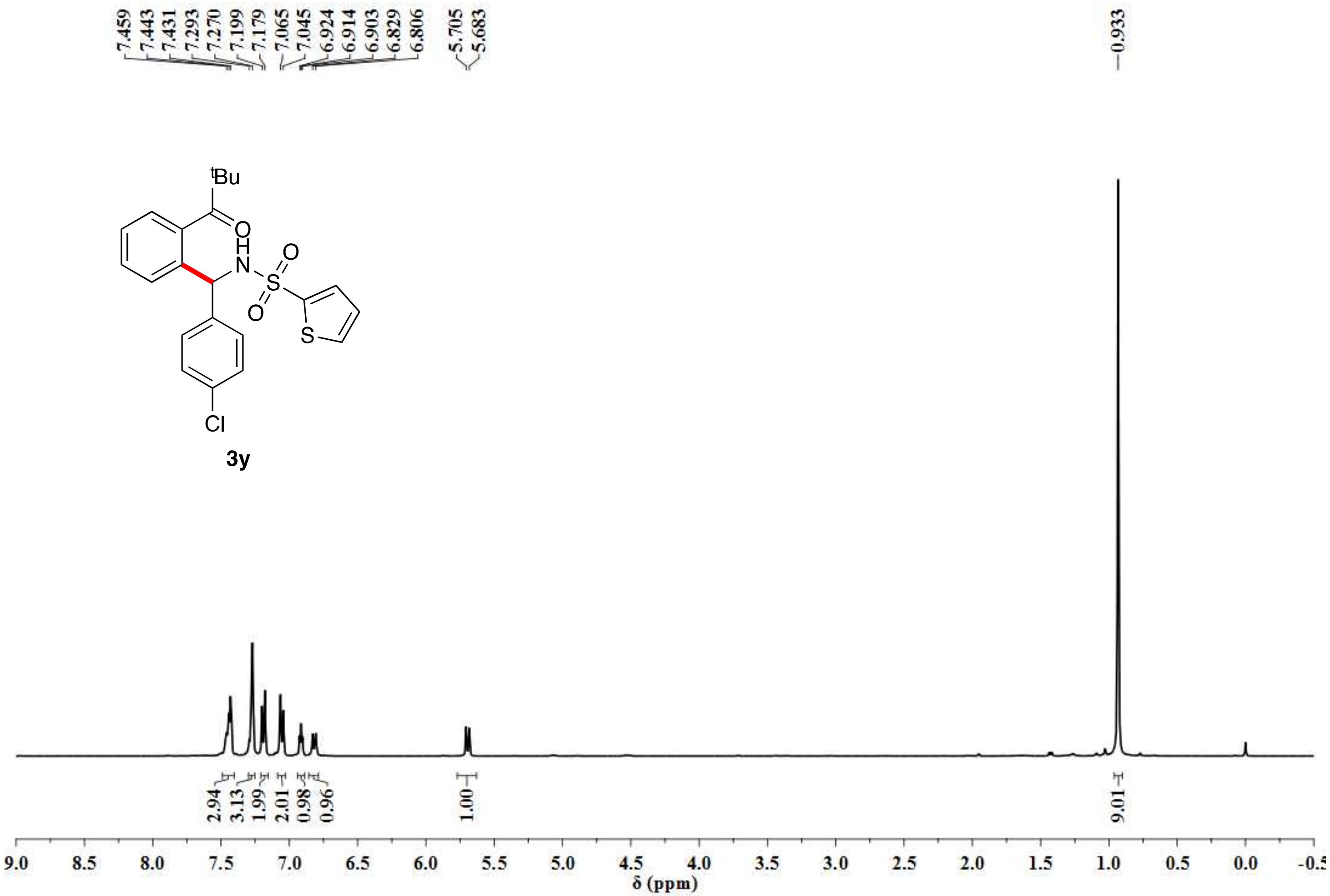

b

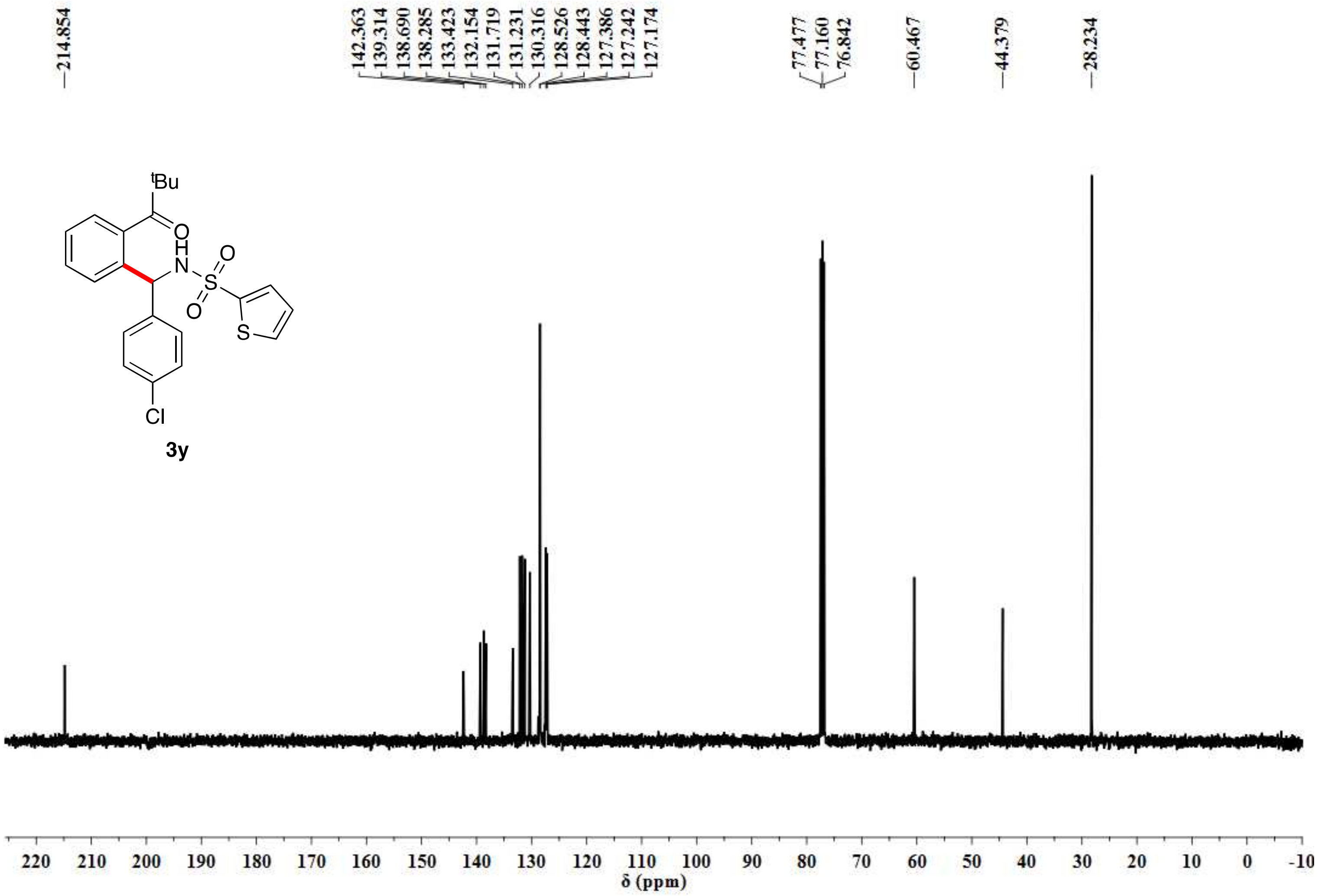
214.854
142.363
139.314
138.690
138.285
133.423
132.154
131.719
131.231
130.316
128.526
128.443
127.386
127.242
127.174
77.477
77.160
76.842
60.467
44.379
28.234
tBu
O
H
N
S
O
O
S
Cl
3y
220 210 200 190 180 170 160 150 140 130 120 110 100 90 80 70 60 50 40 30 20 10 0 -10
δ (ppm)

Supplementary Figure 54. Characterization of product 3z. (a) ^{1}HNMR spectrum. (b) ^{13}C NMR spectrum.

a

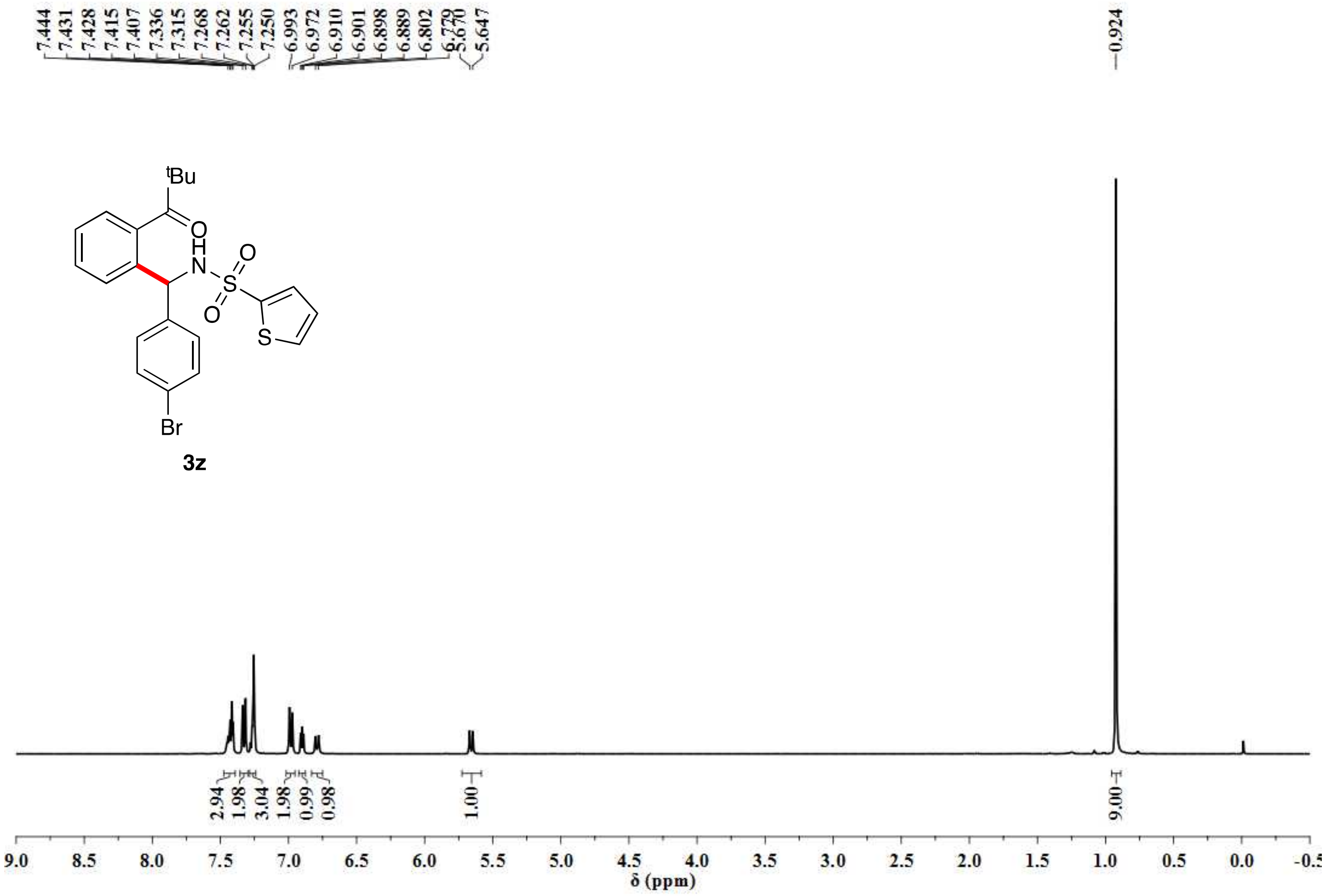

b

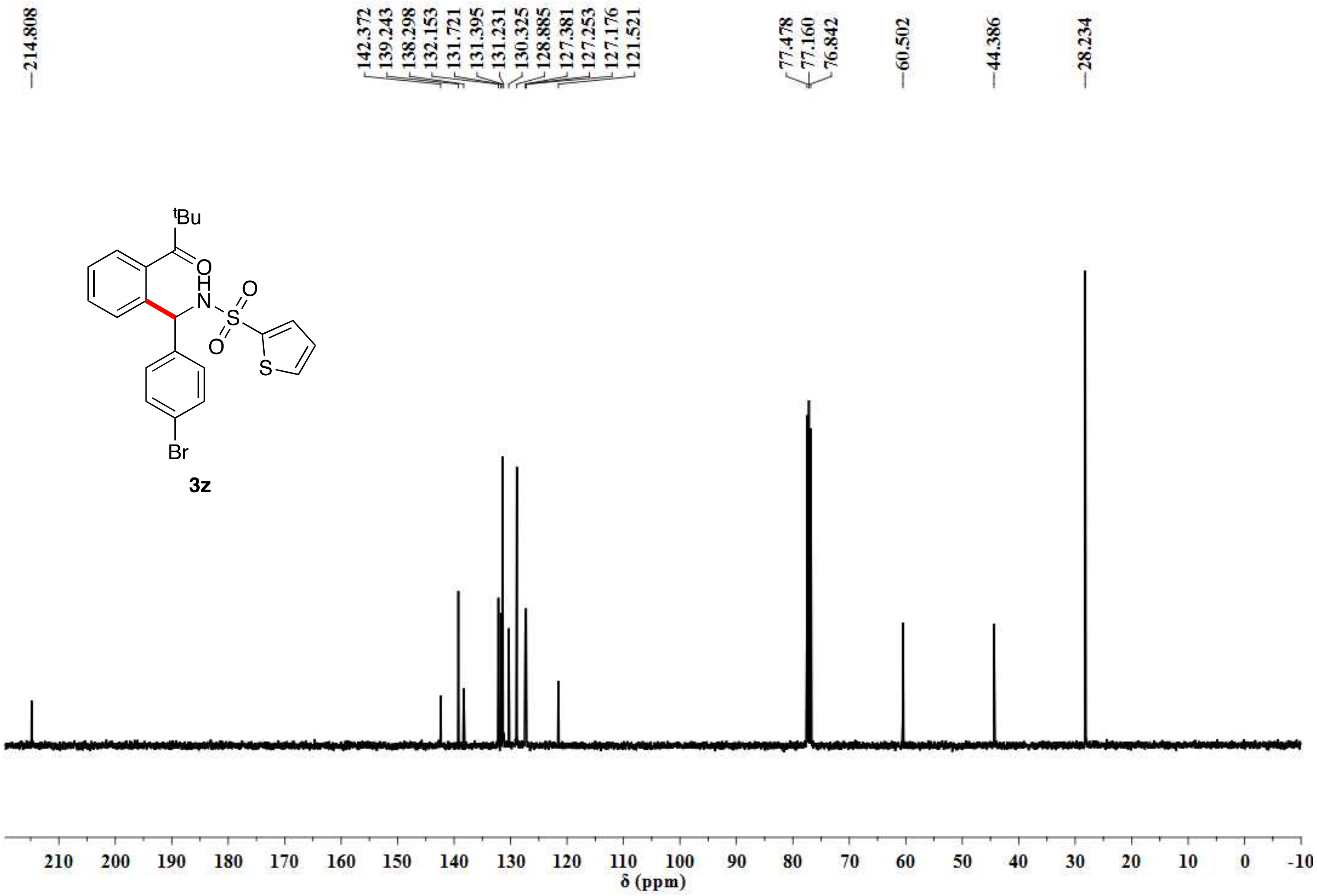
214.808
142.372
139.243
138.298
132.153
131.721
131.395
131.231
130.325
128.885
127.381
127.253
127.176
121.521
77.478
77.160
76.842
60.502
44.386
28.234
tBu
O
H
N
O
S
O
S
Br
3z
210
200
190
180
170
160
150
140
130
120
110
100
90
80
70
60
50
40
30
20
10
0
-10
δ (ppm)

Supplementary Figure 55. Characterization of product 3A. (a) ^{1}HNMR spectrum. (b) ^{13}C NMR spectrum. (c) ^{19}F NMR spectrum

a

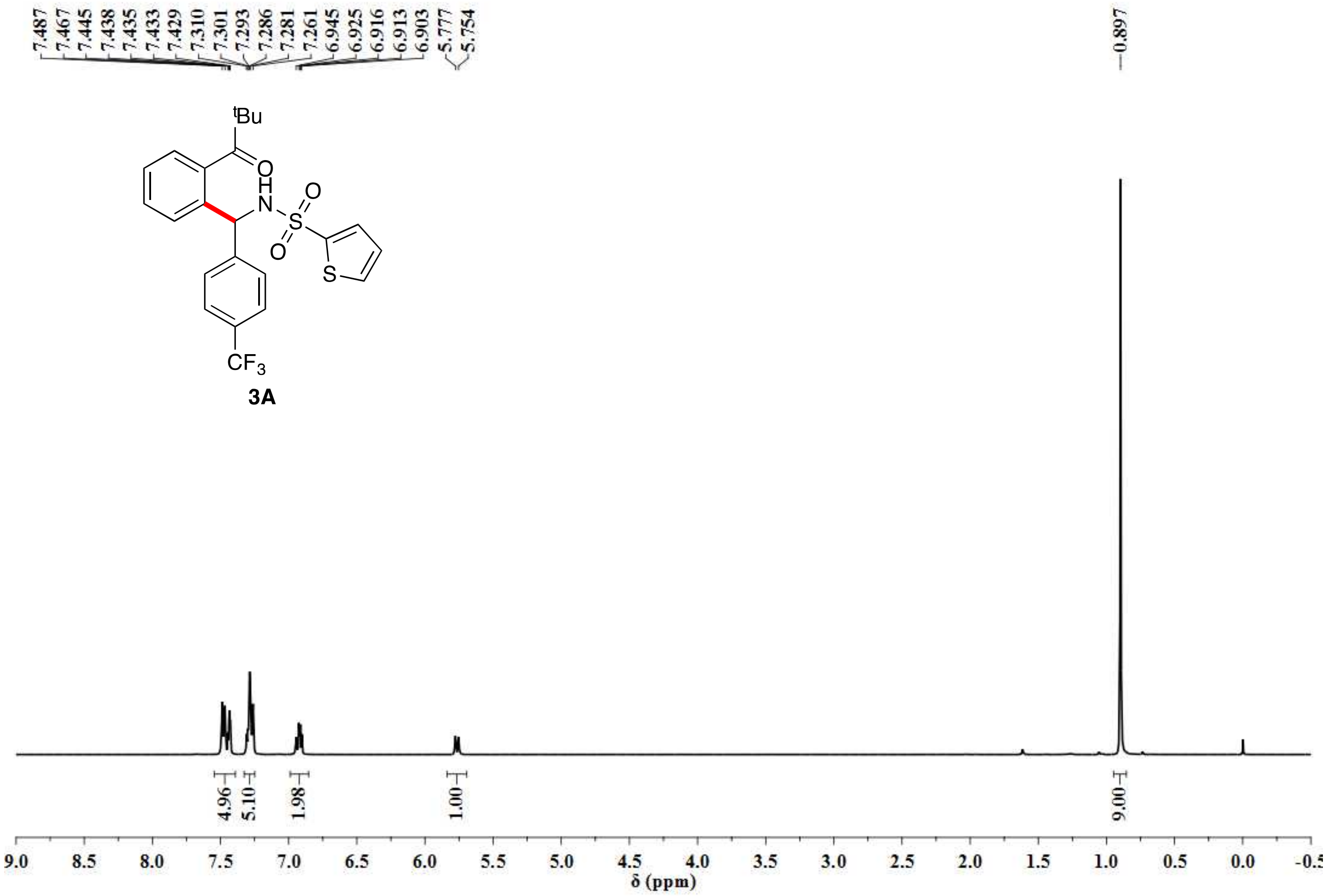

b

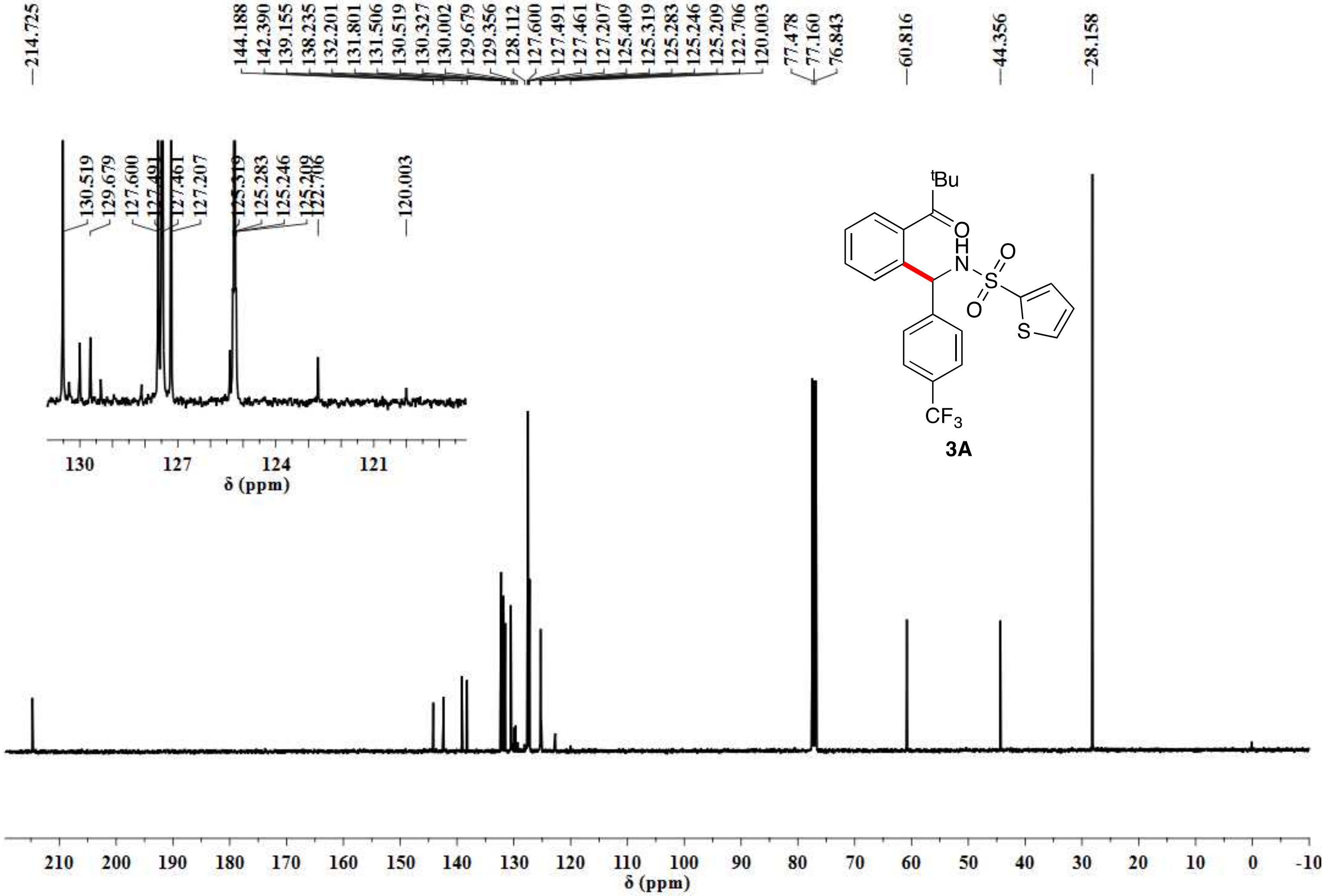
214.725
144.188
142.390
139.155
138.235
132.201
131.801
131.506
130.519
130.327
130.002
129.679
129.356
128.112
127.600
127.491
127.461
127.207
125.409
125.319
125.283
125.246
125.209
122.706
120.003
77.478
77.160
76.843
60.816
44.356
28.158
130.519
129.679
127.600
127.491
127.461
127.207
125.319
125.283
125.246
125.209
122.706
120.003
130
127
124
121
δ (ppm)
^{t}Bu
O
H
N
S
O
O
S
CF_3
3A
210 200 190 180 170 160 150 140 130 120 110 100 90 80 70 60 50 40 30 20 10 0 -10
δ (ppm)

c

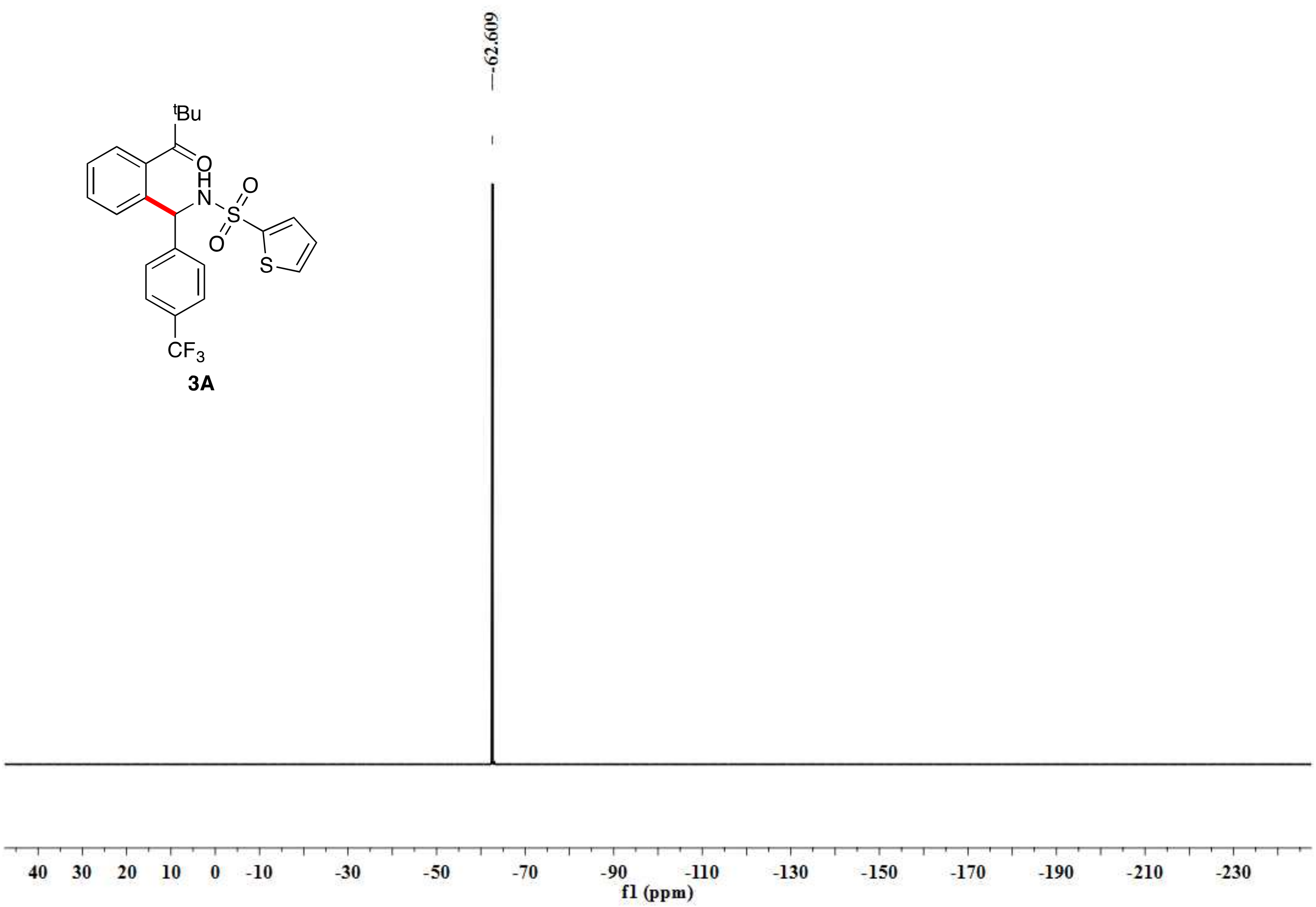
-62.609
tBu
O
H
N
S
O
O
S
CF3
3A
40
30
20
10
0
-10
-30
-50
-70
-90
-110
-130
-150
-170
-190
-210
-230
f1 (ppm)

Supplementary Figure 56. Characterization of product 3B. (a) ^{1}HNMR spectrum. (b) ^{13}C NMR spectrum.

a

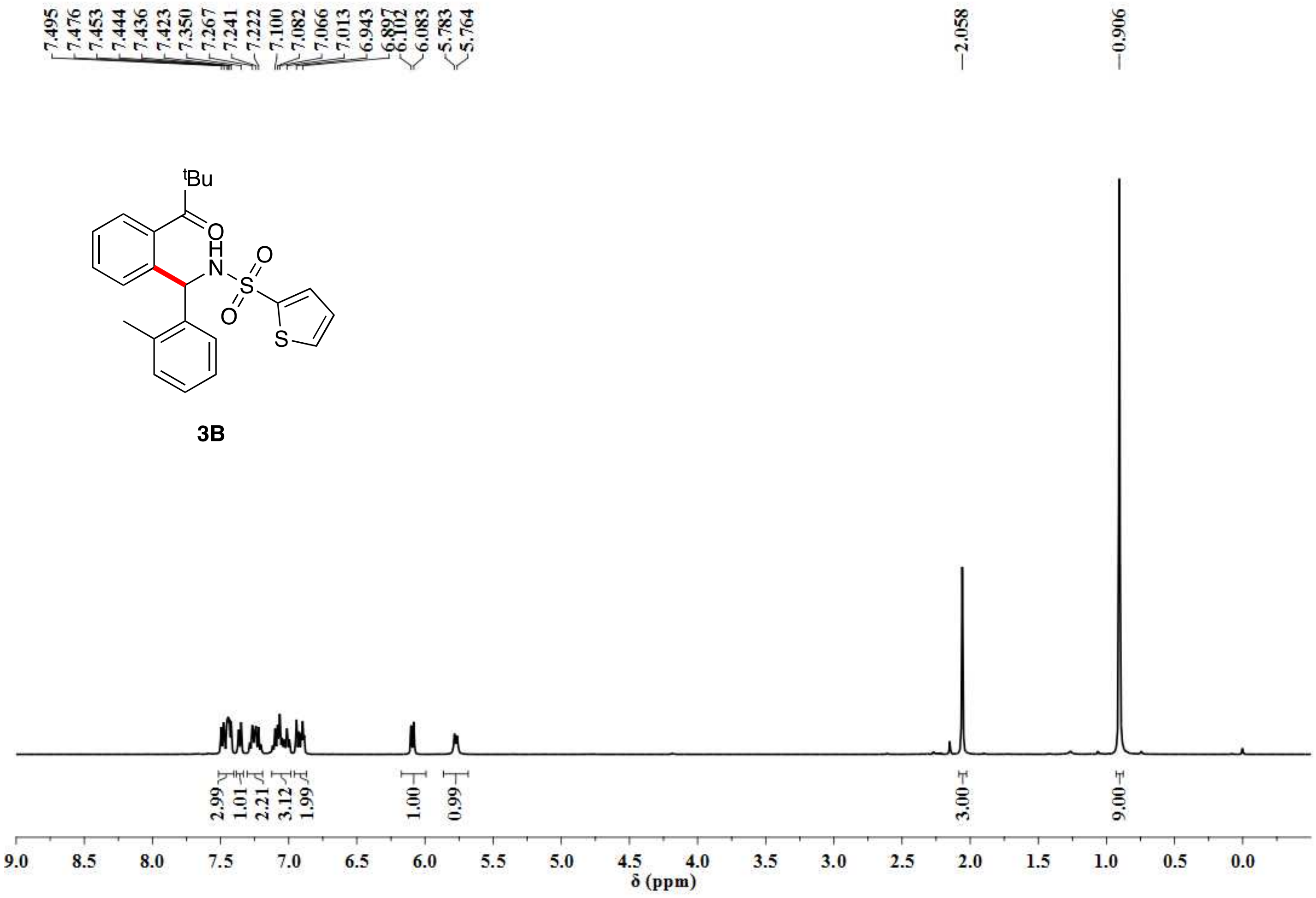

b

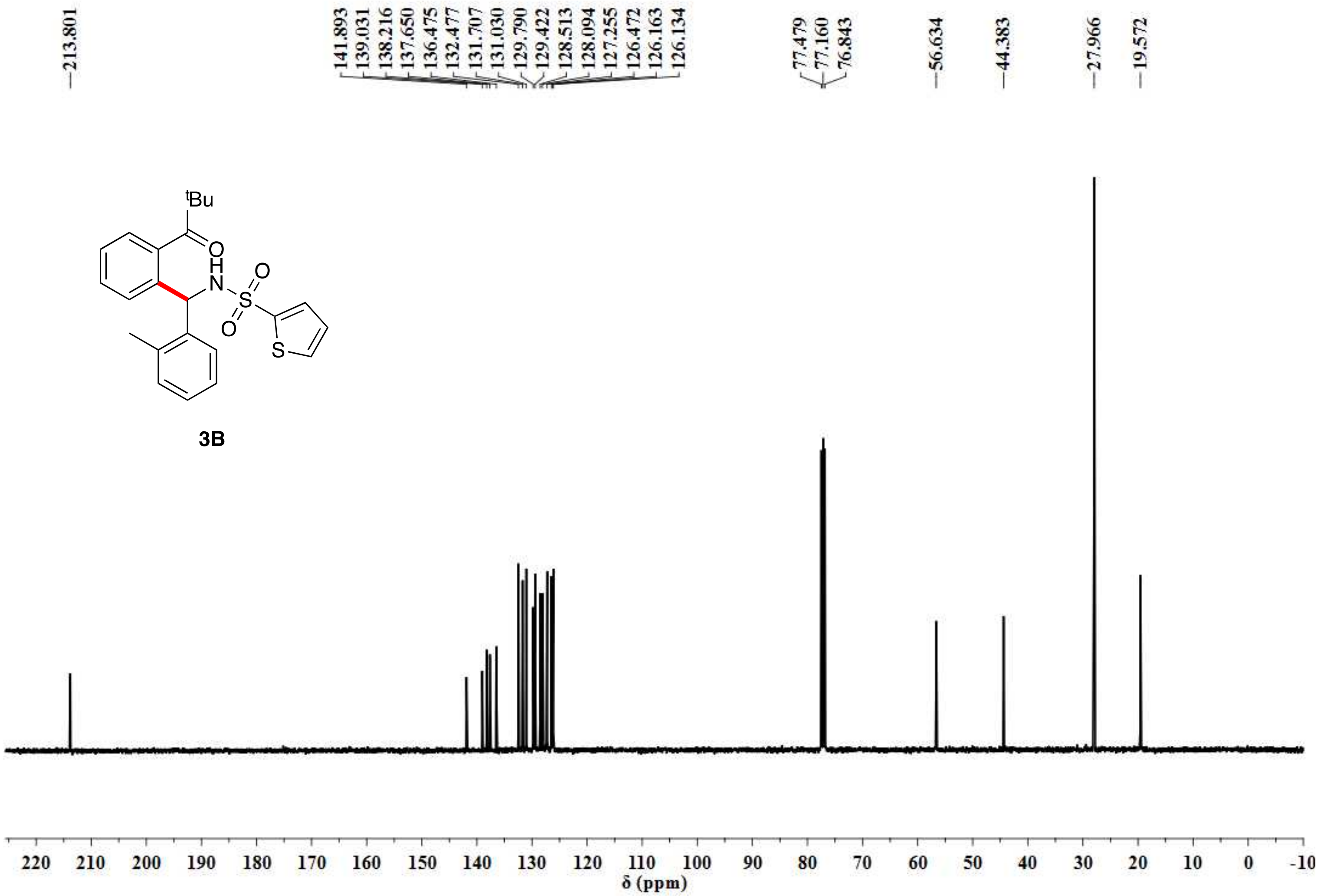

213.801
141.893
139.031
138.216
137.650
136.475
132.477
131.707
131.030
129.790
129.422
128.513
128.094
127.255
126.472
126.163
126.134
77.479
77.160
76.843
56.634
44.383
27.966
19.572
tBu
O
H
N
S
O
O
S
3B
220 210 200 190 180 170 160 150 140 130 120 110 100 90 80 70 60 50 40 30 20 10 0 -10
δ (ppm)

Supplementary Figure 57. Characterization of product 3C. (a) ^{1}HNMR spectrum. (b) ^{13}C NMR spectrum.

a

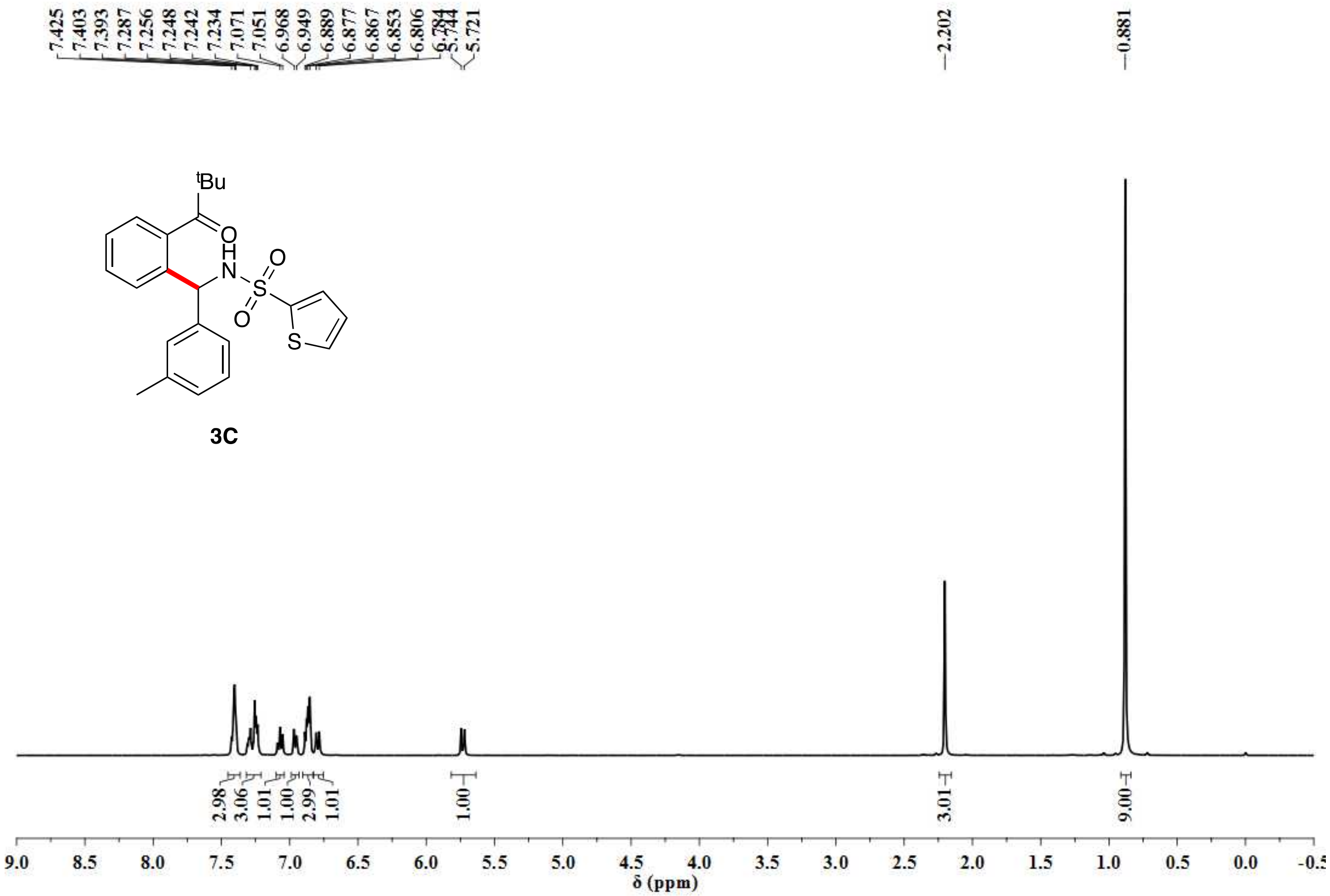

b

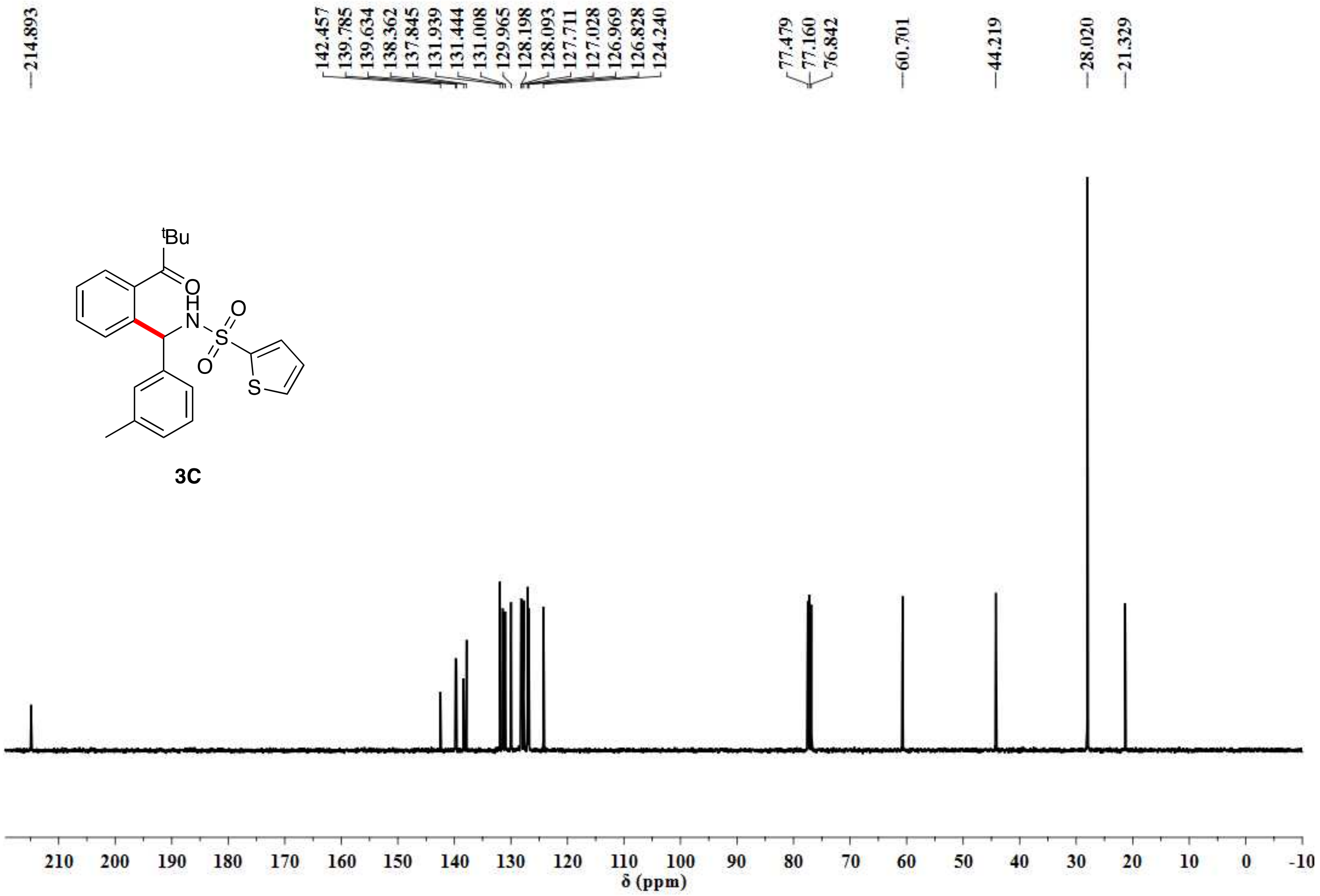

214.893
142.457
139.785
139.634
138.362
137.845
131.939
131.444
131.008
129.965
128.198
128.093
127.711
127.028
126.969
126.828
124.240
77.479
77.160
76.842
60.701
44.219
28.020
21.329
tBu
O
H
N
S
O
O
S
3C
210 200 190 180 170 160 150 140 130 120 110 100 90 80 70 60 50 40 30 20 10 0 -10
δ (ppm)

Supplementary Figure 58. Characterization of product 3D. (a) ^{1}HNMR spectrum. (b) ^{13}C NMR spectrum.

a

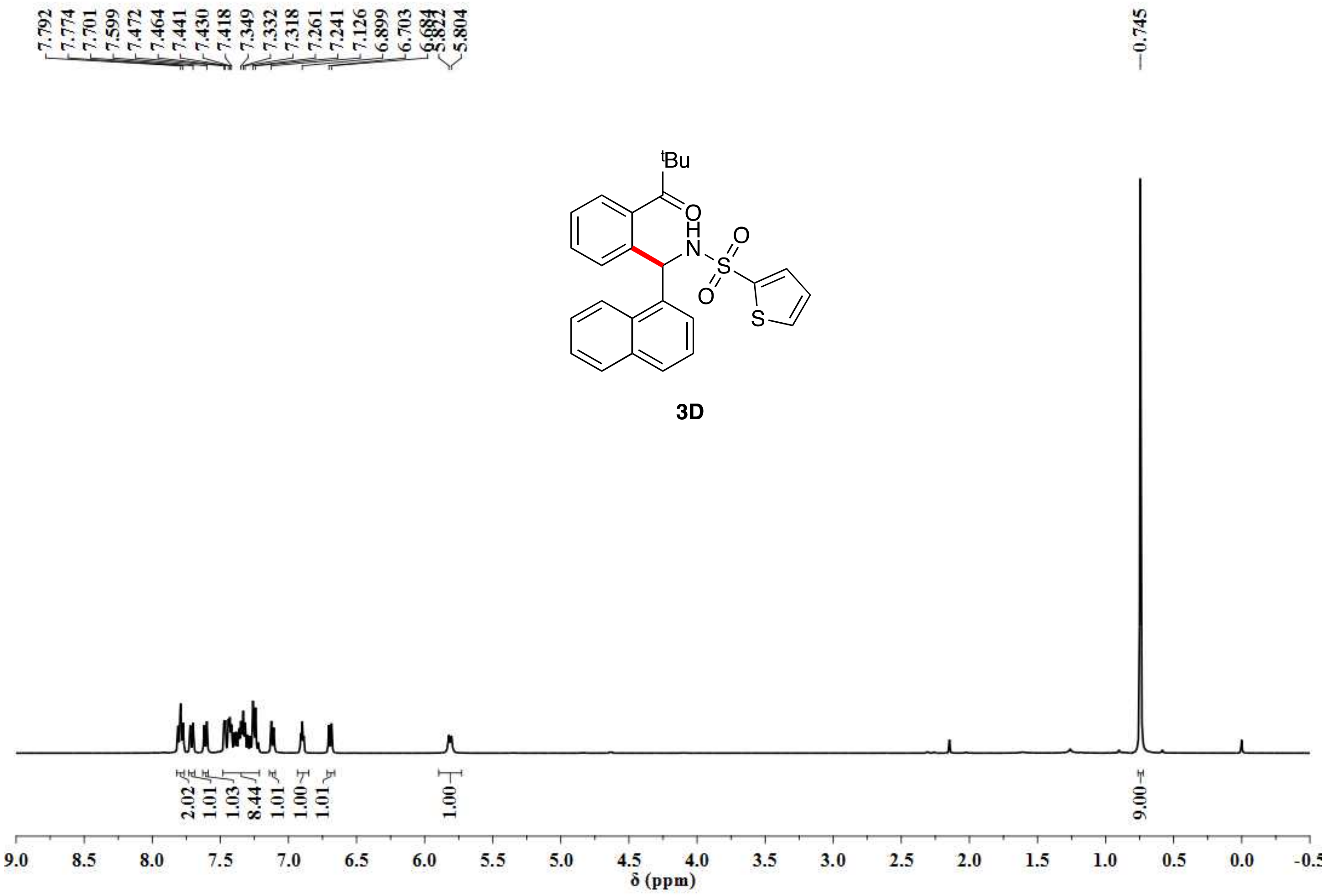

b

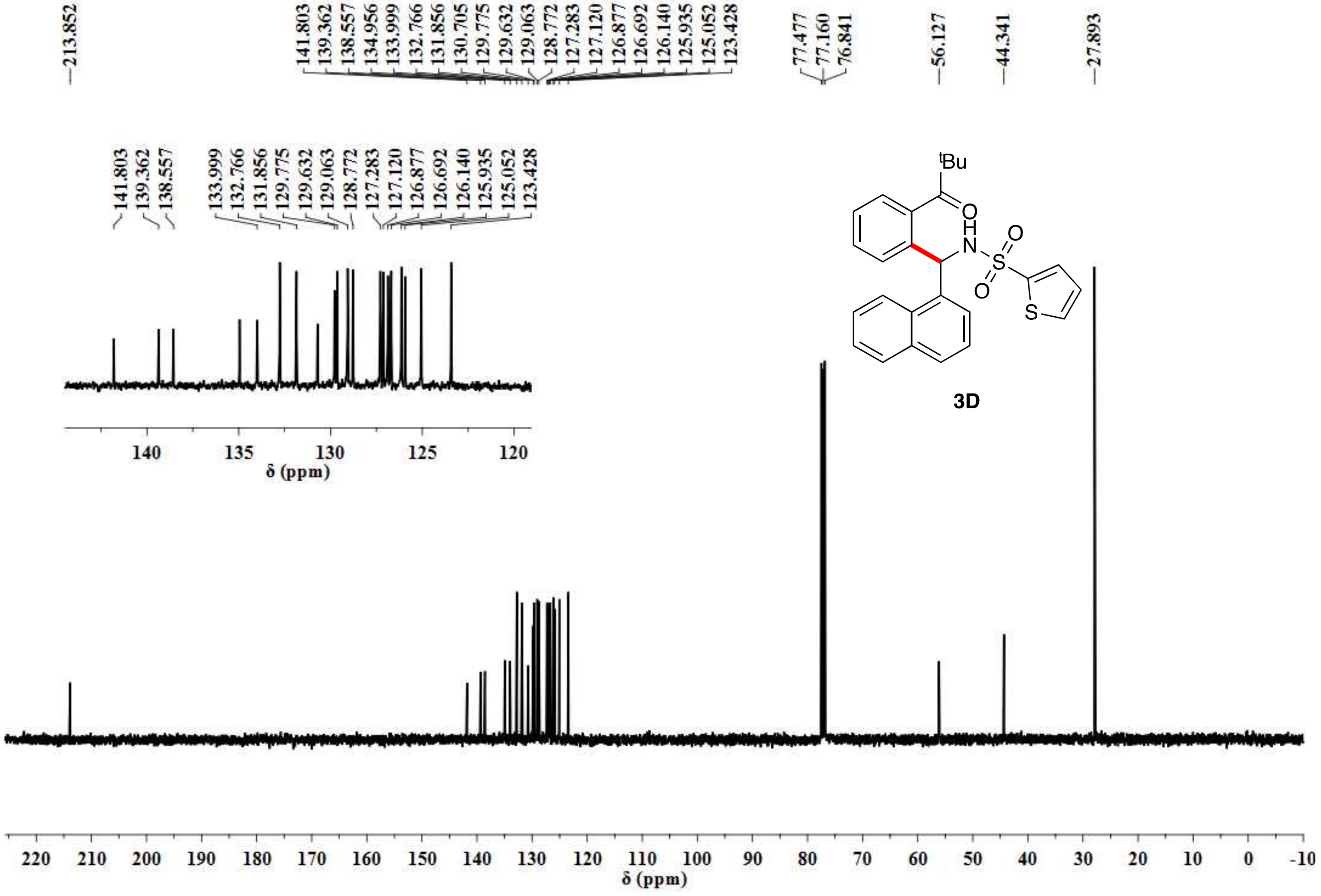
213.852
141.803
139.362
138.557
134.956
133.999
132.766
131.856
130.705
129.775
129.632
129.063
128.772
127.283
127.120
126.877
126.692
126.140
125.935
125.052
123.428
77.477
77.160
76.841
56.127
44.341
27.893
δ (ppm)
tBu
O
H
N
S
O
O
S
3D

Supplementary Figure 59. Characterization of product 3E. (a) ^{1}HNMR spectrum. (b) ^{13}C NMR spectrum.

a

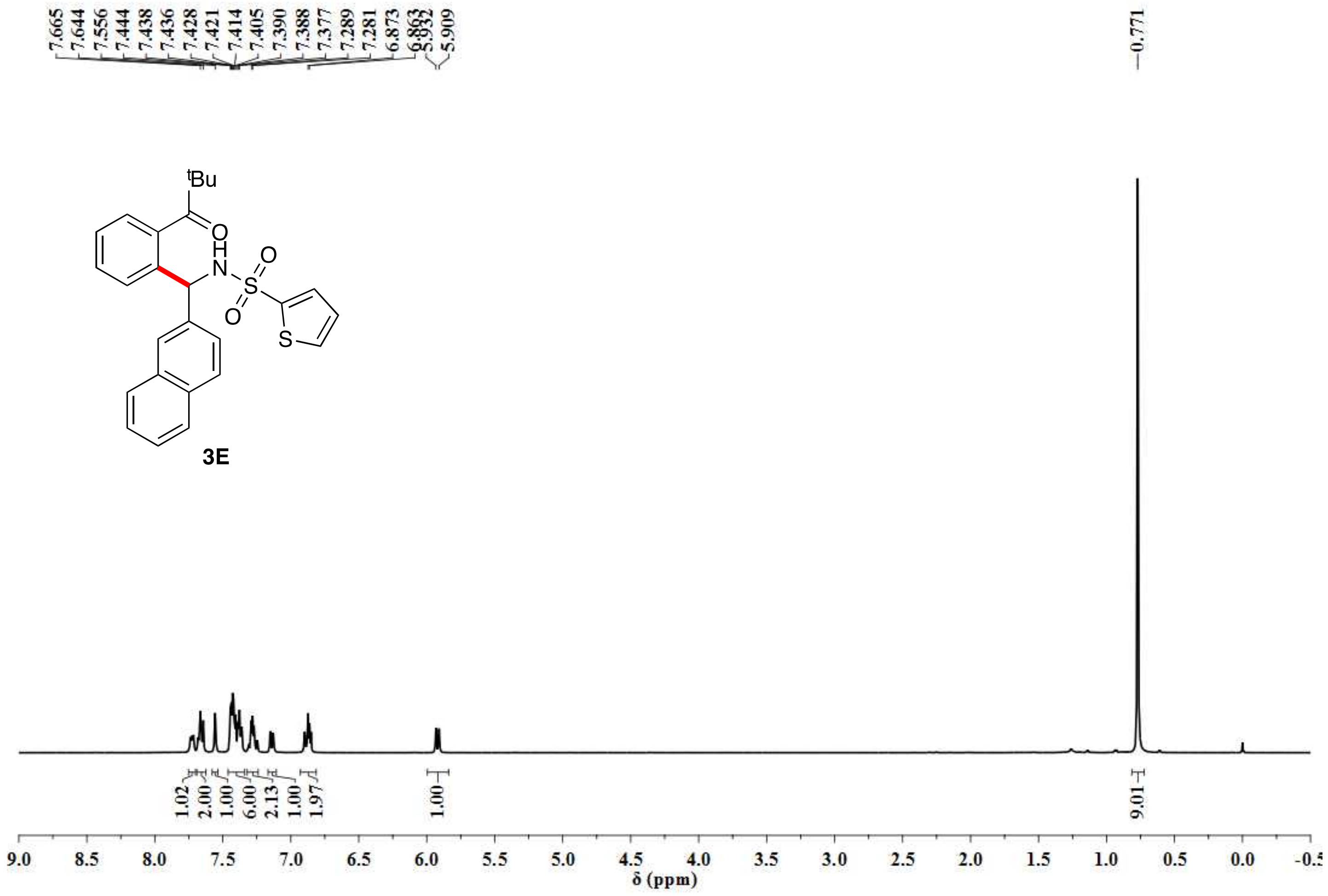

b

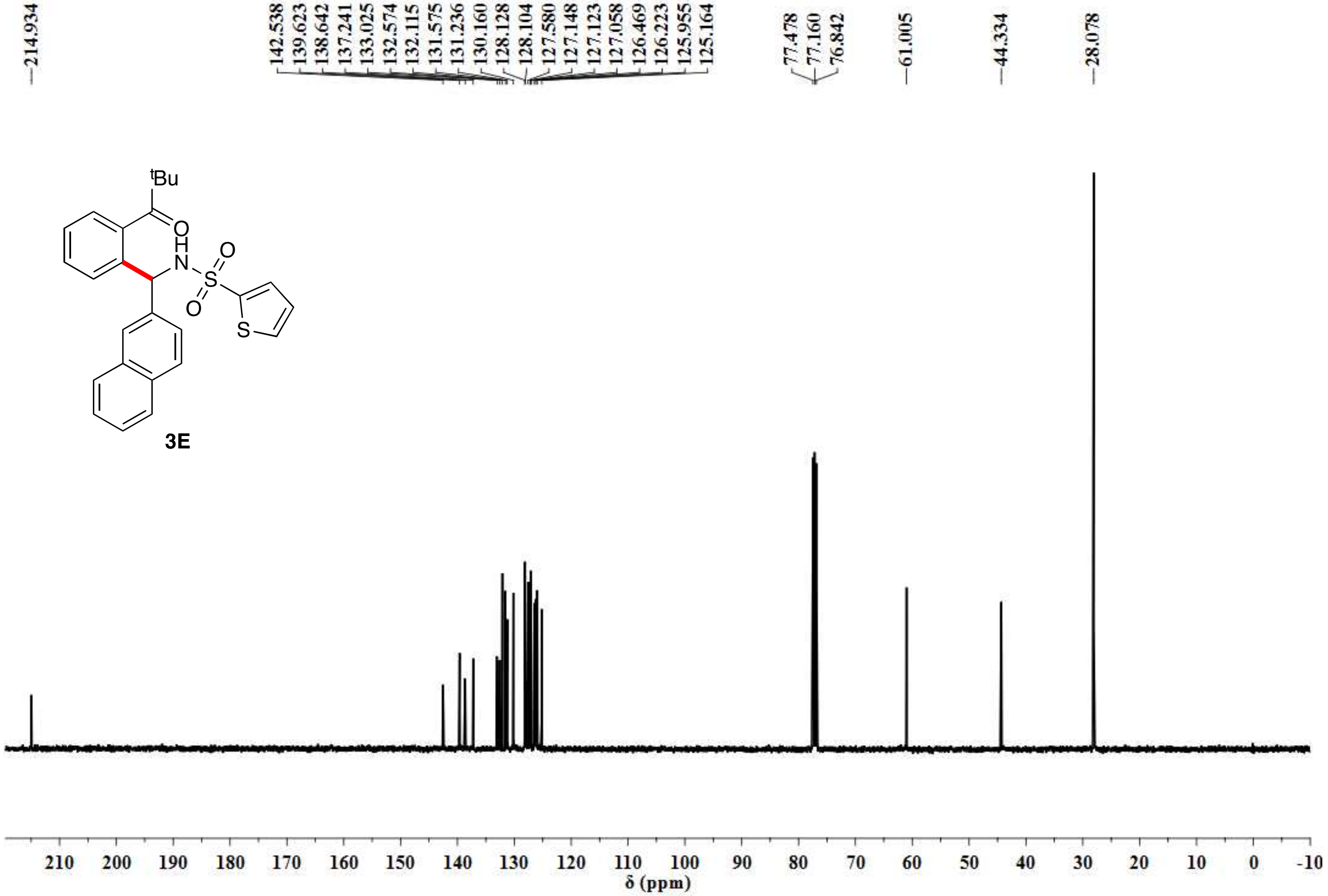

214.934
142.538
139.623
138.642
137.241
133.025
132.574
132.115
131.575
131.236
130.160
128.128
128.104
127.580
127.148
127.123
127.058
126.469
126.223
125.955
125.164
77.478
77.160
76.842
61.005
44.334
28.078
tBu
O
H
N
S
O
O
S
3E
210 200 190 180 170 160 150 140 130 120 110 100 90 80 70 60 50 40 30 20 10 0 -10
δ (ppm)

Supplementary Figure 60. Characterization of product 3F. (a) ^{1}HNMR spectrum. (b) ^{13}C NMR spectrum.

a

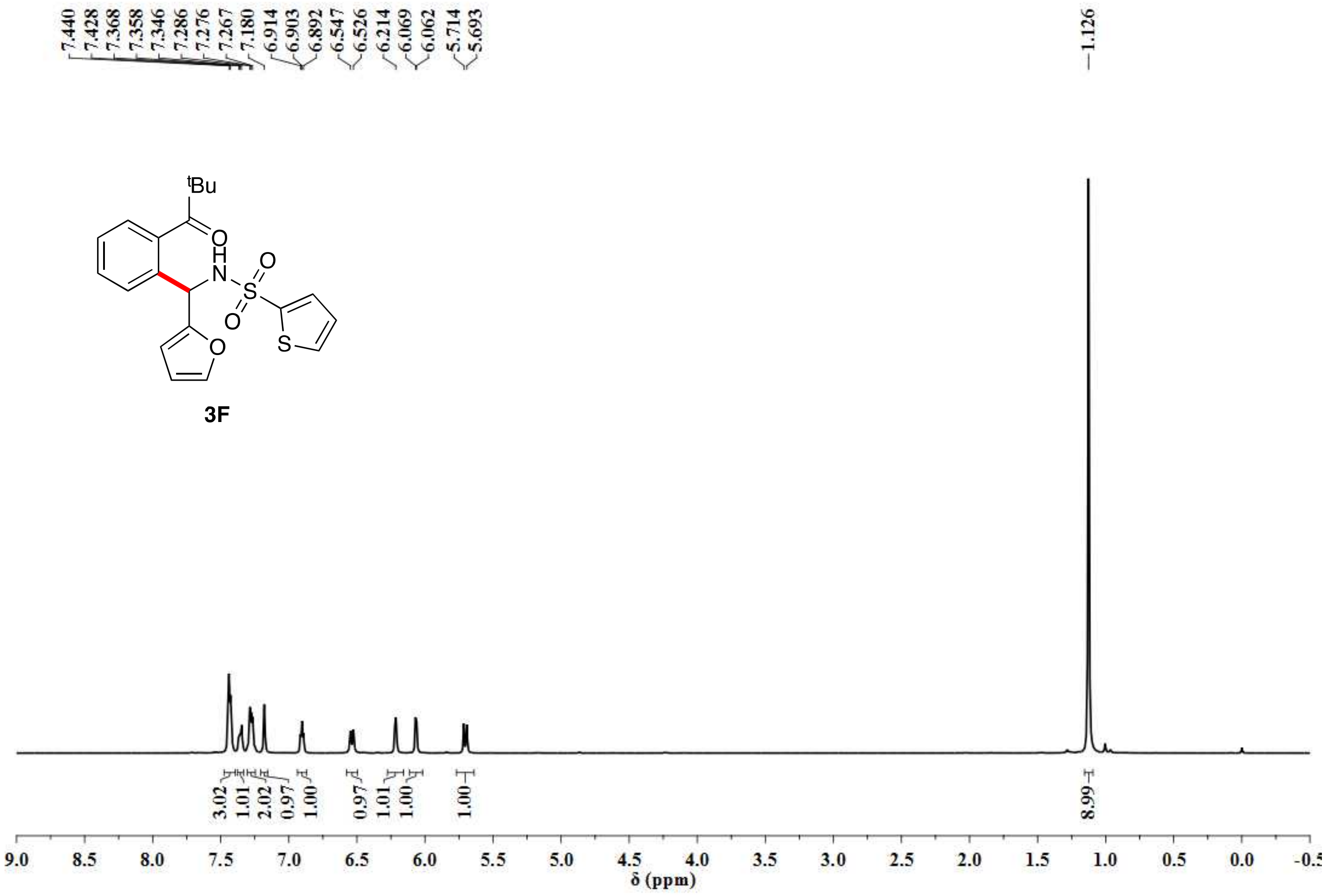

b

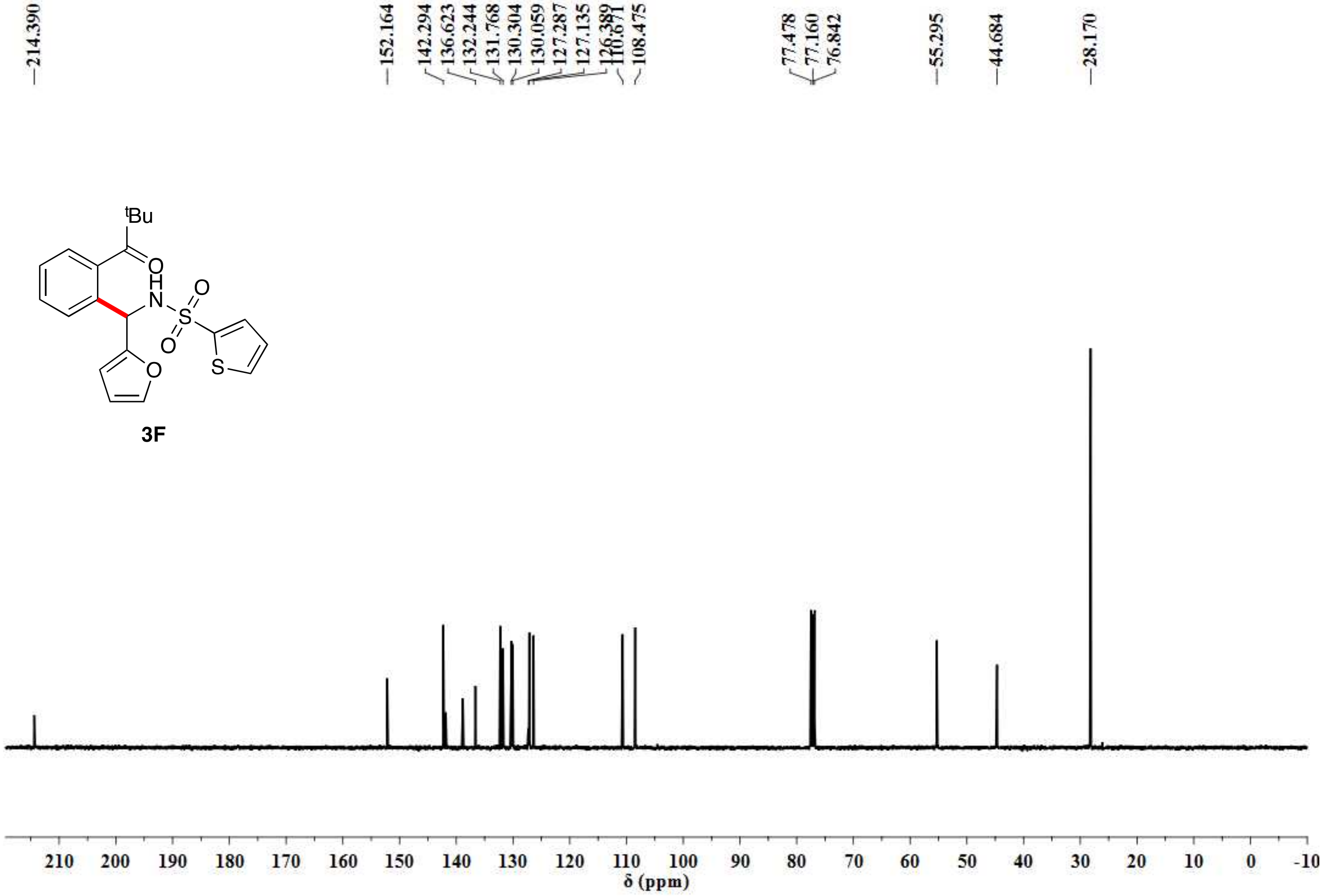
214.390
152.164
142.294
136.623
132.244
131.768
130.304
130.059
127.287
127.135
126.389
110.671
108.475
77.478
77.160
76.842
55.295
44.684
28.170
tBu
O
H
N
S
O
O
O
S
3F
210 200 190 180 170 160 150 140 130 120 110 100 90 80 70 60 50 40 30 20 10 0 -10
δ (ppm)

Supplementary Figure 61. Characterization of product 3G. (a) ^{1}HNMR spectrum. (b) ^{13}C NMR spectrum.

a

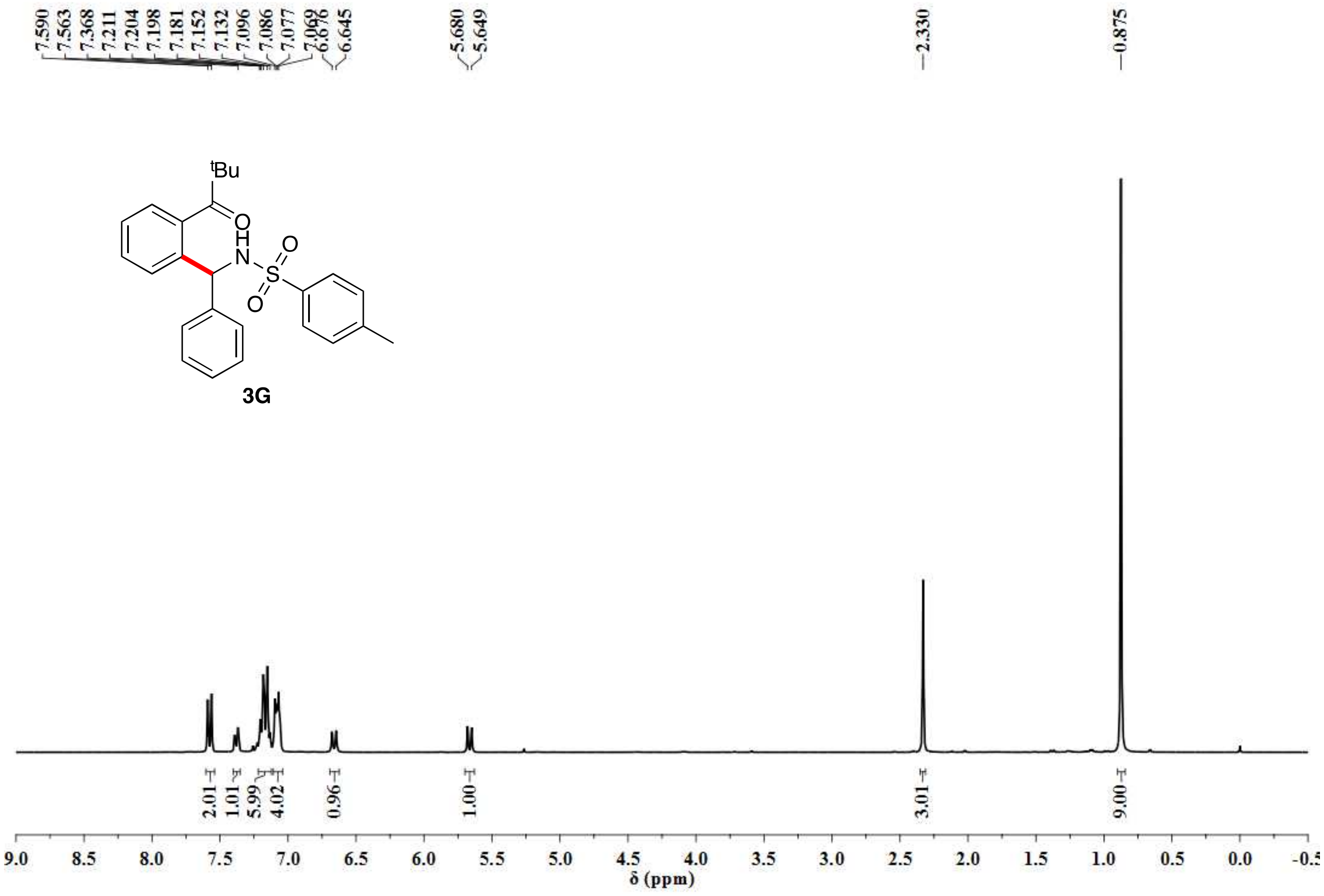

b

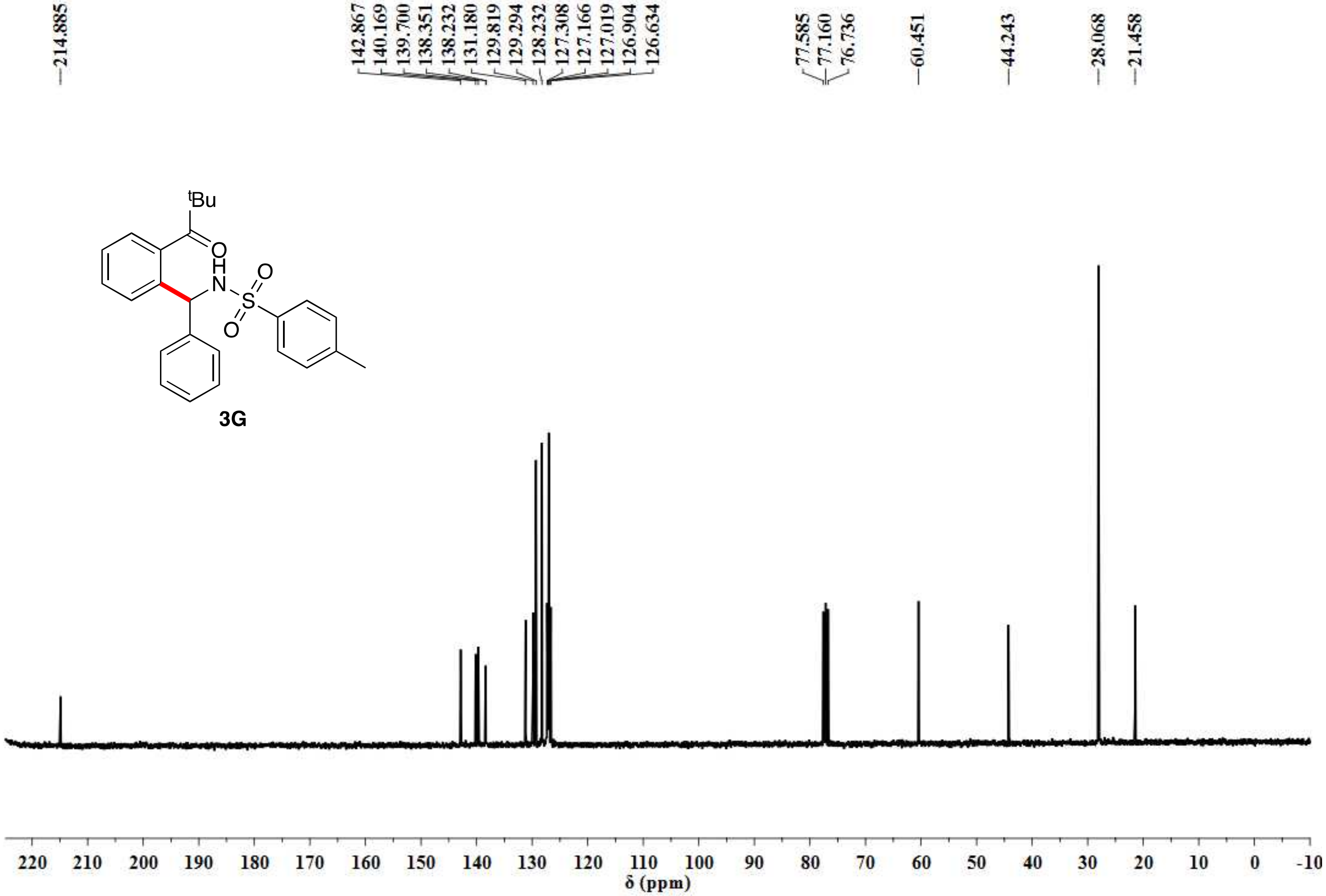
214.885
142.867
140.169
139.700
138.351
138.232
131.180
129.819
129.294
128.232
127.308
127.166
127.019
126.904
126.634
77.585
77.160
76.736
60.451
44.243
28.068
21.458
tBu
O
H
N
S
O
O
3G
220 210 200 190 180 170 160 150 140 130 120 110 100 90 80 70 60 50 40 30 20 10 0 -10
δ (ppm)

Supplementary Figure 62. Characterization of product 3H-major. (a) ^{1}HNMR spectrum. (b) ^{13}C NMR spectrum.

a

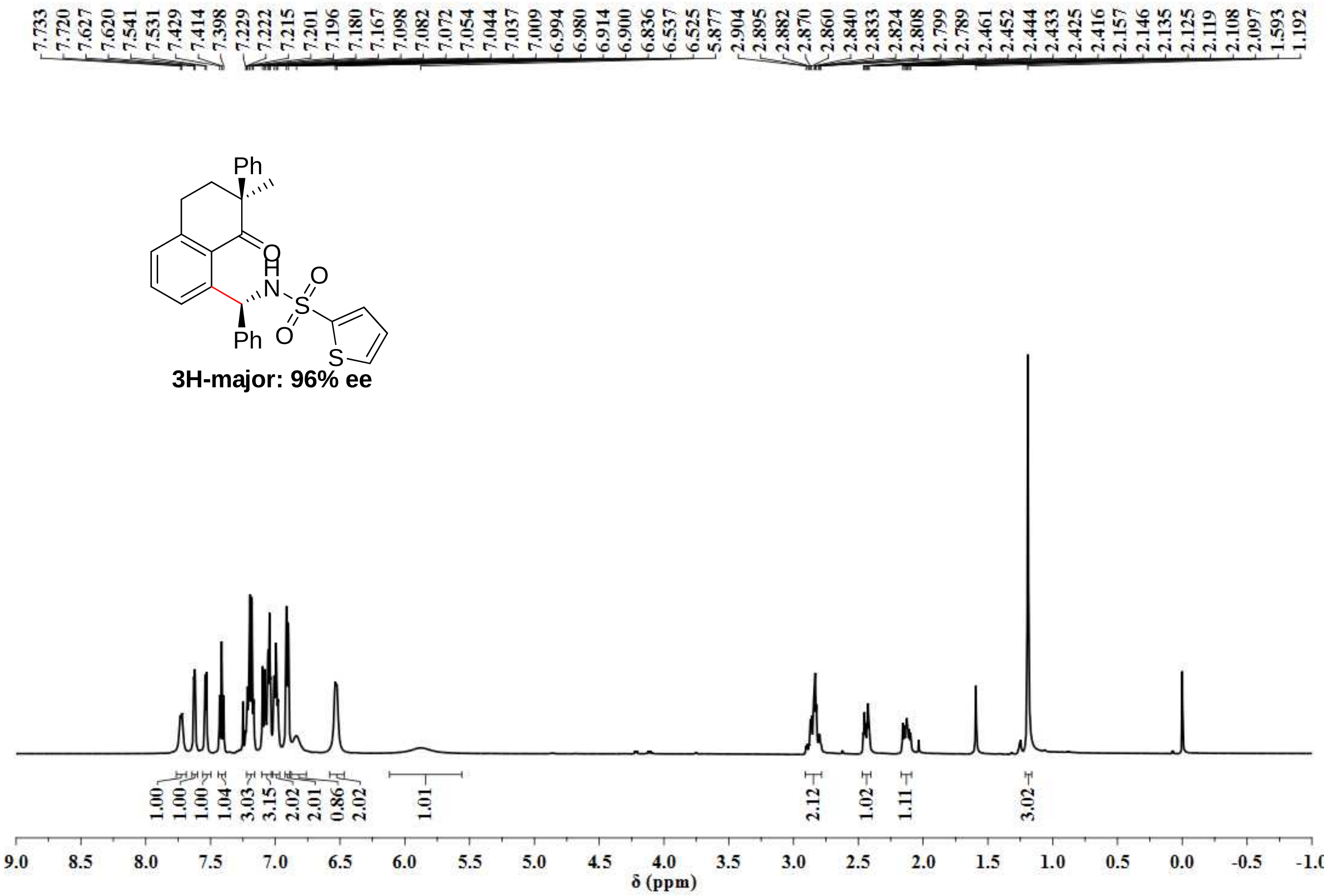

b

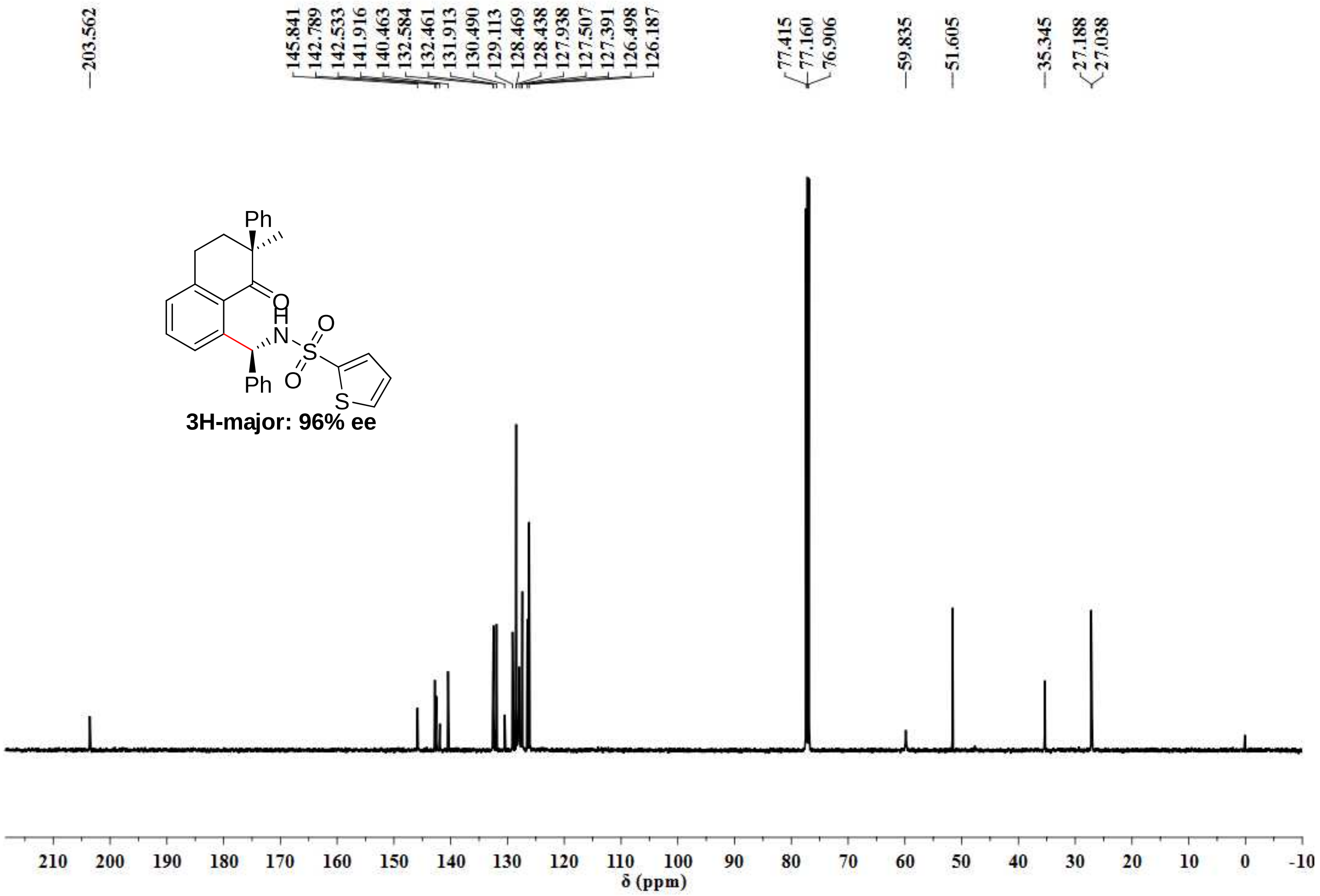
203.562
145.841
142.789
142.533
141.916
140.463
132.584
132.461
131.913
130.490
129.113
128.469
128.438
127.938
127.507
127.391
126.498
126.187
77.415
77.160
76.906
59.835
51.605
35.345
27.188
27.038
Ph
O
H
N
S
O
O
S
Ph
3H-major: 96% ee
210 200 190 180 170 160 150 140 130 120 110 100 90 80 70 60 50 40 30 20 10 0 -10
δ (ppm)

Supplementary Figure 63. Characterization of product 3H-minor. (a) ^{1}HNMR spectrum. (b) ^{13}C NMR spectrum.

a

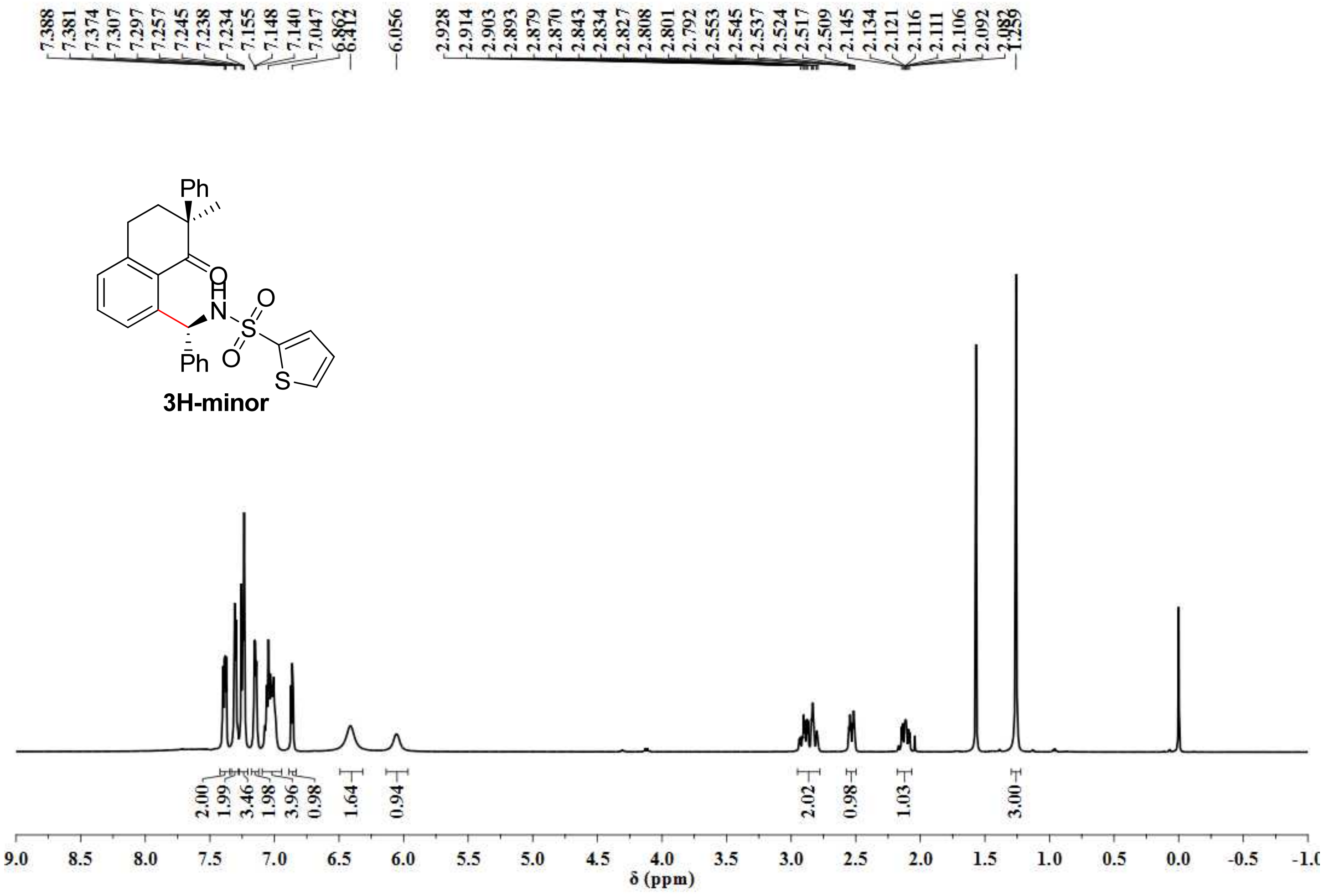

b

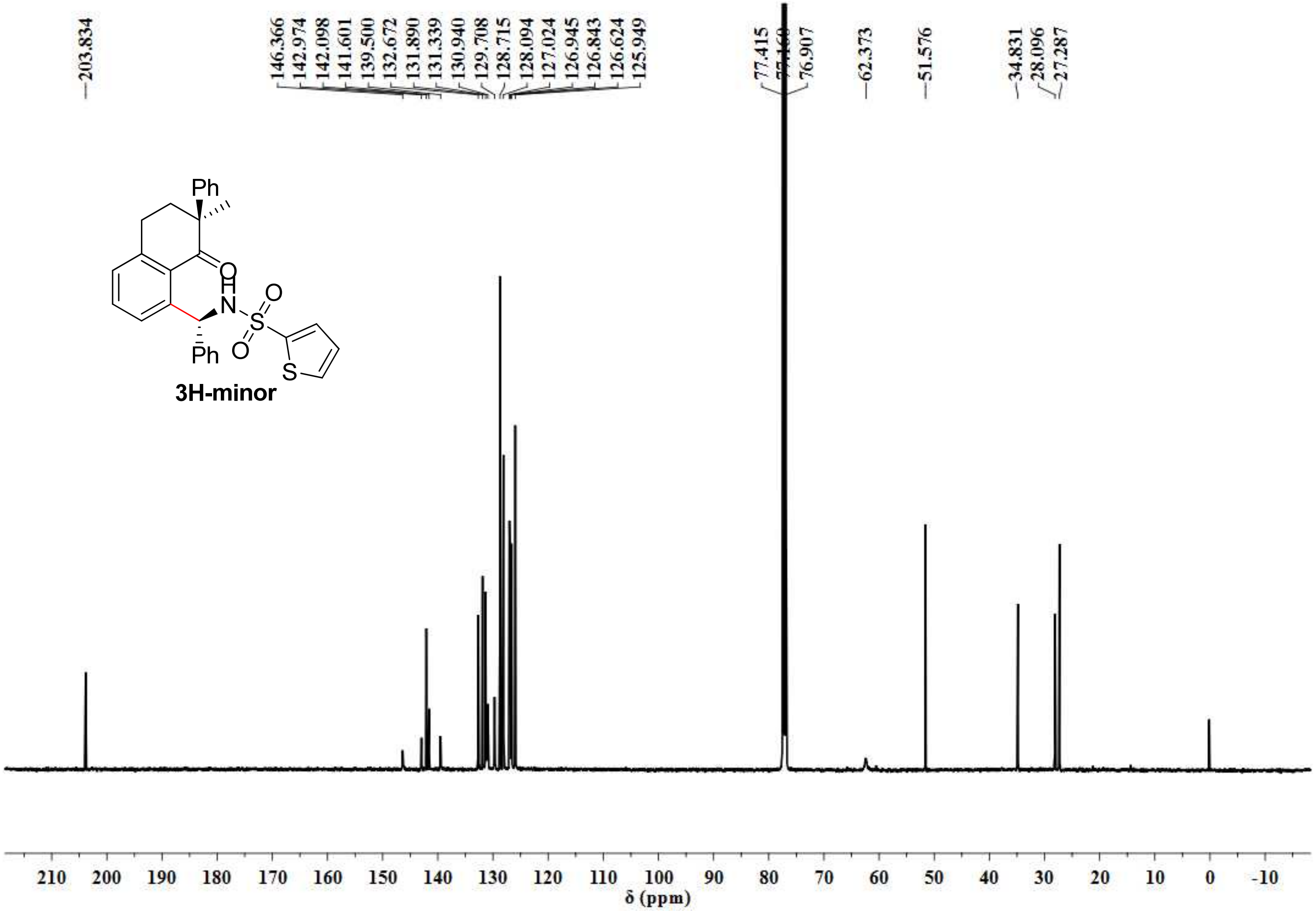

203.834
146.366
142.974
142.098
141.601
139.500
132.672
131.890
131.339
130.940
129.708
128.715
128.094
127.024
126.945
126.843
126.624
125.949
77.415
76.907
62.373
51.576
34.831
28.096
27.287
Ph
O
H
N
S
O
O
Ph
S
3H-minor
210 200 190 180 170 160 150 140 130 120 110 100 90 80 70 60 50 40 30 20 10 0 -10
δ (ppm)

Supplementary Figure 64. Characterization of product 4a. (a) ^{1}HNMR spectrum. (b) ^{13}C NMR spectrum.

a

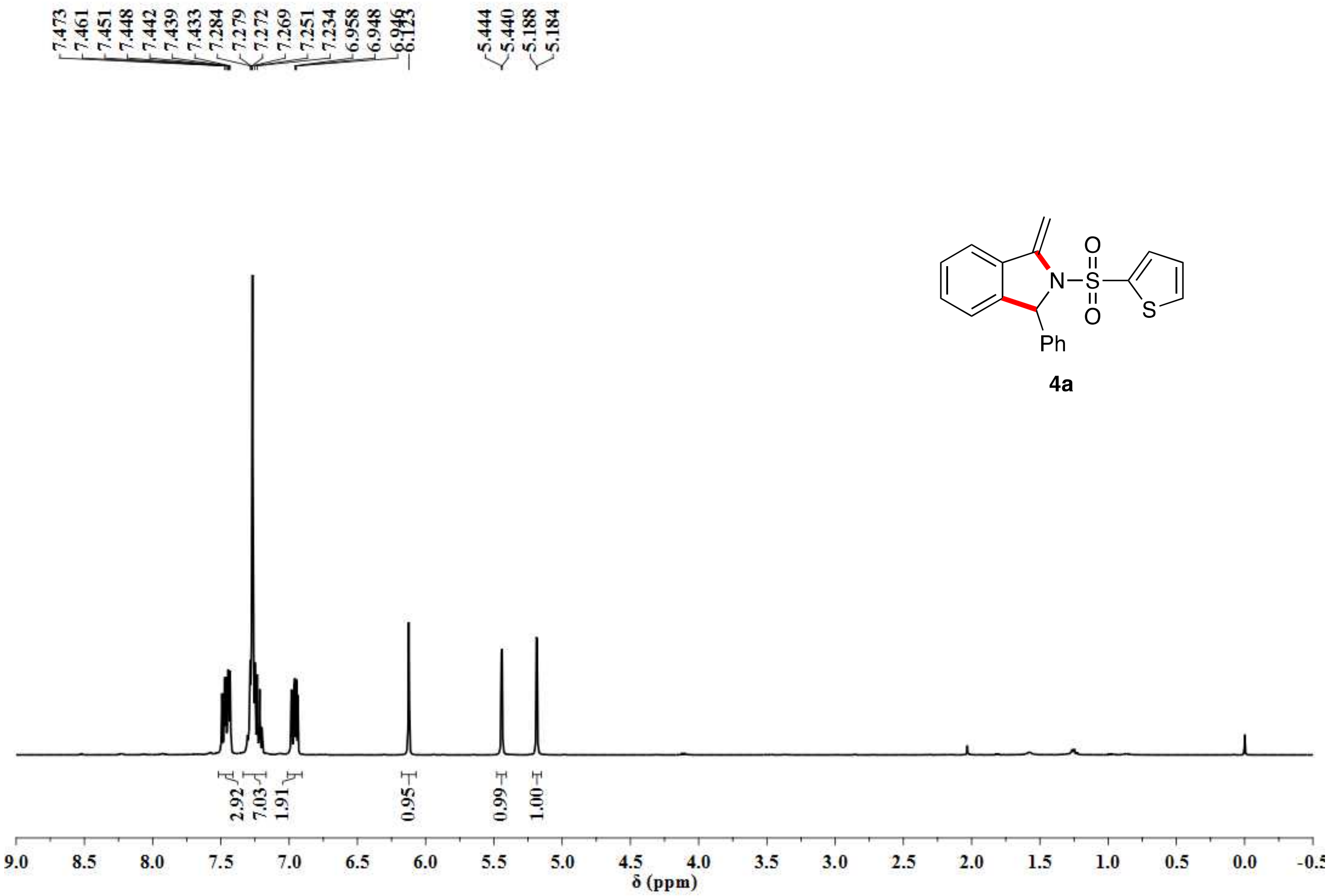

b

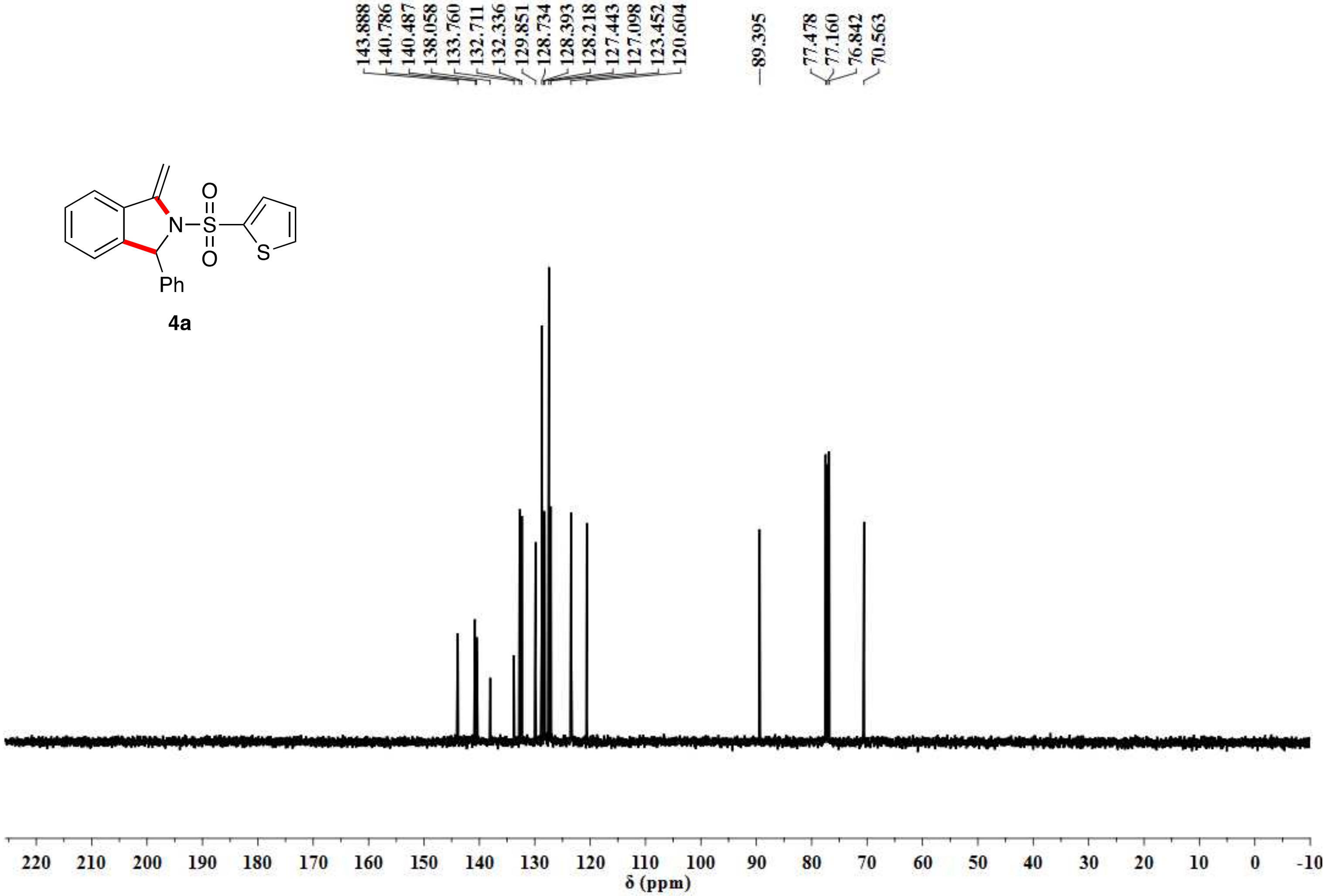

143.888
140.786
140.487
138.058
133.760
132.711
132.336
129.851
128.734
128.393
128.218
127.443
127.098
123.452
120.604
89.395
77.478
77.160
76.842
70.563
N
S
O
O
S
Ph
4a
220
210
200
190
180
170
160
150
140
130
120
110
100
90
80
70
60
50
40
30
20
10
0
-10
δ (ppm)

Supplementary Figure 65. Characterization of product 4b. (a) ^{1}HNMR spectrum. (b) ^{13}C NMR spectrum.

a

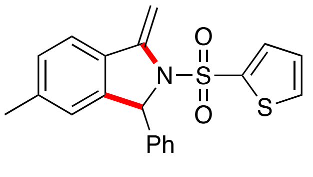

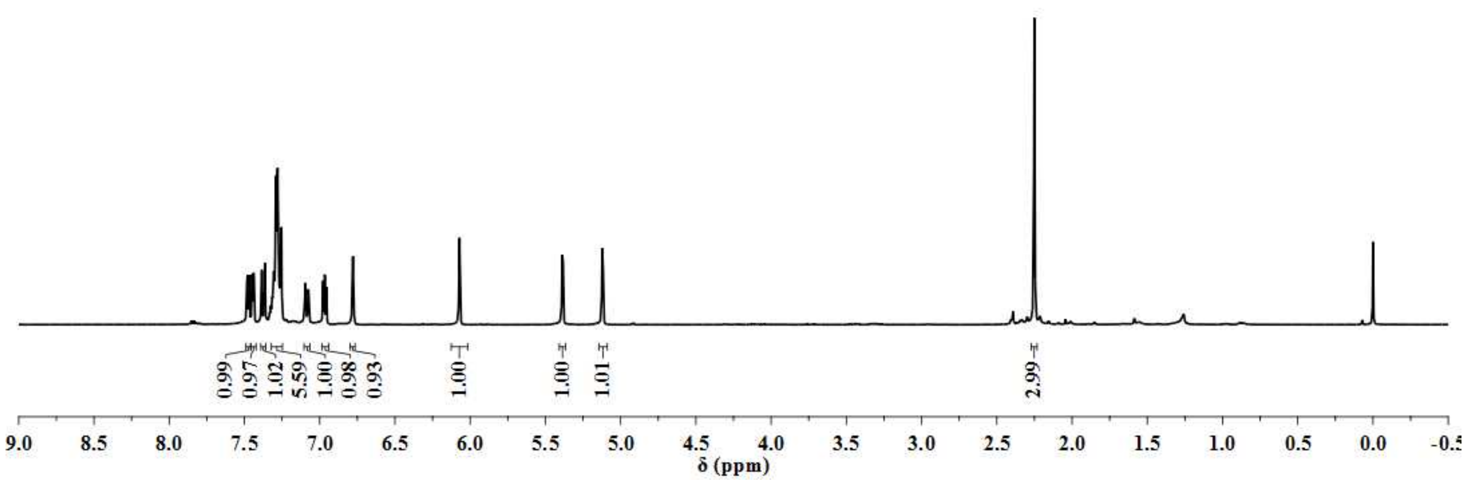

b

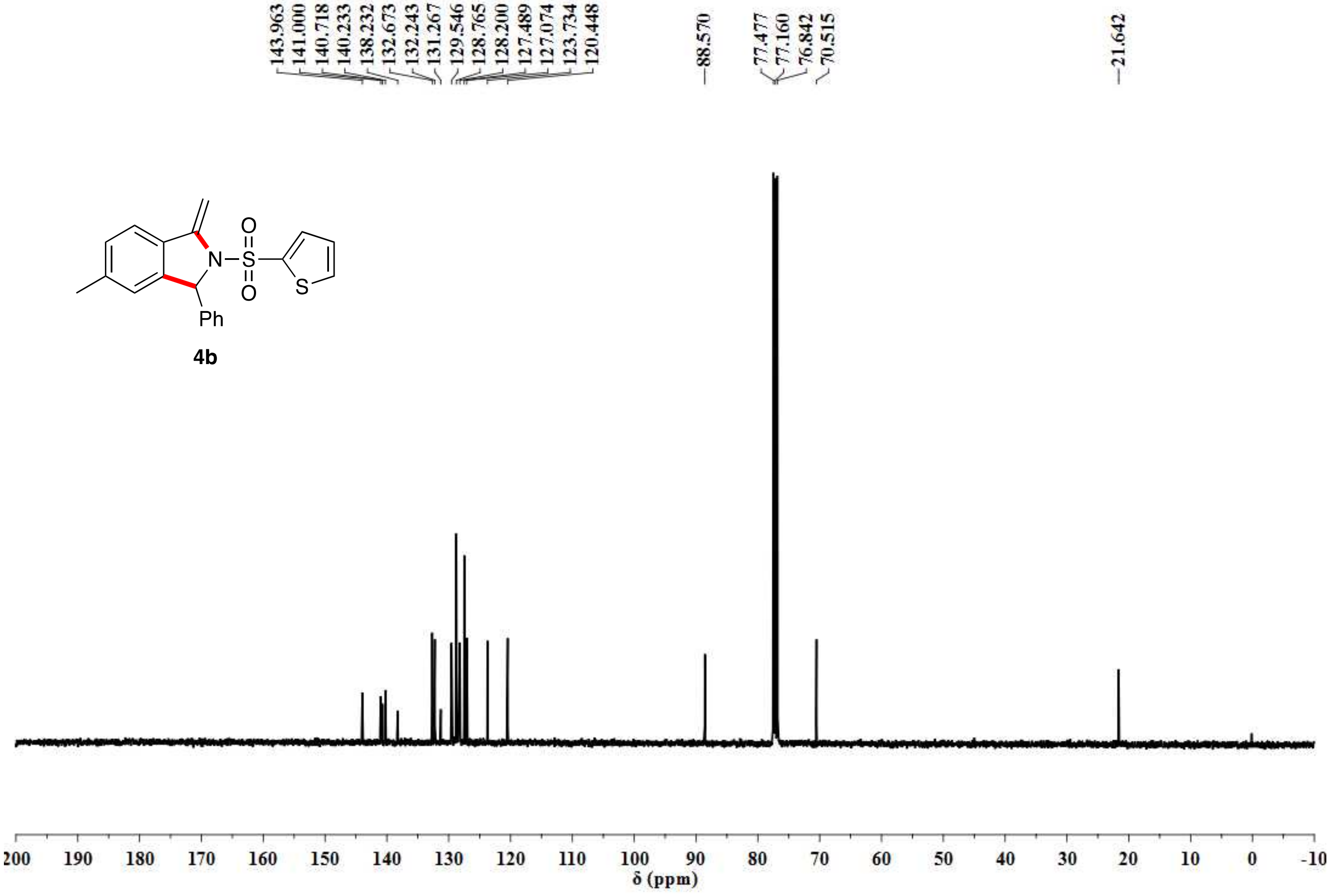
143.963
141.000
140.718
140.233
138.232
132.673
132.243
131.267
129.546
128.765
128.200
127.489
127.074
123.734
120.448
88.570
77.477
77.160
76.842
70.515
21.642
O
N
S
O
S
Ph
4b
200 190 180 170 160 150 140 130 120 110 100 90 80 70 60 50 40 30 20 10 0 -10
δ (ppm)

Supplementary Figure 66. Characterization of product 4c. (a) ^{1}HNMR spectrum. (b) ^{13}C NMR spectrum.

a

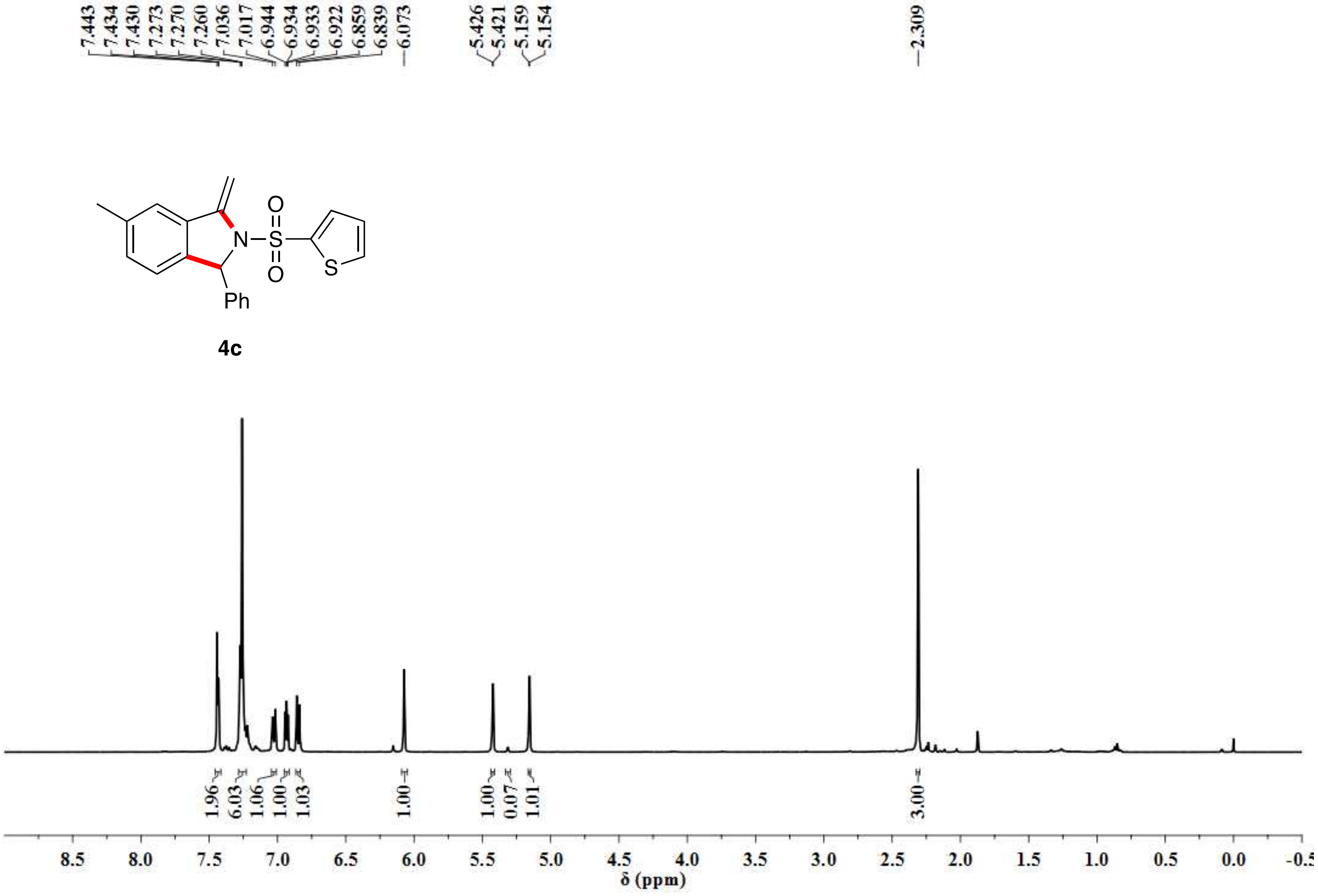

b

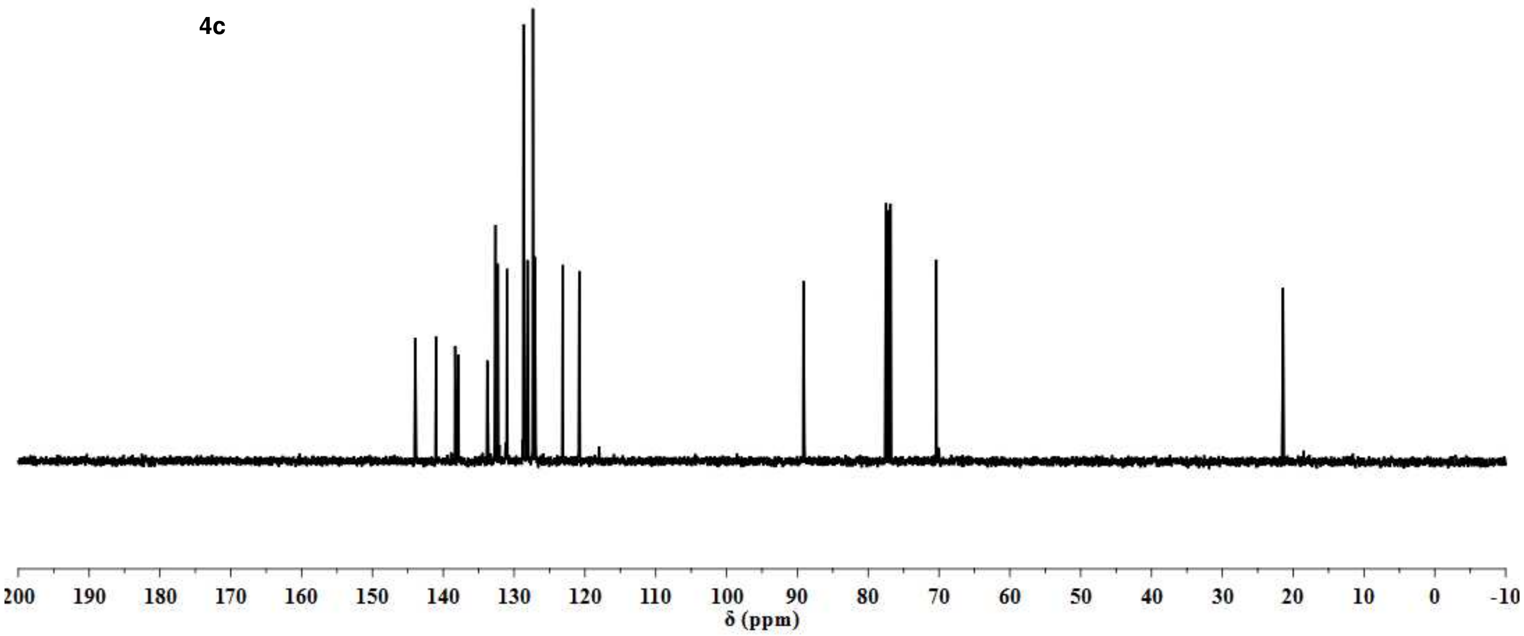
143.951
141.009
138.334
137.959
137.871
133.786
132.637
132.288
130.995
128.676
128.100
127.337
127.066
123.134
120.792
89.151
77.479
77.160
76.843
70.374
21.434
200 190 180 170 160 150 140 130 120 110 100 90 80 70 60 50 40 30 20 10 0 -10
δ (ppm)

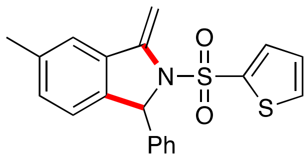
O
N
S
O
S
Ph

4c

Supplementary Figure 67. Characterization of product 4d. (a) ^{1}HNMR spectrum. (b) ^{13}C NMR spectrum.

a

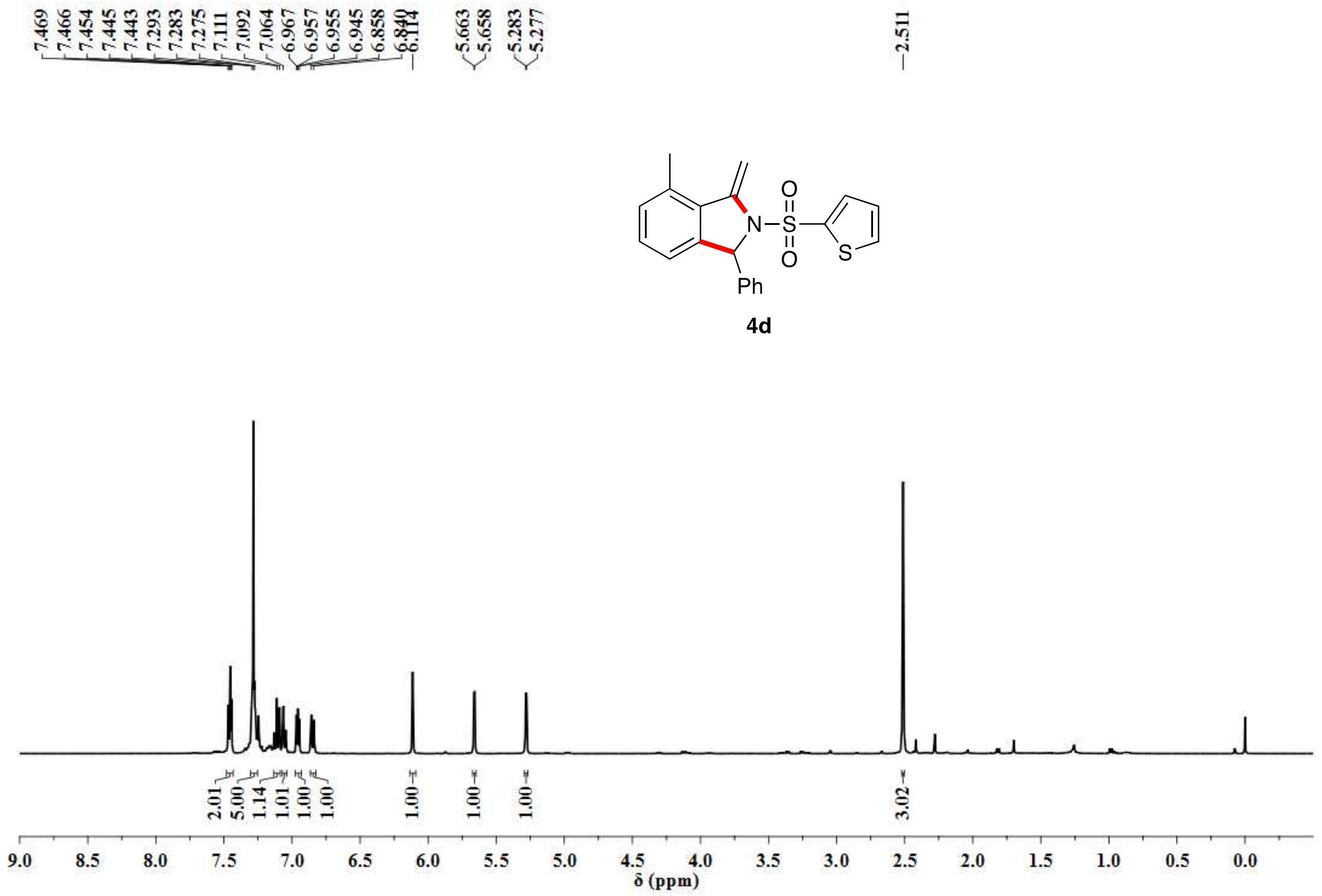

b

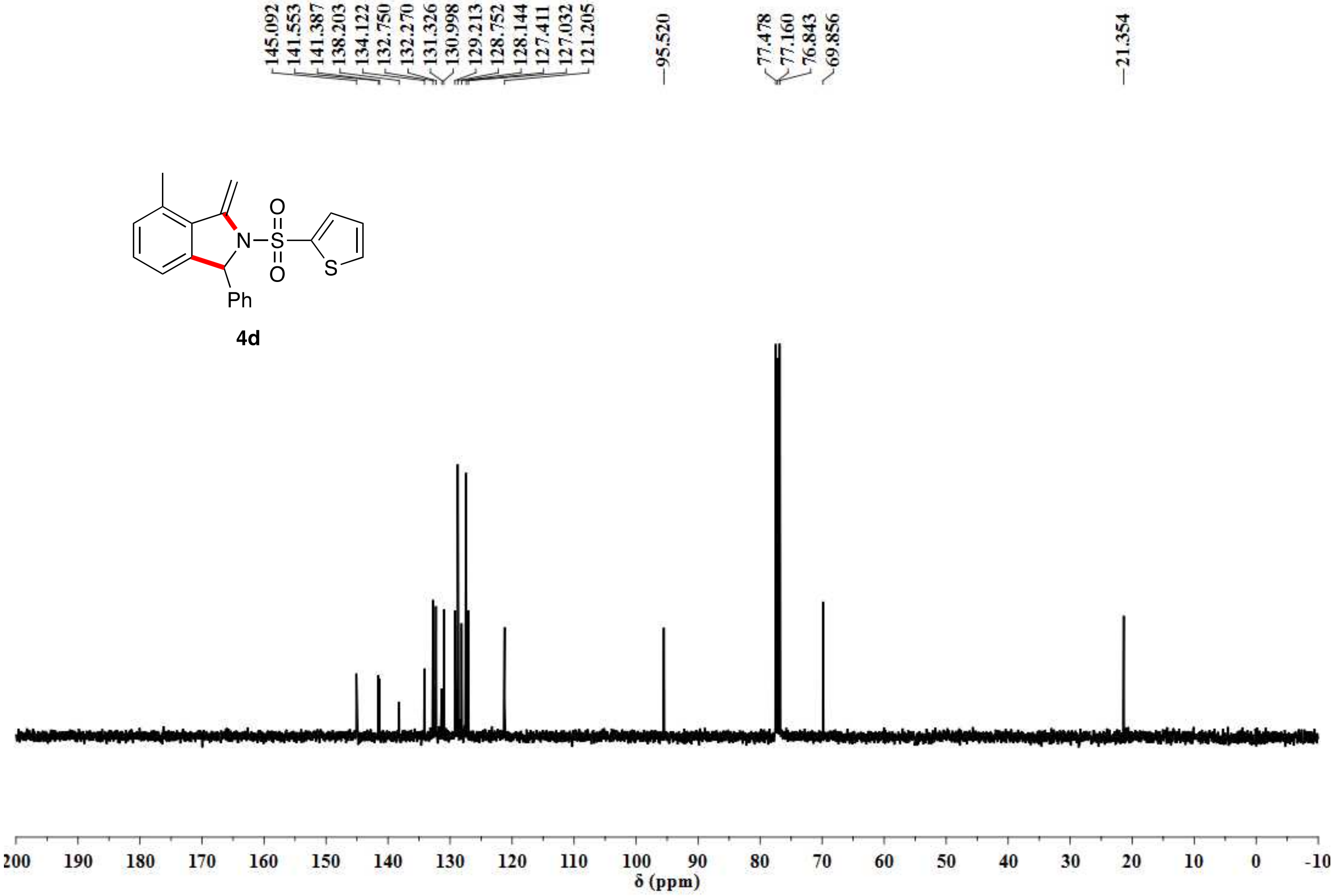
145.092
141.553
141.387
138.203
134.122
132.750
132.270
131.326
130.998
129.213
128.752
128.144
127.411
127.032
121.205
95.520
77.478
77.160
76.843
69.856
21.354
O
N
S
O
S
Ph
4d
200
190
180
170
160
150
140
130
120
110
100
90
80
70
60
50
40
30
20
10
0
-10
δ (ppm)

Supplementary Figure 68. Characterization of product 4e. (a) ^{1}HNMR spectrum. (b) ^{13}C NMR spectrum.

a

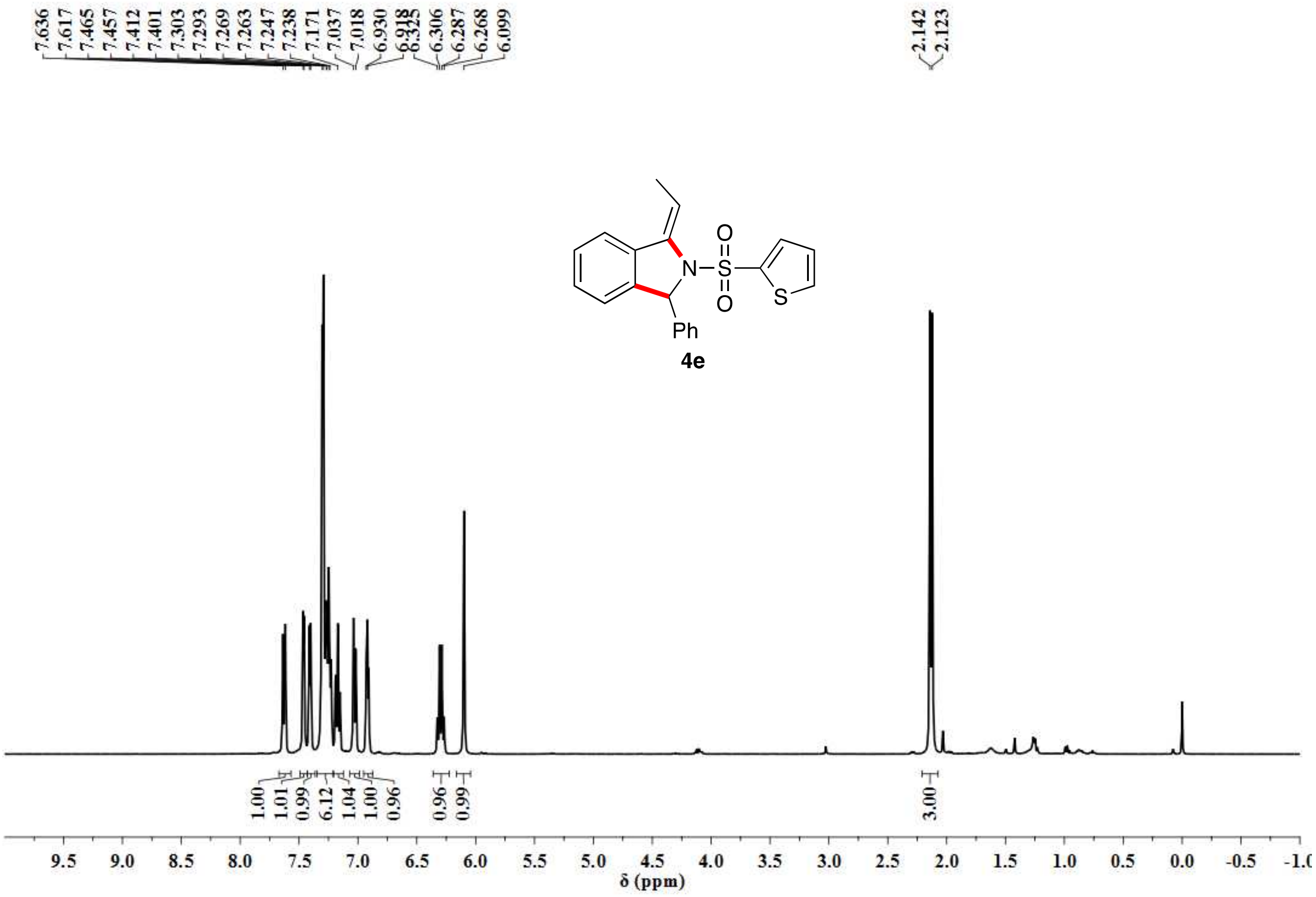

b

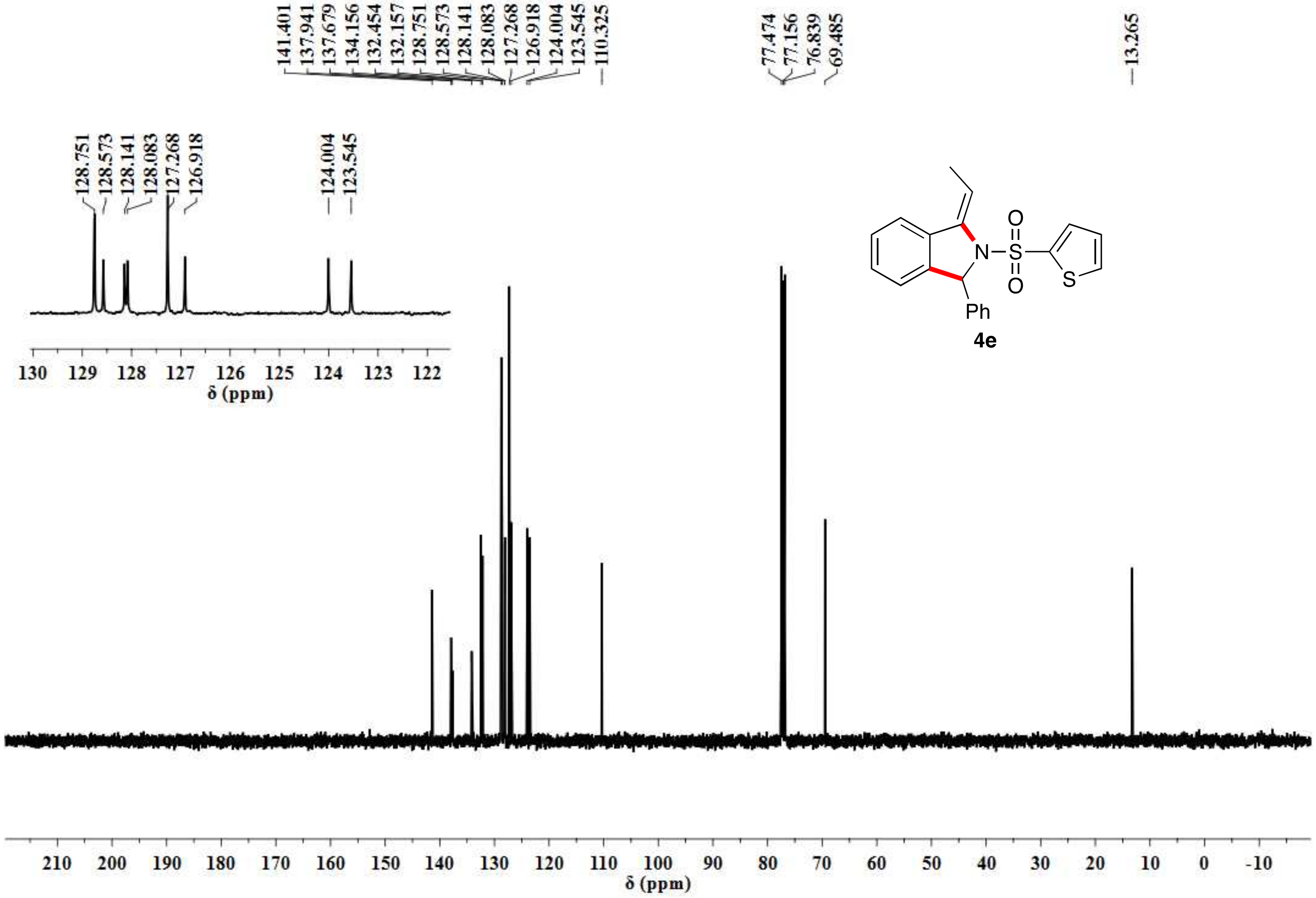
141.401
137.941
137.679
134.156
132.454
132.157
128.751
128.573
128.141
128.083
127.268
126.918
124.004
123.545
110.325
77.474
77.156
76.839
69.485
13.265
128.751
128.573
128.141
128.083
127.268
126.918
124.004
123.545
130 129 128 127 126 125 124 123 122
δ (ppm)
N
S
O
O
S
Ph
4e
210 200 190 180 170 160 150 140 130 120 110 100 90 80 70 60 50 40 30 20 10 0 -10
δ (ppm)

Supplementary Figure 69. Characterization of product 4f. (a) ^{1}HNMR spectrum. (b) ^{13}C NMR spectrum.

a

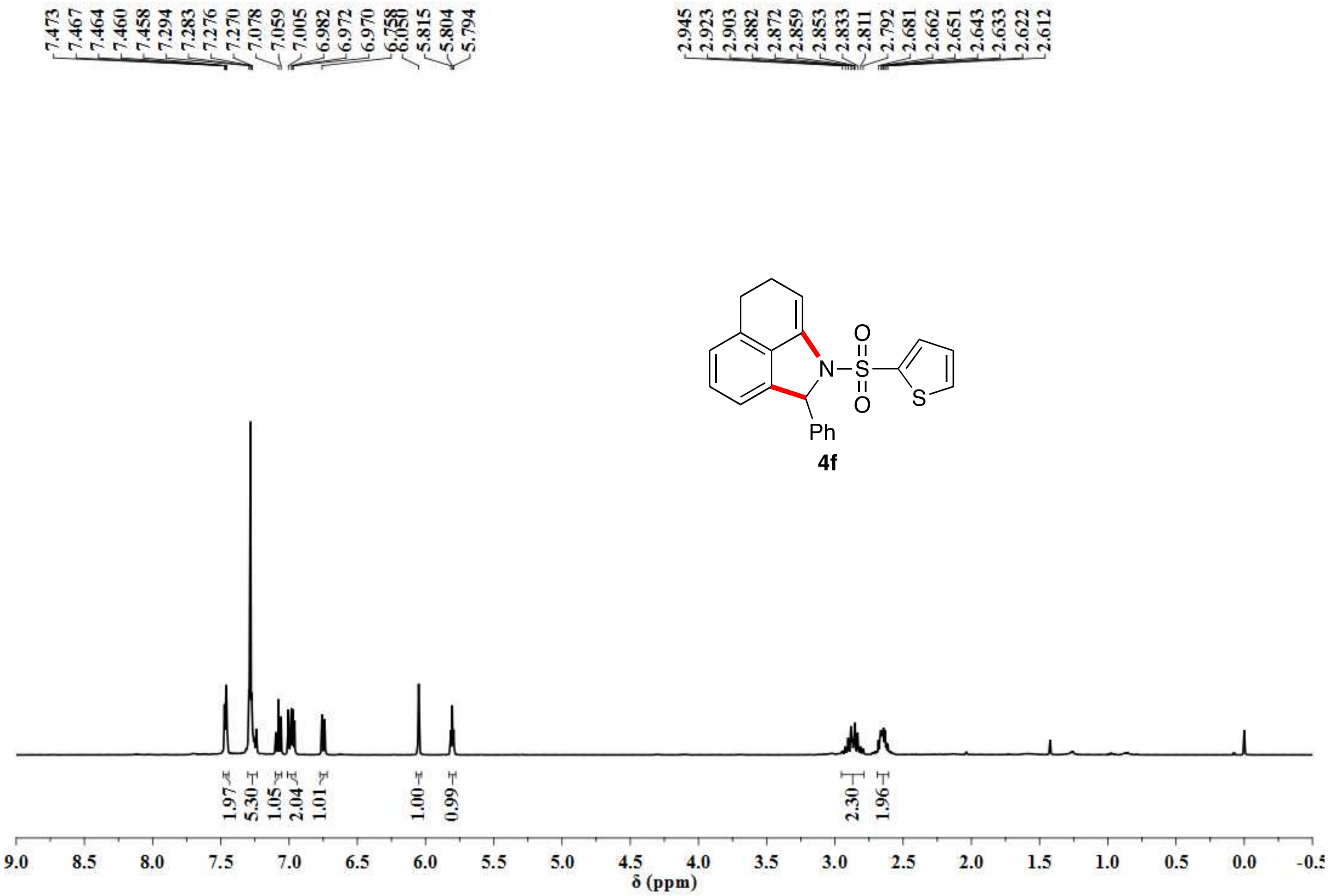

b

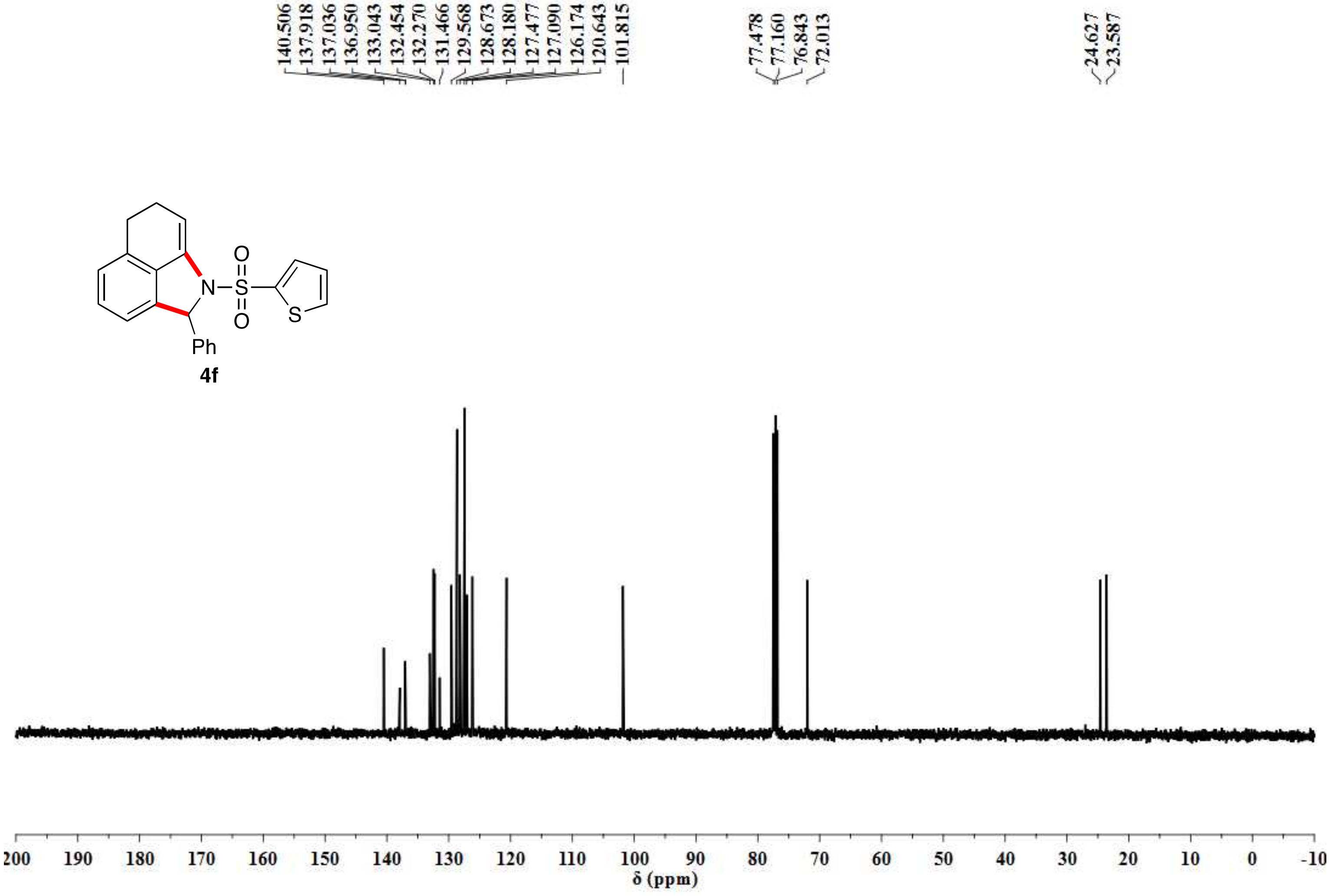
140.506
137.918
137.036
136.950
133.043
132.454
132.270
131.466
129.568
128.673
128.180
127.477
127.090
126.174
120.643
101.815
77.478
77.160
76.843
72.013
24.627
23.587
O
N
S
O
S
Ph
4f
200 190 180 170 160 150 140 130 120 110 100 90 80 70 60 50 40 30 20 10 0 -10
δ (ppm)

Supplementary Figure 70. Characterization of product 4g. (a) ^{1}HNMR spectrum. (b) ^{13}C NMR spectrum.

a

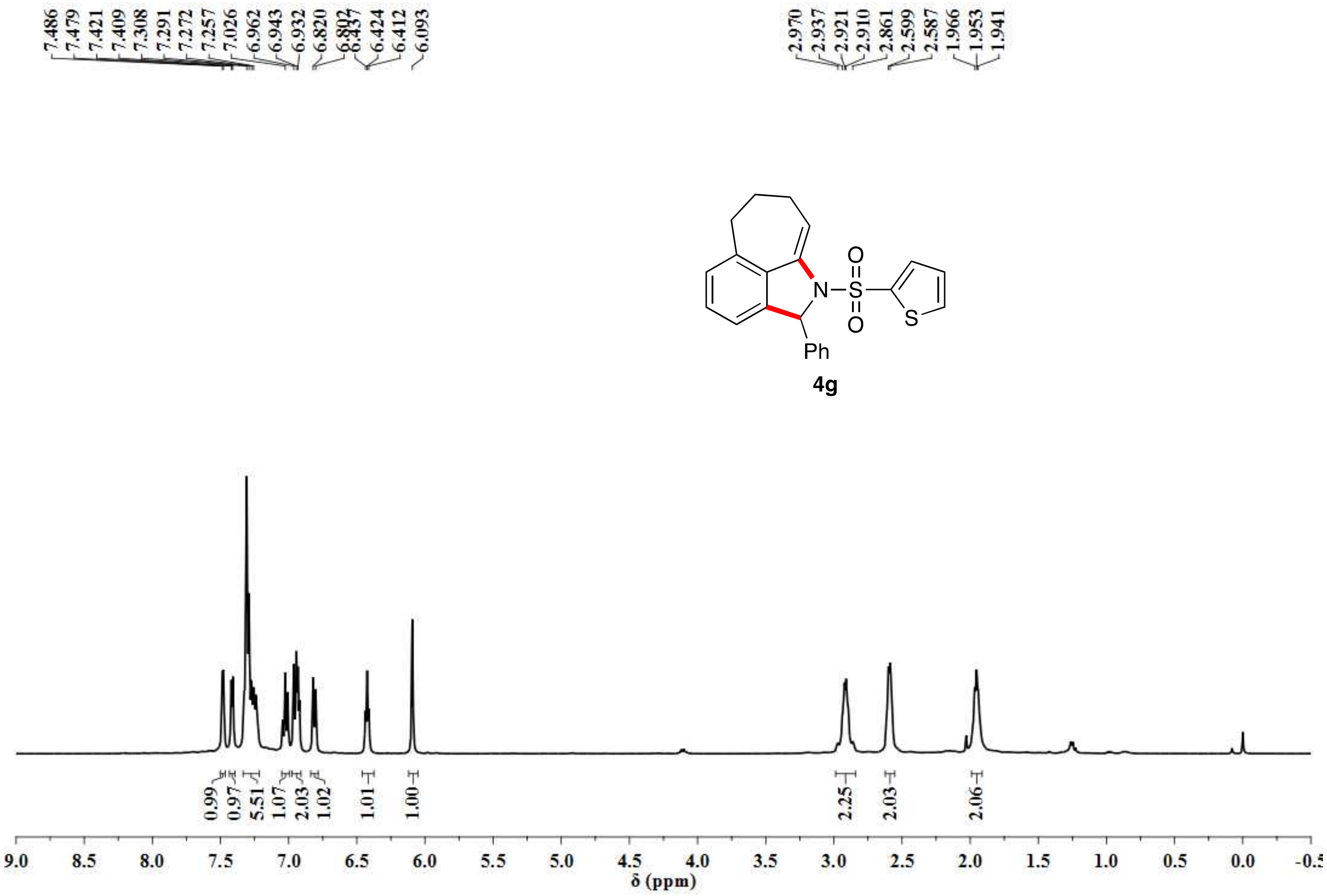

b

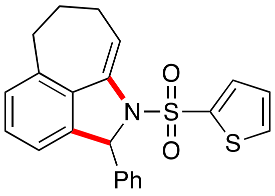

4g

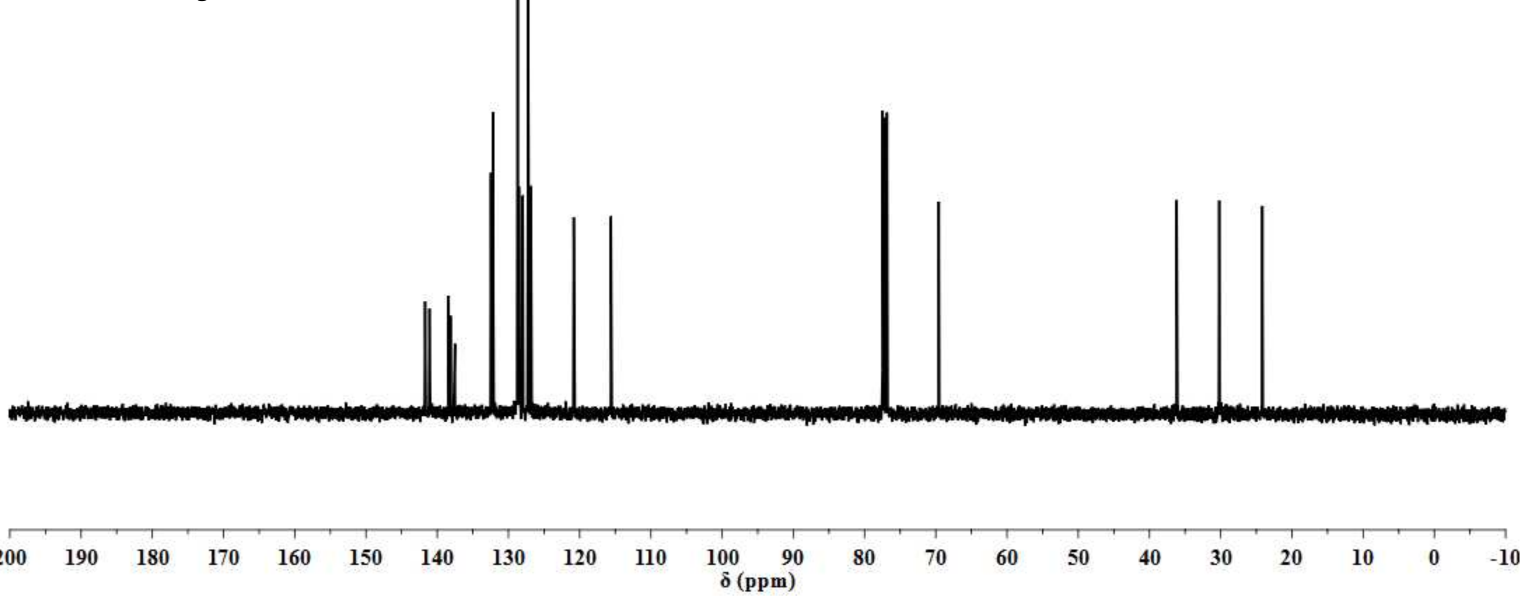

141.700
141.093
138.396
138.127
137.516
132.460
132.161
128.707
128.517
128.473
127.979
127.238
126.839
120.831
115.604
77.478
77.160
76.843
69.576
36.178
30.211
24.153
200 190 180 170 160 150 140 130 120 110 100 90 80 70 60 50 40 30 20 10 0 -10
δ (ppm)

Supplementary Figure 71. Characterization of product 4h. (a) ^{1}HNMR spectrum. (b) ^{13}C NMR spectrum.

a

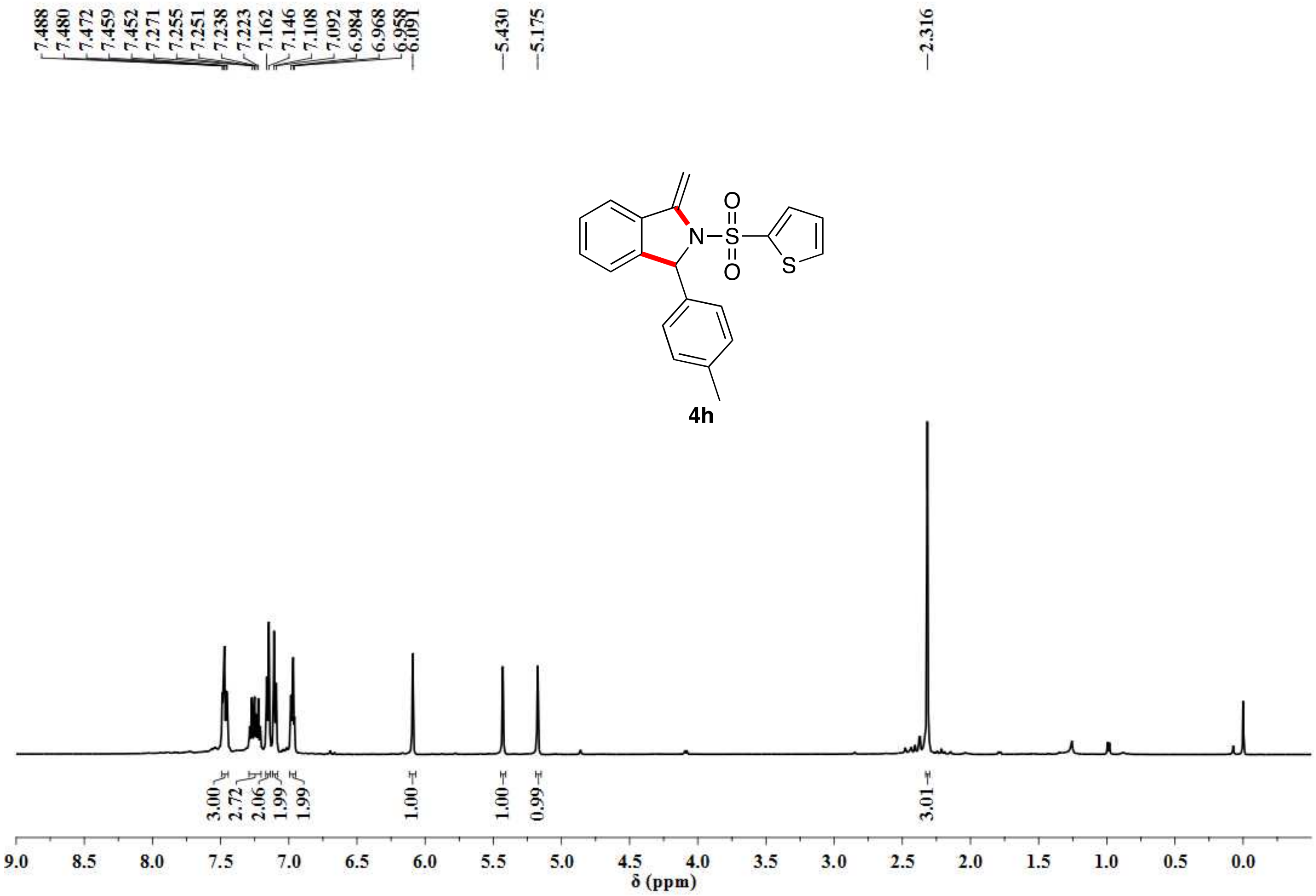

b

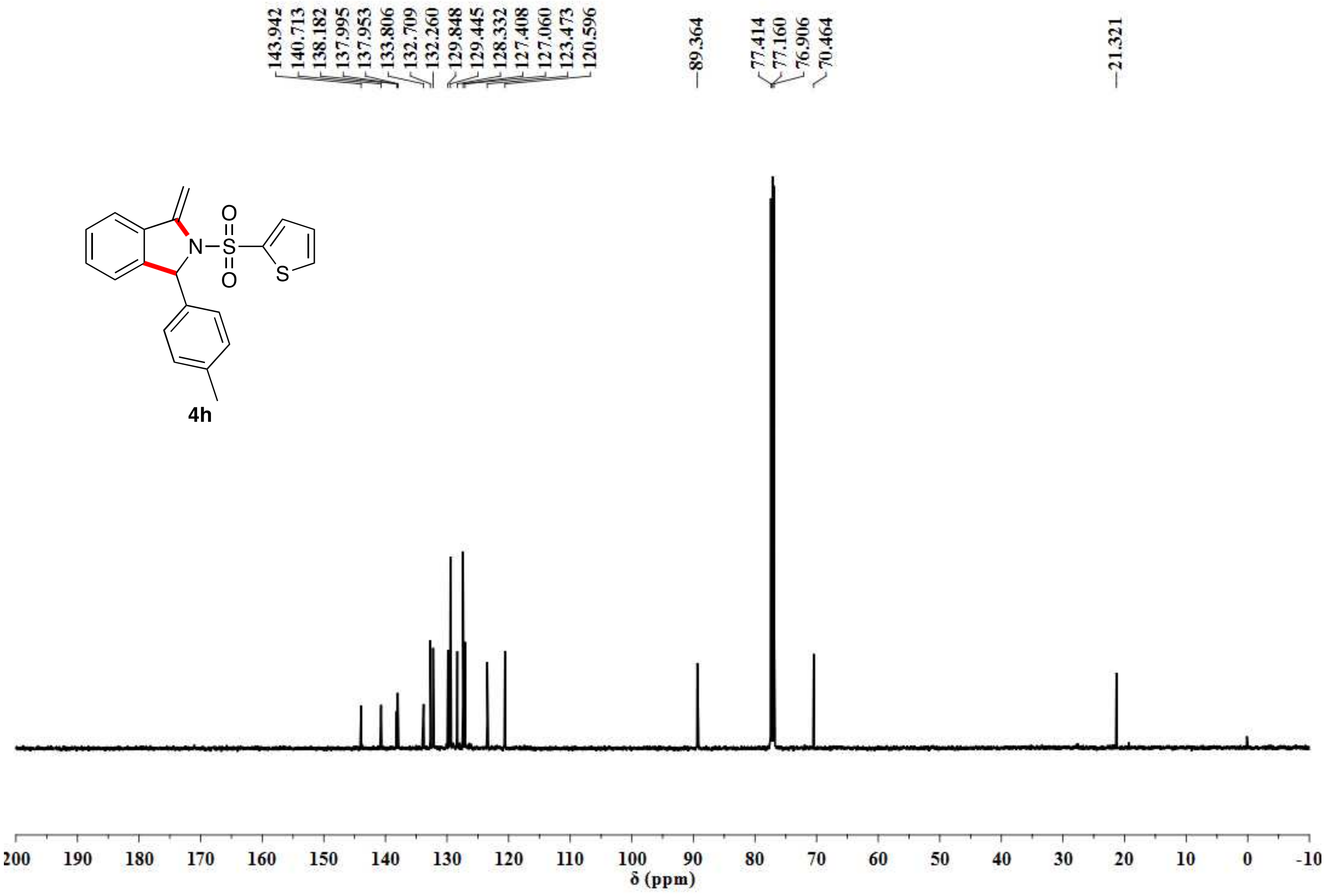
143.942
140.713
138.182
137.995
137.953
133.806
132.709
132.260
129.848
129.445
128.332
127.408
127.060
123.473
120.596
89.364
77.414
77.160
76.906
70.464
21.321
4h
200 190 180 170 160 150 140 130 120 110 100 90 80 70 60 50 40 30 20 10 0 -10
δ (ppm)

Supplementary Figure 72. Characterization of product 4i. (a) ^{1}HNMR spectrum. (b) ^{13}C NMR spectrum.

a

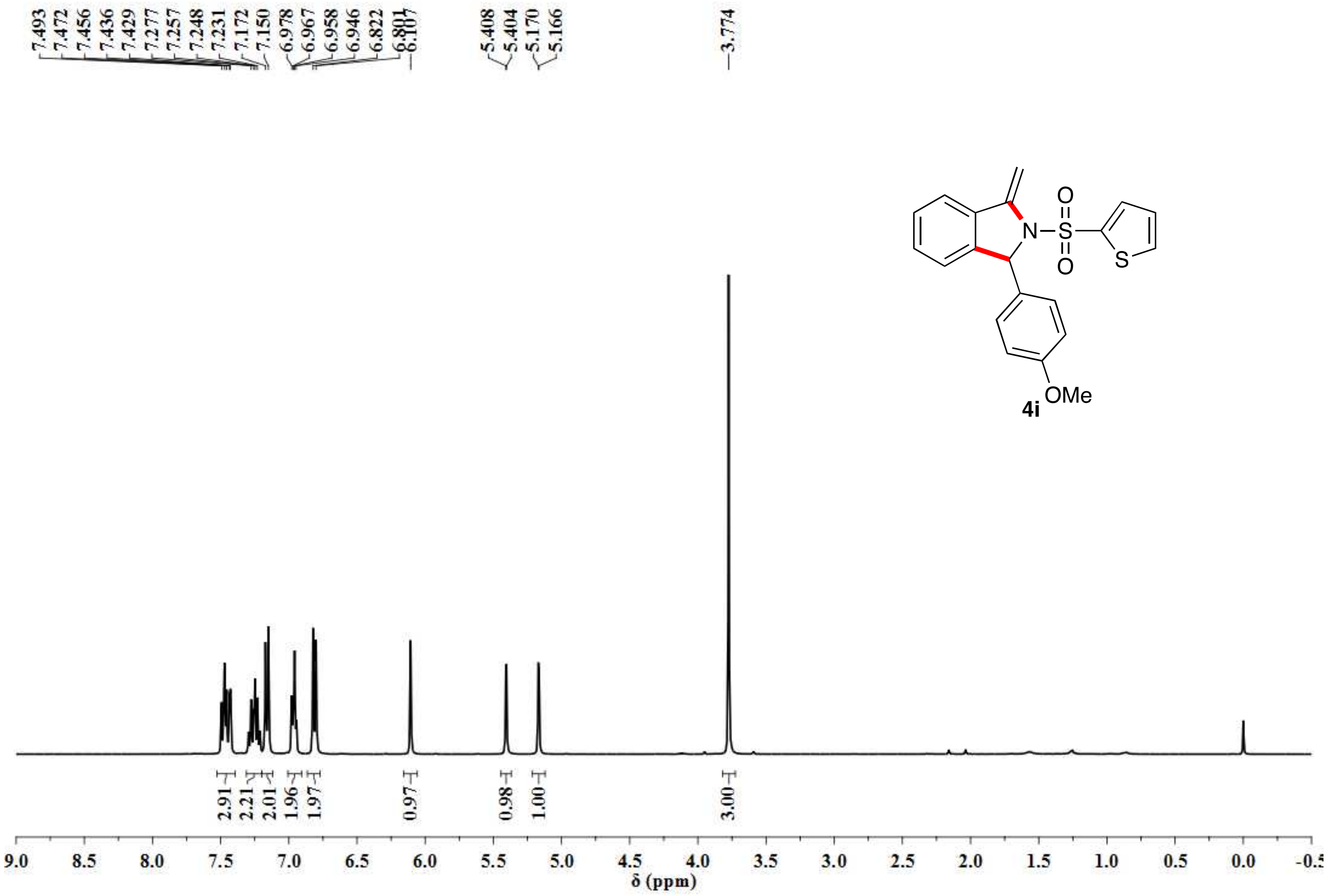

b

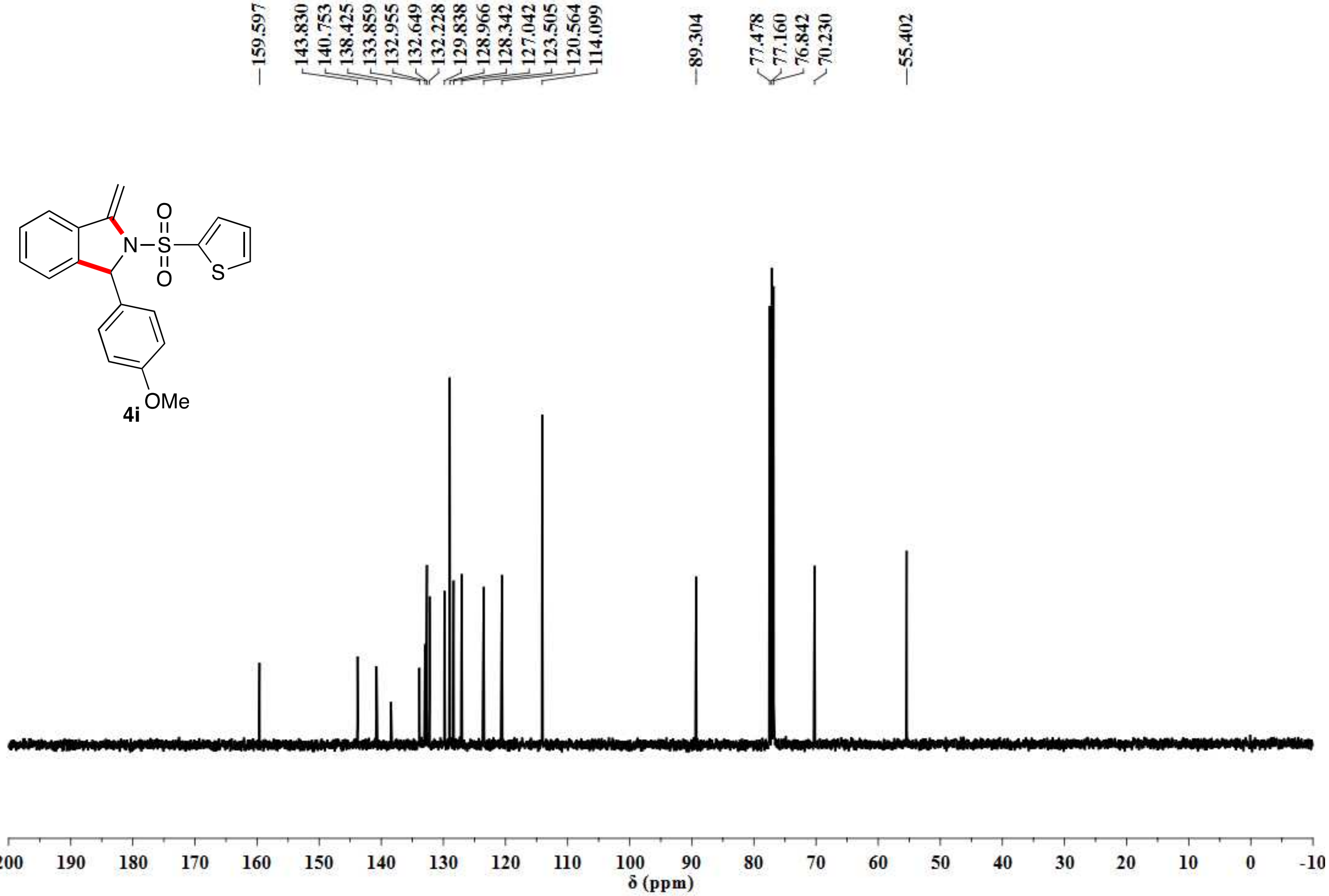

159.597
143.830
140.753
138.425
133.859
132.955
132.649
132.228
129.838
128.966
128.342
127.042
123.505
120.564
114.099
89.304
77.478
77.160
76.842
70.230
55.402
O
N
S
O
S
OMe
4i
200
190
180
170
160
150
140
130
120
110
100
90
80
70
60
50
40
30
20
10
0
-10
δ (ppm)

Supplementary Figure 73. Characterization of product 4j. (a) ^{1}HNMR spectrum. (b) ^{13}C NMR spectrum. (c) ^{19}F NMR spectrum

a

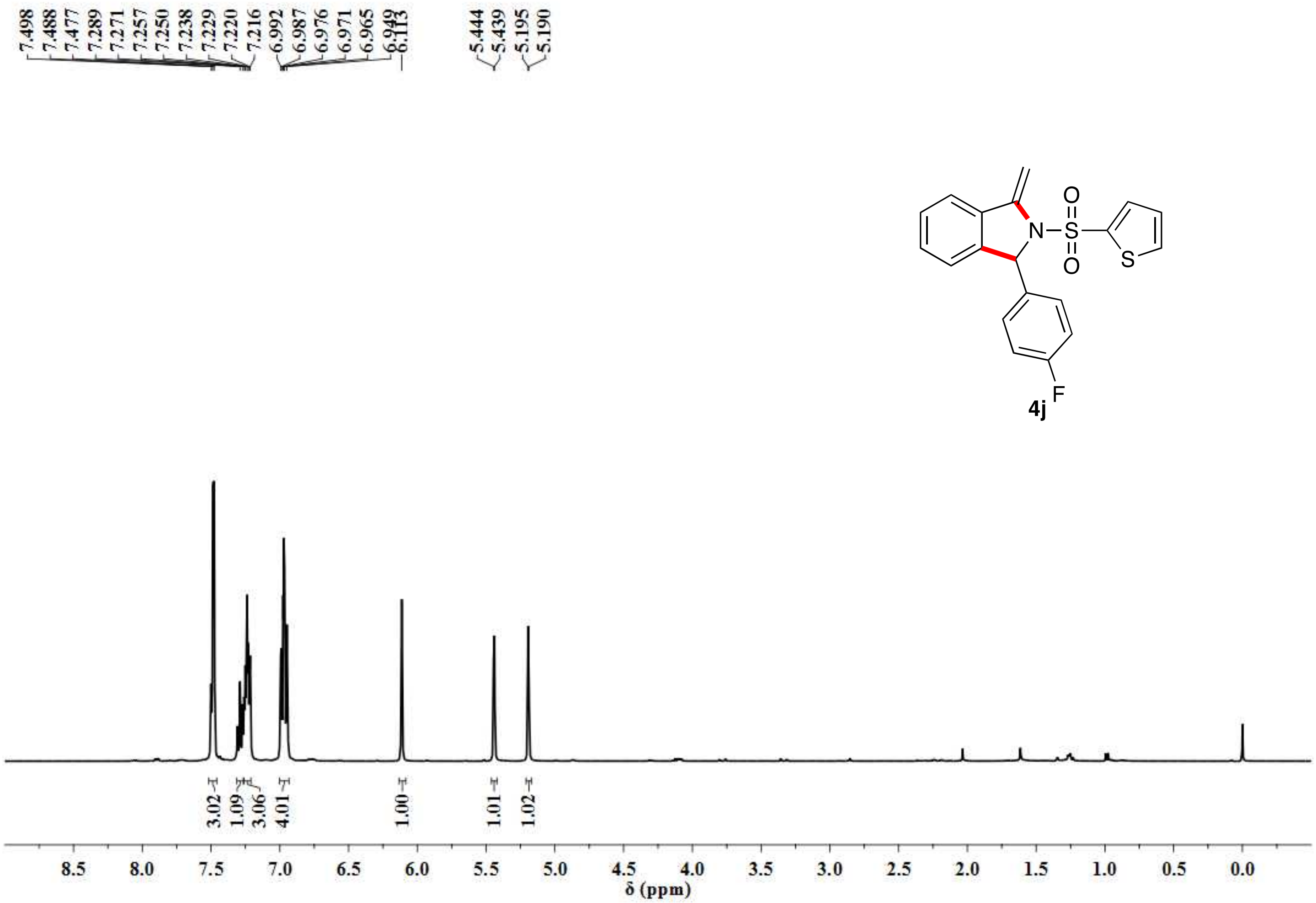

b

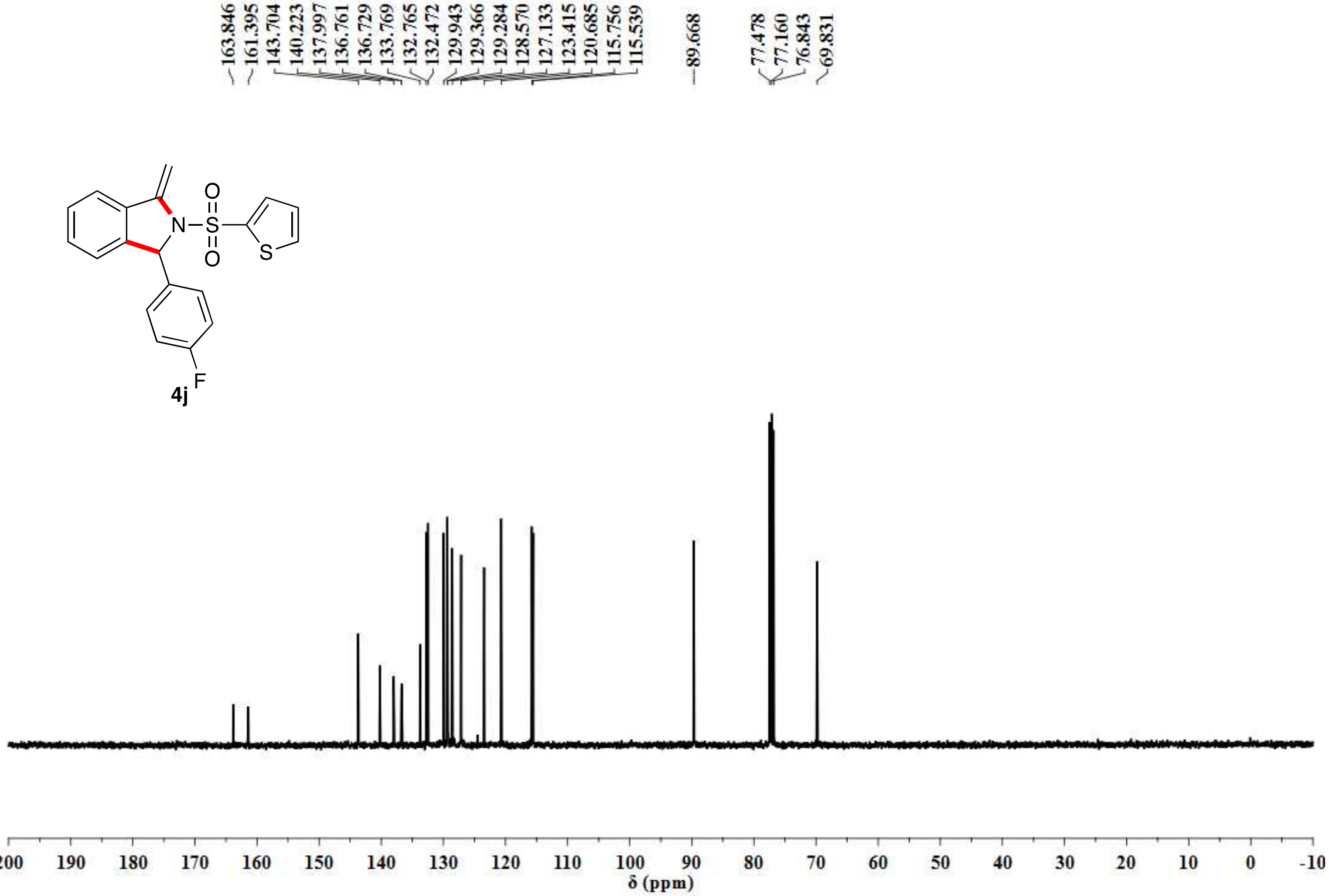
4j
163.846
161.395
143.704
140.223
137.997
136.761
136.729
133.769
132.765
132.472
129.943
129.366
129.284
128.570
127.133
123.415
120.685
115.756
115.539
89.668
77.478
77.160
76.843
69.831
200 190 180 170 160 150 140 130 120 110 100 90 80 70 60 50 40 30 20 10 0 -10
δ (ppm)

c

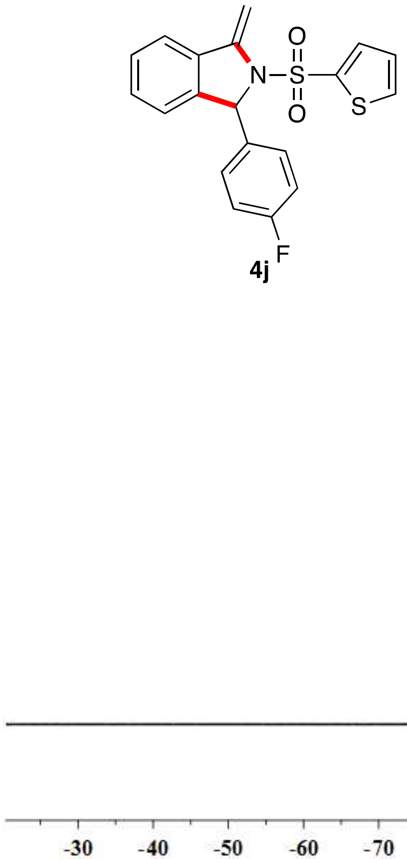

4j

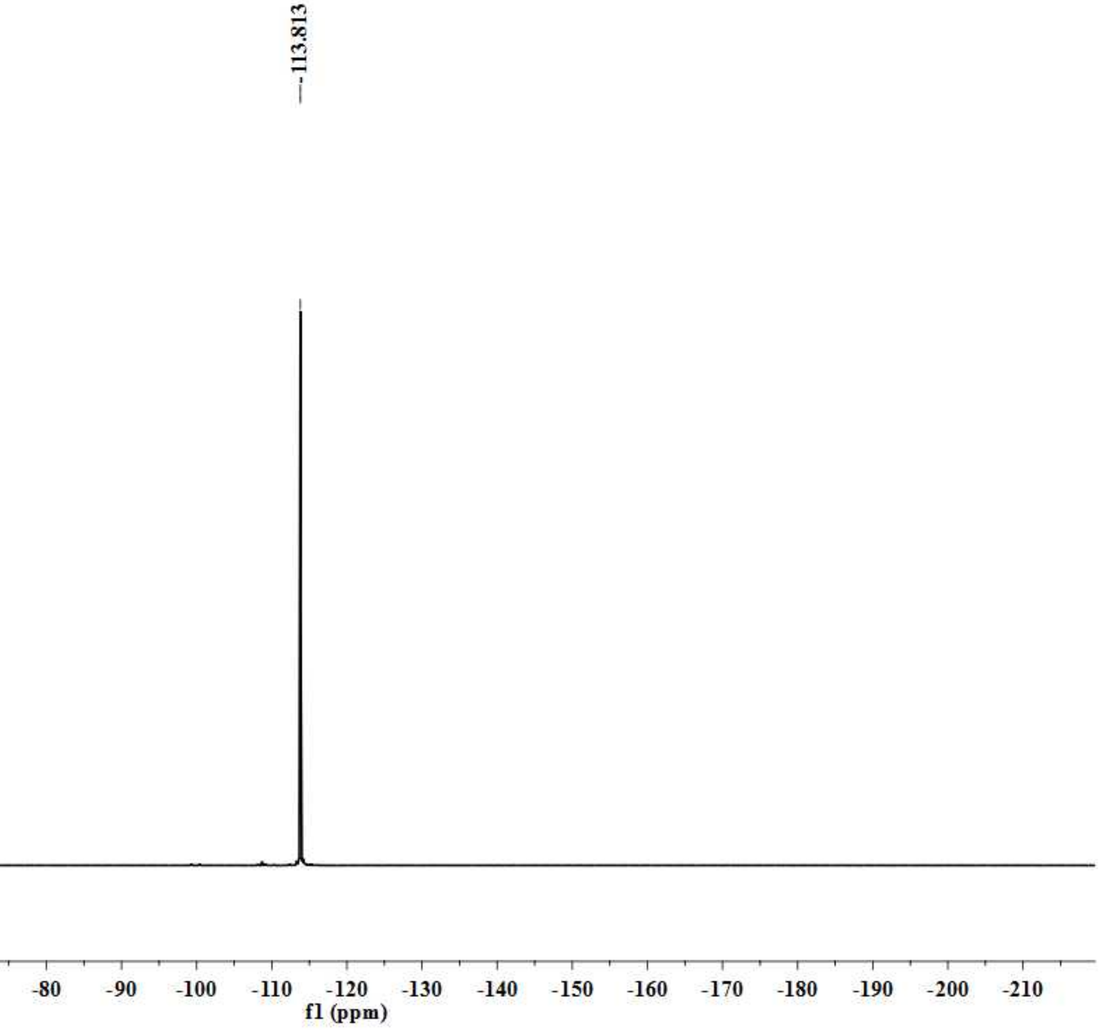
-113.813
-30
-40
-50
-60
-70
-80
-90
-100
-110
-120
-130
-140
-150
-160
-170
-180
-190
-200
-210
f1 (ppm)

Supplementary Figure 74. Characterization of product 4k. (a) ^{1}HNMR spectrum. (b) ^{13}C NMR spectrum.

a

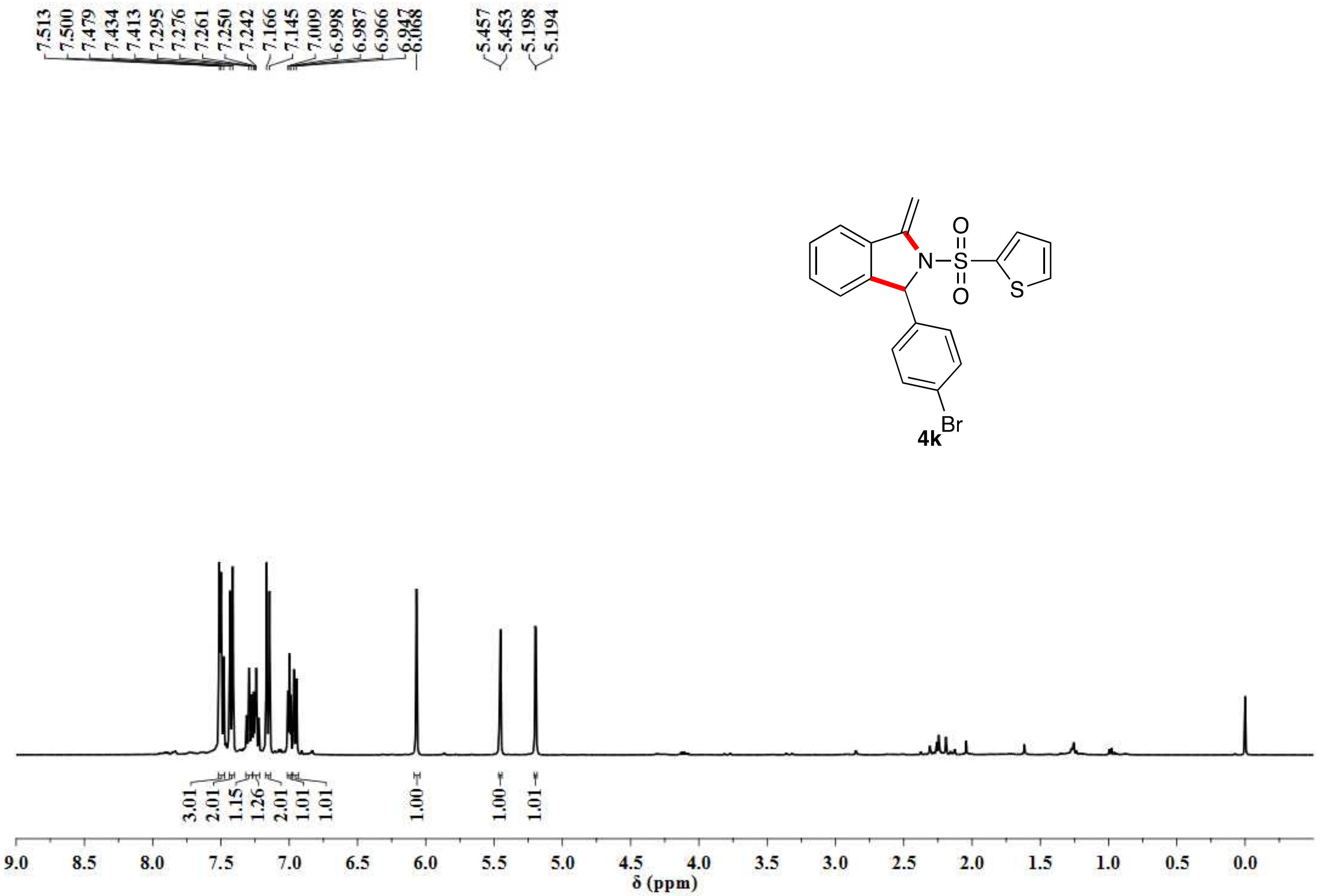

b

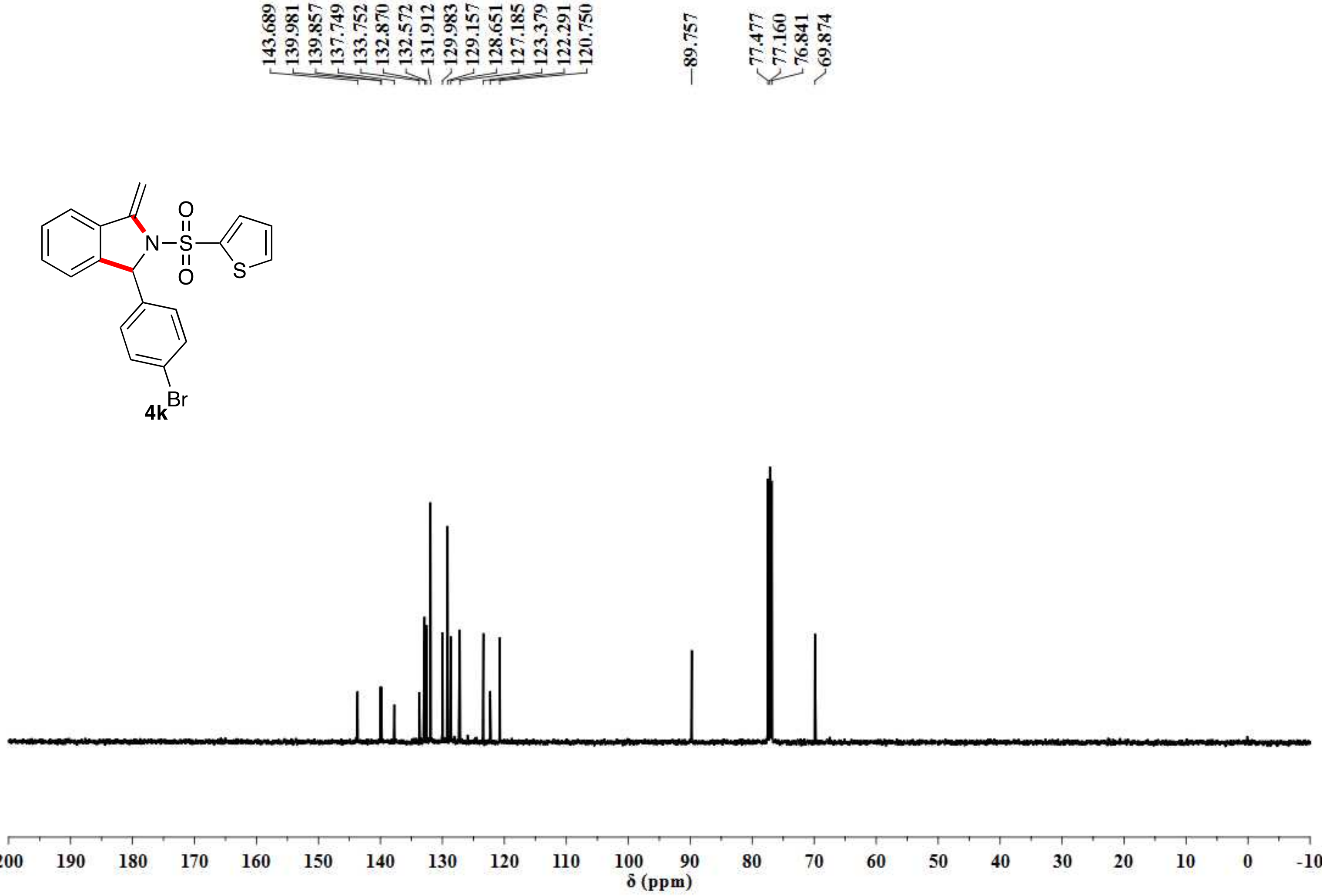
143.689
139.981
139.857
137.749
133.752
132.870
132.572
131.912
129.983
129.157
128.651
127.185
123.379
122.291
120.750
89.757
77.477
77.160
76.841
69.874
O
N
S
O
S
Br
4k
200 190 180 170 160 150 140 130 120 110 100 90 80 70 60 50 40 30 20 10 0 -10
δ (ppm)

Supplementary Figure 75. Characterization of product 4l. (a) ^{1}HNMR spectrum. (b) ^{13}C NMR spectrum.

a

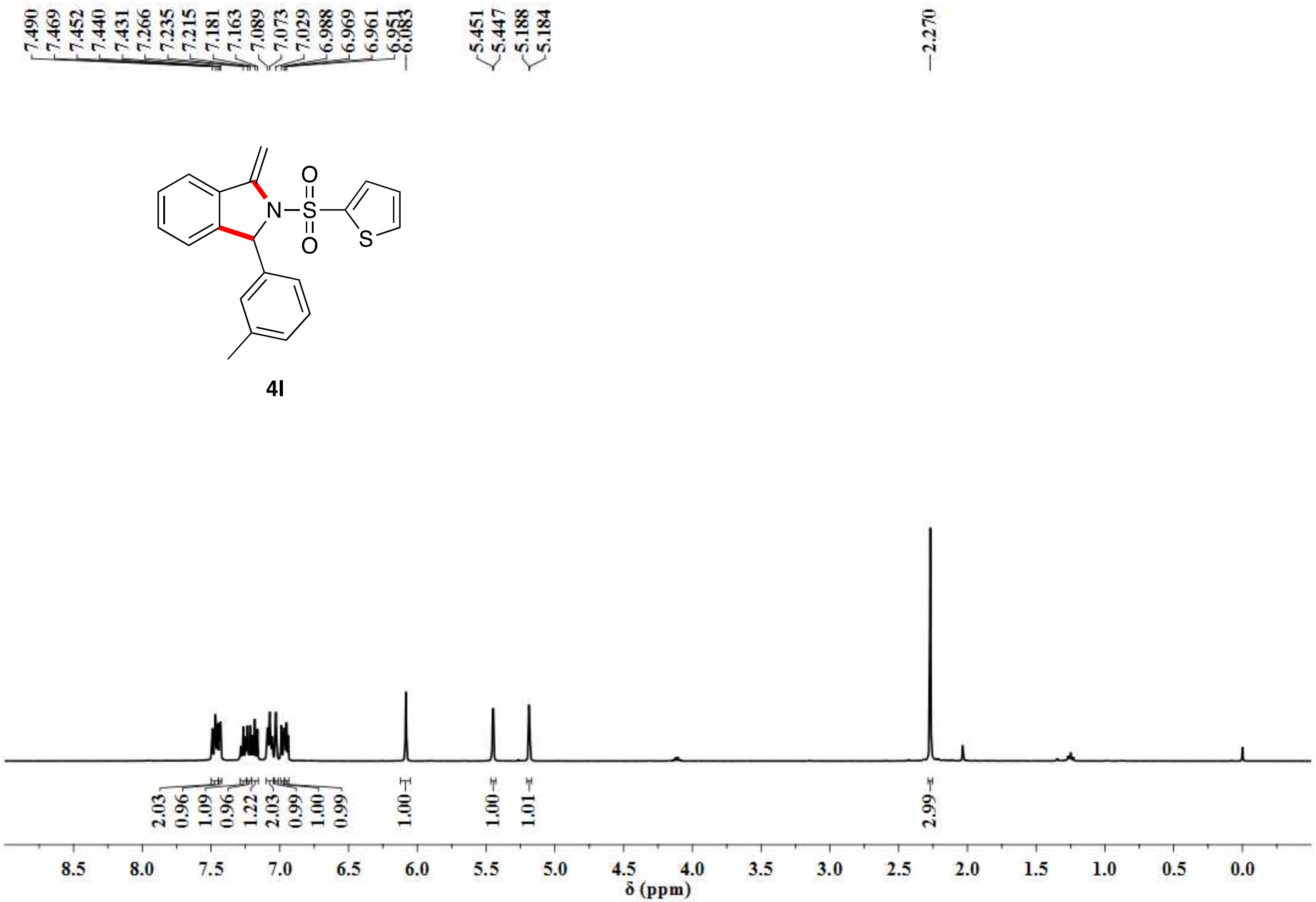

b

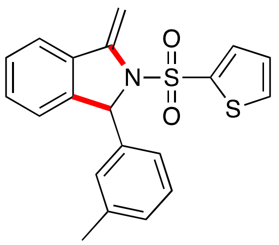

O
N
S
O
S

4l

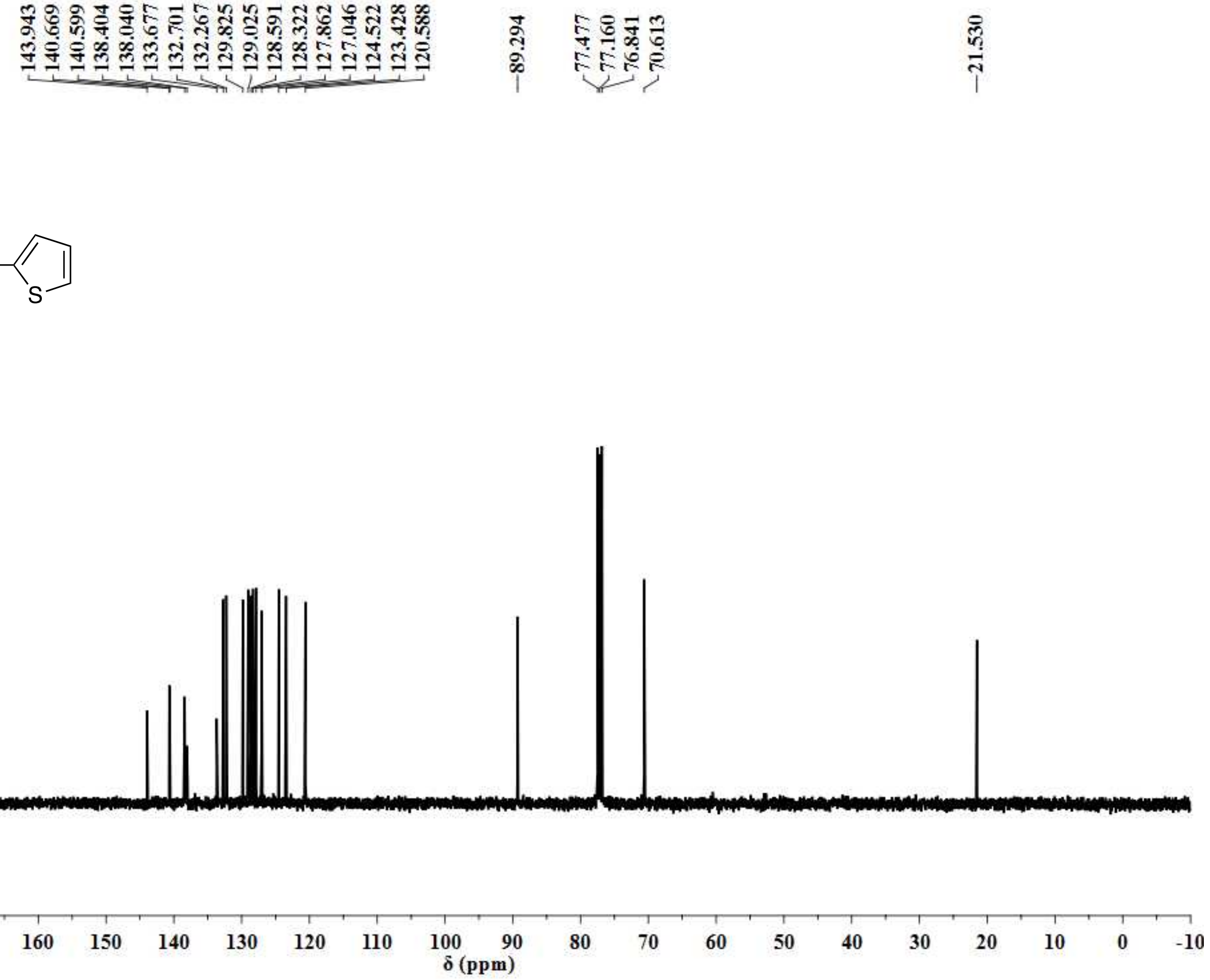

143.943
140.669
140.599
138.404
138.040
133.677
132.701
132.267
129.825
129.025
128.591
128.322
127.862
127.046
124.522
123.428
120.588
89.294
77.477
77.160
76.841
70.613
21.530
200 190 180 170 160 150 140 130 120 110 100 90 80 70 60 50 40 30 20 10 0 -10
δ (ppm)

Supplementary Figure 76. Characterization of product 4m. (a) ^{1}HNMR spectrum. (b) ^{13}C NMR spectrum.

a

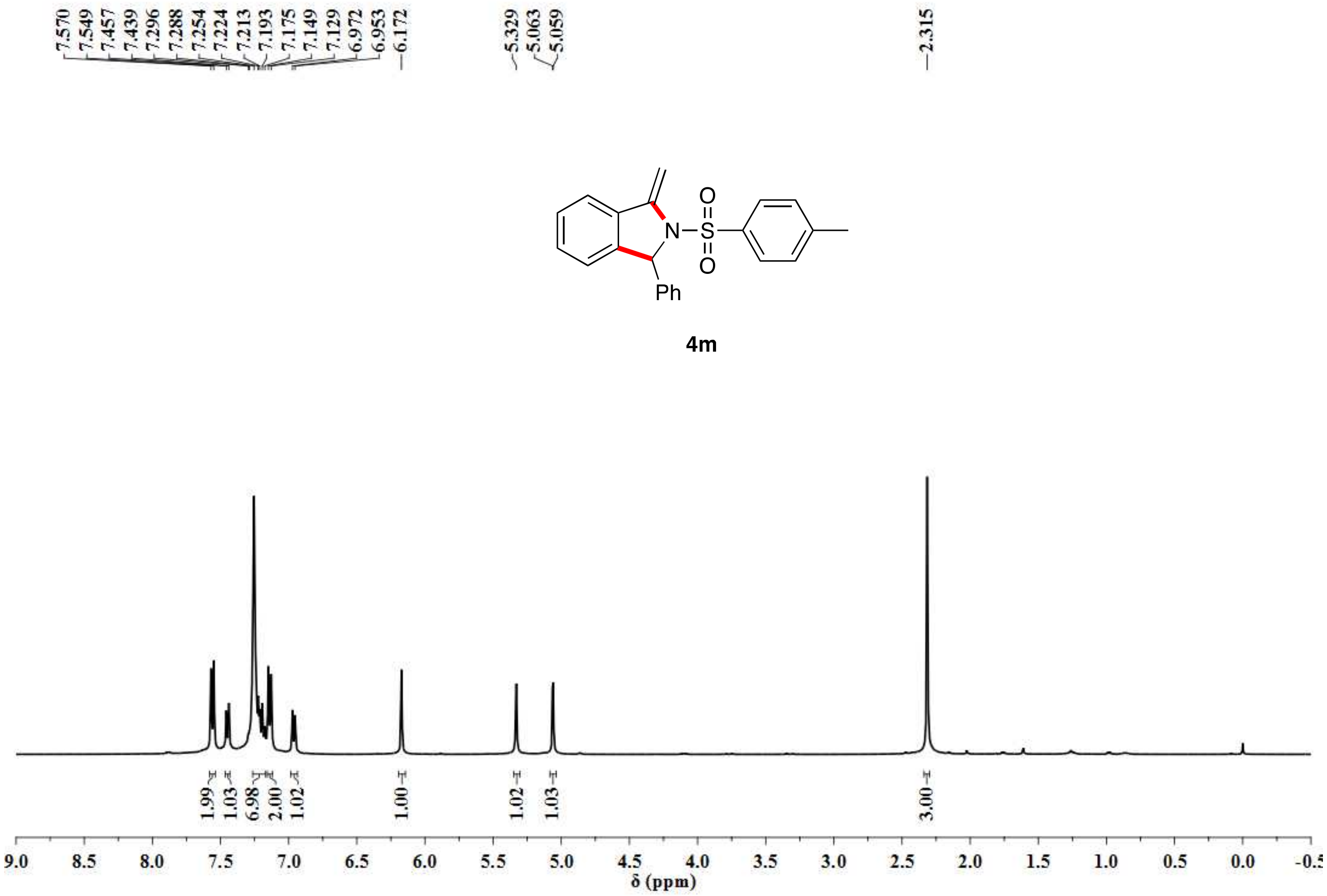

b

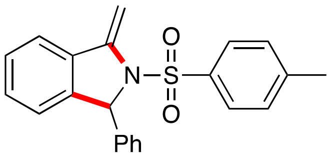
O
N
S
O
Ph

4m

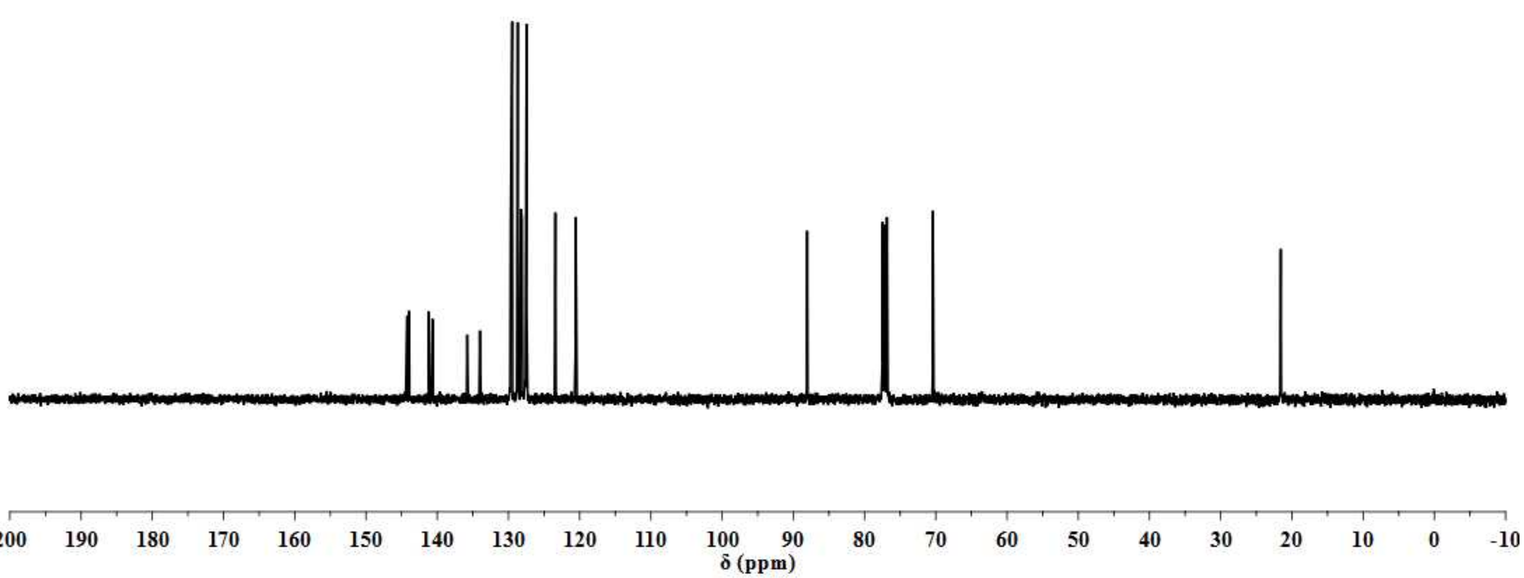
144.217
143.933
141.159
140.664
135.794
134.006
129.699
129.478
128.697
128.254
128.100
127.531
127.451
123.405
120.549
88.051
77.477
77.160
76.842
70.399
21.571
200 190 180 170 160 150 140 130 120 110 100 90 80 70 60 50 40 30 20 10 0 -10
δ (ppm)

Supplementary Figure 77. Characterization of product 5a. (a) ^{1}HNMR spectrum. (b) ^{13}C NMR spectrum.

a

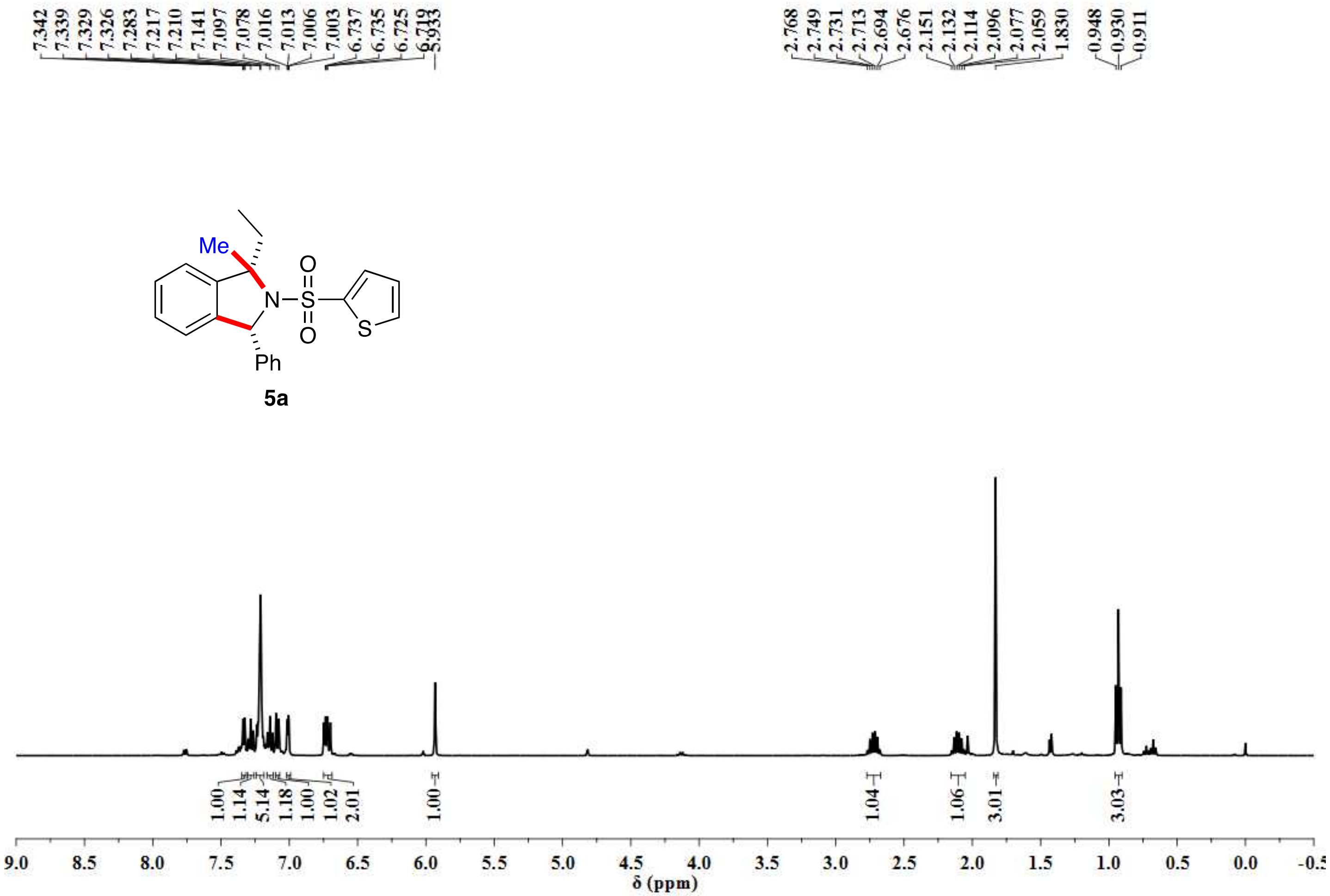

b

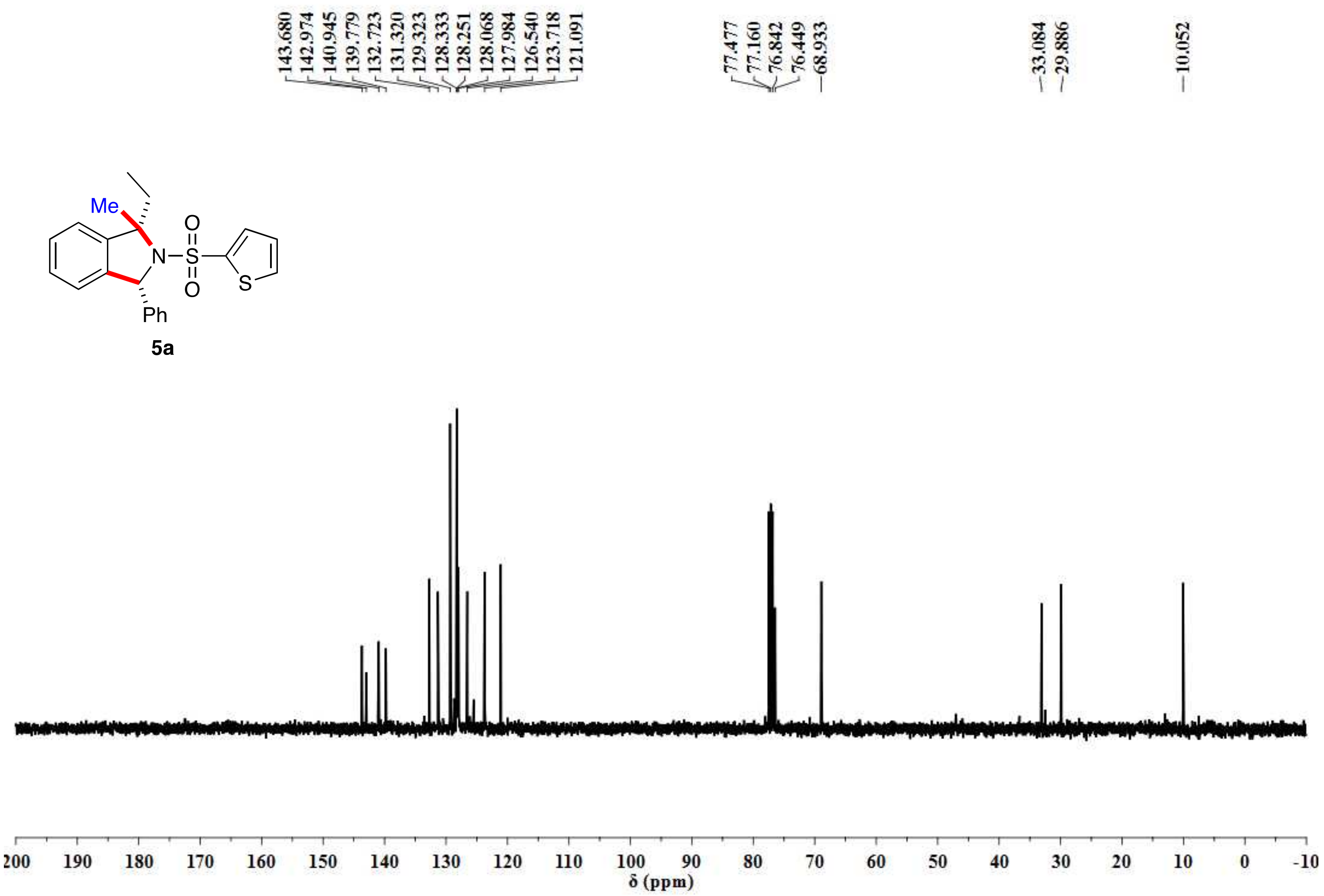

143.680
142.974
140.945
139.779
132.723
131.320
129.323
128.333
128.251
128.068
127.984
126.540
123.718
121.091
77.477
77.160
76.842
76.449
68.933
33.084
29.886
10.052
Me
N
S
O
O
S
Ph
200 190 180 170 160 150 140 130 120 110 100 90 80 70 60 50 40 30 20 10 0 -10
δ (ppm)

5a

Supplementary Figure 78. Characterization of product 5b. (a) ^{1}HNMR spectrum. (b) ^{13}C NMR spectrum.

a

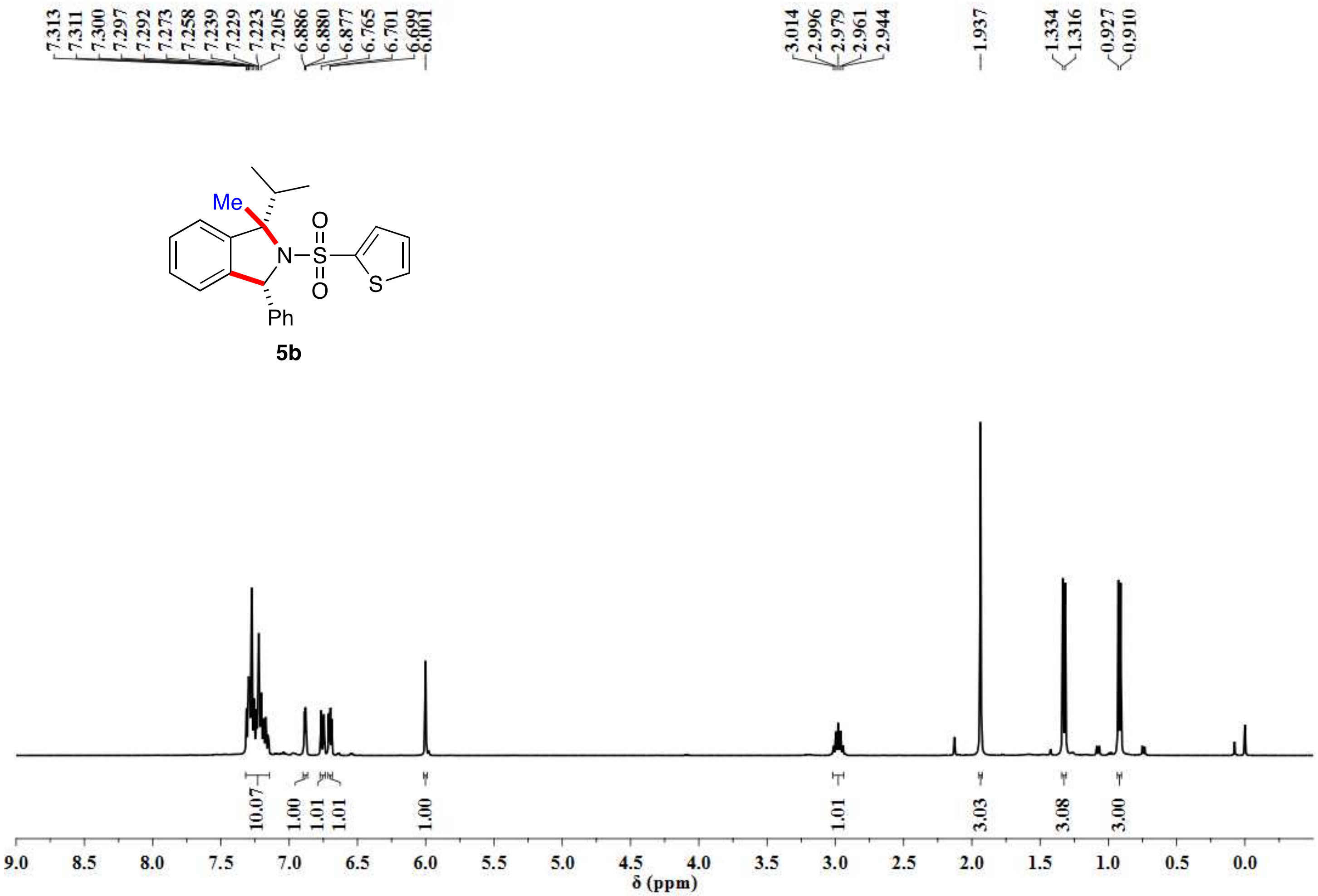

b

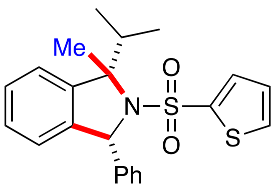

Me
N
S
O
O
S
Ph

5b

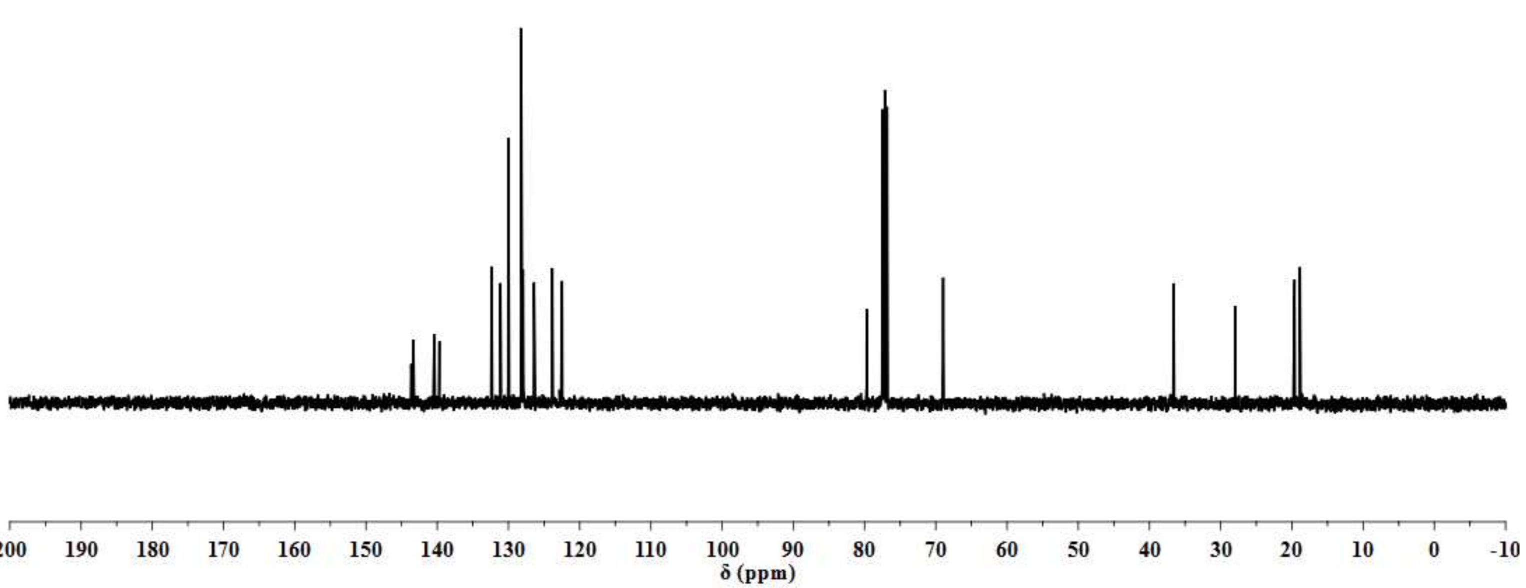

143.654
143.346
140.415
139.690
132.347
131.125
130.019
128.209
127.959
127.896
126.407
123.838
122.464
79.703
77.478
77.160
76.842
68.964
36.595
27.980
19.632
18.867
200 190 180 170 160 150 140 130 120 110 100 90 80 70 60 50 40 30 20 10 0 -10
δ (ppm)

Supplementary Figure 79. Characterization of product 5c. (a) ^{1}HNMR spectrum. (b) ^{13}C NMR spectrum.

a

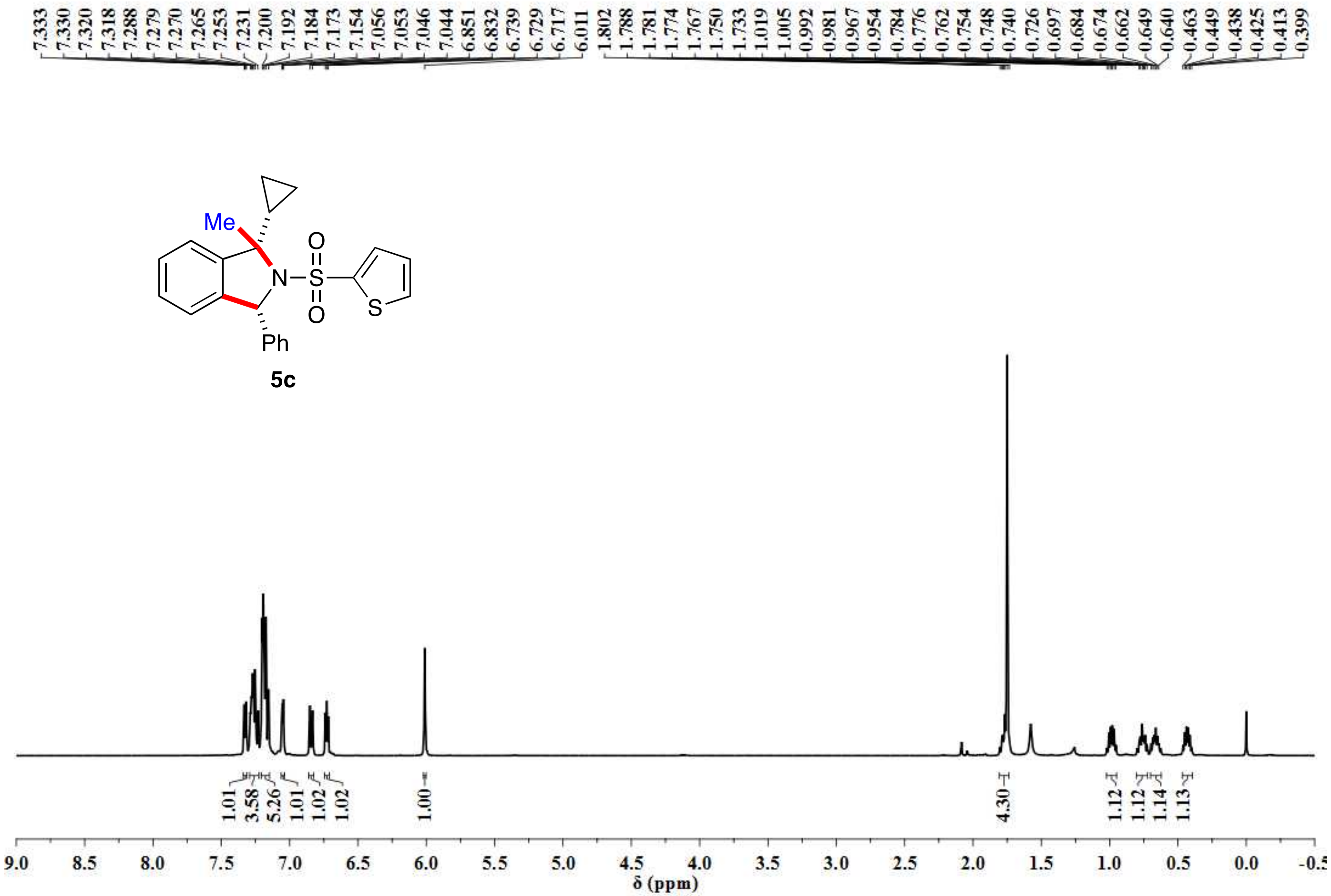

b

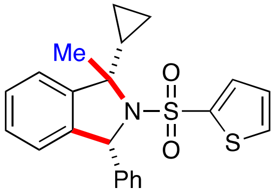
Me
N
S
O
O
S
Ph

5c

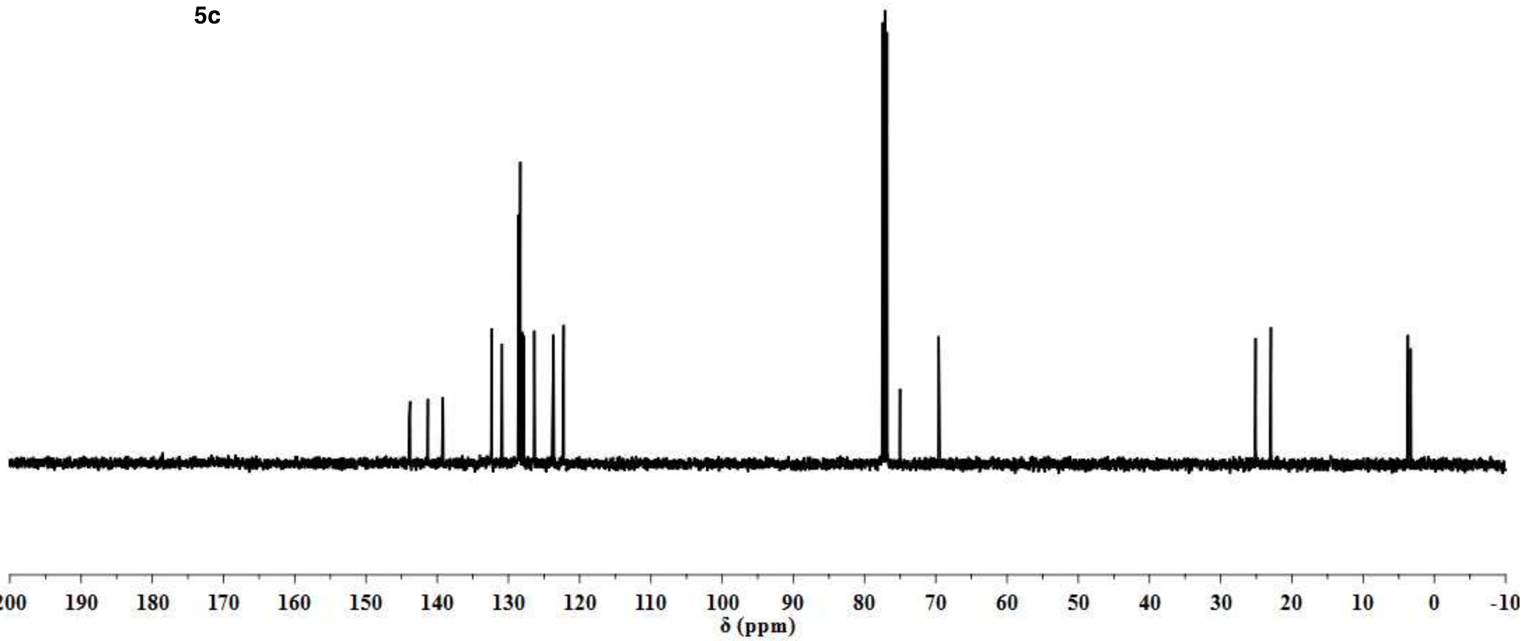
143.970
143.815
141.299
139.189
132.380
130.912
128.571
128.327
128.006
127.832
126.367
123.665
122.239
77.477
77.160
76.842
75.027
69.597
25.104
22.945
3.703
3.310
200 190 180 170 160 150 140 130 120 110 100 90 80 70 60 50 40 30 20 10 0 -10
δ (ppm)

Supplementary Figure 80. Characterization of product 5d. (a) ^{1}HNMR spectrum. (b) ^{13}C NMR spectrum.

a

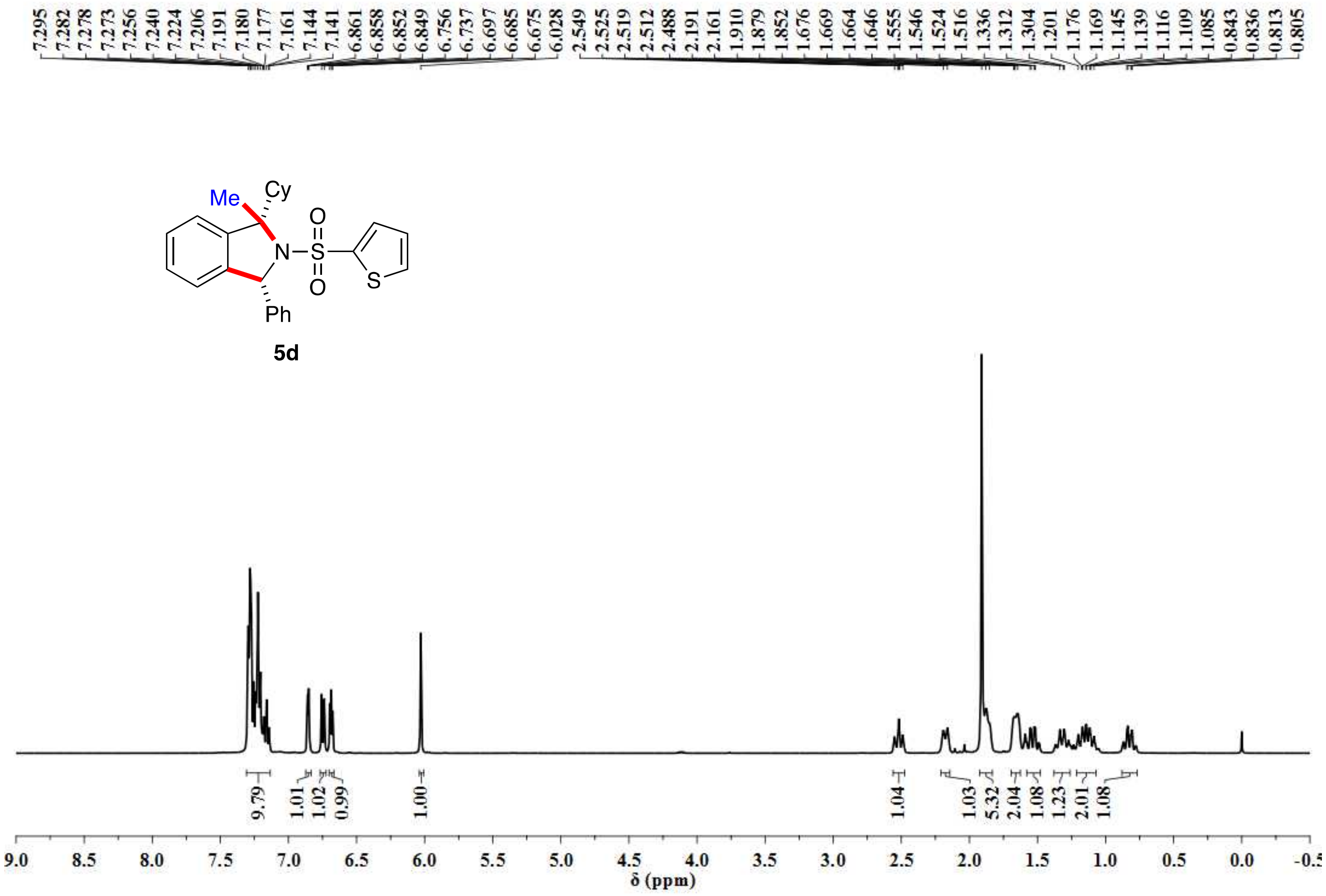

b

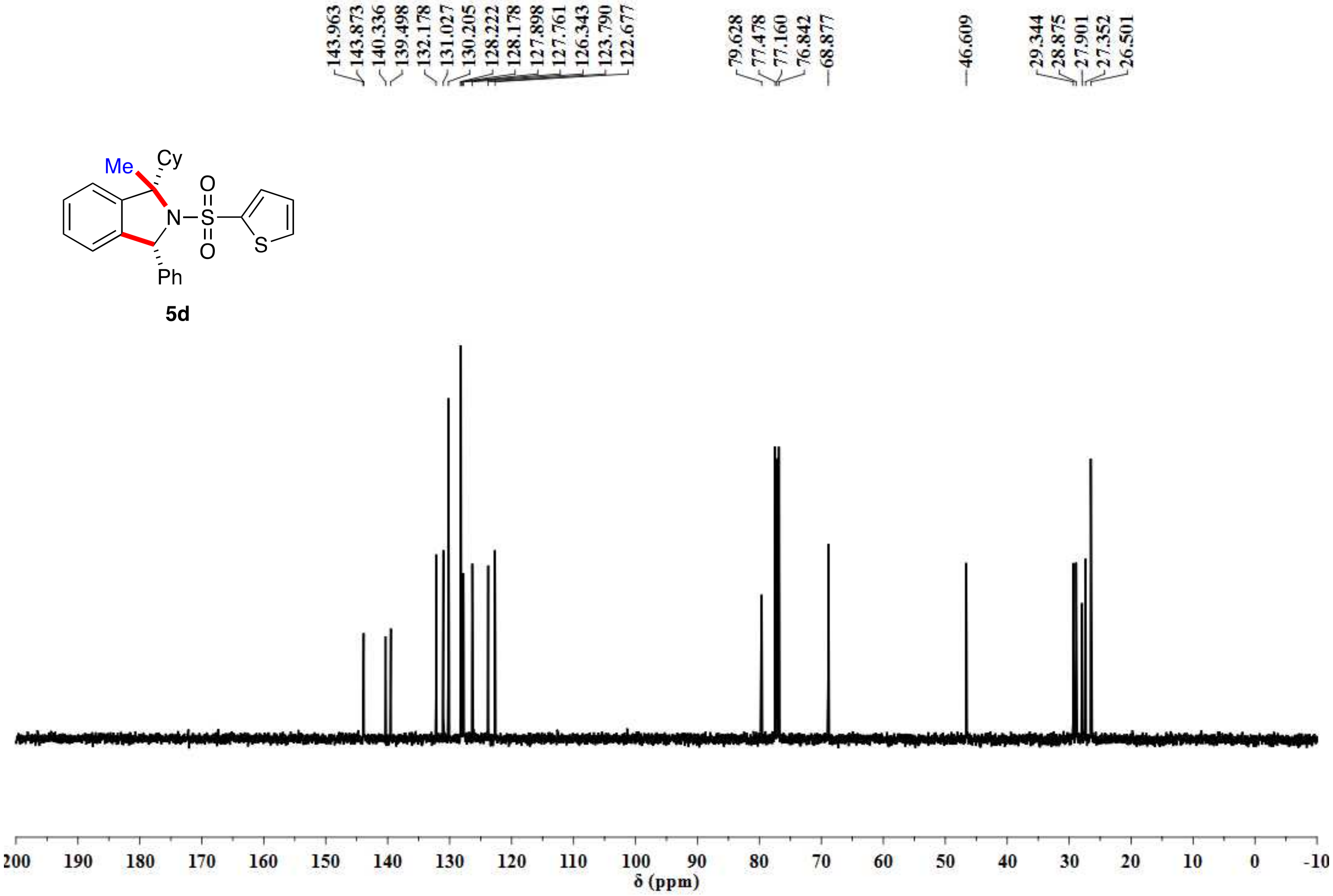
Me
Cy
N
S
O
O
S
Ph
143.963
143.873
140.336
139.498
132.178
131.027
130.205
128.222
128.178
127.898
127.761
126.343
123.790
122.677
79.628
77.478
77.160
76.842
68.877
46.609
29.344
28.875
27.901
27.352
26.501
200 190 180 170 160 150 140 130 120 110 100 90 80 70 60 50 40 30 20 10 0 -10
δ (ppm)

5d

Supplementary Figure 81. Characterization of product 5e. (a) ^{1}HNMR spectrum. (b) ^{13}C NMR spectrum.

a

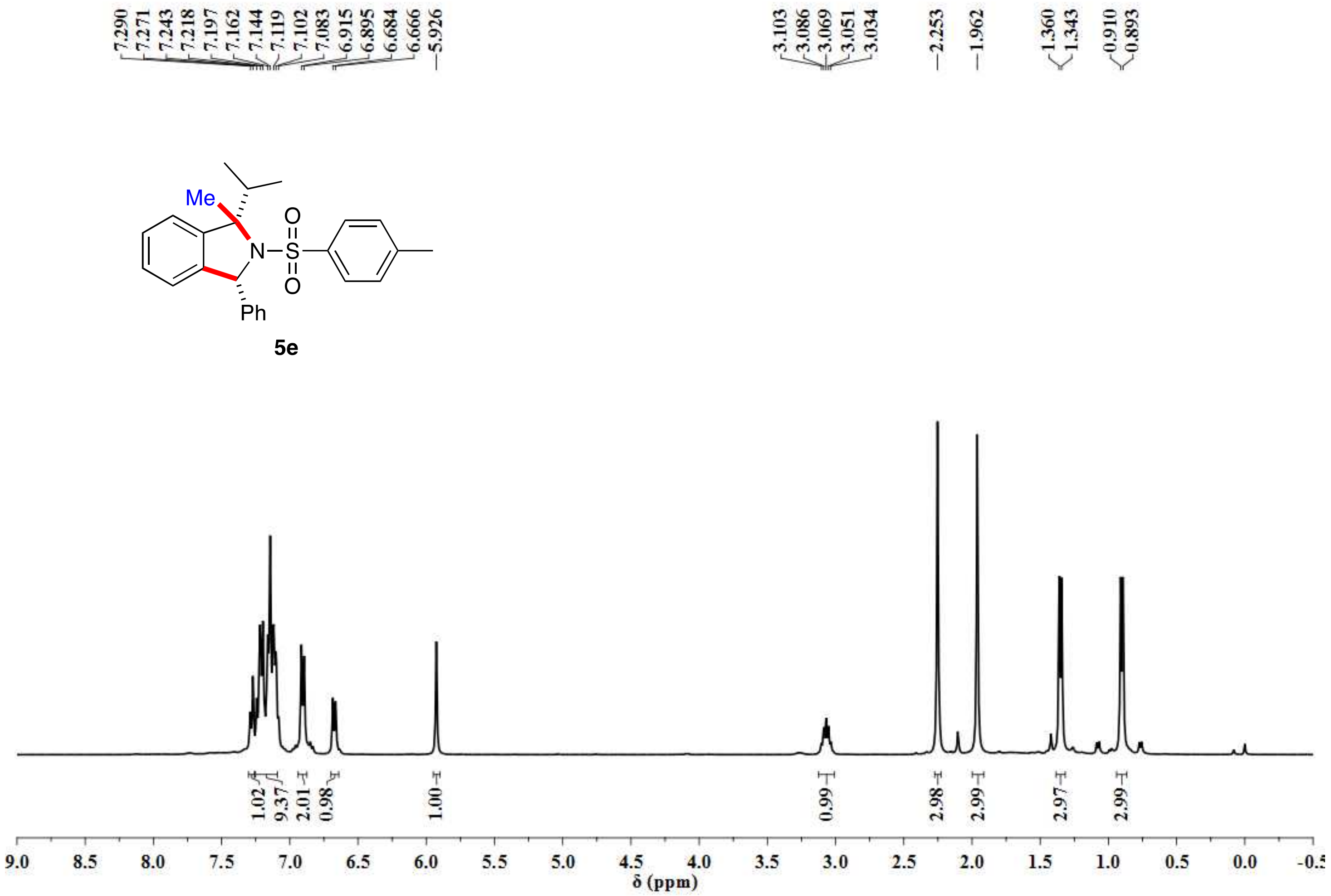

b

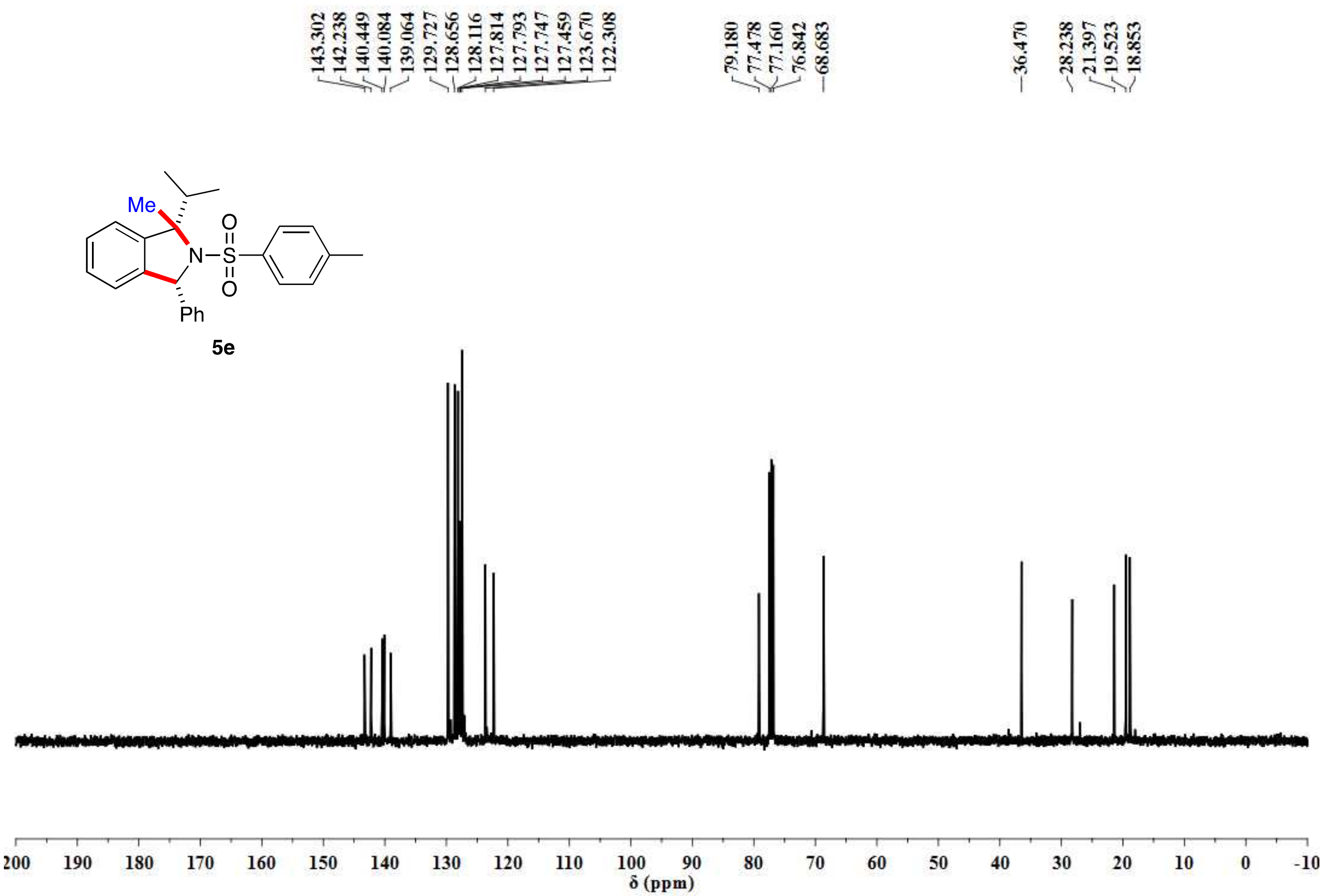
143.302
142.238
140.449
140.084
139.064
129.727
128.656
128.116
127.814
127.793
127.747
127.459
123.670
122.308
79.180
77.478
77.160
76.842
68.683
36.470
28.238
21.397
19.523
18.853
Me
N
S
O
O
Ph
5e
200 190 180 170 160 150 140 130 120 110 100 90 80 70 60 50 40 30 20 10 0 -10
δ (ppm)

Supplementary Figure 82. Characterization of product 5f. (a) ^{1}HNMR spectrum. (b) ^{13}C NMR spectrum.

a

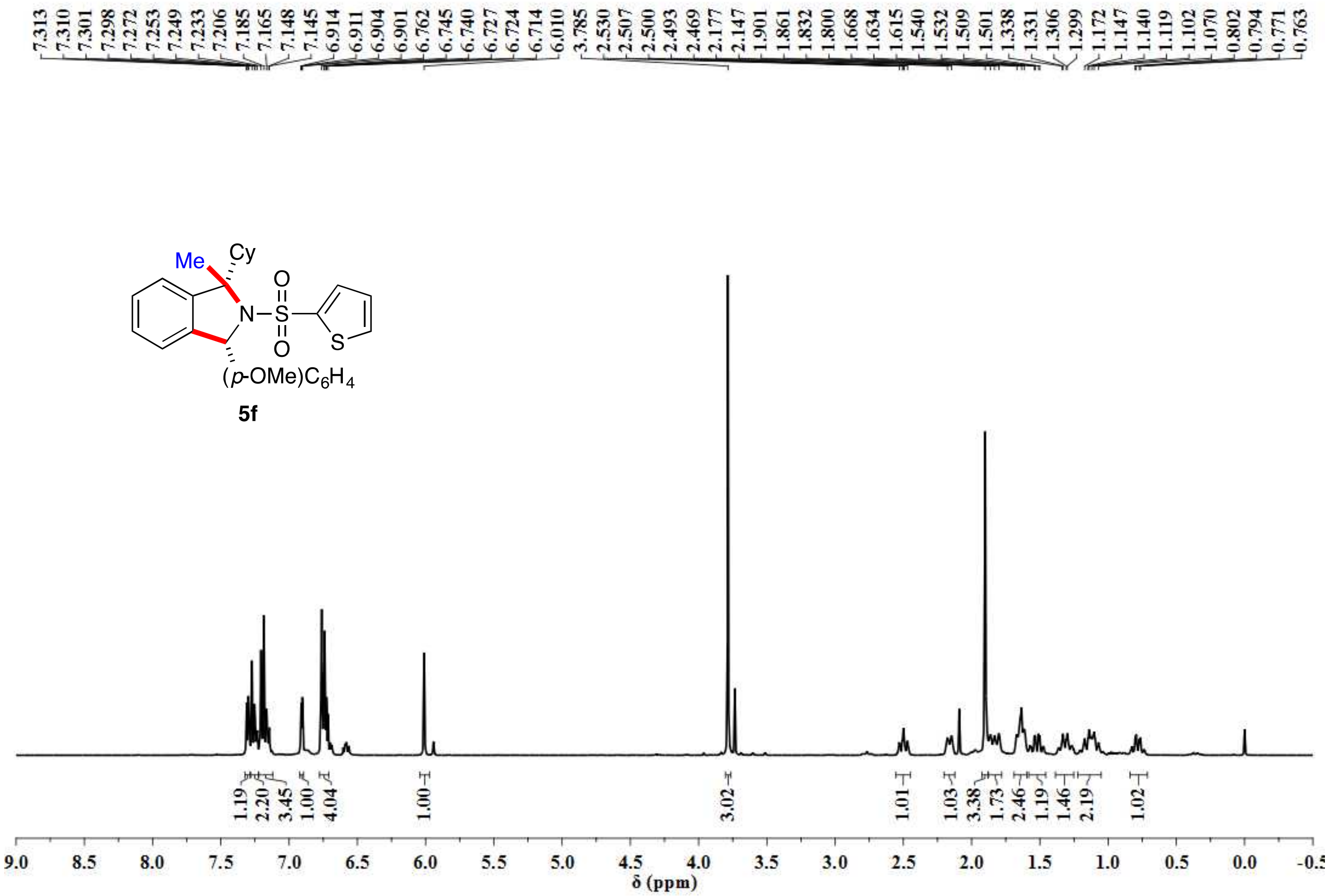

b

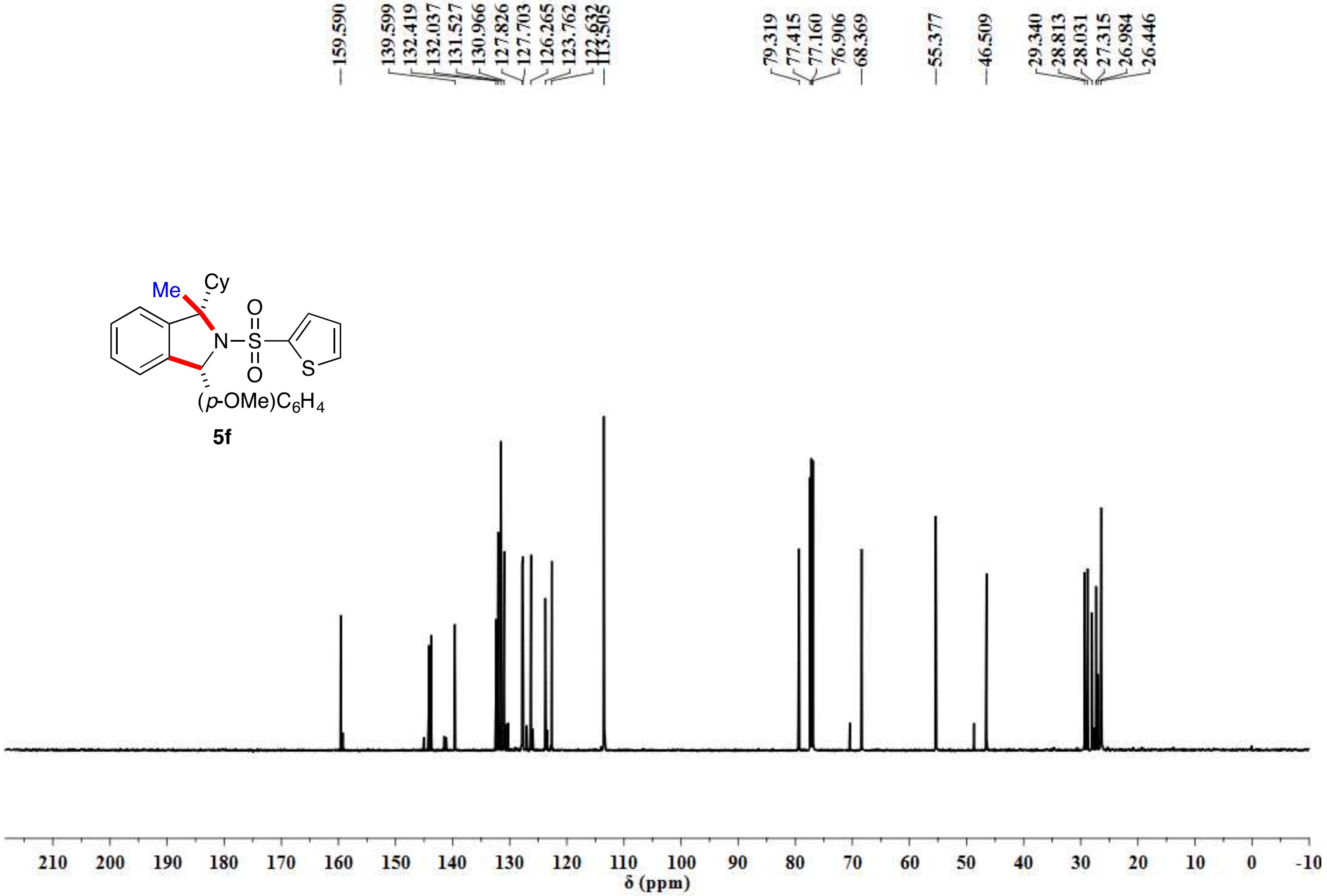

159.590
139.599
132.419
132.037
131.527
130.966
127.826
127.703
126.265
123.762
122.632
113.505
79.319
77.415
77.160
76.906
68.369
55.377
46.509
29.340
28.813
28.031
27.315
26.984
26.446
Me
Cy
N
S
O
O
S
(p-OMe)C6H4
5f
210
200
190
180
170
160
150
140
130
120
110
100
90
80
70
60
50
40
30
20
10
0
-10
δ (ppm)

Supplementary Figure 83. Characterization of product 3o. (a) ^{1}HNMR spectrum. (b) ^{13}C NMR spectrum. (c) ^{19}F-NMR spectrum.

a

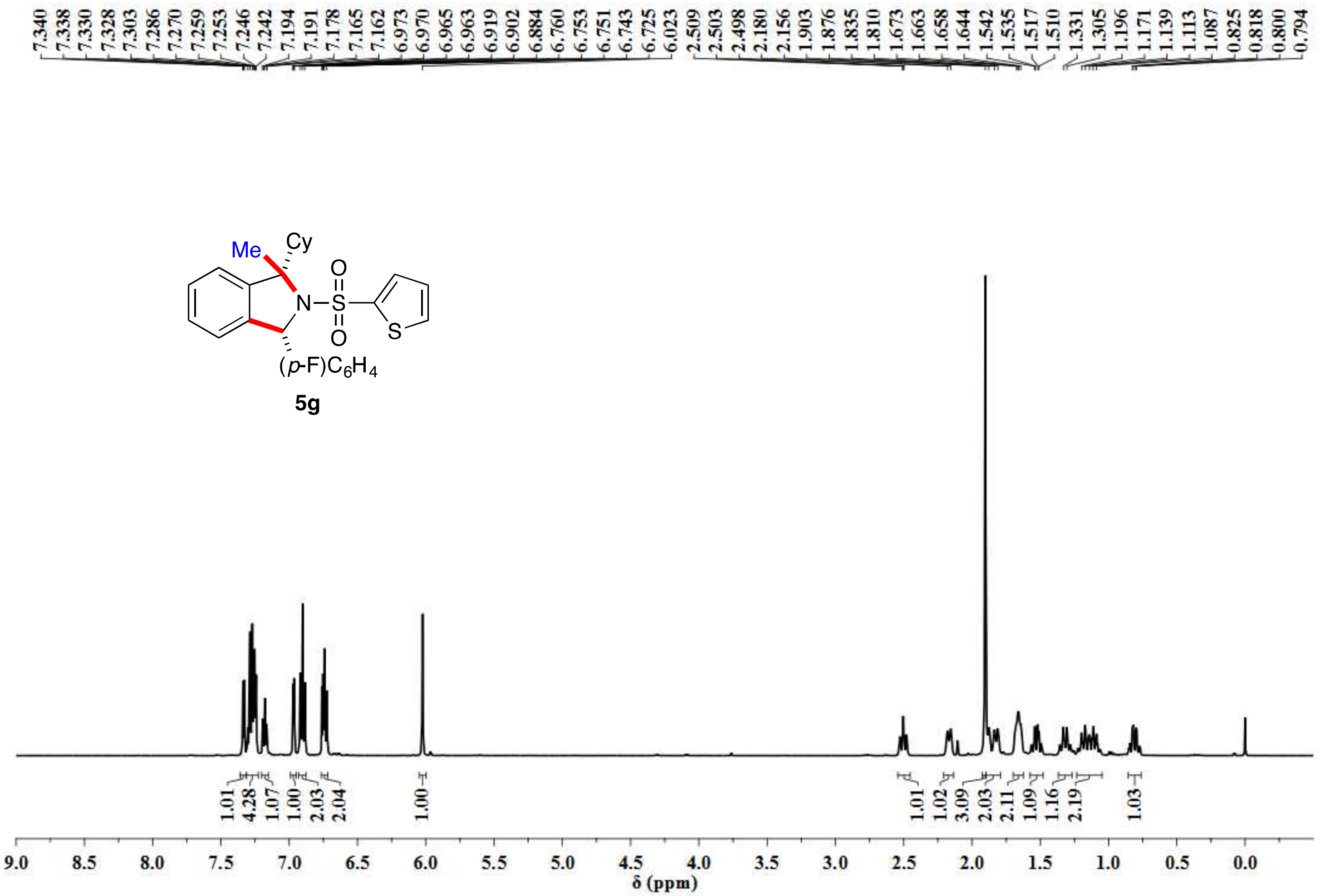

b

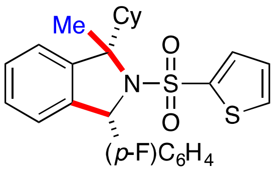
Me
Cy
N
S
O
O
S
(p-F)C6H4

5g

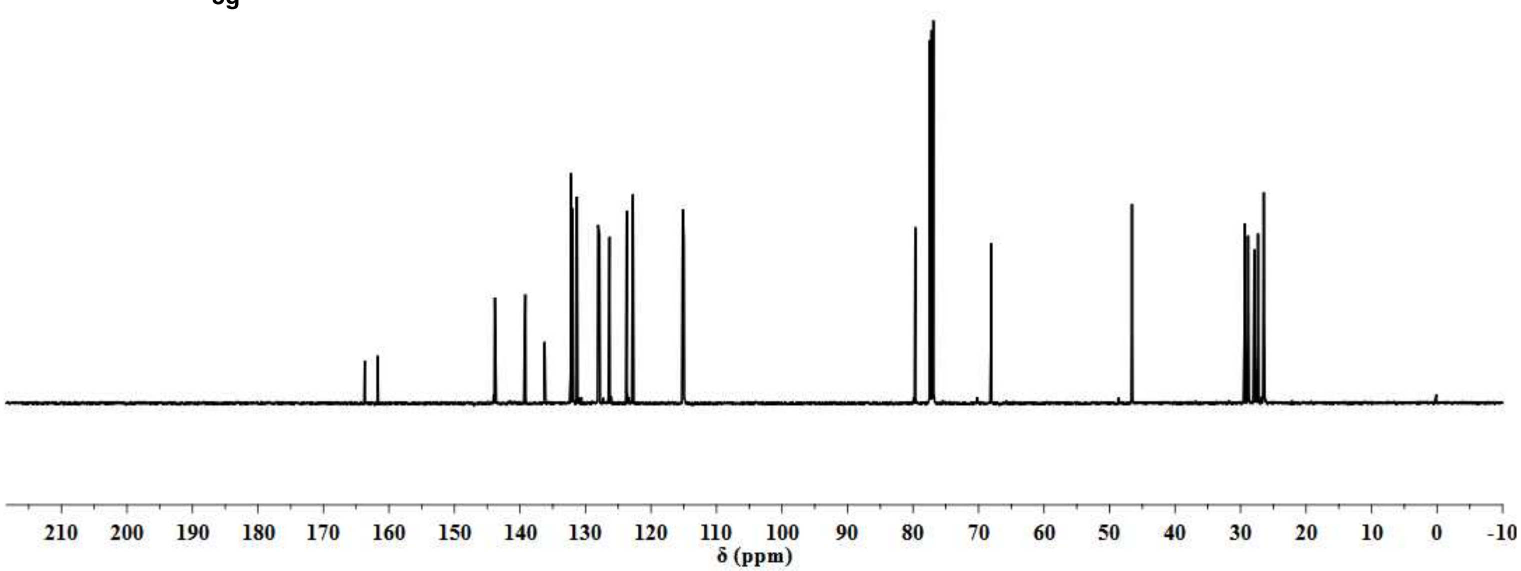
163.684
161.719
143.981
143.842
139.178
136.266
136.239
132.222
131.969
131.903
131.297
128.086
127.857
126.339
123.706
122.761
115.142
114.972
79.657
77.415
77.160
76.906
68.075
46.577
29.379
28.849
27.893
27.327
26.485
26.462
210 200 190 180 170 160 150 140 130 120 110 100 90 80 70 60 50 40 30 20 10 0 -10
δ (ppm)

c

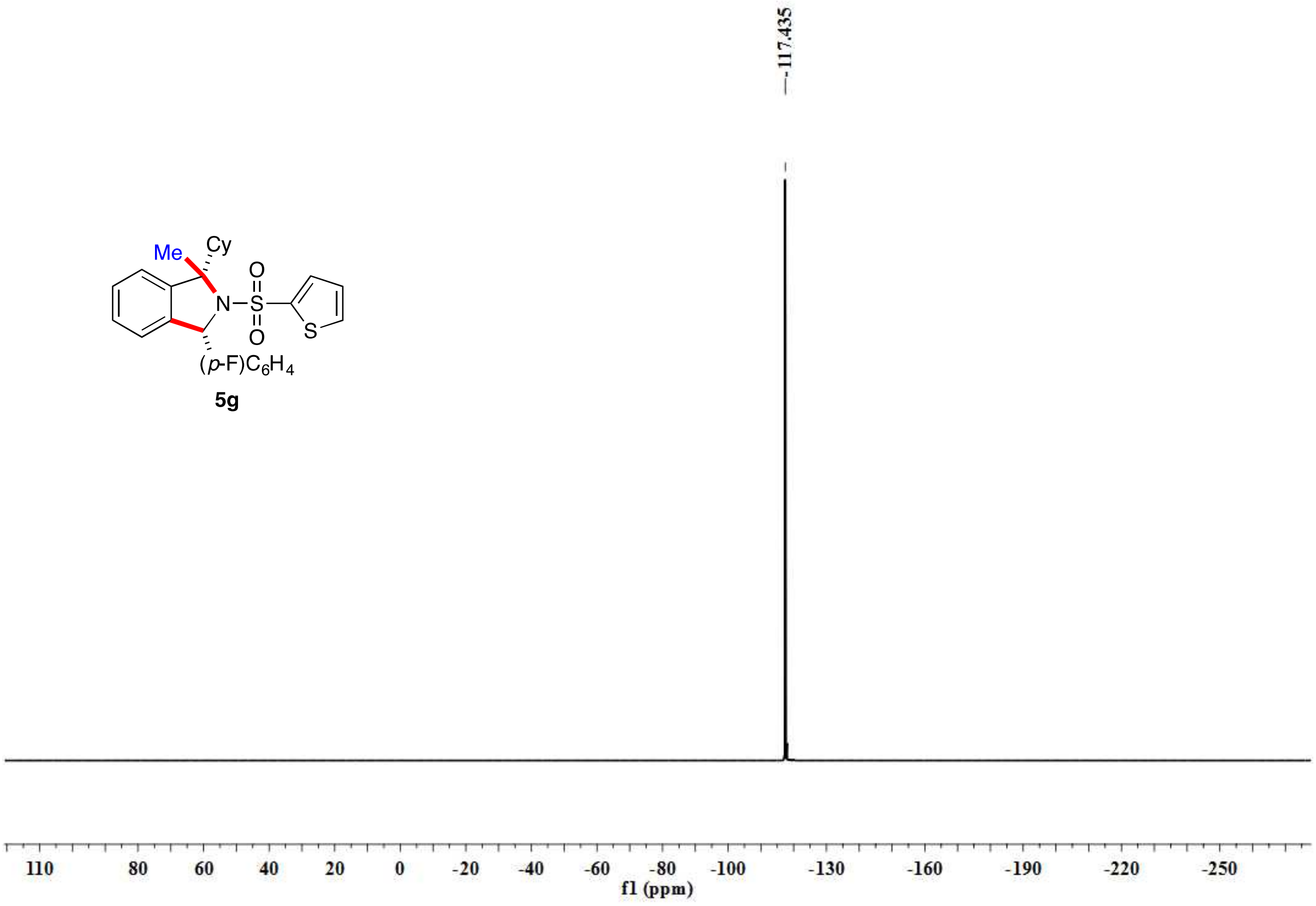
-117.435
Me
Cy
N
S
O
O
S
(p-F)C6H4
5g
110
80
60
40
20
0
-20
-40
-60
-80
-100
-130
-160
-190
-220
-250
f1 (ppm)

Supplementary References

[1] Ushijima, S., Dohi, S., Moriyama, K. & Togo, H. Facile preparation of aromatic ketones from aromatic bromides and arenes with aldehydes. *Tetrahedron* **68**, 1436-1442 (2012).

[2] Mo, F., Trzepkowski, L. J. & Dong, G. Synthesis of *ortho*-acylphenols via Pd-catalyzed ketone-directed hydroxylation of arenes. *Angew. Chem. Int. Ed.* **52**, 13075-13079 (2012).

[3] a) Peyman, A., Hickl, E. & Beckhaus, H.-D. Synthese und thermolyse 1,1′ -disubstituierter *trans*-azoneopentane. -resonanzeffekt und sterischer einfluß der substituenten auf die thermische stabilität sekundärer azoalkane. *Chem. Ber.* **120**, 713-725 (1987). b) Tsuji, Y., Fujio, M. & Tsuno, Y. Substituent effects. 22. The solvolysis of *α*-*t*-butylbenzyl tosylates. *Bull. Chem. Soc. Jpn.* **63**, 856-866 (1990).

[4] Gohier, F., Castanet, A.-S. & Mortier, J. The first regioselective metalation and functionalization of unprotected 4-halobenzoic acids *J. Org. Chem.* **70**, 1501-1504 (2005).

[5] Fujihara, T., Semba, K., Terao, J. & Tsuji, Y. Copper-catalyzed hydrosilylation with a bowl-shaped phosphane ligand: preferential reduction of a bulky ketone in the presence of an aldehyde *Angew. Chem. Int. Ed.* **49**, 1472-1476 (2010).

[6] Kim, H.; Nagaki, A.; Yoshida, J. A flow-microreactor approach to protecting-group-free synthesis using organolithium compounds. *Nat. Commun.* **2**, 264-269 (2011).

[7] Lo Fiego, M. J., Lockhart, M. T. & Chopa, A. B. Catalyst-free alkanoylation of aromatic rings via arylstannanes. Scope and limitations. *J. Organometal. Chem.* **694**, 3674-3678 (2009).

[8] Lauber, M. B. & Stahl, S. S. Efficient aerobic oxidation of secondary alcohols at ambient temperature with an ABNO/NOx catalyst system. *ACS Catal.* **3**,

2612-2616 (2013).

[9] Shibuya, M., Tomizawa, M., Sasano, Y. & Iwabuchi, Y. An expeditious entry to 9-azabicyclo[3.3.1]nonane *N*-oxyl (ABNO): another highly active organocatalyst for oxidation of alcohols *J. Org. Chem.* **74**, 4619-4622 (2009).

[10] Cadamuro, S., Degani, I., Fochi, R., Gatti, A. & Regondi, V. 2-Substituted 1,3-benzoxathiolium tetrafluoroborates as efficient acylating agents for *N,N*-dialkylarylamines. *Synthesis* 544-547 (1986).

[11] Li,H., Zhu, R.-Y., Shi, W.-J., He, K.-H., & Shi, Z.-J. Synthesis of fluorenone derivatives through Pd-catalyzed dehydrogenative cyclization. *Org. Lett.* **14**, 4850-4853 (2012).

[12] Nishino, M., Hirano, K., Satoh, T. & Miura, M. Copper-mediated and copper-catalyzed cross-coupling of indoles and 1,3-azoles: double C-H activation. *Angew. Chem. Int. Ed.* **51**, 6993-6997 (2012).

[13] Zheng, X., Song, B. & Xu, B. Palladium-catalyzed regioselective C–H bond *ortho*-acetoxylation of arylpyrimidines. *Eur. J. Org. Chem.* **23**, 4376-4380 (2010).

[14] Richter, C., Ranganath, K. V. S. & Glorius, F. Enantioselective *α*-arylation of cyclic ketones catalyzed by a combination of an unmodified cinchona alkaloid and a palladium complex. *Adv. Synth. Catal.* **354**, 377-382 (2012).

[15] Dong, D.-J., Li, H.-H. & Tian, S.-K. A highly tunable stereoselective olefination of semistabilized triphenylphosphonium ylides with *N*-sulfonyl imines. *J. Am. Chem. Soc.* **132**, 5018-5020 (2010).

[16] Surmont, R., Verniest, G. & De Kimpe, N. Gold-catalyzed synthesis of 2-aryl-3-fluoropyrroles. *Org. Lett.* **11**, 2920-2923 (2009).

[17] Morimoto, H., Lu, G., Aoyama, N., Matsunaga, S. & Shibasaki, M. Lanthanum aryloxide/pybox-catalyzed direct asymmetric Mannich-type reactions using a trichloromethyl ketone as a propionate equivalent donor. *J. Am. Chem. Soc.* **129**, 9588-9589 (2007).

[18] González, A. S., Arrayás, R. G. & Carretero, J. C. Copper(I)-fesulphos Lewis acid catalysts for enantioselective Mannich-type reaction of *N*-sulfonyl imines. *Org. Lett.* **8**, 2977-2980 (2006).

[19] González, A. S., Arrayás, R. G., Rivero, M. R. & Carretero, J. C. Catalytic asymmetric vinylogous Mannich reaction of *N*-(2-thienyl)sulfonylimines. *Org. Lett.* **10**, 4335-4337 (2008).

[20] Yoshino, T., Ikemoto, H., Matsunaga, S. & Kanai, M. A cationic high-valent Cp^*Co^{III} complex for the catalytic generation of nucleophilic organometallic species: directed C-H bond activation *Angew. Chem. Int. Ed.* **52**, 2207-2221 (2013).

[21] Patel, R., Srivastava, V. P. & Yadav, L. D. S. The first example of saccharin-lithium bromide catalysis: direct synthesis of *N*-tosylimines from alcohols. *Adv. Synth. Catal.* **352**, 1610-1614 (2010).

[22] Zhou, B., Hu, Y. & Wang, C. Manganese-catalyzed direct nucleophilic $C(sp^2)$–H addition to aldehydes and nitriles. *Angew. Chem. Int. Ed.* **54**, 13659-13663 (2015).
